# One‐Step Synthesis of 2,5‐Diaminoimidazoles and Total Synthesis of Methylglyoxal‐Derived Imidazolium Crosslink (MODIC)

**DOI:** 10.1002/anie.201911156

**Published:** 2019-11-12

**Authors:** Venkata R. Sabbasani, Kung‐Pern Wang, Matthew D. Streeter, David A. Spiegel

**Affiliations:** ^1^ Department of Chemistry Yale University 225 Prospect Street New Haven CT 06511 USA

**Keywords:** advanced glycation end products, cyclization, diaminoimidazoles, sigmatropic rearrangement, total synthesis

## Abstract

Here we describe a general method for the synthesis of 2,5‐diaminoimidazoles, which involves a thermal reaction between α‐aminoketones and substituted guanylhydrazines without the need for additives. As one of the few known ways to access the 2,5‐diaminoimidazole motif, our method greatly expands the number of reported diaminoimidazoles and further supports our previous observations that these compounds spontaneously adopt the non‐aromatic 4(*H*) tautomer. The reaction works successfully on both cyclic and acyclic amino ketone starting materials, as well as a range of substituted guanylhydrazines. Following optimization, the method was applied to the efficient synthesis of the advanced glycation end product (AGE) methylglyoxal‐derived imidazolium crosslink (MODIC). We expect that this method will enable rapid access to a variety of biologically important 2,5‐diaminoimidazole‐containing products.

## Graphical abstract

**Whole in one**: A general method was developed for the synthesis of 2,5‐diaminoimidazoles through a thermal reaction between α‐aminoketones and substituted guanylhydrazines. This one‐step reaction works successfully on both cyclic and acyclic amino ketone starting materials, as well as a range of substituted guanylhydrazines. The method was applied to the efficient synthesis of the advanced glycation end product (AGE) methylglyoxal‐derived imidazolium crosslink (MODIC).

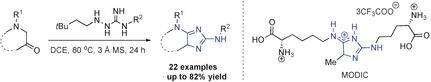

Imidazoles are a common class of heterocycles that are present in various natural products and pharmaceutical compounds.^1^ Indeed, the presence of the imidazole motif in one of the 20 canonical amino acids (histidine) underscores its importance.^2^ Despite the ubiquity of imidazoles, relatively few general methods for their synthesis have been developed.^1^, ^3^, ^4^ In particular, efficient methods to synthesize 2,5‐diaminoimidazoles are notably lacking.^5^ This substructure is a prevalent motif in a number of natural products, including advanced glycation end products^6^, ^7^ (AGEs) **1**–**7** and alkaloids **8**–**11** (Figure 1).^8^ AGEs **1**–**7** are sugar‐derived protein modifications that are widely prevalent among humans and are strongly implicated in diabetic complications,^9^ while alkaloids **8**–**11** exhibit important biological activities.^10^ Indeed, the biological importance and structural complexity of alkaloids **8**–**11** have motivated many synthetic groups to embark on their total syntheses, resulting in several notable successes.^11^, ^12^ Despite these achievements, AGEs **1**–**7** have proven difficult to access, and their biological study has relied on time‐consuming incubation reactions of sugars and amino acids followed by extensive purification to afford the desired materials in low yields.^7^, ^13^

**Figure 1:**
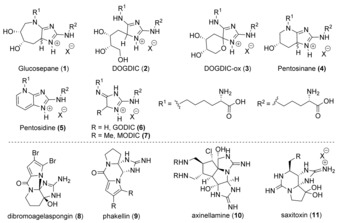
Advanced glycation end products (AGEs) and natural products.

We believe that the efficient synthesis of 2,5‐diaminoimidazoles will greatly facilitate access to these important natural products. To this end, we have developed a one‐step imidazole formation based on a sequence involving condensation, tautomerization, [3,3]‐sigmatropic rearrangement, and cyclodeamination (Scheme 1 A).^14^, ^15^, ^16^

**Scheme 1:**
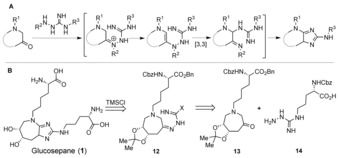
A) A one‐step strategy for imidazole formation. B) Total synthesis of glucosepane, as reported in Ref. 17. TMSCl=chlorotrimethylsilane.

We initially disclosed this method in the context of our total synthesis of the AGE glucosepane **1** (Scheme 1 B).^17^ During the course of this synthesis, a major hurdle arose in the construction of the compound's 2,5‐diaminoimidazole core. This hurdle was overcome using a two‐step route to the 2,5‐diaminoimidazole motif from aminoketone **13** and guanylhydrazine **14**.^17^, ^18^

In this previous work, we then went on to test the scope of this process by performing reactions between both cyclic (**15 a**) and acyclic (**15 b**) aminoketones and guanylhydrazine **16**.^17^ Corresponding guanylhydrazones **17 a** and **17 b** were readily prepared upon gentle heating in methanol. Subjection of **17 a** to heating at 130 °C under microwave irradiation for 16 h in the presence of chlorotrimethylsilane (TMSCl) yielded an inseparable mixture of two regioisomeric imidazoles **18 a** and **19 a**, which were isolated in 33 % yield (Table 1, entry 1). Under the same reaction conditions, **17 b** delivered the diaminoimidazole **18 b** in 12 % yield. Although this route provided the desired non‐aromatic imidazole products **18 a** and **18 b**, it required strongly acidic conditions and high temperatures and gave low yields. Moreover, the formation of the undesired isomer **19 a** necessitated the improvement of these reaction conditions.

**Table 1:** Initial results for the synthesis of 2,5‐diaminoimidazole.^17^

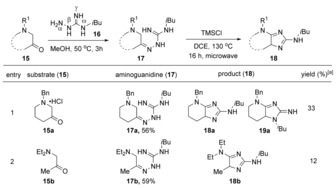

[a] Yields after high‐performance liquid chromatography (HPLC). DCE=1,2‐dichloroethane.

We hypothesized that under these conditions, TMSCl silylates the guanylhydrazone (**17**), thereby driving tautomerization to the enamine, which facilitates [3,3]‐rearrangement. To make the reaction more efficient, we sought to alleviate the need for TMSCl by employing a sacrificial substituent R^2^ on the α‐nitrogen of guanylhydrazine **20**. We hypothesized that pre‐installation of this substituent would not only favorably shift the tautomeric equilibrium, but would also promote the reaction to form the imidazole product (**18**) in one‐step, without the need for additives (Table 2).

**Table 2:** Optimization of reaction condition.

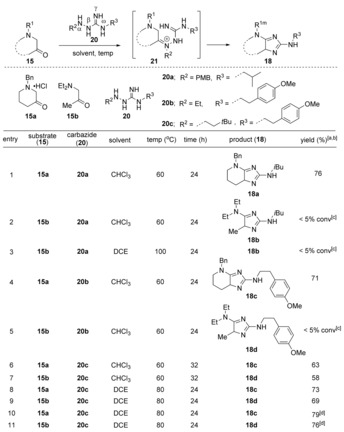

[a] Reaction conditions: 0.60 mmol of **15** and 0.12 mmol of **20** was used. [b] Yields after HPLC. [c] Observed by LC‐MS. [d] 3 Å molecular sieves were added.

To test this hypothesis, we treated cyclic ketone **15 a,** with PMB‐substituted guanylhydrazine **20 a** in CHCl_3_ at 60 °C. Consistent with our hypothesis, the reaction provided the 2,5‐diaminoimidazole **18 a** in 76 % yield (Table 2, entry 1). The formation of imidazole **18 a** in one step increased the reaction efficiency by avoiding the formation of unwanted isomer (**19 a**) under the mild reaction conditions. Exposure of acyclic ketone **15 b** to **20 a** under identical reaction conditions, however, led to almost complete recovery of the starting materials, even after heating at 100 °C in DCE (entries 2 and 3). Changing the PMB subunit of **20 a** to an ethyl substituent (**20 b**) showed a similar reaction profile with **15 a** and **15 b**; **15 a** afforded the imidazole product (**18 c**), but no reaction occurred with **15 b** (entries 4 and 5).

We believed that the guanylhydrazine **20** successfully condensed onto acyclic aminoketone **15 b**, forming enamine intermediate **22** (observed by LC/MS); however, because of the relatively modest steric bulk of the guanylhydrazine N(α)‐ethyl substituent, we speculated that the guanidino group would be facing away from the enamine, thus not fulfilling the conformational requirement for successful [3,3]‐sigmatropic rearrangement (Figure 2).^19^ Based on this analysis, we hypothesized that we could shift the conformational equilibrium toward the desired orientation by appending a bulky alkyl substituent at N(α) position of the guanylhydrazine.^20^ As shown in Figure 2, we expected this bulky substituent to destabilize unproductive conformations **23 a** and **23 b**, and thereby favor conformation **23 c** required for [3,3]‐rearrangement.

**Figure 2:**
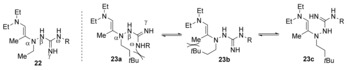
Plausible conformers of tautomerized iminium ion.

Indeed, use of 3,3‐dimethylbutyl‐substituted guanylhydrazine **20 c** enabled both cyclic and acyclic aminoketones **15 a** and **15 b** to participate in the reaction sequence and afforded imidazole products **18 c** and **18 d** in 63 % and 58 % yields, respectively (Table 2, entries 6 and 7). Changing the solvent from CHCl_3_ to DCE and increasing the reaction temperature to 80 °C increased the yields of products **18 c** and **18 d** to 73 % and 69 %, respectively (entries 8 and 9). Adding molecular sieves to the reaction mixture further improved yields to 79 % and 76 % for **18 c** and **18 d**, respectively (entries 10 and 11), likely by accelerating the formation of iminium ion intermediate **21**.

With this optimized one‐step protocol in hand, we examined the reactivity of a number of cyclic α‐aminoketones (Table 3, entries 1–8). We probed the influence of the 3‐piperidinone nitrogen substituents by comparing the reactivity of *N*‐ethyl‐3‐piperidinone (**15 e**) to 3‐piperidinone (**15 f**). Both substrates provided 4(*H*)‐imidazole products **18 e** and **18 f** in 73 % and 66 % yields, respectively (entries 1 and 2). Substrates with the aminoketones masked with carboxylate groups such as Cbz (**15 g**) and Boc (**15 h**) provided 1(*H*)‐imidazole products **18 g** and **18 h** in yields of 69 % and 64 %, respectively (entries 3 and 4). In line with our previous calculations,^17^, ^21^ compounds **18 g** and **18 h** existed as 1(*H*)‐imidazoles, thus highlighting the importance of the substituent at position 5 of the imidazole in stabilizing the 1(*H*)‐imidazole tautomer. The proximal methyl group of piperidinone **15 i** did not greatly affect the reaction profile, since imidazole **18 i** was obtained in 68 % yield (entry 5).

**Table 3:** Ketone scope of the optimized reaction.

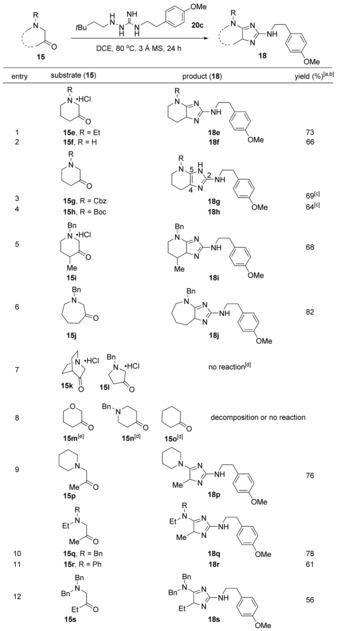

[a] Reaction conditions: 0.12 mmol of **20 c**, 0.36–0.60 mmol of **15** and 10 mg of 3 Å MS. [b] Yields after HPLC. [c] Imidazoles **18 g** and **18 h** were purified by silica‐gel column chromatography. [d] Most of the starting material was recovered. [e] Decomposed under the reaction conditions.

The reaction of azepane **15 j** afforded 4(*H*)‐imidazole **18 j** in excellent yield (Table 3, entry 6). In contrast, bridged α‐aminoketone **15 k** and 3‐pyrollidinone **15 l** did not provide any product under the reaction conditions (entry 7), likely due to strained ring systems present in the reaction intermediates.^22^ Interestingly, the reaction did not proceed for α‐keto ether **15 m**, β‐aminoketone **15 n**, or cyclohexanone **15 o** (entry 8), which reflects the need for an α‐amino substituent in the ketone substrates for this process. We believe that the lack of reactivity for the substrates shown in entry 8 stems from insufficient electron donation from the α‐carbonyl substituent, which is required to facilitate the [3,3]‐sigmatropic rearrangement.

Next, we examined a range of acyclic aminoketone substrates by varying the substituents on the aminoketone (Table 3, entries 9–12). Piperidinyl acetone **15 p** afforded product **18 p** in 76 % yield (entry 9). Aminoacetones **15 q** and **15 r** provided the products **18 q** and **18 r** in 78 % and 61 % yields, respectively (entries 10 and 11). The reaction with **15 s** also generated the imidazole product **18 s** in moderate yield (56 %, entry 12).

Having demonstrated the successful reaction of both cyclic and acyclic aminoketones, we next examined the scope with respect to the guanylhydrazine component by varying the guanidinyl ω‐nitrogen substituents **20**. These guanylhydrazines were tested against representative cyclic and acyclic α‐aminoketones (**15 a** and **15 b**, respectively; Table 4). Gratifyingly, the mono‐substituted guanylhydrazine **20 d** smoothly participated in the reaction with both **15 a** and **15 b** to provide imidazole products **18 t** and **18 u** in 73 % and 53 % yields, respectively (entry 1). Allyl‐ and sterically hindered piperidine‐guanylhydrazines **20 e** and **20 f** also afforded the imidazole products **18 v**–**18 y** in good yields with aminoketones **15 a** and **15 b** (entries 2 and 3). Guanylhydrazine **20 g**, furnished the imidazole products **18 z** and **18 aa** in 53 % and 51 % yields, respectively (entry 4).

**Table 4:** Guanylhydrazine scope for the optimized reaction.

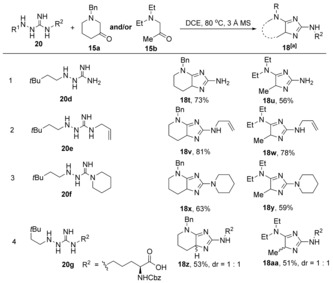

[a] Yields after HPLC.

Methylglyoxal‐derived imidazolium crosslink (MODIC, **7**) is an AGE formed in humans through a reaction between arginine and lysine side chains and one equivalent of methylglyoxal.7c This modification is believed to be involved in end‐stage renal disease processes in diabetic patients.6f Furthermore, MODIC has been shown to be one of the most prevalent AGEs found in bakery products and other common foodstuffs.6g Studies suggest that a high intake of AGE‐containing foods and beverages over time could promote oxidative stress and degenerative changes in different tissues and contribute to disease.6g Despite the correlation between MODIC and disease, to date, detailed investigations into the mechanisms through which MODIC contributes to pathogenesis have been hampered by a scarcity of material available for study. Thus, after optimization of the [3,3]‐sigmatropic rearrangement/cyclization sequence, we pursued a total synthesis of this chemically interesting and biologically important crosslink (Scheme 2).

**Scheme 2:**
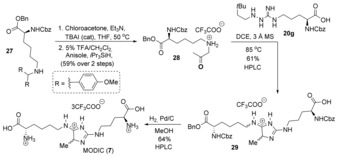
Total synthesis of MODIC. TBAI=tetrabutylammonium iodide, THF=tetrahydrofuran.

The total synthesis of **7** commenced with nucleophilic substitution of lysine **27** to chloroacetone, followed by acidic deprotection of the resulting tertiary amine. This provided aminoketone **28** in 59 % yield over two steps. The rearrangement/cyclization sequence between **28** and **20 g** induced the formation of 4(*H*)‐imidazole **29** in 61 % yield. Global hydrogenolytic removal of the benzyl and carboxybenzyl ester protecting groups using palladium on carbon under an atmosphere of hydrogen gas enabled rapid access to **7** after HPLC purification, as either the trifluoroacetate or formate salts. Spectral data obtained from ^1^H and ^13^C NMR experiments using synthetic **7** were identical to those reported by Lederer and colleagues.7c

In summary, we have developed an efficient synthesis of 2,5‐diaminoimidazoles from α‐aminoketones and guanylhydrazines under mild reaction conditions and without the need for additives. The key feature of this method is the use of a sacrificial alkyl amine on the guanidine, which facilitates a [3,3]‐sigmatropic rearrangement/cyclization sequence with an enamine generated in situ. This method enables access to a wide range of 2,5‐diaminoimidazoles, including the advanced glycation end product MODIC in 23 % overall yield over four steps. Notably, this work represents the first total synthesis of MODIC. Access to this AGE in chemically homogeneous form could lead to useful tools for studying MODIC′s role in biological processes,^23^ as well as potential therapeutic interventions for diseases related to its formation. The utility of this strategy for synthesizing a wide range of biologically important imidazoles, AGEs, and other natural products is also under active investigation.

## Conflict of interest

The authors declare no conflict of interest.

## Supporting information

As a service to our authors and readers, this journal provides supporting information supplied by the authors. Such materials are peer reviewed and may be re‐organized for online delivery, but are not copy‐edited or typeset. Technical support issues arising from supporting information (other than missing files) should be addressed to the authors.

Supplementary
