# Supplementary material for: One‐Step Synthesis of 2,5‐Diaminoimidazoles and Total Synthesis of Methylglyoxal‐Derived Imidazolium Crosslink (MODIC)
Source: Angew Chem Int Ed Engl. 2019 Nov 12;58(52):18913–7. doi: 10.1002/anie.201911156 (PMC6973230; doi:10.1002/anie.201911156)
Supplement: Supplementary file 1 — Supplementary [file ANIE-58-18913-s001.pdf]

Angewandte Chemie
Eine Zeitschrift der Gesellschaft Deutscher Chemiker

Supporting Information

One-Step Synthesis of 2,5-Diaminoimidazoles and Total Synthesis of Methylglyoxal-Derived Imidazolium Crosslink (MODIC)

*Venkata R. Sabbasani, Kung-Pern Wang, Matthew D. Streeter, and David A. Spiegel**

anie_201911156_sm_miscellaneous_information.pdf

INDEX

1. General information for chemical synthesis page S3
2. Synthesis and characterization of guanylhydrazrazines **20a–g** page S3–S7
3. General reaction procedure for 2,5-diaminoimidazoles **18** page S7–S8
4. Characterization of 2,5-diaminoimidazoles **18** page S8–S18
5. Synthesis and characterization of compounds **S6**, **28** and **29** page S18–S20
6. MODIC tris-TFA salt synthesis and characterization page S20
7. MODIC tris-formate salt synthesis and characterization page S21
8. ^{1}H, ^{13}C, ^{13}C-APT, and gHSQC NMR spectra of compound **20a** page S22
9. ^{1}H, ^{13}C, gCOSY, and gHSQC NMR spectra of compound **20b** page S23–S24
10. ^{1}H, ^{13}C, gCOSY, and gHSQC NMR spectra of compound **20c** page S25–S26
11. ^{1}H, ^{13}C, and gCOSY NMR spectra of compound **20d** page S27
12. ^{1}H and ^{13}C NMR spectra of compound **20e** page S28
13. ^{1}H and ^{13}C NMR spectra of compound **20f** page S29
14. ^{1}H, ^{13}C, ^{13}C-APT, gCOSY, and gHSQC NMR spectra of **20g** page S30–S31
15. ^{1}H, ^{13}C, ^{13}C-APT, gCOSY, and gHSQC NMR spectra of **18c** page S32–S33
16. ^{1}H, ^{13}C, ^{13}C-APT, gCOSY, and gHSQC NMR spectra of **18d** page S34–S36
17. ^{1}H, ^{13}C, ^{13}C-APT, gCOSY, and gHSQC NMR spectra of **18e** page S37–S38
18. ^{1}H, ^{13}C, ^{13}C-APT, gCOSY, and gHSQC NMR spectra of **18f** page S39–S341
19. ^{1}H, ^{13}C, ^{13}C-APT, gCOSY, and gHSQC NMR spectra of **18g** page S42–S43
20. ^{1}H, ^{13}C, gCOSY, and gHSQC NMR spectra of **18h** page S44–S45
21. ^{1}H, ^{13}C, ^{13}C-APT, gCOSY, and gHSQC NMR spectra of **18i** page S46–S48
22. ^{1}H, ^{13}C, ^{13}C-APT, gCOSY, and gHSQC NMR spectra of **18j** page S49–S50
23. ^{1}H, ^{13}C, ^{13}C-APT, gCOSY, and gHSQC NMR spectra of **18p** page S51–S52
24. ^{1}H, ^{13}C, ^{13}C-APT, gCOSY, and gHSQC NMR spectra of **18q** page S53–S54
25. ^{1}H, ^{13}C, ^{13}C-APT, gCOSY, and gHSQC NMR spectra of **18r** page S55–S56
26. ^{1}H, ^{13}C, ^{13}C-APT, gCOSY, and gHSQC NMR spectra of **18s** page S57–S58
27. ^{1}H, ^{13}C, ^{13}C-APT, gCOSY, and gHSQC NMR spectra of **18t** page S59–S60
28. ^{1}H, ^{13}C, gCOSY, and gHSQC NMR spectra of **18u** page S61–S62
29. ^{1}H, ^{13}C, ^{13}C-APT, gCOSY, and gHSQC NMR spectra of **18v** page S63–S64
30. ^{1}H, ^{13}C, ^{13}C-APT, gCOSY, and gHSQC NMR spectra of **18w** page S65–S66
31. ^{1}H, ^{13}C, ^{13}C-APT, gCOSY, and gHSQC NMR spectra of **18x** page S67–S68
32. ^{1}H, and ^{13}C NMR spectra of compound **18y** page S69
33. ^{1}H, ^{13}C, gCOSY, and gHSQC NMR spectra of **18z** page S70–S71
34. ^{1}H, ^{13}C, gCOSY, and gHSQC NMR spectra of **18aa** page S72–S73
35. ^{1}H, ^{13}C, gCOSY, and gHSQC NMR spectra of **S6** page S74–S75
36. ^{1}H, ^{13}C, gCOSY, and gHSQC NMR spectra of **28** page S76–S77
37. ^{1}H, ^{13}C, ^{13}C-APT, gCOSY, and gHSQC NMR spectra of **29** page S78–S80
38. ^{1}H, ^{13}C, ^{13}C-APT NMR spectra of MODIC (**7**) tris TFA salt page S81
39. gCOSY, HMBC, gHSQC NMR spectra of MODIC (**7**) tris TFA salt page S82–S83
40. ^{1}H, ^{13}C, ^{13}C-APT NMR spectra of MODIC (**7**) tris FA salt page S84
41. gCOSY, and gHSQC NMR spectra of MODIC (**7**) tris FA salt page S85–S86

General Information for Chemical Synthesis

Starting materials were used as received unless otherwise noted. All moisture sensitive reactions were performed in an inert, dry atmosphere of nitrogen in oven dried glassware. Reagent grade solvents were used for extractions and flash chromatography. 3 Å molecular sieve was activated at 135 °C for 12 hours before use. Reaction progress was monitored by LC-MS analyses performed on a Waters UPLC/MS instrument equipped with a RP-C18 column (1.7 µm particle size, 2.1x50 mm), dual atmospheric pressure chemical ionization (API)/electrospray (ESI) mass spectrometry detector, and photodiode array detector. Flash column chromatography was performed using RediSepRf NP-silica (40-63 µm 60 Å) or Teledyne RediSepRf Gold RP-C18 column (20-40 µm 100 Å) in Teledyne ISCO CombiFlash Rf 200 purification system unless otherwise specified. The solvent compositions reported for all chromatographic separations are on a volume/volume (v/v) zbasis. Infrared (IR) spectra were recorded on a Thermo Nicolet 6700 FT-IR Spectrometer. ^{1}H-NMR spectra were recorded on Agilent DD2 400 MHz, 500 MHz, 600 MHz spectrometer and reported in parts per million (ppm) on the δ scale relative to $CDCl_3$ (δ 7.26), Methanol-*d4* (δ 3.31), ACN-*d3* (δ 1.94), D_2O (δ 4.79) as an internal standard. Data are reported as follows: chemical shift, multiplicity (s = singlet, d = doublet, t = triplet, q = quartet, br = broad, m = multiplet), coupling constants (Hz), and integration. ^{13}C-NMR spectra were recorded on Agilent DD2 125 MHz, and 150 MHz spectrometers and were reported in parts per million (ppm) on the δ scale relative to $CDCl_3$ (δ 77.00), Methanol-*d4* (δ 49.00), ACN-*d3* (δ 1.32).

One-pot General Synthesis of Guanylhydrazrazines

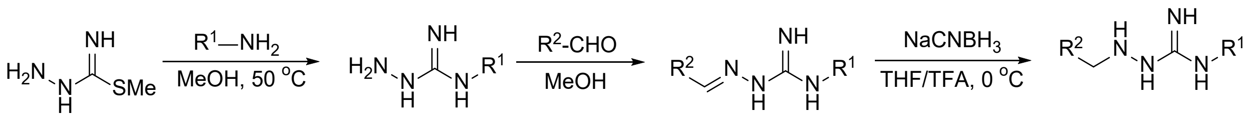

Guanylhydrazrazines **20a–20c**, **20e**, and **20f** were synthesized from methyl hydrazinecarbimidothioate by replacing SMe with corresponding amines followed by condensation with aldehyde, which then reduced with sodium cyanoborohydride.

***N*-isobutyl-2-(4-methoxybenzyl)hydrazine-1-carboximidamide TFA salt (20a):**

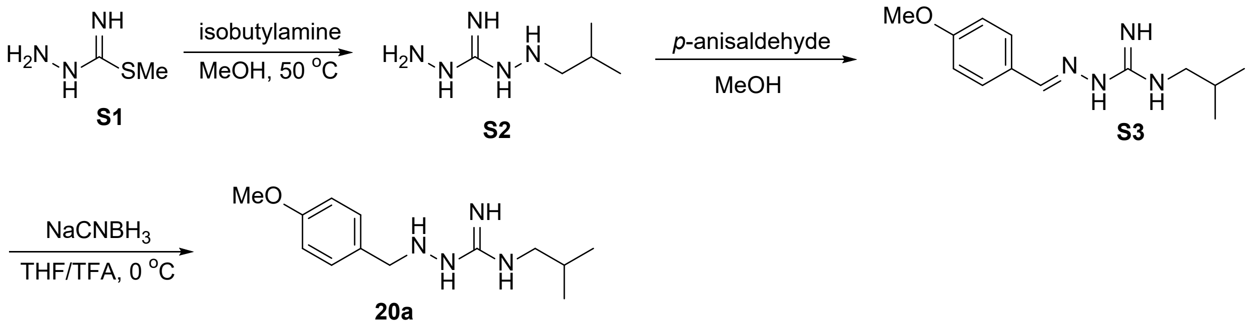

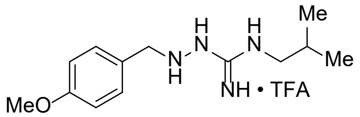

20a

Methyl hydrazinecarbimidothioate hydroiodide **S1** (640 mg, 2.75 mmol) and isobutylamine (241 mg, 3.30 mmol) were dissolved in MeOH (3 mL) and the reaction vessel was capped and heated up to 50 ºC in oil bath for 12 h. After 12h, LC-MS indicated full consumption of the starting material with the formation of the desired carboximidhydrazide **S2**. Solvent and excess amount of the isobutylamine were removed under reduced pressure and the crude *N'*-isobutylhydrazinecarboximidhydrazide **S2** was used directly for the next step without further purification. The hydrazide **S2** was re-dissolved in anhydrous MeOH (3 mL) followed by the addition of the *p*-anisaldehyde (449 mg, 3.30 mmol). The reaction mixture stirred for 4 h at room temperature (monitored by LC-MS). After complete consumption of **S2** the solvent was removed under reduced pressure and the crude product **S3** was re-dissolved in THF (5 mL) and cooled down to 0 ºC. After stirred at 0 ºC for 5 mins, $NaCNBH_3$ (518 mg, 8.25 mmol) was added in three portions, followed by slow addition of TFA (1 mL). The reaction was stirred at the same temperature for 30 mins. LC-MS indicated all starting materials were consumed with the formation of the desired carboximidamide **20a**. Reaction was quenched with water (5 mL) and extracted with DCM (15 mL x 4). Combined organic layers were washed with water, brine and dried over anhydrous Na_2SO_4. Solvent was removed under reduced pressure and the crude product was purified by Teledyne ISCO CombiFlash using RP-C18 column (H_2O and MeCN both buffered with 0.1% trifluoroacetic acid were used as the mobile phase). The combined fractions were lyophilized to obtain TFA salt of **20a** in 63% yield (630 mg, 1.72 mmol, *E/Z* = 10:1) as a white fluffy solid.

Major isomer: ^{1}H NMR (500 MHz, $CDCl_3$) δ 7.22 (d, *J* = 8.2 Hz, 2H), 6.87 (d, *J* = 8.2 Hz, 2H), 6.31 (s, 2H), 4.25 (s, 1H), 3.88 (s, 2H), 3.78 (s, 3H), 2.94 – 2.82 (m, 2H), 1.83 – 1.67 (m, 1H), 0.96 – 0.75 (m, 6H); ^{13}C NMR (126 MHz, $CDCl_3$) δ 159.61, 157.17, 130.47, 127.50, 114.30, 55.33, 55.24, 48.42, 28.04, 19.64.
HR-MS: $(M+H)^+$ = 251.1753 (experimental); exact mass = 251.1866 (theoretical)
IR *f* (cm^{-1}): 3347, 2964, 1649, 1612, 1513, 1466, 1251, 1102.

2-ethyl-*N*-(4-methoxyphenethyl)hydrazine-1-carboximidamide TFA salt (20b):

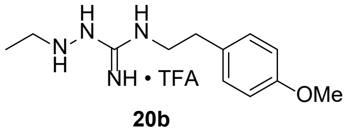

20b

Compound **20b** was synthesized followed the same reaction sequence as compound **20a**. Starting material **S1** (500 mg, 2.14 mmol) was reacted with 2-(4-methoxyphenyl)ethan-1-amine (343 mg, 2.27 mmol) followed by the condensation of the newly formed hydrazine with acetaldehyde (188 mg, 4.28 mmol) to provide the crude hydrazone. Methanol was removed and the crude material was re-dissolved in THF (5 mL) and reduced the hydrazone with $NaCNBH_3$ (807 mg, 807 mg) and TFA (1 mL). Purified the crude product using Teledyne ISCO CombiFlash with RP-C18 column (H_2O and MeCN both buffered with 0.1% trifluoroacetic acid were used as the mobile phase) to provide TFA salt of carboximidamide **20b** (330 mg) in 44 % yield as a white fluffy solid.

Major isomer: ^{1}H NMR (500 MHz, $CDCl_3$) δ 8.58 (s, 1H), 7.12 (d, *J* = 8.2 Hz, 2H), 6.85 (d, *J* = 8.0 Hz, 2H), 6.74 – 6.30 (m, 2H), 3.76 (s, 3H), 3.53 – 3.30 (m, 2H), 2.96 – 2.64 (m, 4H), 1.10 –

0.89 (m, 3H). ^{13}C NMR (126 MHz, $CDCl_3$) δ 158.68, 157.22, 129.78, 128.97, 114.41, 55.33, 46.14, 42.46, 34.08, 12.47.
HR-MS: $(M+H)^+$ = 237.1731 (experimental); exact mass = 237.1710 (theoretical)
IR *f* (cm^{-1}): 3304, 2975, 1643, 1613, 1513, 1247, 1202, 1134,1102.

2-(3,3-dimethylbutyl)-*N*-(4-methoxyphenethyl)hydrazine-1-carboximidamide TFA salt (20c):

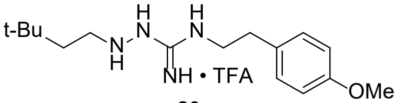

20c

Compound **20c** was synthesized followed the same reaction sequence as compound **20a**. Starting material **S1** (1.00 g, 4.3 mmol) was reacted with 2-(4-methoxyphenyl)ethan-1-amine (0.68 g, 4.5 mmol) followed by the condensation of the newly formed hydrazine with 3,3-dimethylbutanal (0.52 g, 5.16 mmol) to provide the crude hydrazone. Methanol was removed and the crude material was re-dissolved in THF (15 mL) and reduced the hydrazone with $NaCNBH_3$ (1.62 g, 25.8 mmol) and TFA (1 mL). Purified the crude product using Teledyne ISCO CombiFlash with RP-C18 column (H_2O and MeCN both buffered with 0.1% trifluoroacetic acid were used as the mobile phase) to provide the carboximidamide **20c** (900 mg) in 51 % yield as TFA salt.

^{1}H NMR (500 MHz, $CDCl_3$) δ 10.35 (s, 1H), 7.70 (s, 1H), 7.11 (d, *J* = 8.0 Hz, 2H), 6.85 (d, *J* = 8.0 Hz, 2H), 6.36 (s, 1H), 3.78 (s, 3H), 3.62 – 3.03 (m, 2H), 2.83 (t, *J* = 6.6 Hz, 2H), 2.78 – 2.61 (m, 2H), 1.40 – 1.03 (m, 2H), 0.85 (s, 9H). ^{13}C NMR (126 MHz, $CDCl_3$) δ 158.59, 157.47, 129.67, 129.21, 114.27, 55.15, 48.19, 41.71, 41.10, 33.86, 29.52, 29.38.
HR-MS: $(M+H)^+$ = 293.2345 (experimental); exact mass = 293.2336 (theoretical)
IR *f* (cm^{-1}): 3187, 2958, 1646, 1514, 1468, 1249, 1201, 1179, 1140.

***N*-allyl-2-(3,3-dimethylbutyl)hydrazine-1-carboximidamide TFA salt (20e):**

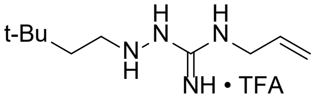

20e

Compound **20e** was synthesized followed the same reaction sequence as compound **20a**. Starting material **S1** (150 mg, 0.65 mmol) was reacted with allylamine (148 mg, 2.60 mmol) followed by the condensation of the newly formed hydrazine with 3,3-dimethylbutanal (78 mg, 0.78 mmol) to provide the crude hydrazone. Methanol was removed and the crude material was re-dissolved in THF (5 mL) and reduced the hydrazone with $NaCNBH_3$ (245 mg, 3.90 mmol) and TFA (1 mL). Purified the crude product using Teledyne ISCO CombiFlash with RP-C18 column (H_2O and MeCN both buffered with 0.1% trifluoroacetic acid were used as the mobile phase) to provide the carboximidamide **20e** (99 mg) in 37% yield as TFA salt.

^{1}H NMR (600 MHz, $CDCl_3$) δ 9.77 (s, 1H), 8.29 (s, 1H), 7.21 (s, 1H), 6.71 (s, 1H), 5.84 (dd, *J* = 20.0, 9.3 Hz, 1H), 5.29 (t, *J* = 13.7 Hz, 2H), 4.00 – 3.62 (m, 2H), 2.95 – 2.67 (m, 2H), 1.47 – 1.21 (m, 2H), 0.89 (s, 9H). ^{13}C NMR (151 MHz, $CDCl_3$) δ 157.66, 131.56, 118.01, 48.33, 43.22, 41.10, 29.62, 29.39.
HR-MS: $(M+H)^+$ = 199.1821 (experimental); exact mass = 199.1917 (theoretical)
IR *f* (cm^{-1}):3288, 2958, 2259, 1638, 1470, 1201,1138, 1103.

N'-(3,3-dimethylbutyl)piperidine-1-carboximidhydrazide TFA salt (20f):

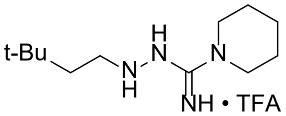

20f

Compound **20f** was synthesized followed the same reaction sequence as compound **20a**. Starting material **S1** (150 mg, 0.65 mmol) was reacted with piperidine (66 mg, 0.78 mmol) followed by the condensation of the newly formed hydrazine with 3,3-dimethylbutanal (78 mg, 0.78 mmol) to provide the crude hydrazone. Methanol was removed and the crude material was re-dissolved in THF (5 mL) and reduced the hydrazone with $NaCNBH_3$ (245 mg, 3.90 mmol) and TFA (1 mL). Purified the crude product using Teledyne ISCO CombiFlash with RP-C18 column (H_2O and MeCN both buffered with 0.1% trifluoroacetic acid were used as the mobile phase) to provide the carboximidamide **20f** (92 mg) in 42% yield as TFA salt.

^{1}H NMR (600 MHz, $CDCl_3$) δ 9.14 (s, 1H), 7.20 (bs, 2H), 3.39 (t, J = 5.2 Hz, 4H), 2.82 – 2.78 (m, 2H), 1.66 – 1.52 (m, 6H), 1.42 – 1.16 (m, 2H), 0.85 (s, 9H). ^{13}C NMR (151 MHz, $CDCl_3$) δ 156.81, 48.25, 46.73, 41.03, 29.55, 29.34, 24.95, 23.54.
HR-MS: $(M+H)^+$ = 227.2119 (experimental); exact mass = 227.2230 (theoretical)
IR f (cm^{-1}):3208, 2954, 2866, 1674, 1637,1200, 1137.

2-(3,3-dimethylbutyl)hydrazine-1-carboximidamide TFA salt (20d):

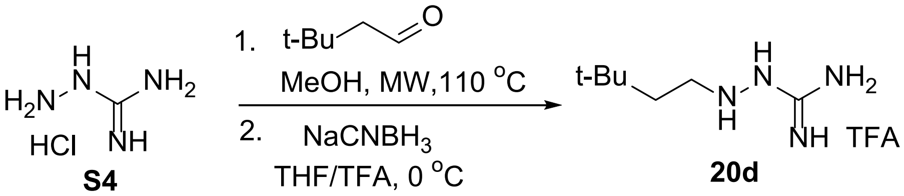

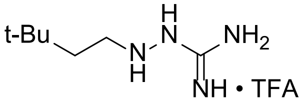

20d

Aminoguanidine hydrochloride **S4** (1.00 g, 9.05 mmol) in anhydrous MeOH (5 mL) was added the 3,3-dimethylbutanal (1.08 g,10.86 mmol) at room temperature and heated in microwave at 110 °C for 10 min. The reaction mixture cooled room temperature and methanol was removed under reduced pressure. The crude mixture was re-dissolved in THF (10 mL) and cooled down to 0 °C. After stirred at 0 °C for 5 mins, $NaCNBH_3$ (3.37 g, 54.3 mmol) was added in three portions, followed by slow addition of TFA (1.5 mL). The reaction was stirred at the same temperature for 30 mins. LC-MS indicated all starting materials were consumed with the formation of the desired carboximidamide **20d**. Reaction was quenched with water (5 mL) and extracted with DCM (15 mL x 4). Combined organic layers were washed with water, brine and dried over anhydrous Na_2SO_4. Solvent was removed under reduced pressure and the crude product was purified by Teledyne ISCO CombiFlash using RP-C18 column (H_2O and MeCN both buffered with 0.1% trifluoroacetic acid were used as the mobile phase) to obtain the desired product **20d** (1.4 g, 55% yield) as TFA salt.

^{1}H NMR (600 MHz, CD_3OD) δ 4.97 (s, 6H), 3.01–2.51 (m, 2H), 1.51–1.23 (m, 2H), 0.90 (s, 9H); ^{13}C NMR (151 MHz, CD_3OD) δ c158.89, 47.54, 40.73, 28.94, 28.49.
HR-MS: $(M+H)^+$ = 159.1655 (experimental); exact mass = 159.1604 (theoretical)
IR f (cm^{-1}): 3287, 2958, 1643, 1527, 1454, 1188, 1138.

(S)-2-(((benzyloxy)carbonyl)amino)-5-(2-(3,3-dimethylbutyl)hydrazine-1-carboximidamido) pentanoic acid TFA salt (20g):

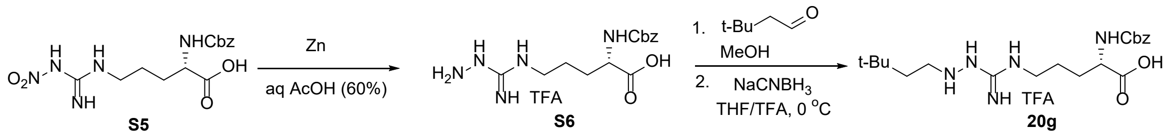

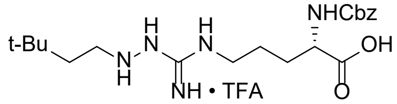

20g

Nitro-arginine **S5** (1.00 g, 2.83 mmol) in 60% aqueous AcOH (10 mL) was added Zn (1.48 g, 22.64 mmol) at room temperature in two portions. The reaction mixture stirred at room temperature for 5 minutes and passed through a pad of celite. Most of the solvent (AcOH) was removed under reduced pressure and the crude product was purified by Teledyne ISCO CombiFlash using RP-C18 column (H_2O and MeCN both buffered with 0.1% trifluoroacetic acid were used as the mobile phase) to obtain the desired product **S6** (980 mg) in 79% yield as TFA salt. The amino-arginine **S6** (980 mg, 2.24 mmol) was dissolved in anhydrous MeOH (5 mL) followed by the addition of the 3,3-dimethylbutanal (269 mg, 2.69 mmol). The reaction mixture was stirred for 4 h at room temperature (monitored by LC-MS). After complete consumption of **S6** the solvent was removed under reduced pressure and the crude product **S6** was dissolved in THF (10 mL) and cooled down to 0 °C. After stirred at 0 °C for 5 mins, $NaCNBH_3$ (844 mg, 13.44 mmol) was added in three portions, followed by slow addition of TFA (1.5 mL). The reaction was stirred at the same temperature for 30 mins. LC-MS indicated all starting materials were consumed with the formation of the desired carboximidamide **20g**. Reaction was quenched with water (5 mL) and extracted with DCM (15 mL x 4). Combined organic layers were washed with water, brine and dried over anhydrous Na_2SO_4. Solvent was removed under reduced pressure and the crude product was purified by Teledyne ISCO CombiFlash using RP-C18 column (H_2O and MeCN both buffered with 0.1% trifluoroacetic acid were used as the mobile phase) to obtain the desired product **20g** (660 mg, 56% yield) as TFA salt.

^{1}H NMR (600 MHz, DMSO-d$_6$) δ 12.63 (s, 1H), 8.63 (s, 1H), 8.12 (s, 1H), 7.57 (d, J = 8.2 Hz, 1H), 7.37 – 7.23 (m, 5H), 6.52 (s, 3H), 5.08 (s, 1H), 5.00 (s, 2H), 3.93 (td, J = 8.5, 4.8 Hz, 1H), 3.10 (t, J = 7.9 Hz, 2H), 2.64 (t, J = 8.3 Hz, 2H), 1.70 (dt, J = 17.5, 6.3 Hz, 1H), 1.55 – 1.47 (m, 3H), 1.34 – 1.22 (m, 2H), 0.84 (s, 9H). ^{13}C NMR (151 MHz, DMSO-d$_6$) δ 174.14, 166.72, 156.59, 137.38, 128.80, 128.28, 128.19, 65.88, 53.93, 47.64, 40.98, 40.47, 29.81, 29.72, 28.26, 25.85.
HR-MS: $(M+H)^+$ = 408.2612 (experimental); exact mass = 408.2605 (theoretical)
IR f (cm^{-1}): 3305, 2956, 1641, 1525, 1454, 1186, 1138.

General Procedure for the Synthesis of 2,5-Diaminoimidazoles

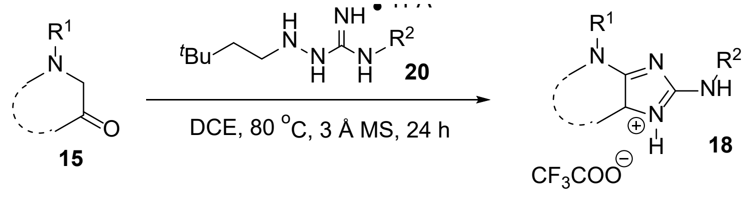

Alpha-aminoketone **15** (3 equivalence), carboximidamide **20** (1 equivalence) and 3 Å molecular sieves (10 mg) were dissolved in anhydrous DCE in an oven-dried 10 mL Schlenk tube equipped with a magnetic stirring bar under nitrogen atmosphere. The tube was capped and heated up to 80 °C in oil bath for 24h. After 24h, the tube was cooled down to room temperature and the solvent was removed under reduced pressure. The crude 4(*H*)-imidazole product **18** was purified by preparatory HPLC with a SunFire Prep C18 OBD 5μm 10x150 mm reversed-phase column as the stationary phase. H_2O and MeCN both buffered with 0.1% trifluoroacetic acid were used as the mobile phase. The characterization data for **18a** and **18b** can be found in Ref 1.

4-benzyl-*N*-(4-methoxyphenethyl)-5,6,7,7a-tetrahydro-4(*H*)-imidazo[4,5-b]pyridin-2-amine TFA salt (18c):

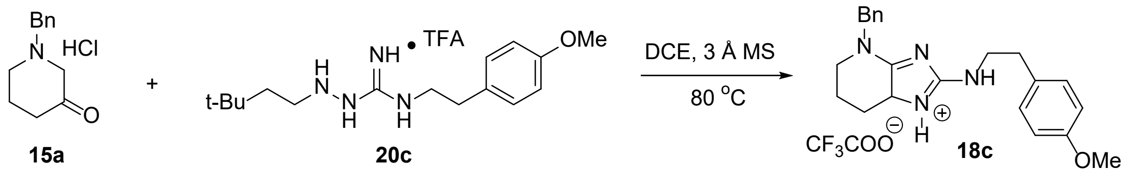

1-benzylpiperidin-3-one hydrochloride **15a** (81 mg, 0.36 mmol), carboximidamide **20c** (50 mg, 0.12 mmol), 3 Å molecular sieves (10 mg) were dissolved in anhydrous DCE (1.5 mL) in an oven-dried 10 mL Schlenk tube equipped with a magnetic stirring bar under nitrogen atmosphere. The tube was capped and heated up to 80 °C in oil bath for 24h. After 24h, the tube was cooled down to room temperature and the solvent was removed under reduced pressure. The crude 4(*H*)-imidazole product **18c** was purified by preparatory HPLC with a SunFire Prep C18 OBD 5μm 10x150 mm reversed-phase column as the stationary phase. H_2O and MeCN both buffered with 0.1% trifluoroacetic acid were used as the mobile phase. HPLC conditions: UV collection 254 nm, flow rate 20 mL/min, 15% → 45% MeCN linear gradient over 21 minutes. The HPLC fractions were combined and lyophilized. The title compound **18c** isolated as white solid (45 mg, TFA salt, 79% yield).

Prep HPLC Retention Time: 17.13 min
^{1}H NMR (600 MHz, $CDCl_3$) δ 9.44 (s, 1H), 8.91 (s, 1H), 7.43 – 7.34 (m, 3H), 7.28 – 7.21 (m, 2H), 7.12 (d, *J* = 8.6 Hz, 2H), 6.82 (d, *J* = 8.5 Hz, 2H), 4.78 (d, *J* = 14.4 Hz, 1H), 4.74 (d, *J* = 14.5 Hz, 1H), 4.42 (dd, *J* = 11.6, 6.9 Hz, 1H), 3.77 (s, 3H), 3.74 – 3.69 (m, 2H), 3.33 (td, *J* = 6.9, 3.4 Hz, 2H), 2.87 (t, *J* = 7.5 Hz, 2H), 2.60 – 2.45 (m, 1H), 1.96 – 1.66 (m, 2H), 1.44 (ddd, *J* = 20.7, 12.2, 8.8 Hz, 1H). ^{13}C NMR (151 MHz, $CDCl_3$) δ 179.72, 169.03, 158.28, 134.06, 130.11, 129.83, 129.18, 128.77, 128.28, 113.92, 58.91, 55.22, 53.64, 45.76, 44.87, 35.22, 24.08, 18.09.
HR-MS: $(M+H)^+$ = 363.2174 (experimental); exact mass = 363.2179 (theoretical)
IR *f* (cm^{-1}):2958, 1669, 1626, 1514, 1440, 1248, 1200, 1141.

N^5,N^5-diethyl-N^2-(4-methoxyphenethyl)-4-methyl-4(*H*)-imidazole-2,5-diamine TFA salt (18d):

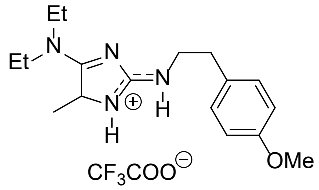

18d

Compound **18d** was synthesized using the same reaction procedure of **18c** using (diethylamino)acetone **15b** (62 mg, 0.48 mmol), carboximidamide **20c** (50 mg, 0.12 mmol) and 3 Å molecular sieves (10 mg). The crude 4(*H*)-imidazole product **18d** was purified by preparatory HPLC with a SunFire Prep C18 OBD 5µm 10x150 mm reversed-phase column as the stationary phase. H_2O and MeCN both buffered with 0.1% trifluoroacetic acid were used as the mobile phase. HPLC conditions: UV collection 254 nm, flow rate 20 mL/min, 15% → 35% MeCN linear gradient over 20 minutes. The HPLC fractions were combined and lyophilized. The title compound **18d** isolated as white solid (38 mg, TFA salt, 76% yield).

Prep HPLC Retention Time: 16.51 min
^{1}H NMR (600 MHz, $CDCl_3$) δ 9.71 (s, 1H), 8.96 (s, 1H), 7.11 (d, *J* = 8.5 Hz, 2H), 6.81 (d, *J* = 8.5 Hz, 2H), 4.62 (q, *J* = 7.3 Hz, 1H), 3.76 (s, 3H), 3.67 – 3.55 (m, 4H), 3.41 (dq, *J* = 14.3, 7.1 Hz, 1H), 3.33 (dq, *J* = 14.3, 7.1 Hz, 1H), 2.84 – 2.81 (m, 2H), 1.45 (d, *J* = 5.8 Hz, 3H), 1.31 (t, *J* = 7.1 Hz, 3H), 1.23 (t, *J* = 7.1 Hz, 3H). ^{13}C NMR (151 MHz, $CDCl_3$) δ 180.45, 167.47, 158.22, 130.30, 129.82, 113.86, 57.17, 55.20, 44.66, 44.61, 43.58, 35.36, 18.41, 13.82, 11.85.
HR-MS: $(M+H)^+$ = 303.2167 (experimental); exact mass = 303.2179 (theoretical)
IR *f* (cm^{-1}):2926, 1679, 1613, 1514, 1423, 1354, 1248, 1201,1137.

4-ethyl-*N*-(4-methoxyphenethyl)-5,6,7,7a-tetrahydro-4(*H*)-imidazo[4,5-b]pyridin-2-amine TFA salt (18e):

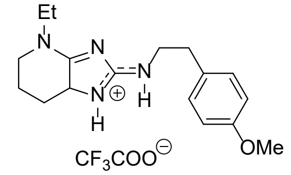

18e

Compound **18e** was synthesized using the same reaction procedure of **18c** using 1-ethylpiperidin-3-one hydrochloride **15e** (30 mg, 0.22 mmol), carboximidamide **20c** (30 mg, 0.07 mmol) and 3 Å molecular sieves (10 mg). The crude 4(*H*)-imidazole product **18e** was purified by preparatory HPLC with a SunFire Prep C18 OBD 5µm 10x150 mm reversed-phase column as the stationary phase. H_2O and MeCN both buffered with 0.1% trifluoroacetic acid were used as the mobile phase. HPLC conditions: UV collection 254 nm, flow rate 20 mL/min, 15% → 25% MeCN linear gradient over 24 minutes. The HPLC fractions were combined and lyophilized. The title compound **18e** isolated as white solid (21 mg, TFA salt, 73% yield).

Prep HPLC Retention Time: 18.69 min
^{1}H NMR (600 MHz, $CDCl_3$) δ 10.18 (s, 1H), 9.49 (s, 1H), 7.12 (d, *J* = 8.2 Hz, 2H), 6.81 (d, *J* = 8.4 Hz, 2H), 4.30 (dd, *J* = 11.4, 6.6 Hz, 1H), 3.76 (s, 3H), 3.71 (dq, *J* = 14.5, 7.2 Hz, 1H), 3.67 – 3.63 (m, 2H), 3.53 (dq, *J* = 14.2, 7.2 Hz, 1H), 3.40 (td, *J* = 6.6, 3.1 Hz, 2H), 2.95 – 2.73 (m, 2H), 2.64 – 2.35 (m, 1H), 1.97 – 1.81 (m, 2H), 1.50 – 1.35 (m, 1H), 1.24 (t, *J* = 7.2 Hz, 3H). ^{13}C NMR (151 MHz, $CDCl_3$) δ 178.83, 169.22, 158.21, 130.35, 129.84, 113.84, 58.53, 55.22, 45.86, 45.18, 44.70, 35.24, 24.06, 18.26, 12.04.
HR-MS: $(M+H)^+$ = 301.2026 (experimental); exact mass = 301.2023 (theoretical)
IR *f* (cm^{-1}):2962, 2910, 1693,1671, 1624, 1513, 1201, 1179, 1131.

N-(4-methoxyphenethyl)-5,6,7,7a-tetrahydro-4(H)-imidazo[4,5-b]pyridin-2-amine TFA salt (18f):

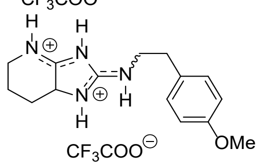

18f

Compound **18f** was synthesized using the same reaction procedure of **18c** using piperidin-3-one hydrochloride **15f** (123 mg, 0.91 mmol), carboximidamide **20c** (37 mg, 0.09 mmol) and 3 Å molecular sieves (10 mg). The crude 4(*H*)-imidazole product **18f** was purified by preparatory HPLC with a SunFire Prep C18 OBD 5μm 10x150 mm reversed-phase column as the stationary phase. H_2O and MeCN both buffered with 0.1% trifluoroacetic acid were used as the mobile phase. HPLC conditions: UV collection 254 nm, flow rate 20 mL/min, 0% → 26% MeCN linear gradient over 40 minutes. The HPLC fractions were combined and lyophilized. The title compound **18f** isolated as white solid (23 mg, TFA salt, 66% yield).

Prep HPLC Retention Time: 30.49 min

Major isomer: ^{1}H NMR (600 MHz, DMSO-d$_6$) δ 7.86 (s, 3H), 7.21 – 7.10 (m, 2H), 6.90 – 6.79 (m, 2H), 4.41 – 4.20 (m, 1H), 3.69 (s, 3H), 3.53 – 3.41 (m, 2H), 2.82 – 2.66 (m, 4H), 1.82 – 1.73 (m, 1H), 1.66 – 1.58 (m, 2H), 1.54 (td, J = 8.1, 3.9 Hz, 1H). ^{13}C NMR of all conformational isomers (151 MHz, DMSO-d$_6$) δ 182.07, 181.25, 175.85, 174.89, 168.74, 158.43, 158.33, 158.26, 158.20, 157.17, 130.74, 130.52, 130.33, 130.27, 130.21, 130.06, 129.94, 114.25, 114.21, 114.16, 58.65, 58.30, 58.04, 57.82, 55.42, 55.39, 44.61, 44.41, 44.03, 43.67, 38.76, 34.81, 34.47, 33.90, 33.60, 28.01, 27.89, 24.12, 24.04, 22.84, 22.67, 17.37, 17.27.

HR-MS: $(M+H)^+$ = 273.1709 (experimental); exact mass = 273.1710 (theoretical)

IR *f* (cm^{-1}):3445, 3050, 1691, 1514, 1273, 1201, 1052, 1025, 1006.

benzyl 2-((4-methoxyphenethyl)amino)-3,5,6,7-tetrahydro-4(H)-imidazo[4,5-b]pyridine-4-carboxylate TFA salt (18g):

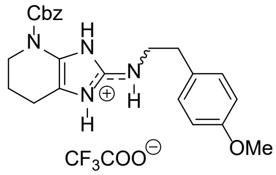

18g

Compound **18g** was synthesized using the same reaction procedure of **18c** using 1-*N*-Cbz-3-piperidone **15g** (117 mg, 0.5 mmol), carboximidamide **20c** (41 mg, 0.1 mmol) and 3 Å molecular sieves (20 mg). The crude imidazole product **18g** was purified by using Teledyne Isco with a normal phase RediSep column as the stationary phase. CH_2Cl_2 and MeOH were buffered with 0.1% TFA and used as mobile phase. Column conditions: 100% CH_2Cl_2 for 2 column volumes (CVs) followed by 0 → 15% MeOH over 20 CVs. The fractions were combined and evaporated under reduced pressure. The title compound **18g** was isolated as yellow oil (36mg, TFA salt, 69% yield).

All conformationalisomers: ^{1}H NMR (600 MHz, $CDCl_3$) δ 8.91 (s, 1H), 7.54–7.20 (m, 5H), 7.15–6.93 (m, 2H), 6.83–6.59 (m, 2H), 5.43–4.85 (m, 2H), 4.26–3.50 (m, 5H), 3.43–3.11 (m, 2H), 2.92–2.67 (m, 2H), 2.38 (m, 2H), 2.09–1.59 (m, 2H). ^{13}C NMR (151 MHz, $CDCl_3$) δ 163.05, 162.82, 158.35, 152.57, 144.49, 129.68, 128.67, 128.45, 128.16, 117.69, 115.75, 114.11, 113.92, 107.24, 68.24, 55.15, 45.06, 43.83, 34.79, 29.04, 21.45, 18.60.

HR-MS: $(M+H)^+$ = 407.2166 (experimental); exact mass = 407.2078 (theoretical)

IR f (cm^{-1}): 2958, 1693, 1612, 1513, 1248, 1199.

***tert*-butyl 2-((4-methoxyphenethyl)amino)-3,5,6,7-tetrahydro-4(*H*)-imidazo[4,5-b]pyridine-4-carboxylate TFA salt (18h):**

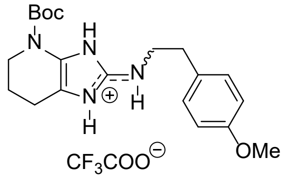
18h

Compound **18h** was synthesized using the same reaction procedure of **18c** using 1-*N*-Boc-3-piperidone **15h** (100 mg, 0.5 mmol), carboximidamide **20c** (41 mg, 0.1 mmol) and 3 Å molecular sieves (20 mg). The crude imidazole product **18h** was purified by using Teledyne Isco with a normal phase RediSep column as the stationary phase. CH_2Cl_2 and MeOH were buffered with 0.1% TFA and used as mobile phase. Column conditions: 100% CH_2Cl_2 for 2 column volumes (CVs) followed by 0 → 15% MeOH over 20 CVs. The fractions were combined and evaporated under reduced pressure. The title compound **18h** was isolated as yellow oil (31 mg, TFA salt, 64% yield).
Major Isomer: ^{1}H NMR (500 MHz, $CDCl_3$) δ 8.77 (s, 1H), 7.09 (d, *J* = 8.1 Hz, 2H), 6.76 (d, *J* = 7.0 Hz, 2H), 5.26 (s, 1H), 3.70 (s, 3H), 3.56 (t, *J* = 5.4 Hz, 2H), 3.38 (t, *J* = 7.6 Hz, 2H), 2.81 (t, *J* = 7.4 Hz, 2H), 2.45 (t, *J* = 6.2 Hz, 2H), 1.88 (t, *J* = 5.9 Hz, 2H), 1.46 (s, 9H);
Minor isomer: ^{1}H NMR (500 MHz, $CDCl_3$) δ 3.28 – 3.18 (m, 2H), 2.29 – 2.21 (m, 2H), 1.83 – 1.78 (m, 2H).
^{13}C NMR (126 MHz, $CDCl_3$) of both the isomers δ 163.00, 162.72, 158.31, 130.05, 129.72, 121.72, 117.86, 115.53, 114.05, 77.33, 77.08, 76.82, 55.13, 53.42, 45.00, 43.90, 34.80, 30.05, 29.62, 29.24, 28.35, 28.30, 28.26, 28.08, 27.86, 27.80, 18.74.
HR-MS: $(M-1)^+$ = 371.2135 (experimental); exact mass = 371.2089 (theoretical)
IR f (cm^{-1}): 2962, 1693, 1612, 1513, 1369, 1249, 1150.

4-benzyl-*N*-(4-methoxyphenethyl)-7-methyl-5,6,7,7a-tetrahydro-4(*H*)-imidazo[4,5-b]pyridin-2-amine TFA salt (18i):

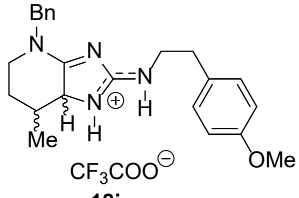
18i (dr = 2:1)

Compound **18i** was synthesized using the same reaction procedure of **18c** using 1-benzyl-4-methylpiperidin-3-one hydrochloride **15i** (53 mg, 0.22 mmol), carboximidamide **20c** (30 mg, 0.07 mmol) and 3 Å molecular sieves (10 mg). The crude 4(*H*)-imidazole product **18i** was purified by preparatory HPLC with a SunFire Prep C18 OBD 5μm 10x150 mm reversed-phase column as the stationary phase. H_2O and MeCN both buffered with 0.1% trifluoroacetic acid were used as the mobile phase. HPLC conditions: UV collection 254 nm, flow rate 5 mL/min, 0% → 50% MeCN linear gradient over 40 minutes. The HPLC fractions were combined and lyophilized. The title compound **18i** isolated as white solid (21 mg, TFA salt, 73% yield, dr = 2:1).

Prep HPLC Retention Time: 34.49 min
Major diastereomer: ^{1}H NMR (600 MHz, $CDCl_3$) δ 10.15 (s, 1H), 9.29 (bs, 1H), 7.57–7.28 (m, 3H), 7.25 (m, 2H), 7.12 (m, 2H), 6.81 (m, 2H), 5.03–4.56 (m, 2H), 4.02 (d, *J* = 10.9 Hz, 1H), 3.76 (s, 3H), 3.74–3.64 (m, 2H), 3.45–3.23 (m, 2H), 2.87 (m, 2H), 1.92 (h, *J* = 5.8 Hz, 1H), 1.68 (qt, *J* = 11.3, 5.6 Hz, 1H), 1.57–1.44 (m, 1H), 1.19 (d, *J* = 6.4 Hz, 3H).
Minor diastereomer: δ 4.48 (d, *J* = 6.1 Hz, 1H), 2.68 (m, 2H), 2.18–2.01 (m, 2H), 0.85 (s, 3H).
Both the diastereomers: ^{13}C NMR (151 MHz, $CDCl_3$) δ 179.10, 178.42, 169.33, 169.31, 158.26, 158.24, 134.12, 134.08, 130.18, 129.89, 129.83, 129.15, 129.11, 128.79, 128.72, 128.55, 128.33,

113.90, 113.88, 77.20, 76.99, 76.78, 65.17, 62.66, 55.21, 55.19, 53.56, 53.54, 46.02, 44.86, 44.81, 44.06, 35.29, 35.27, 32.26, 27.63, 27.32, 26.34, 18.92, 12.93.
HR-MS: $(M+H)^+$ = 377.2327 (experimental); exact mass = 377.2366 (theoretical)
IR f (cm^{-1}): 2961, 1688, 1623, 1513, 1426, 1248, 1199.

4-benzyl-*N*-(4-methoxyphenethyl)-4,5,6,7,8,8a-hexahydroimidazo[4,5-b]azepin-2-amine TFA salt (18j):

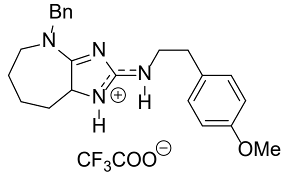

18j

Compound **18j** was synthesized using the same reaction procedure of **18c** using 1-benzyl-azepan-3-one **15j** (71 mg, 0.35 mmol), carboximidamide **20c** (30 mg, 0.07 mmol) and 3 Å molecular sieves (10 mg). The crude 4(*H*)-imidazole product **18j** was purified by preparatory HPLC with a SunFire Prep C18 OBD 5μm 10x150 mm reversed-phase column as the stationary phase. H_2O and MeCN both buffered with 0.1% trifluoroacetic acid were used as the mobile phase. HPLC conditions: UV collection 254 nm, flow rate 20 mL/min, 10% → 55% MeCN linear gradient over 18 minutes. The HPLC fractions were combined and lyophilized. The title compound **18j** isolated as white solid (28 mg, TFA salt, 82% yield).

Prep HPLC Retention Time: 14.78 min
^{1}H NMR (600 MHz, $CDCl_3$) δ 10.80 (s, 1H), 9.74 (s, 1H), 7.42 – 7.30 (m, 3H), 7.24 (d, *J* = 6.7 Hz, 2H), 7.17 – 6.99 (m, 2H), 6.87 – 6.64 (m, 2H), 4.87 – 4.66 (m, 2H), 4.66 – 4.48 (m, 1H), 3.74 (s, 3H), 3.64 (dt, *J* = 8.8, 6.2 Hz, 2H), 3.37 (qd, *J* = 14.9, 8.0 Hz, 2H), 2.85 (t, *J* = 7.6 Hz, 2H), 2.19 (d, *J* = 13.1 Hz, 1H), 2.03 – 1.92 (m, 1H), 1.78 (dd, *J* = 15.1, 4.6 Hz, 1H), 1.61 (q, *J* = 13.4 Hz, 1H), 1.41 (dt, *J* = 13.7, 10.8 Hz, 1H), 1.32 – 1.23 (m, 1H). ^{13}C NMR (151 MHz, $CDCl_3$) δ 183.02, 168.02, 158.19, 134.66, 130.39, 129.86, 129.06, 128.57, 128.34, 113.86, 63.38, 55.42, 55.20, 51.70, 44.68, 35.48, 30.61, 28.09, 27.50.
HR-MS: $(M+H)^+$ = 377.2343 (experimental); exact mass = 377.2336 (theoretical)
IR *f* (cm^{-1}): 2945, 1677, 1614, 1513, 1427, 1248, 1177, 1143.

N-(4-methoxyphenethyl)-4-methyl-5-(piperidin-1-yl)-4(*H*)-imidazol-2-amine TFA salt (18p):

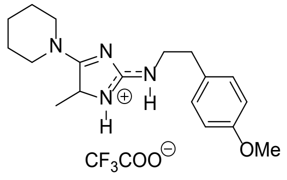

18p

Compound **18p** was synthesized using the same reaction procedure of **18c** using 1-(1-piperidinyl)acetone **15p** (49 mg, 0.35 mmol), carboximidamide **20c** (30 mg, 0.07 mmol) and 3 Å molecular sieves (10 mg). The crude 4(*H*)-imidazole product **18p** was purified by preparatory HPLC with a SunFire Prep C18 OBD 5μm 10x150 mm reversed-phase column as the stationary phase. H_2O and MeCN both buffered with 0.1% trifluoroacetic acid were used as the mobile phase. HPLC conditions: UV collection 254 nm, flow rate 5 mL/min, 15% → 35% MeCN linear gradient over 24 minutes. The HPLC fractions were combined and lyophilized. The title compound **18p** isolated as white solid (23 mg, TFA salt, 82% yield).

Prep HPLC Retention Time: 19.40 min
^{1}H NMR (600 MHz, $CDCl_3$) δ 9.62 (s, 1H), 8.90 (s, 1H), 7.10 (d, *J* = 8.6 Hz, 2H), 6.81 (d, *J* = 8.6 Hz, 2H), 4.61 (q, *J* = 6.5 Hz, 1H), 3.85 – 3.67 (m, 5H), 3.62 (q, J = 6.9 Hz, 2H), 3.49 – 3.36 (m, 2H), 2.85 – 2.78 (m, 2H), 1.79 – 1.62 (m, 6H), 1.44 (d, J = 6.4 Hz, 3H). ^{13}C NMR (151 MHz,

$CDCl_3$) δ 179.86, 167.62, 158.21, 130.30, 129.85, 113.83, 56.78, 55.20, 49.26, 47.66, 44.61, 35.31, 26.28, 25.16, 23.52, 18.38.
HR-MS: $(M+H)^+$ = 315.2195 (experimental); exact mass = 315.2179 (theoretical)
IR *f* (cm^{-1}): 2943, 1686, 1613, 1539, 1514, 1423, 1248, 1201, 1180, 1132.

N^5-benzyl-N^5-ethyl-N^2-(4-methoxyphenethyl)-4-methyl-4(*H*)-imidazole-2,5-diamine TFA salt (18q):

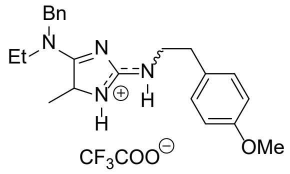

18q

Compound **18q** was synthesized using the same reaction procedure of **18c** using 1-(benzyl(ethyl)amino)propan-2-one **15q** (48 mg, 0.25 mmol), carboximidamide **20c** (20 mg, 0.05 mmol) and 3 Å molecular sieves (10 mg). The crude 4(*H*)-imidazole product **18q** was purified by preparatory HPLC with a SunFire Prep C18 OBD 5μm 10x150 mm reversed-phase column as the stationary phase. H_2O and MeCN both buffered with 0.1% trifluoroacetic acid were used as the mobile phase. HPLC conditions: UV collection 254 nm, flow rate 5 mL/min, 0% → 50% MeCN linear gradient over 70 minutes. The HPLC fractions were combined and lyophilized. The title compound **18q** isolated as white solid (19 mg, TFA salt, 78% yield, *E/Z* = 1 : 0.85).

Prep HPLC Retention Time: 56.20 min
Mixture of isomers: ^1H NMR (600 MHz, $CDCl_3$) δ 9.86 (s, 1H), 9.12 (bs, 1H), 7.61–7.29 (m, 4H), 7.24–7.01 (m, 3H), 6.82–6.78 (m, 2H), 5.04–4.26 (m, 3H), 3.76–3.75 (m, 3H), 3.72–3.56 (m, 2H), 3.45–3.26 (m, 2H), 2.88–2.81 (m, 2H), 1.50–1.40 (m, 3H), 1.29–1.15 (m, 3H). ^{13}C NMR (151 MHz, $CDCl_3$) δ 181.67, 181.19, 167.55, 167.40, 158.25, 158.22, 134.52, 133.58, 130.30, 130.21, 129.97, 129.84, 129.83, 129.34, 129.06, 128.70, 128.48, 128.04, 126.67, 114.31, 113.87, 113.82, 57.57, 57.32, 55.21, 55.20, 52.82, 50.98, 44.74, 44.71, 44.34, 43.79, 35.44, 35.39, 18.49, 18.45, 13.34, 11.34.
HR-MS: $(M+H)^+$ = 365.2339 (experimental); exact mass = 365.2336 (theoretical)
IR *f* (cm^{-1}): 2946, 1680, 1611, 1514, 1422, 1355, 1201, 1139.

N^5-ethyl-N^2-(4-methoxyphenethyl)-4-methyl-N^5-phenyl-4(*H*)-imidazole-2,5-diamine TFA salt (18r):

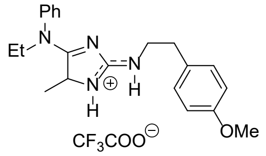

18r

Compound **18r** was synthesized using the same reaction procedure of **18c** using 1-(ethyl(phenyl)amino)propan-2-one **15r** (44 mg, 0.25 mmol), carboximidamide **20c** (20 mg, 0.05 mmol) and 3 Å molecular sieves (10 mg). The crude 4(*H*)-imidazole product **18r** was purified by preparatory HPLC with a SunFire Prep C18 OBD 5μm 10x150 mm reversed-phase column as the stationary phase. H_2O and MeCN both buffered with 0.1% trifluoroacetic acid were used as the mobile phase. HPLC conditions: UV collection 254 nm, flow rate 5 mL/min, 0% → 45% MeCN linear gradient over 40 minutes. The HPLC fractions were combined and lyophilized. The title compound **18r** isolated as white solid (14 mg, TFA salt, 61% yield).

Prep HPLC Retention Time: 35.01 min
^1H NMR (500 MHz, $CDCl_3$) δ 9.01 (s, 1H), 8.51 (s, 1H), 7.54–7.47 (m, 3H), 7.21 (d, *J* = 7.4 Hz, 2H), 7.14 (d, *J* = 8.1 Hz, 2H), 6.85 (d, *J* = 8.1 Hz, 2H), 4.57 (q, *J* = 6.8 Hz, 1H), 4.25 (dt, *J* = 13.6,

6.9 Hz, 1H), 3.79 (s, 3H), 3.76–3.71 (m, 3H), 2.87 (t, *J* = 7.4 Hz, 2H), 1.23 (t, *J* = 7.2 Hz, 3H), 0.90 (d, *J* = 6.7 Hz, 3H). ^{13}C NMR (126 MHz, $CDCl_3$) δ 181.05, 167.29, 158.33, 139.68, 130.57, 130.11, 129.89, 129.77, 126.63, 113.94, 58.31, 55.22, 49.27, 44.83, 35.36, 16.41, 11.92.
HR-MS: $(M+H)^+$ = 351.2176 (experimental); exact mass = 351.2179 (theoretical)
IR *f* (cm^{-1}): 2962, 1682, 1605, 1592, 1514, 1456, 1424, 1201, 1145.

N^5,N^5-dibenzyl-4-ethyl-N^2-(4-methoxyphenethyl)-4(*H*)-imidazole-2,5-diamine TFA salt (18s):

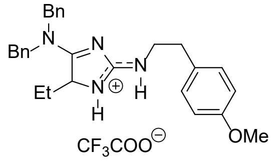

18s

Compound **18s** was synthesized using the same reaction procedure of **18c** using 1-(dibenzylamino)butan-2-one **15s** (40 mg, 0.15 mmol), carboximidamide **20c** (12 mg, 0.03 mmol) and 3 Å molecular sieves (10 mg). The crude 4(*H*)-imidazole product **18s** was purified by preparatory HPLC with a SunFire Prep C18 OBD 5μm 10x150 mm reversed-phase column as the stationary phase. H_2O and MeCN both buffered with 0.1% trifluoroacetic acid were used as the mobile phase. HPLC conditions: UV collection 254 nm, flow rate 5 mL/min, 0% → 55% MeCN linear gradient over 50 minutes. The HPLC fractions were combined and lyophilized. The title compound **18s** isolated as white solid (9 mg, TFA salt, 56% yield).

Prep HPLC Retention Time: 45.58 min
^{1}H NMR (600 MHz, $CDCl_3$) δ 10.30 (s, 1H), 9.53 (s, 1H), 7.48 – 7.38 (m, 3H), 7.39 – 7.34 (m, 3H), 7.17 (dd, *J* = 6.6, 2.9 Hz, 2H), 7.15 – 7.09 (m, 4H), 6.82 (d, *J* = 8.5 Hz, 2H), 4.83 (d, *J* = 14.4 Hz, 1H), 4.70 (dd, *J* = 6.3, 3.5 Hz, 1H), 4.63 (d, *J* = 14.4 Hz, 1H), 4.54 (d, *J* = 16.0 Hz, 1H), 4.38 (d, *J* = 16.0 Hz, 1H), 3.87 – 3.67 (m, 5H), 2.93 – 2.86 (m, 2H), 2.03 – 1.93 (m, 1H), 1.74 – 1.65 (m, 1H), 0.88 (t, *J* = 7.3 Hz, 3H). ^{13}C NMR (151 MHz, $CDCl_3$) δ 181.17, 168.11, 158.43, 134.53, 133.27, 130.41, 130.14, 129.66, 129.24, 129.07, 128.84, 128.82, 127.12, 114.05, 62.95, 55.38, 52.29, 51.66, 44.96, 35.64, 25.41, 8.70.
HR-MS: $(M+H)^+$ = 441.2665 (experimental); exact mass = 441.2649 (theoretical)
IR *f* (cm^{-1}): 2970, 2957, 2939, 1683, 1606, 1528, 1514, 1456, 1437, 1248, 1202, 1142.

4-benzyl-5,6,7,7a-tetrahydro-4(*H*)-imidazo[4,5-b]pyridin-2-amine TFA salt (18t):

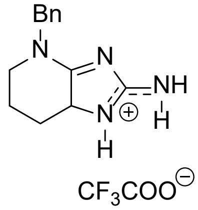

18t

Compound **18t** was synthesized using the same reaction procedure of **18c** using 1-benzylpiperidin-3-one hydrochloride **15a** (47 mg, 0.21 mmol), carboximidamide **20d** (19 mg, 0.07 mmol) and 3 Å molecular sieves (10 mg). The crude 4(*H*)-imidazole product **18t** was purified by preparatory HPLC with a SunFire Prep C18 OBD 5μm 10x150 mm reversed-phase column as the stationary phase. H_2O and MeCN both buffered with 0.1% trifluoroacetic acid were used as the mobile phase. HPLC conditions: UV collection 254 nm, flow rate 20 mL/min, 0% → 30% MeCN linear gradient over 20 minutes. The HPLC fractions were combined and lyophilized. The title compound **18t** isolated as white solid (17 mg, TFA salt, 73% yield, *E/Z* conformational isomers = 7 : 1).

Prep HPLC Retention Time: 14.66 min

^{1}H NMR (500 MHz, $CDCl_3$) δ 10.84 (s, 1H), 9.68 (s, 1H), 7.50–7.00 (m, 5H), 6.18 (s, 1H), 4.75 (s, 2H), 4.48 (dd, *J* = 11.7, 6.9 Hz, 1H), 3.47–3.19 (m, 2H), 2.54 (q, *J* = 11.6, 8.2 Hz, 1H), 1.91–1.69 (m, 2H), 1.51 (ddd, *J* = 20.8, 12.2, 8.7 Hz, 1H); 13C NMR (126 MHz, $CDCl_3$) δ 180.19, 170.09, 133.87, 129.19, 128.77, 128.22, 59.53, 53.70, 45.67, 23.86, 17.91.
HR-MS: $(M+H)^+$ = 229.1488 (experimental); exact mass = 229.1488 (theoretical)
IR *f* (cm^{-1}): 3183, 1633, 1496, 1457, 1425,1356, 1131.

***N*^{5},*N*^{5}-diethyl-4-methyl-4(*H*)-imidazole-2,5-diamine TFA salt (18u):**

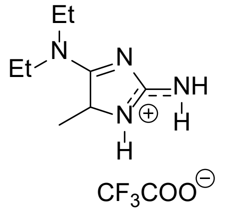

18u

Compound **18u** was synthesized using the same reaction procedure of **18c** using (diethylamino)acetone **15b** (45 mg, 0.35 mmol), carboximidamide **20d** (19 mg, 0.07 mmol) and 3 Å molecular sieves (10 mg). The crude 4(*H*)-imidazole product **18u** was purified by preparatory HPLC with a SunFire Prep C18 OBD 5μm 10x150 mm reversed-phase column as the stationary phase. H_2O and MeCN both buffered with 0.1% trifluoroacetic acid were used as the mobile phase. HPLC conditions: UV collection 254 nm, flow rate 20 mL/min, 0% → 13% MeCN linear gradient over 26 minutes. The HPLC fractions were combined and lyophilized. The title compound **18u** isolated as white solid (11 mg, TFA salt, 56% yield).
Prep HPLC Retention Time: 13.31 min
^{1}H NMR (600 MHz, $CDCl_3$) δ 11.29 (s, 1H), 9.88 (s, 1H), 5.77 (s, 1H), 4.68 (q, *J* = 6.5 Hz, 1H), 3.59 (dh, *J* = 13.8, 7.1 Hz, 2H), 3.44 (dq, *J* = 14.2, 7.0 Hz, 1H), 3.35 (dq, *J* = 14.5, 7.3 Hz, 1H), 1.52 (d, *J* = 6.6 Hz, 3H), 1.33 (t, *J* = 7.2 Hz, 3H), 1.24 (t, *J* = 7.2 Hz, 3H). ^{13}C NMR (151 MHz, $CDCl_3$) δ 180.99, 168.60, 57.97, 44.72, 43.68, 18.27, 13.91, 11.90.
HR-MS: $(M+H)^+$ = 169.1473 (experimental); exact mass = 169.1448 (theoretical)
IR *f* (cm^{-1}): 3170, 2956, 1672, 1499, 1419, 1353, 1200, 1136.

***N*-allyl-4-benzyl-5,6,7,7a-tetrahydro-4(*H*)-imidazo[4,5-b]pyridin-2-amine TFA salt (18v):**

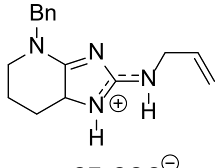

18v

Compound **18v** was synthesized using the same reaction procedure of **18c** using 1-benzylpiperidin-3-one hydrochloride **15a** (34 mg, 0.15 mmol), carboximidamide **20e** (16 mg, 0.05 mmol) and 3 Å molecular sieves (10 mg). The crude 4(*H*)-imidazole product **18v** was purified by preparatory HPLC with a SunFire Prep C18 OBD 5μm 10x150 mm reversed-phase column as the stationary phase. H_2O and MeCN both buffered with 0.1% trifluoroacetic acid were used as the mobile phase. HPLC conditions: UV collection 254 nm, flow rate 5 mL/min, 0% → 35% MeCN linear gradient over 40 minutes. The HPLC fractions were combined and lyophilized. The title compound **18v** isolated as white solid (15 mg, TFA salt, 81% yield).

Prep HPLC Retention Time: 32.50 min
^{1}H NMR (500 MHz, $CDCl_3$) δ 9.93 (s, 1H), 9.27 (s, 1H), 7.60–7.32 (m, 3H), 7.30–7.26 (m, 2H), 5.89 (ddt, *J* = 16.1, 10.6, 5.4 Hz, 1H), 5.28 (d, *J* = 17.1 Hz, 1H), 5.20 (d, *J* = 10.3 Hz, 1H), 4.79 (s, 2H), 4.46 (dd, *J* = 11.6, 6.8 Hz, 1H), 4.14 (t, *J* = 5.5 Hz, 2H), 3.44–3.31 (m, 2H), 2.69–2.47 (m, 1H), 2.01–1.61 (m, 2H), 1.61–1.44 (m, 1H); ^{13}C NMR (126 MHz, $CDCl_3$) δ 179.85, 169.12, 134.06, 132.91, 129.16, 128.76, 128.32, 117.26, 59.05, 53.70, 45.76, 45.46, 24.09, 18.11.
HR-MS: $(M+H)^+$ = 269.1781 (experimental); exact mass = 269.1761 (theoretical)

IR *f* (cm^{-1}): 3224, 1675, 1626, 1440, 1199, 1138.

N^2-allyl-N^5,N^5-diethyl-4-methyl-4(*H*)-imidazole-2,5-diamine TFA salt (18w):

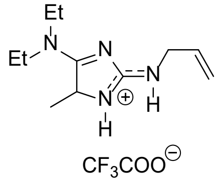

18w

Compound **18w** was synthesized using the same reaction procedure of **18c** using (diethylamino)acetone **15b** (32 mg, 0.25 mmol), carboximidamide **20e** (16 mg, 0.05 mmol) and 3 Å molecular sieves (10 mg). The crude 4(*H*)-imidazole product **18w** was purified by preparatory HPLC with a SunFire Prep C18 OBD 5μm 10x150 mm reversed-phase column as the stationary phase. H_2O and MeCN both buffered with 0.1% trifluoroacetic acid were used as the mobile phase. HPLC conditions: UV collection 254 nm, flow rate 5 mL/min, 0% → 26% MeCN linear gradient over 30 minutes. The HPLC fractions were combined and lyophilized. The title compound **18w** isolated as white solid (12 mg, TFA salt, 78% yield).

Prep HPLC Retention Time: 23.70 min
^{1}H NMR (600 MHz, $CDCl_3$) δ 10.67 (s, 1H), 9.60 (s, 1H), 6.01–5.75 (m, 1H), 5.32–5.03 (m, 2H), 4.63 (q, *J* = 6.7 Hz, 1H), 4.12–3.98 (m, 2H), 3.82–3.53 (m, 2H), 3.44–3.35 (m, 2H), 1.50 (d, *J* = 6.7 Hz, 3H), 1.32 (t, *J* = 7.2 Hz, 3H), 1.24 (t, *J* = 7.1 Hz, 3H); ^{13}C NMR (151 MHz, $CDCl_3$) δ 180.55, 167.67, 133.28, 116.92, 77.19, 76.98, 76.77, 57.30, 45.19, 44.60, 43.62, 18.48, 13.91, 11.84.
HR-MS: $(M+H)^+$ = 209.1758 (experimental); exact mass = 209.1761 (theoretical)
IR *f* (cm^{-1}): 3223, 2926, 1689, 1613, 1536, 1417, 1352, 1201, 1135.

4-benzyl-2-(piperidin-1-yl)-5,6,7,7a-tetrahydro-4(*H*)-imidazo[4,5-b]pyridine TFA salt (18x):

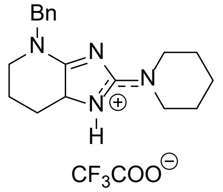

18x

Compound **18x** was synthesized using the same reaction procedure of **18c** using 1-benzylpiperidin-3-one hydrochloride **15a** (34 mg, 0.15 mmol), carboximidamide **20f** (17 mg, 0.05 mmol) and 3 Å molecular sieves (10 mg). The crude 4(*H*)-imidazole product **18x** was purified by preparatory HPLC with a SunFire Prep C18 OBD 5μm 10x150 mm reversed-phase column as the stationary phase. H_2O and MeCN both buffered with 0.1% trifluoroacetic acid were used as the mobile phase. HPLC conditions: UV collection 254 nm, flow rate 5 mL/min, 0% → 45% MeCN linear gradient over 50 minutes. The HPLC fractions were combined and lyophilized. The title compound **18x** isolated as white solid (13 mg, TFA salt, 63% yield).

Prep HPLC Retention Time: 37.86 min
^{1}H NMR (500 MHz, $CDCl_3$) δ 8.94 (s, 1H), 7.44–7.35 (m, 3H), 7.30–7.13 (m, 2H), 4.83–4.72 (m, 2H), 4.70–4.48 (m, 1H), 3.83 (t, *J* = 5.1 Hz, 2H), 3.61–3.48 (m, 2H), 3.33 (td, *J* = 6.7, 2.9 Hz, 2H), 2.63–2.42 (m, 1H), 1.99–1.58 (m, 8H), 1.62–1.42 (m, 1H); ^{13}C NMR (126 MHz, $CDCl_3$) δ 179.32, 166.89, 134.01, 129.14, 128.68, 128.15, 59.57, 53.48, 47.85, 47.12, 45.70, 25.39, 25.01, 23.99, 23.49, 18.00.
HR-MS: $(M+H)^+$ = 297.2076 (experimental); exact mass = 297.2074 (theoretical)
IR *f* (cm^{-1}): 2926, 1673, 1613, 1456, 1281, 1201.

N,N-diethyl-4-methyl-2-(piperidin-1-yl)-4(H)-imidazol-5-amine TFA salt (18y):

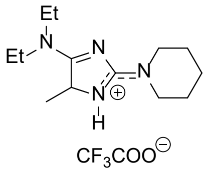

18y

Compound **18y** was synthesized using the same reaction procedure of **18c** using (diethylamino)acetone **15b** (32 mg, 0.25 mmol), carboximidamide **20f** (17 mg, 0.05 mmol) and 3 Å molecular sieves (10 mg). The crude 4(H)-imidazole product **18y** was purified by preparatory HPLC with a SunFire Prep C18 OBD 5μm 10x150 mm reversed-phase column as the stationary phase. H_2O and MeCN both buffered with 0.1% trifluoroacetic acid were used as the mobile phase. HPLC conditions: UV collection 254 nm, flow rate 5 mL/min, 0% → 50% MeCN linear gradient over 40 minutes. The HPLC fractions were combined and lyophilized. The title compound **18y** isolated as white solid (10 mg, TFA salt, 59% yield).

Prep HPLC Retention Time: 33.50 min
Major isomer: ^{1}H NMR (600 MHz, CD_3OD) δ 4.93 (q, J = 6.7 Hz, 1H), 3.88–3.74 (m, 2H), 3.64 (qd, J = 6.9, 3.4 Hz, 2H), 3.49–3.45 (m, 3H), 3.24–3.16 (m, 1H), 1.75–1.62 (m, 6H), 1.47 (d, J = 6.7 Hz, 3H), 1.29 (t, J = 7.4 Hz, 3H), 1.23 (t, J = 7.1 Hz, 3H); ^{13}C NMR (151 MHz, CD_3OD) δ 180.07, 161.51, 57.72, 49.06, 46.18, 44.50, 43.21, 25.15, 24.97, 23.20, 17.37, 12.64, 10.70.
HR-MS: $(M+H)^+$ = 237.2083 (experimental); exact mass = 237.2074 (theoretical)
IR f (cm^{-1}): 2926, 1673, 1613, 1456, 1281, 1201.

5-((4-benzyl-5,6,7,7a-tetrahydro-4(H)-imidazo[4,5-b]pyridin-2-yl)amino)-2-(((benzyloxy)carbonyl)amino)pentanoic acid TFA salt (18z):

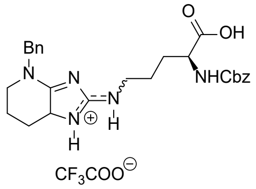

18z

Compound **18z** was synthesized using the same reaction procedure of **18c** using 1-benzylpiperidin-3-one hydrochloride **15a** (34 mg, 0.15 mmol), carboximidamide **20g** (25 mg, 0.05 mmol) and 3 Å molecular sieves (10 mg). The crude 4(H)-imidazole product **18z** was purified by preparatory HPLC with a SunFire Prep C18 OBD 5μm 10x150 mm reversed-phase column as the stationary phase. H_2O and MeCN both buffered with 0.1% trifluoroacetic acid were used as the mobile phase. HPLC conditions: UV collection 254 nm, flow rate 5 mL/min, 0% → 45% MeCN linear gradient over 40 minutes. The HPLC fractions were combined and lyophilized. The title compound **18z** isolated as white solid (16 mg, TFA salt, 53% yield, E/Z = 3.5 : 1, dr = 1:1).

Prep HPLC Retention Time: 33.99 min
Major isomer: ^{1}H NMR (600 MHz, CD_3CN) δ 9.29 (s, 1H), 8.94 (bs, 1H), 7.76–7.00 (m, 10H), 6.14 (d, J = 8.2 Hz, 1H), 5.23–5.00 (m, 2H), 4.90–4.60 (m, 2H), 4.50 (dd, J = 11.5, 7.4 Hz, 1H), 4.27–4.12 (m, 1H), 3.59–3.24 (m, 4H), 2.46–2.35 (m, 1H), 1.93–1.89 (m, 1H), 1.85–1.65 (m, 5H), 1.52–1.41 (m, 1H). ^{13}C NMR (151 MHz, CD_3CN) δ 180.21, 169.45, 169.42, 156.38, 137.10, 135.05, 134.97, 128.84, 128.47, 128.44, 128.31, 128.17, 128.03, 127.89, 127.66, 66.13, 58.68, 53.59, 53.10, 45.90, 41.93, 28.15, 25.58, 23.65, 17.47.
HR-MS: $(M+H)^+$ = 478.2461 (experimental); exact mass = 478.2449 (theoretical)
IR f (cm^{-1}): 3282, 2957, 1686, 1623, 1531, 1455, 1427, 1347, 1201, 1138.

2-(((benzyloxy)carbonyl)amino)-5-((5-(diethylamino)-4-methyl-4(*H*)-imidazol-2-yl)amino)pentanoic acid TFA salt (18aa):

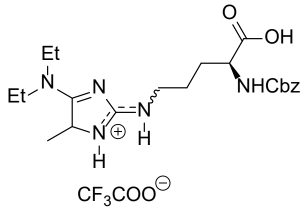
18aa

Compound **18aa** was synthesized using the same reaction procedure of **18c** using (diethylamino)acetone **15b** (27 mg, 0.21 mmol), carboximidamide **20g** (16 mg, 0.03 mmol) and 3 Å molecular sieves (10 mg). The crude 4(*H*)-imidazole product **18aa** was purified by preparatory HPLC with a SunFire Prep C18 OBD 5μm 10x150 mm reversed-phase column as the stationary phase. H_2O and MeCN both buffered with 0.1% trifluoroacetic acid were used as the mobile phase. HPLC conditions: UV collection 254 nm, flow rate 5 mL/min, 0% → 45% MeCN linear gradient over 40 minutes. The HPLC fractions were combined and lyophilized. The title compound **18aa** isolated as white solid (8 mg, TFA salt, 51% yield, *E/Z* = 2.5:1).

Prep HPLC Retention Time: 32.60 min
Mixture of isomers: ^{1}H NMR (600 MHz, CD_3OD) δ 7.47–7.12 (m, 5H), 5.08 (s, 2H), 4.94–4.88 (m, 1H), 4.26–4.15 (m, 1H), 3.68–3.56 (m, 2H), 3.54–3.42 (m, 2H), 3.23–3.14 (m, 2H), 1.99–1.86 (m, 1H), 1.78–1.64 (m, 3H), 1.52–1.40 (m, 3H), 1.32–1.26 (m, 3H), 1.26–1.18 (m, 3H); ^{13}C NMR (151 MHz, CD_3OD) δ 171.86, 157.26, 157.19, 136.68, 128.03, 127.62, 127.38, 127.34, 66.31, 57.70, 53.57, 44.45, 43.08, 40.41, 28.28, 24.91, 17.25, 12.67, 10.77.
HR-MS: $(M+H)^+$ = 418.2518 (experimental); exact mass = 418.2449 (theoretical)
IR *f* (cm^{-1}):3316, 2955, 1681, 1605, 1453, 1206, 1137.

benzyl N2-((benzyloxy)carbonyl)-N6-(bis(4-methoxyphenyl)methyl)-N6-(2-oxopropyl)-L-lysinate (19)

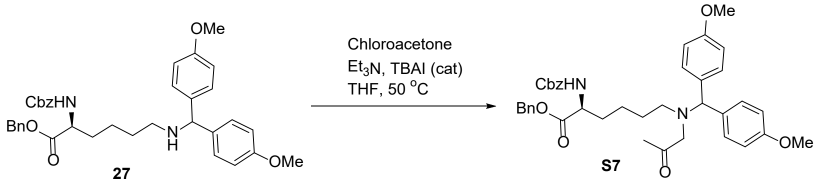

The known Dod-Lys-OBzl **27**[1] (1 g, 1.67 mmol) in THF (25 mL) were added chloroacetone (1.4 mL, 16.7 mmol), Et_3N (2.4 mL, 16.7 mmol) and catalytic amount of tetrabutylammonium iodide (TBAI) at room temperature. The reaction mixture was heated at 50 ºC for 48 h and brought to room temperature. The crude mixture was dissolved in water (20 mL), washed with EtOAc (2x20 mL) and the combined organic layers were dried over anhydrous sodium sulfate. The filtrate was concentrated and purified by silica gel column chromatography (hexanes/EtOAc) to provide dod-Lys amino ketone **S7** in 72% yield (0.78 g).

^{1}H NMR (500 MHz, $CDCl_3$) δ 7.42–7.22 (m, 14H), 6.91–6.77 (m, 4H), 5.60 (d, *J* = 8.3 Hz, 1H), 5.32–5.02 (m, 4H), 4.89 (s, 1H), 4.42 (td, *J* = 8.0, 4.9 Hz, 1H), 3.73 (s, 6H), 3.25 (s, 2H), 2.58–2.46 (m, 2H), 2.01 (s, 3H), 1.86–1.70 (m, 1H), 1.69–1.56 (m, 1H), 1.51–1.35 (m, 2H), 1.34–1.20 (m, 2H); ^{13}C NMR (126 MHz, $CDCl_3$) δ 209.37, 172.39, 158.62, 156.02, 136.39, 135.45, 134.35, 134.31, 129.39, 129.37, 128.61, 128.52, 128.42, 128.25, 128.13, 128.05, 113.81, 69.88, 67.00, 66.89, 61.02, 55.15, 53.92, 51.57, 32.39, 27.68, 26.58, 22.96.
HR-MS: $(M+H)^+$ = 653.3222 (experimental); exact mass = 653.3221 (theoretical)

IR *f* (cm^{-1}): 2951, 1713, 1609, 1508, 1455, 1346, 1243, 1174, 1033.

benzyl N2-((benzyloxy)carbonyl)-N6-(2-oxopropyl)-L-lysinate TFA salt (28)

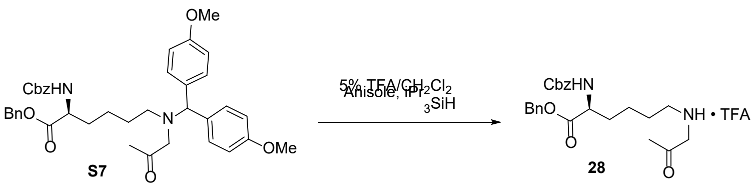

Dod-Lys-aminoketone **S7** (250 mg, 0.38 mmol) in CH_2Cl_2 (8 mL) were added triisopropylsilane (0.39 mL, 1.9 mmol), anisole (0.6 mL, 0.57 mmol) and TFA (0.4 mL) at 0 ºC. The reaction mixture stirred for additional 2 h (monitored by TLC) at 0 ºC. Volatiles were evaporated by rotary evaporator and purified by Teledyne Isco with a reverse phase 15.5 g RediSep C18 column as the stationary phase. Water and ACN with 0.1 % TFA were used as the mobile phase. Column conditions: 10% ACN for 2 column volumes (CVs), 10 to 50% ACN over 10 CVs, 50% ACN for 2 CVs, 50 to 80 % ACN over 1 CVs, 80% ACN over 1 CV and finally 100% ACN over 5 CVs. The combined fractions were lyophilized to obtain a white TFA salt of lysine-aminoketone **28** (166 mg, 82% yield).

^{1}H NMR (500 MHz, CD_3OD) δ 7.51 – 7.12 (m, 11H), 5.18 (d, *J* = 12.3 Hz, 1H), 5.13 (d, *J* = 12.3 Hz, 1H), 5.08 (d, *J* = 3.1 Hz, 2H), 4.23 (dd, *J* = 9.4, 5.0 Hz, 1H), 4.06 (s, 2H), 2.92 (ddd, *J* = 9.4, 7.0, 3.2 Hz, 2H), 2.22 (s, 3H), 1.97 – 1.78 (m, 1H), 1.78 – 1.61 (m, 3H), 1.56 – 1.18 (m, 2H). ^{13}C NMR (126 MHz, CD_3OD) δ 200.03, 172.26, 157.32, 136.71, 135.81, 128.19, 128.09, 127.96, 127.87, 127.64, 127.39, 66.54, 66.29, 54.51, 53.81, 46.87, 30.51, 25.75, 24.96, 22.39.
HR-MS: $(M+H)^+$ = 427.2231 (experimental); exact mass = 427.2227 (theoretical)
IR *f* (cm^{-1}): 2957, 1733, 1676, 1521, 1456, 1202, 1137.

(2S)-5-(((E)-5-(((S)-6-(benzyloxy)-5-(((benzyloxy)carbonyl)amino)-6-oxohexyl)imino)-4-methyl-4,5-dihydro-1H-imidazol-2-yl)amino)-2-(((benzyloxy)carbonyl)amino)pentanoic acid TFA salt (29)

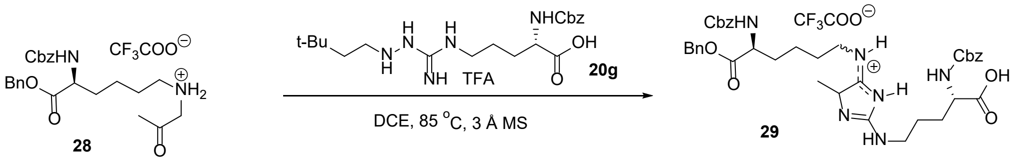

Compound **29** was synthesized using the same reaction procedure of **18c** using lys-aminoketone TFA salt **28** (27 mg, 0.05 mmol), guanylhydrazrazine **20g** (52 mg, 0.1 mmol), and 3 Å molecular sieves (10 mg). The crude 4(*H*)-imidazole product **29** was purified by preparatory HPLC with a SunFire Prep C18 OBD 5μm 10x150 mm reversed-phase column as the stationary phase. H_2O and MeCN both buffered with 0.1% trifluoroacetic acid were used as the mobile phase. HPLC conditions: UV collection 254 nm, flow rate 20 mL/min, 10% → 60% MeCN linear gradient over 30 minutes. The HPLC fractions were combined and lyophilized. The title compound **29** isolated as white solid (25 mg, 61% yield, *E/Z* = 1.2:1).

Prep HPLC Retention Time: 23.73 min

Mixture of *E/Z*-isomers: ^{1}H NMR (600 MHz, CD_3OD) δ 7.39–7.23 (m, 15H), 5.20–5.03 (m, 6H), 4.59–4.49 (m, 1H), 4.33–4.04 (m, 2H), 3.52–3.33 (m, 3H), 3.26–3.21 (m, 1H), 1.98–1.78 (m, 2H), 1.76–1.57 (m, 6H), 1.45–1.33 (m, 3H); ^{13}C NMR (151 MHz, CD_3OD) δ 181.70, 180.90, 173.99, 172.50, 172.46, 167.40, 157.28, 157.26, 136.71, 136.68, 135.82, 135.80, 128.15, 128.05, 128.03, 127.91, 127.89, 127.78, 127.60, 127.56, 127.38, 127.36, 127.34, 127.32, 66.45, 66.26, 66.24, 58.12, 58.10, 57.47, 53.99, 53.35, 53.25, 53.13, 42.57, 42.40, 41.55, 41.51, 30.57, 30.52, 28.53, 28.08, 27.50, 25.75, 24.86, 22.51, 17.25, 17.17.

HR-MS: $(M+H)^+$ = 715.3444 (experimental); exact mass = 715.3450 (theoretical)

IR *f* (cm^{-1}): 3292, 2938, 1639, 1612, 1425, 1345, 1201, 1136, 739, 721, 797.

MODIC tris-trifluoroacetate

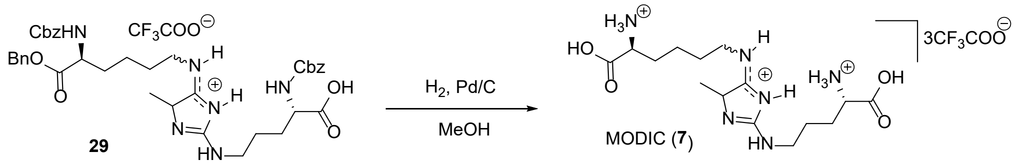

Protected MODIC **29** (10 mg, 0.012 mmol) in MeOH (2 mL) and the solution was purged with nitrogen for 5 min. 10% Pd/C (1.1 mg, 0.0012 mmol) was added to the vial and the resulting slurry was further purged with nitrogen (1 min) before H_2 gas was added via a double walled balloon. Upon completion as indicated by LCMS analysis (~ 3 h), the reaction mixture was purged with nitrogen (5 min) and TFA (1 mL) was added. The crude reaction was filtered through a pad of celite with the aid of MeOH containing 0.1% TFA (15 mL) and the filtrate was evaporated to dryness in vacuo. The crude mixture was purified by preparative scale HPLC. Preparatory HPLC was performed with a SunFire Prep C18 OBD 5 μm 10x150 mm reversed-phase column as the stationary phase. Water and MeOH with 0.1 % TFA were used as the mobile phase. HPLC conditions: UV collection 254 nm, flow rate 5 mL/min, 0% MeOH linear gradient over 5 minutes, 0% → 5% MeOH over 3 min, 5% MeOH linear gradient over 5 min, and 5% → 100% MeOH over 5 min. The run was finished with a 100% MeOH wash. The HPLC fractions were combined and lyophilized, and the title compound **7** was isolated in mixture of *E/Z*-isomers in 64% yield (5 mg, 0.007 mmol, >95% pure, *E/Z* = 2.5:1) as a white fluffy solid.

Prep HPLC Retention Time: 13.82 min

Major isomer: ^{1}H NMR (600 MHz, Deuterium Oxide) δ 4.54 (q, J = 7.2 Hz, 1H), 3.96–3.85 (m, 2H), 3.29 – 3.24 (m, 2H), 3.17 (t, J = 6.7 Hz, 2H), 1.90–1.75 (m, 4H), 1.70–1.46 (m, 4H), 1.43–1.28 (m, 2H), 1.27 (d, J = 7.0 Hz, 3H); ^{13}C NMR (151 MHz, Deuterium Oxide) δ 180.66, 171.95, 171.69, 166.34, 58.00, 52.62, 52.43, 42.40, 41.35, 29.14, 27.04, 26.83, 23.57, 21.28, 17.38.

Selected for minor isomer: ^{1}H NMR (600 MHz, Deuterium Oxide) δ 4.46 (q, J = 7.0 Hz, 1H), 3.40 – 3.31 (m, 4H), 1.24 (d, J = 6.9 Hz, 3H). ^{13}C NMR (151 MHz, Deuterium Oxide) δ 181.89, 171.82, 171.81, 167.37, 57.26, 41.60, 41.52, 26.73, 26.67, 24.54, 17.56.

HR-MS: $(M+H)^+$ = 357.2211 (experimental); exact mass = 357.2245 (theoretical)

IR *f* (cm^{-1}): 2962, 1674, 1613, 1417, 1188, 1129, 1016, 838, 797, 722.

MODIC tris-formate:

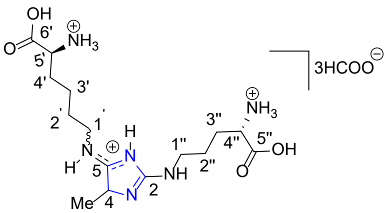

MODIC

For the formic salt of MODIC, TFA salt of isoimidazole **29** (3 mg, 0.00036 mmol) in MeOH (1 mL) and the solution was purged with nitrogen for 3 min. 10% Pd/C (0.4 mg, 0.00036 mmol) was added to the vial and the resulting slurry was further purged with nitrogen (1 min) before H_2 gas was added via a double walled balloon. Upon completion as indicated by LCMS analysis (~ 3 h), the reaction mixture was purged with nitrogen (3 min) and formic acid (3 mL) was added. The crude reaction was filtered through a pad of celite with the aid of MeOH containing 1% formic acid (15 mL), the filtrate was stirred for 1 h at room temperature and evaporated to dryness in vacuo. The crude mixture was purified by preparative scale HPLC. Preparatory HPLC was performed with a SunFire Prep C18 OBD 5 μm 10x150 mm reversed-phase column as the stationary phase. Water and MeOH with 0.1 % formic acid were used as the mobile phase. HPLC conditions: UV collection 254 nm, flow rate 5 mL/min, 0% MeOH linear gradient over 5 minutes, 0% → 5% MeOH over 3 min, 5% MeOH linear gradient over 5 min, and 5% → 100% MeOH over 5 min. The run was finished with a 100% MeOH wash. The HPLC fractions were combined and lyophilized, and the title compound was isolated in mixture of *E*/*Z*-isomers in 58% yield (1 mg, >95% pure) as a white fluffy solid.

Table S1: Comparison to previously published ^{1}H and ^{13}C NMR δ(ppm) values for MODIC formate salt in D_2O.

^{1}H NMR	Lederer Assignment δ (ppm)	Our Assignment δ (ppm)
H-4	4.72	4.72
H_3C-4	1.45	1.45
H_2-1'	3.43	3.43
H_2-2'	1.68	1.68
H_2-3'	1.44	1.44
H_2-4'	1.90	1.90
H-5'	3.72	3.72
H_2-1"	3.33	3.33
H_2-2"	1.69–1.75	1.67–1.75
H_2-3"	1.90	1.90
H-4"	3.76	3.76
$HCOO^-$	8.41	8.42

	Lederer Assignment *J* (Hz)	Our Assignment *J* (Hz)
$^{3}J_{4,Me\text{-}4}$	6.9	7.0
$^{3}J_{1',2'}$	7.0	6.9
$^{3}J_{2',3'}$	7.2	-
$^{3}J_{3',4'}$	7.2	-
$^{3}J_{4',5'}$	6.1	6.1
$^{3}J_{1'',2''}$	6.8	6.8
$^{3}J_{3'',4''}$	6.1	6.1

^{13}C NMR	Lederer Assignment δ (ppm)	Our Assignment δ (ppm)
C-2	167.0	167.24
C-4	58.6	58.81
C-5	181.3	181.55
H_3C-4	18.1	18.22
C-1'	43.0	43.28
C-2'	27.8	28.06
C-3'	21.9	22.21
C-4'	30.4	30.64
C-5'	55.0	55.29
C-6'	174.7	174.98
C-1"	42.1	42.32
C-2"	24.2	24.48
C-3"	28.0	28.25
C-4"	54.7	54.97
C-5"	175.1	175.33
$HCOO^-$	170.7	171.16

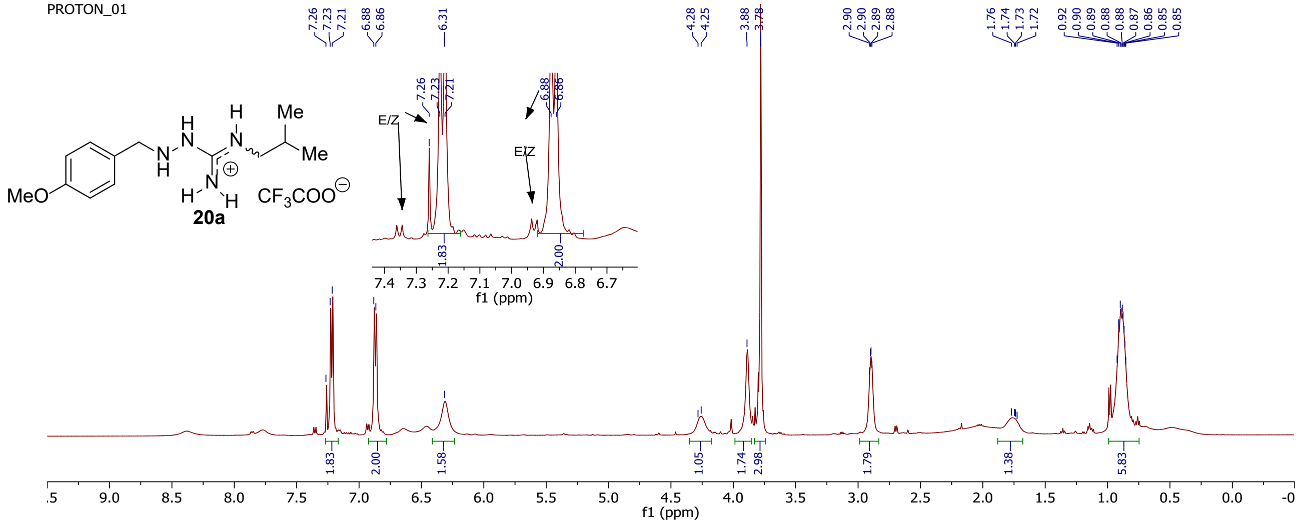
PROTON_01
7.26
7.23
7.21
6.88
6.86
6.31
4.28
4.25
3.88
3.78
2.90
2.90
2.89
2.88
1.76
1.74
1.73
1.72
0.92
0.90
0.89
0.88
0.88
0.87
0.86
0.85
0.85
E/Z
E/Z
MeO
CF3COO
20a
f1 (ppm)

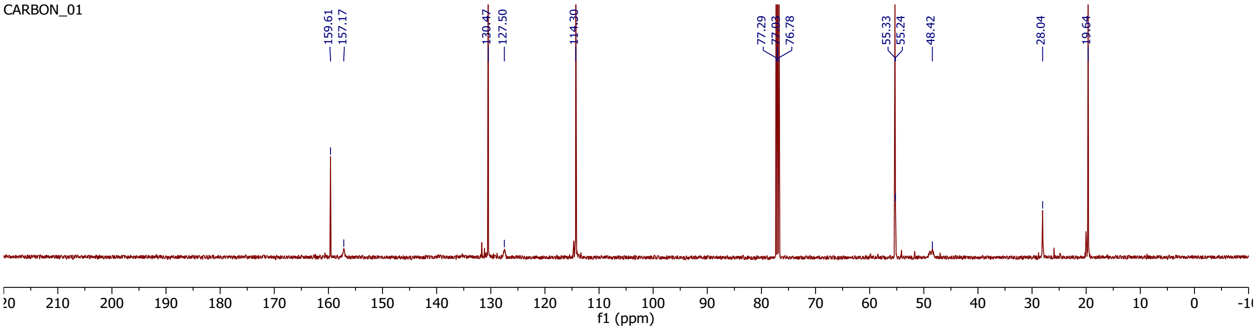
CARBON_01
159.61
157.17
130.47
127.50
114.30
77.29
77.03
76.78
55.33
55.24
48.42
28.04
19.64
f1 (ppm)

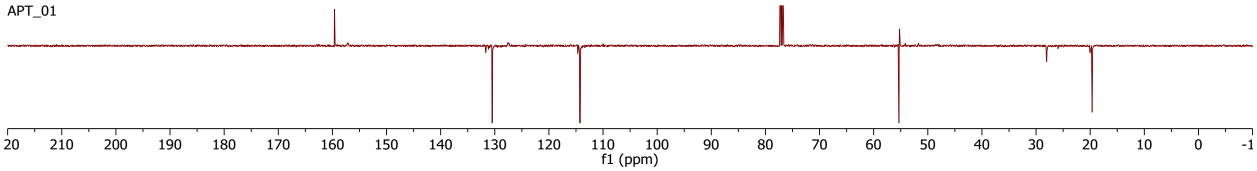
APT_01
f1 (ppm)

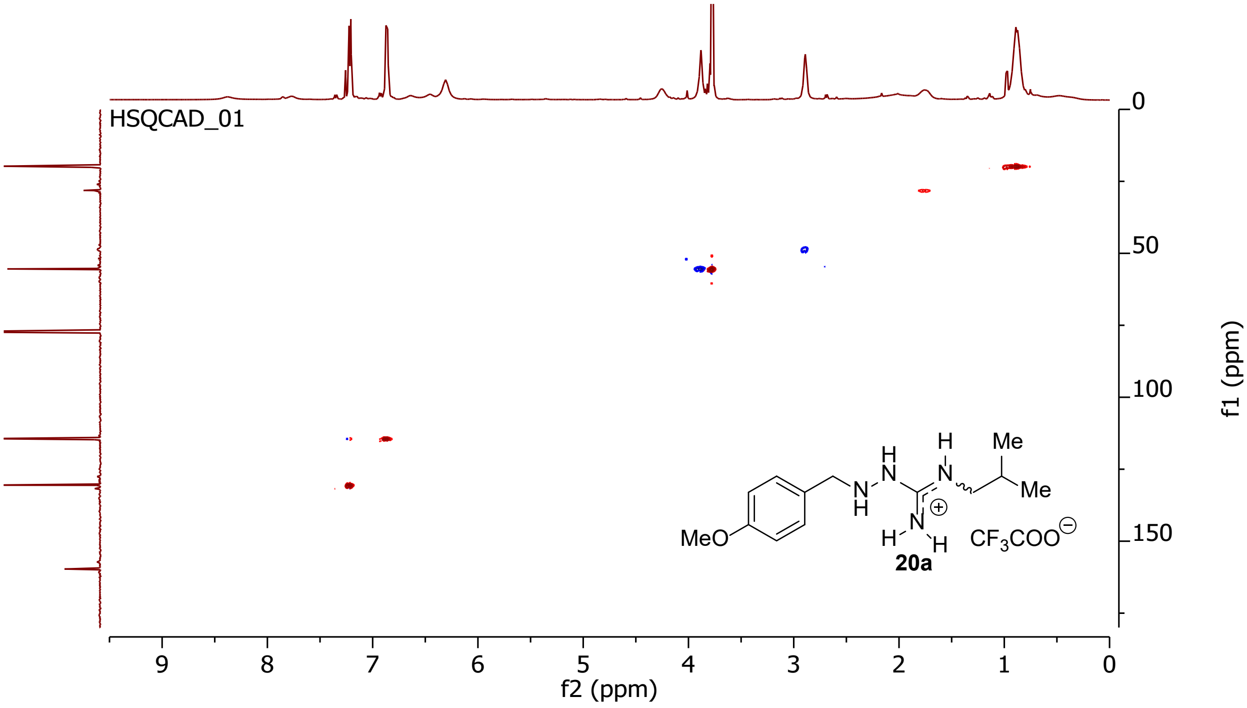
HSQCAD_01
MeO
CF3COO
20a
f2 (ppm)
f1 (ppm)

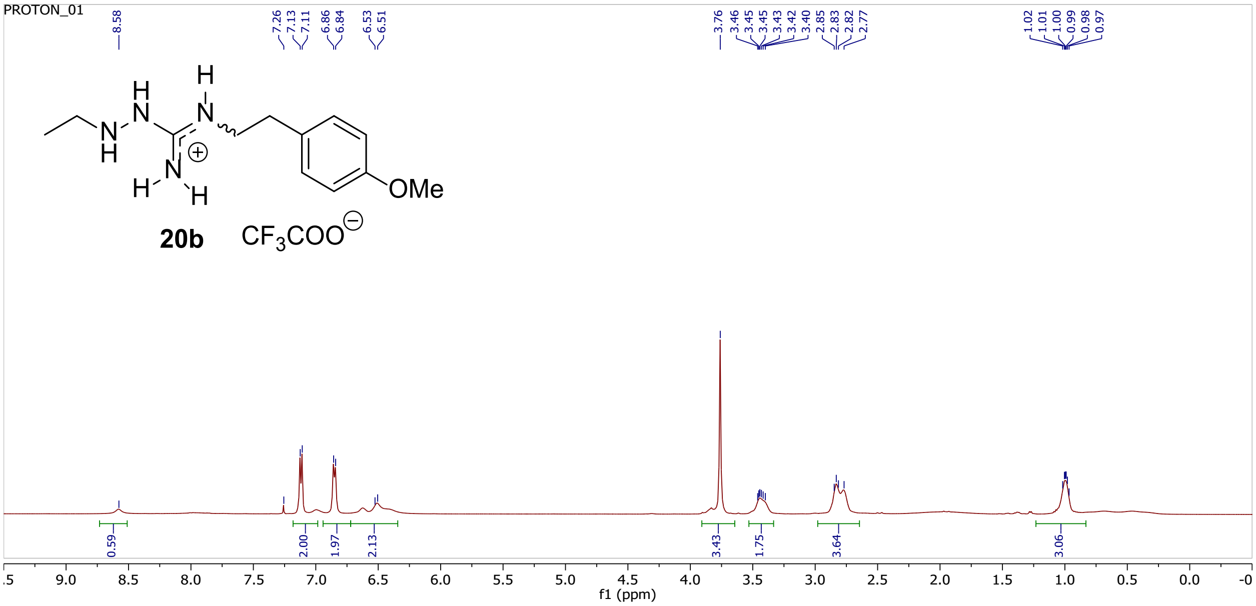
PROTON_01
8.58
7.26
7.13
7.11
6.86
6.84
6.53
6.51
3.76
3.46
3.45
3.45
3.43
3.42
3.40
2.85
2.83
2.82
2.77
1.02
1.01
1.00
0.99
0.98
0.97
OMe
20b
CF3COO
0.59
2.00
1.97
2.13
3.43
1.75
3.64
3.06
f1 (ppm)

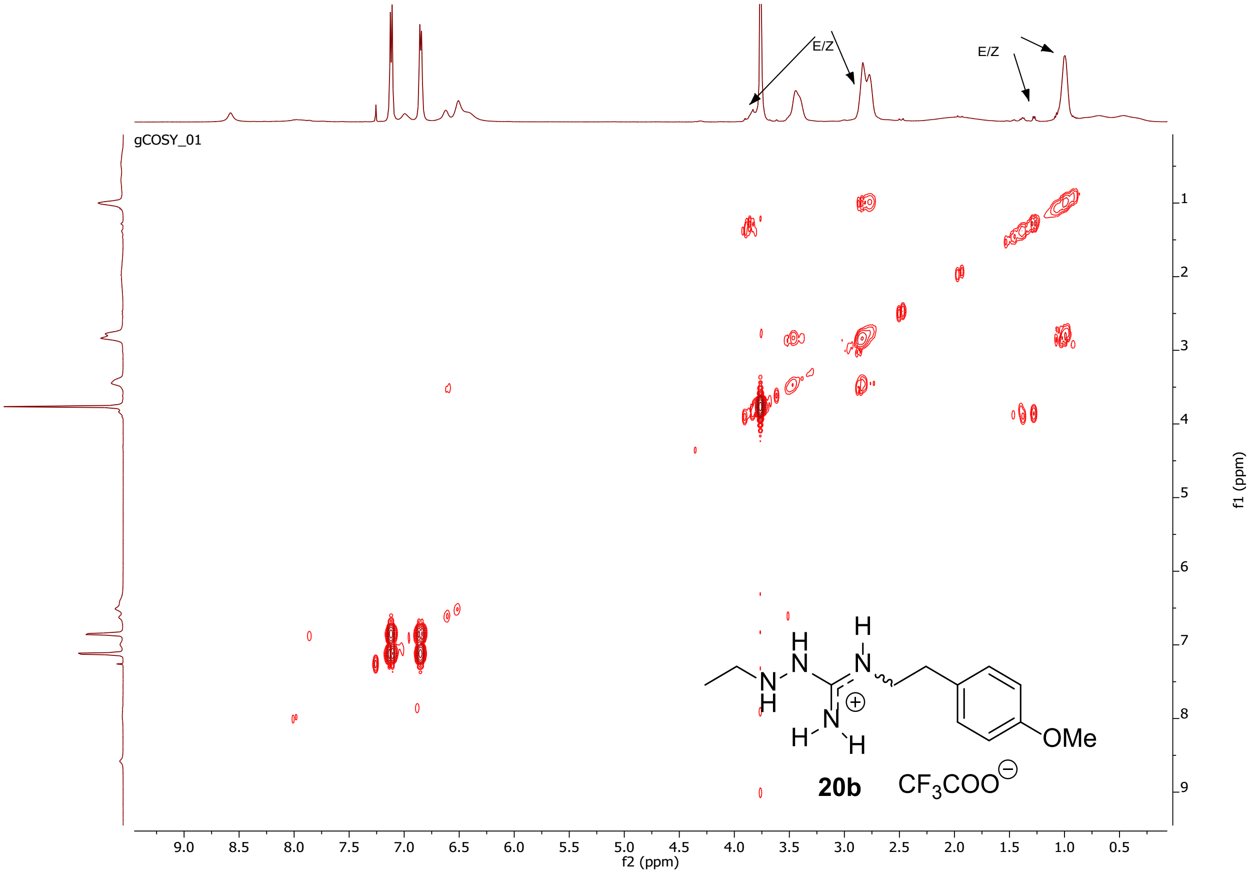
E/Z
E/Z
gCOSY_01
OMe
20b
CF3COO
f2 (ppm)
f1 (ppm)

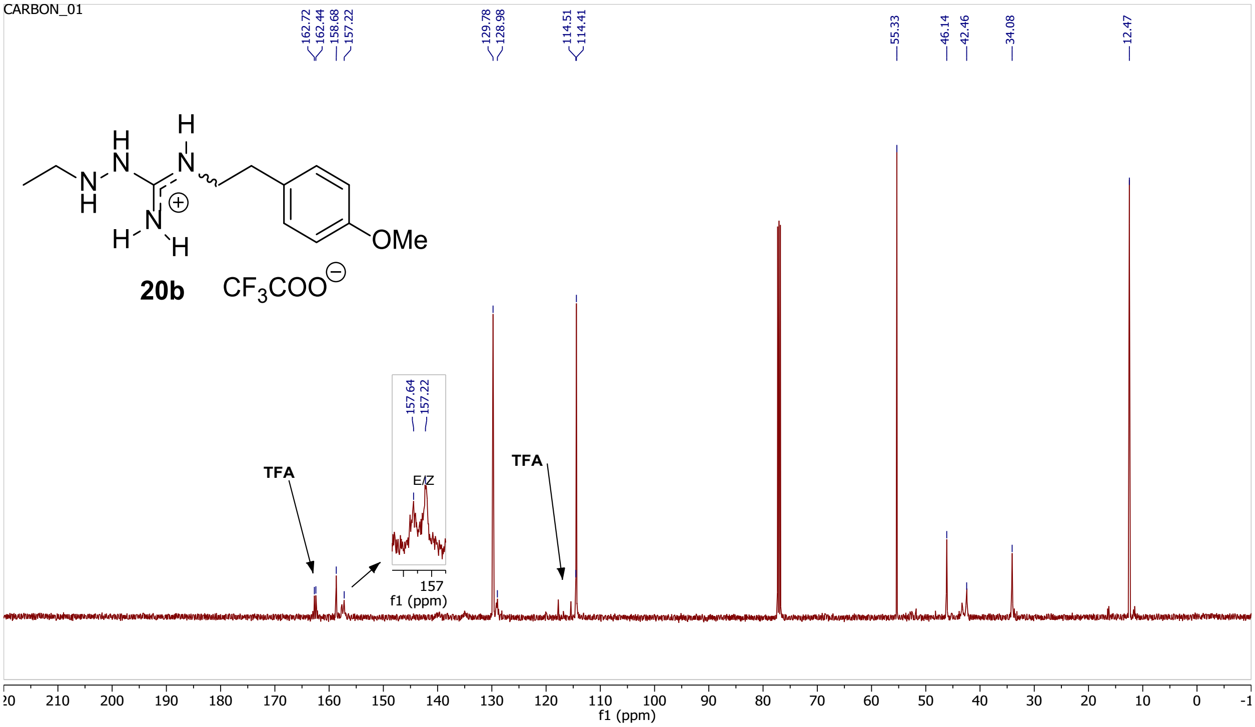
CARBON_01
162.72
162.44
158.68
157.22
129.78
128.98
114.51
114.41
55.33
46.14
42.46
34.08
12.47
20b
CF3COO
OMe
TFA
TFA
157.64
157.22
E/Z
157
f1 (ppm)
f1 (ppm)

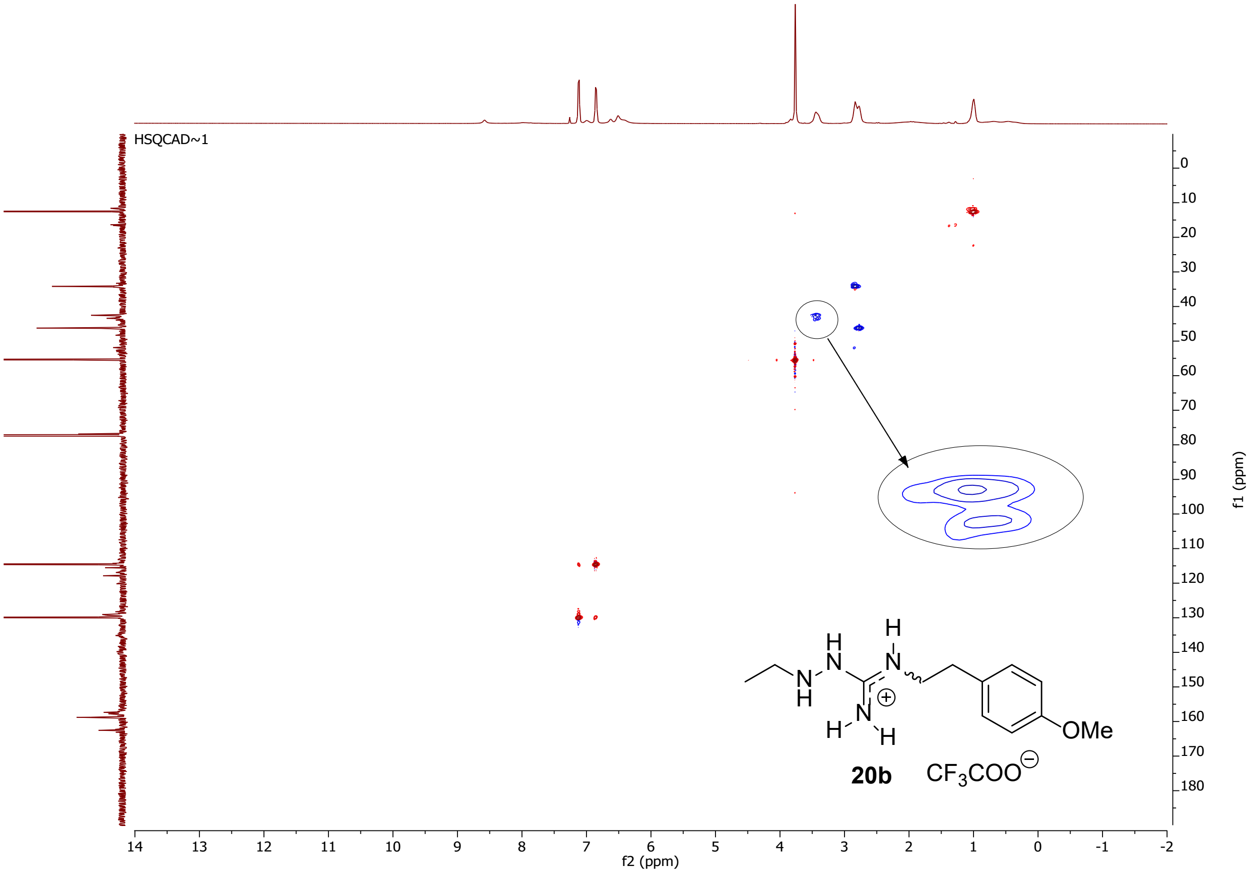
HSQCAD~1
20b
CF3COO
OMe
f1 (ppm)
f2 (ppm)

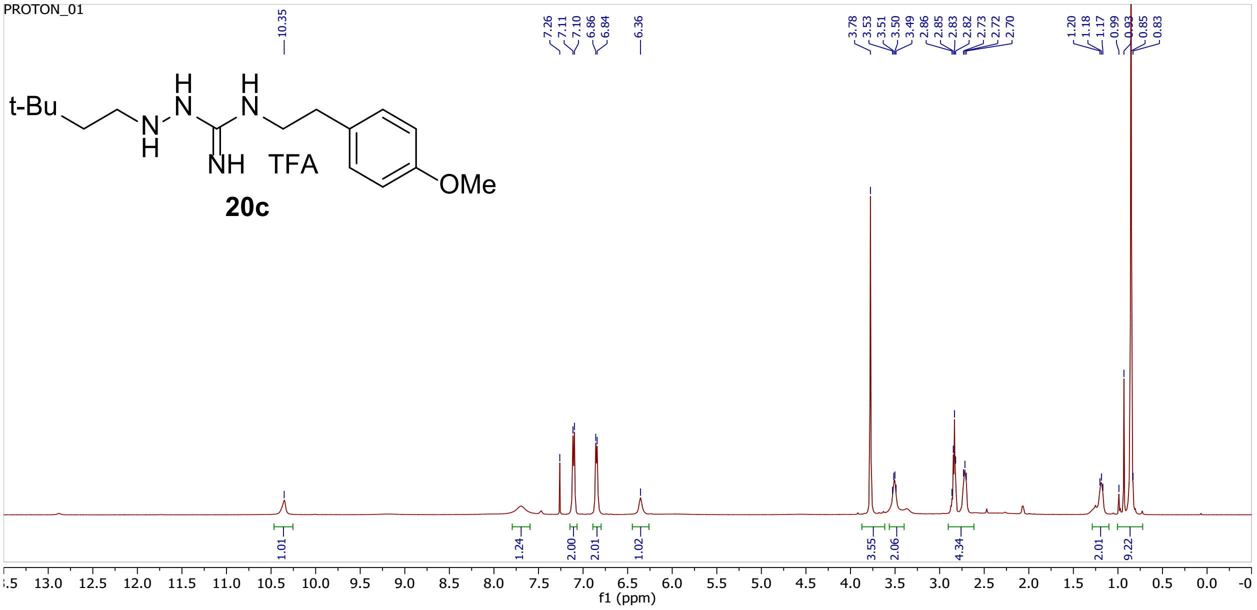
PROTON_01
10.35
7.26 7.11 7.10 6.86 6.84
6.36
3.78 3.53 3.51 3.50 3.49 2.86 2.85 2.83 2.82 2.73 2.72 2.70
1.20 1.18 1.17 0.99 0.93 0.85 0.83
t-Bu
NH TFA
OMe
20c
1.01 1.24 2.00 2.01 1.02 3.55 2.06 4.34 2.01 9.22
f1 (ppm)

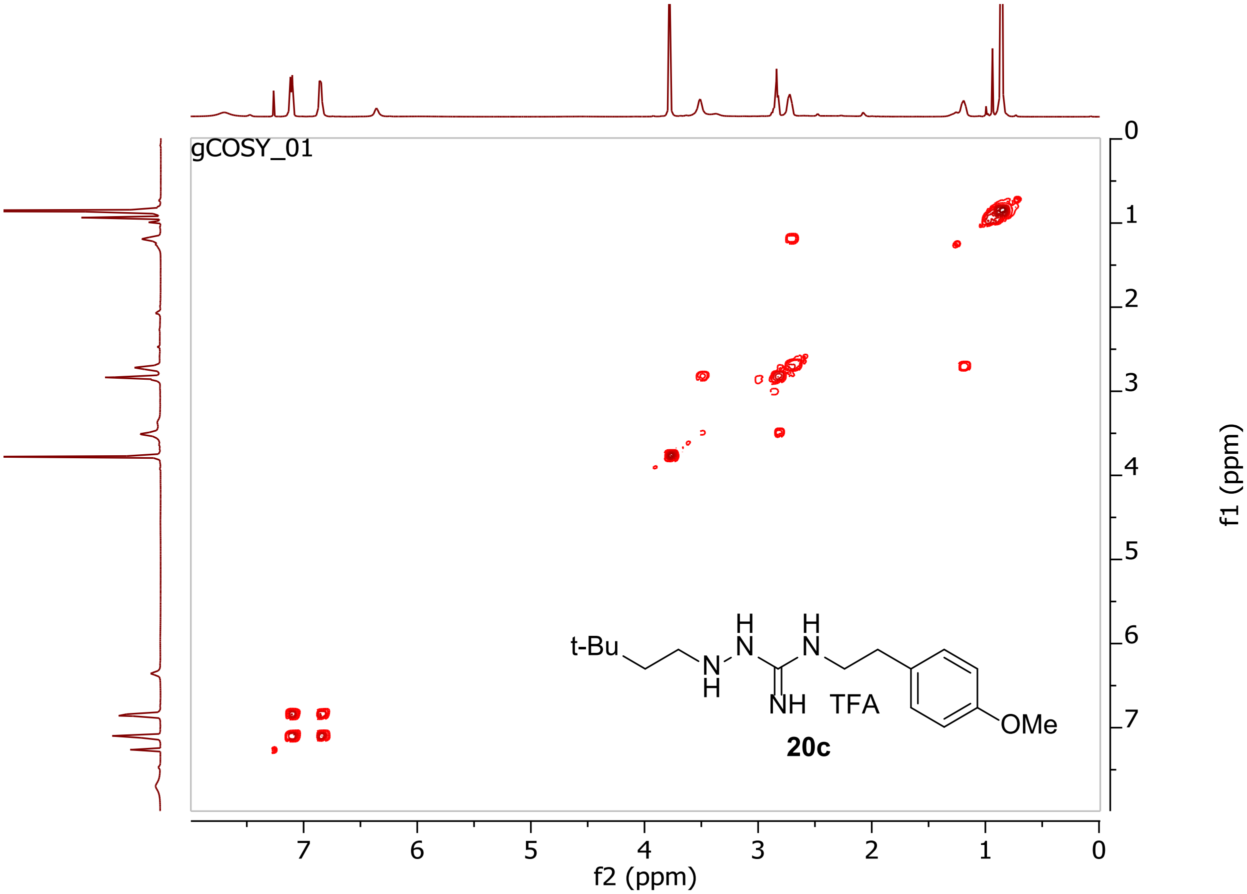
gCOSY_01
t-Bu
NH TFA
OMe
20c
f2 (ppm)
f1 (ppm)

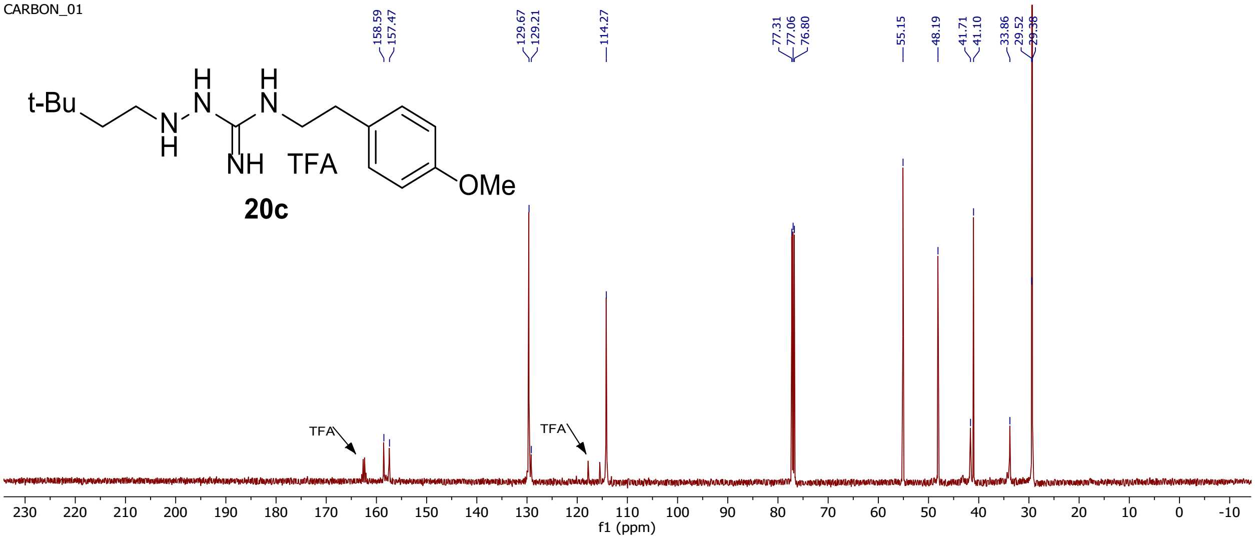
CARBON_01
158.59
157.47
129.67
129.21
114.27
77.31
77.06
76.80
55.15
48.19
41.71
41.10
33.86
29.52
29.38
t-Bu
H
N
N
H
H
N
NH TFA
OMe
20c
TFA
TFA
f1 (ppm)

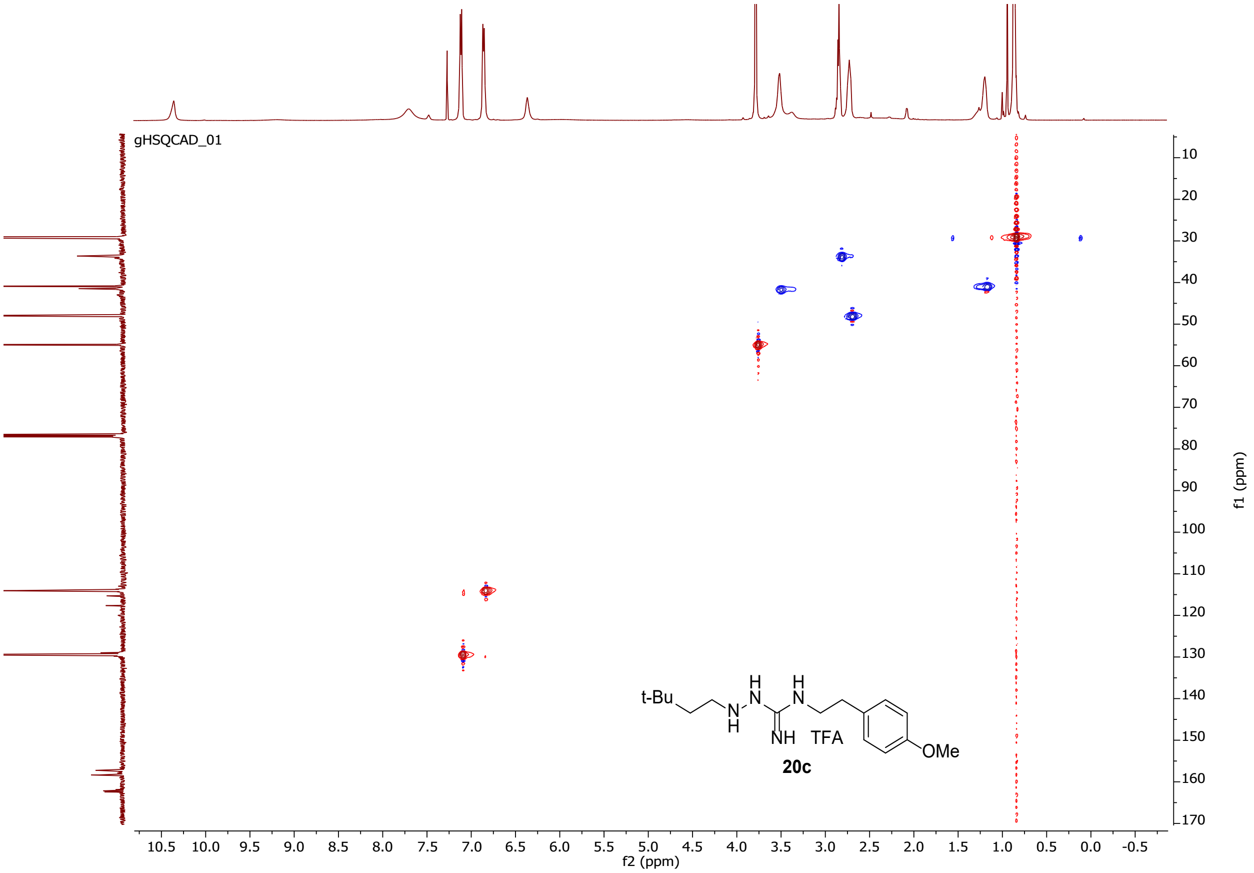
gHSQCAD_01
t-Bu
H
N
N
H
H
N
NH TFA
OMe
20c
f2 (ppm)
f1 (ppm)

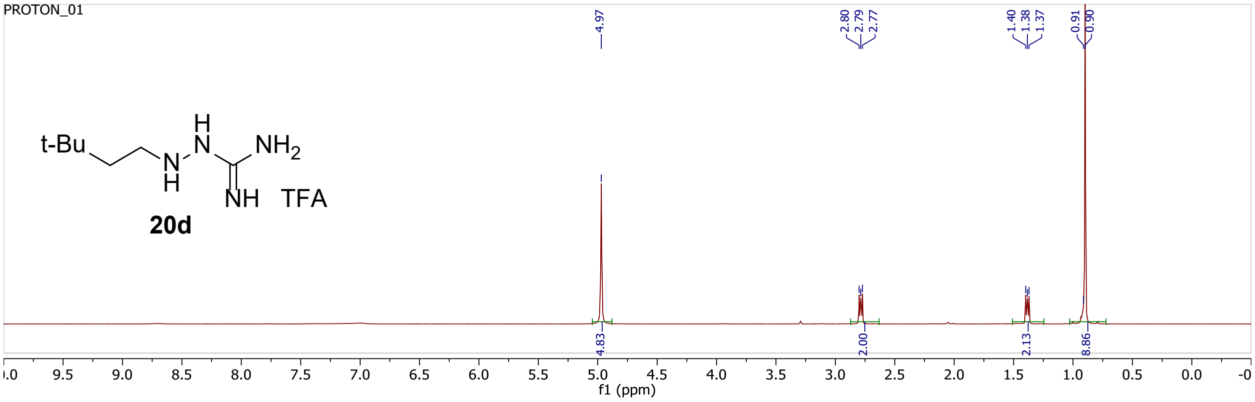
PROTON_01
t-Bu
H
N
NH2
N
H
NH
TFA
20d
4.97
2.80
2.79
2.77
1.40
1.38
1.37
0.91
0.90
4.83
2.00
2.13
8.86
f1 (ppm)

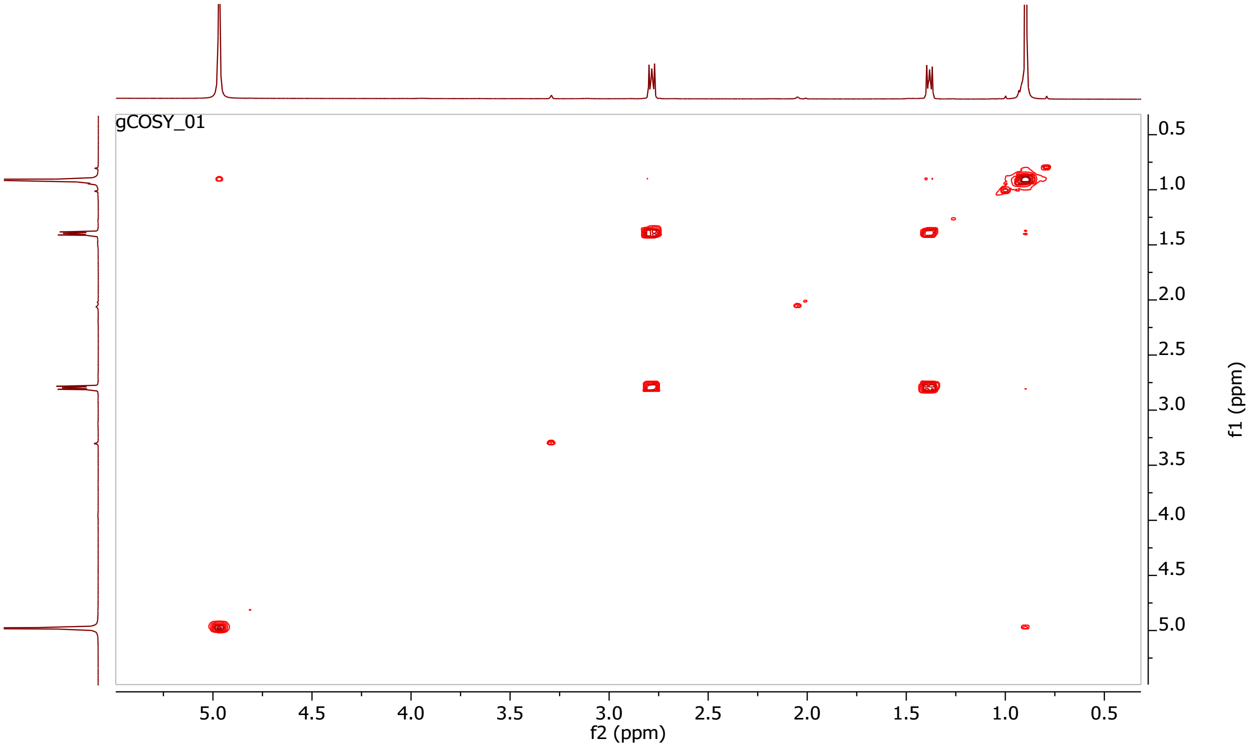
gCOSY_01
f2 (ppm)
f1 (ppm)

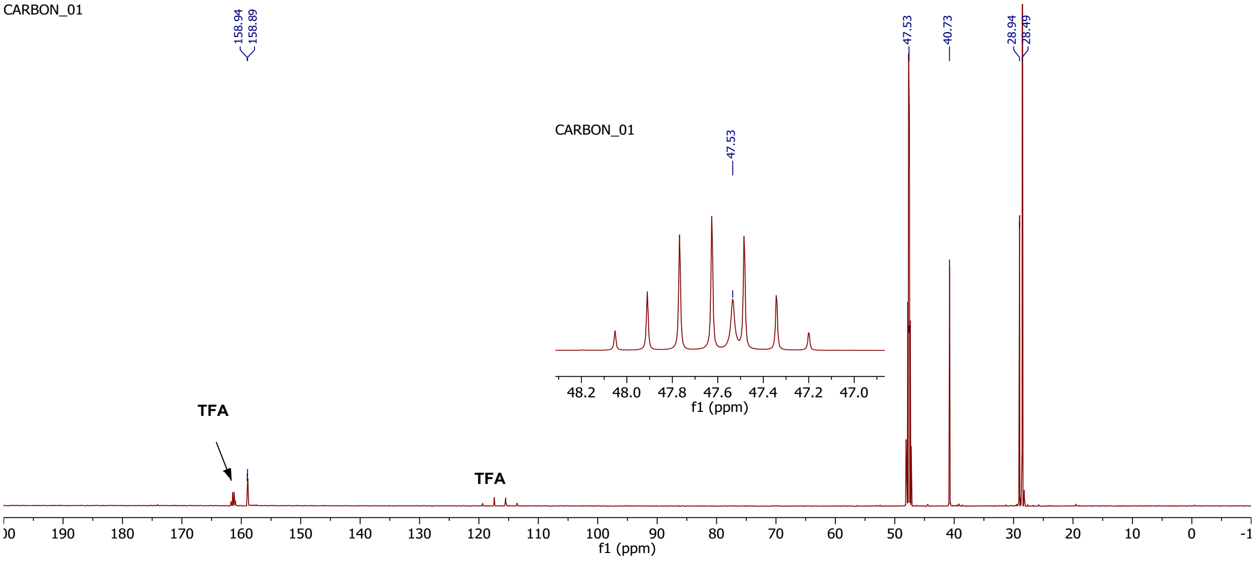
CARBON_01
158.94
158.89
47.53
40.73
28.94
28.49
CARBON_01
47.53
f1 (ppm)
TFA
TFA
f1 (ppm)

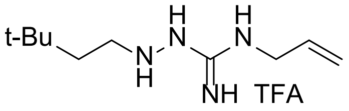

20e

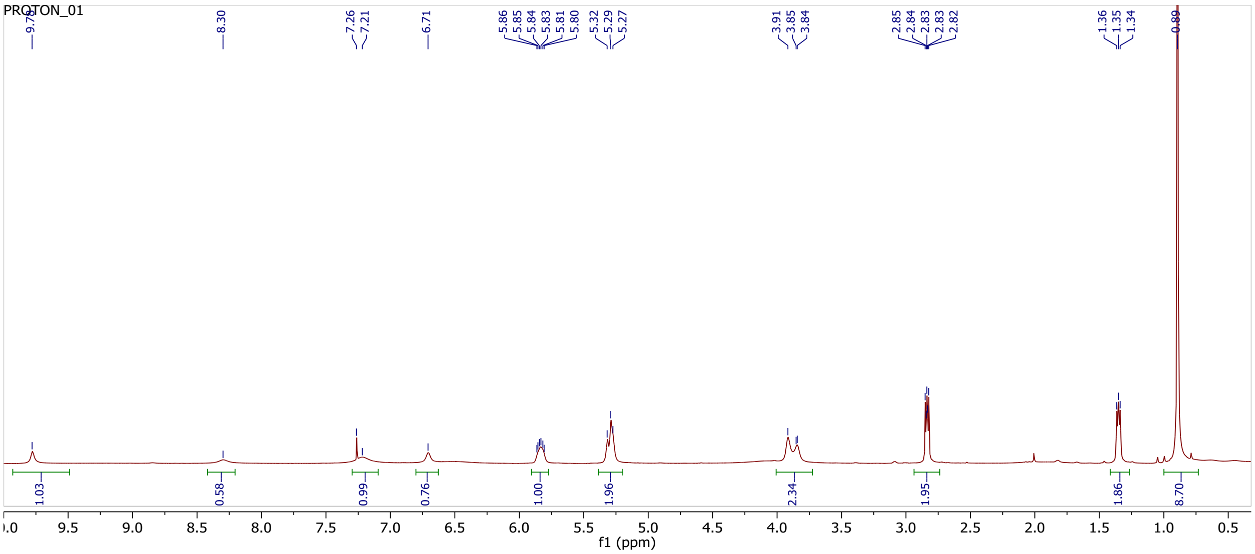

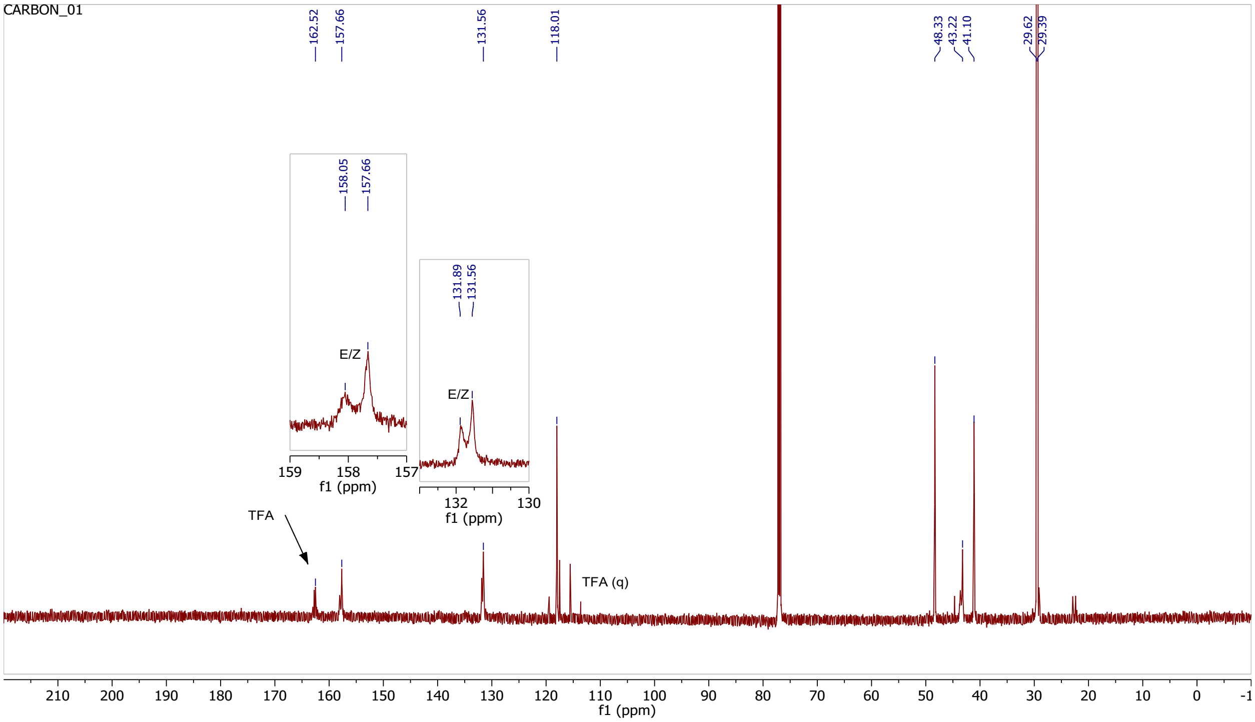

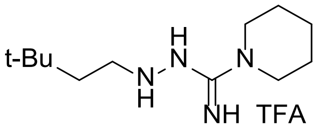

t-Bu
H
N
N
H
N
NH
TFA

20f

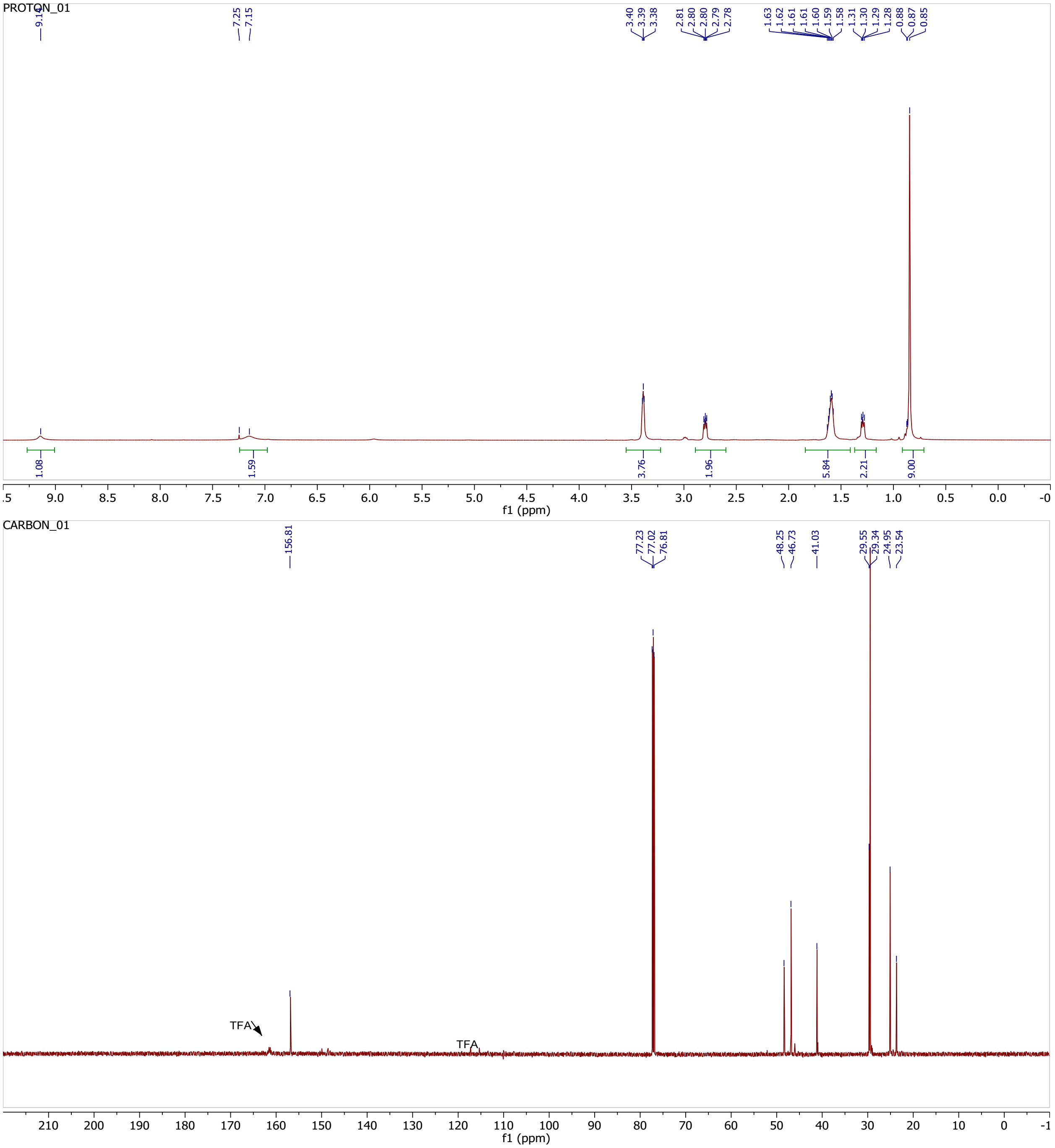

PROTON_01
9.14
7.25
7.15
3.40
3.39
3.38
2.81
2.80
2.80
2.79
2.78
1.63
1.62
1.61
1.61
1.60
1.59
1.58
1.31
1.30
1.29
1.28
0.88
0.87
0.85
1.08
1.59
3.76
1.96
5.84
2.21
9.00
f1 (ppm)
CARBON_01
156.81
77.23
77.02
76.81
48.25
46.73
41.03
29.55
29.34
24.95
23.54
TFA
TFA
f1 (ppm)

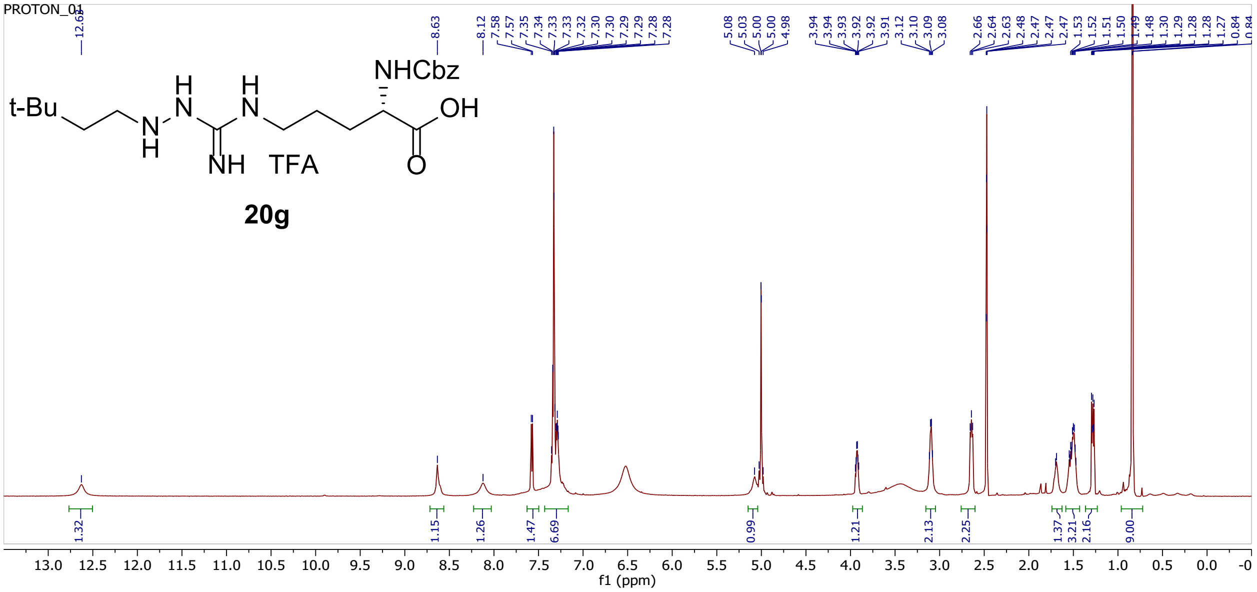
PROTON_01
t-Bu
NHCbz
OH
NH
TFA
20g
f1 (ppm)

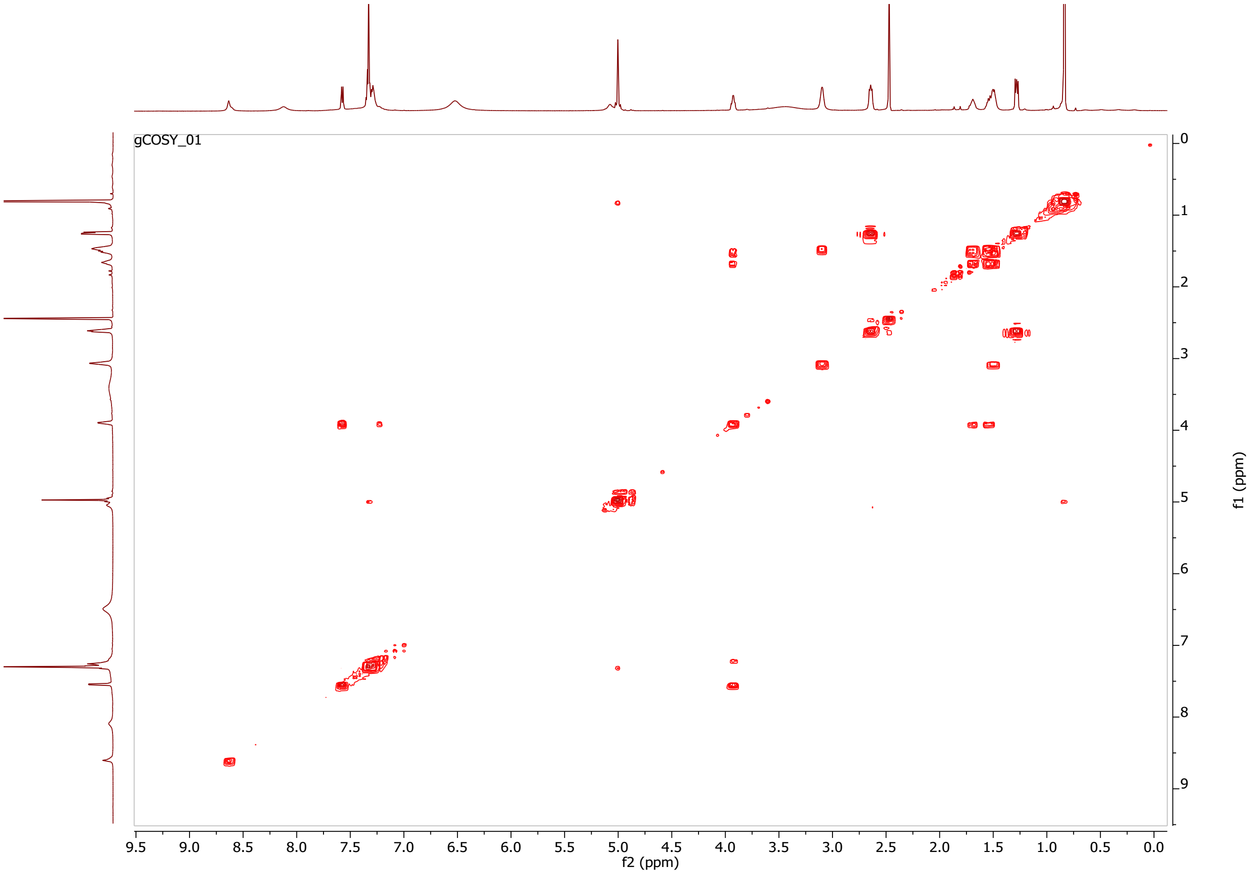
gCOSY_01
f2 (ppm)
f1 (ppm)

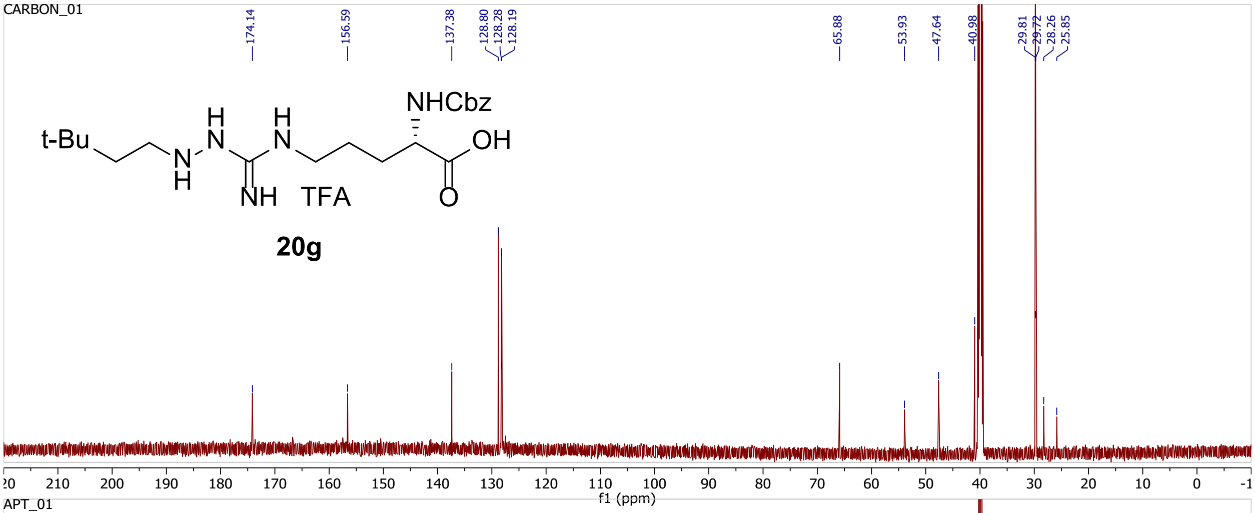
CARBON_01
174.14
156.59
137.38
128.80
128.28
128.19
65.88
53.93
47.64
40.98
29.81
29.72
28.26
25.85
NHCbz
t-Bu
H
N
H
N
N
H
NH
TFA
OH
O
20g
f1 (ppm)

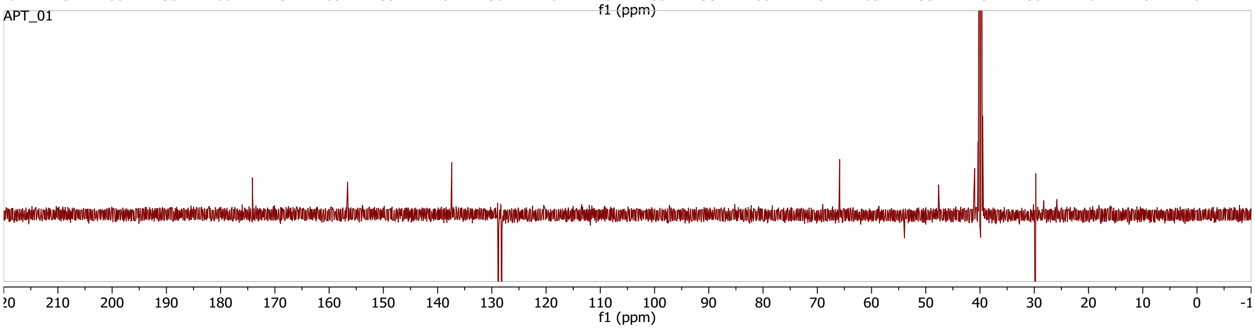
APT_01
f1 (ppm)

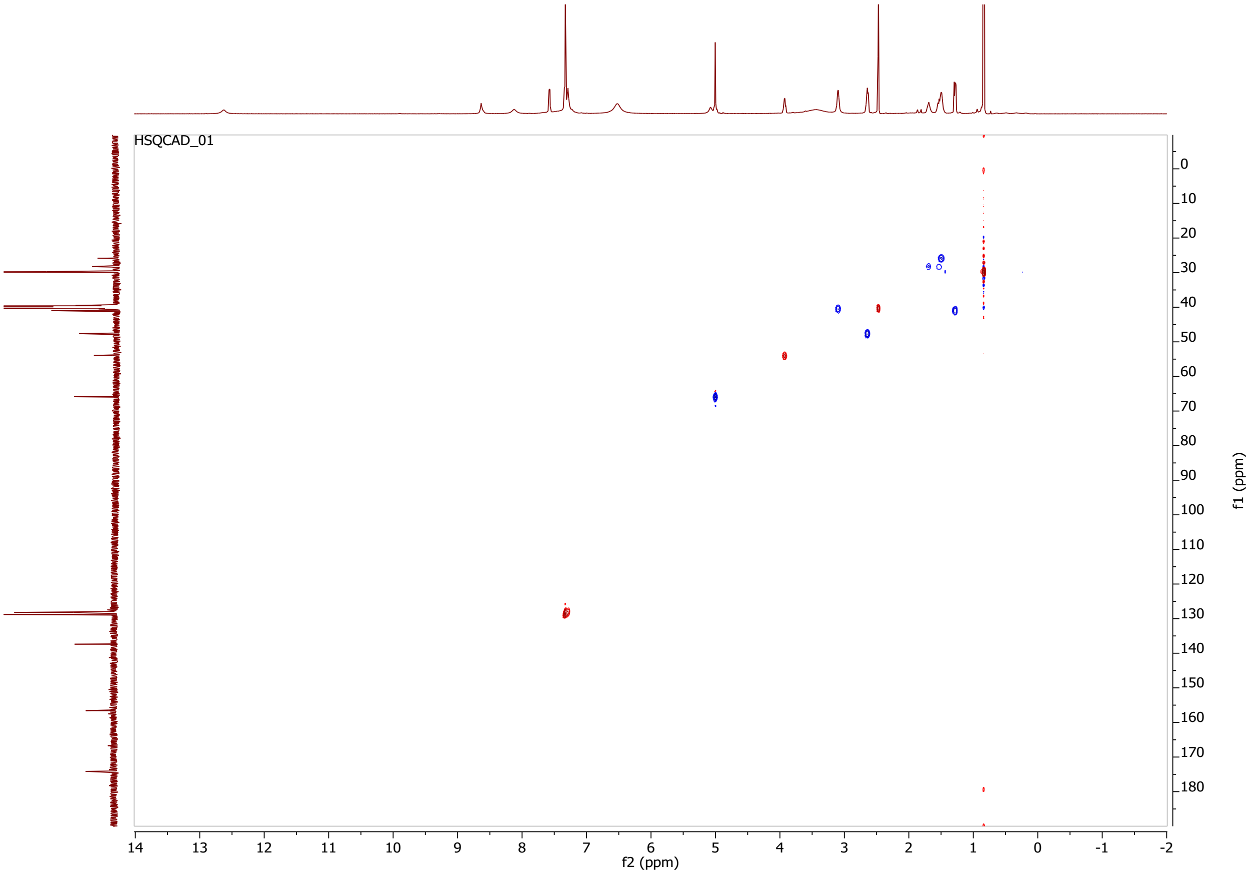
HSQCAD_01
f2 (ppm)
f1 (ppm)

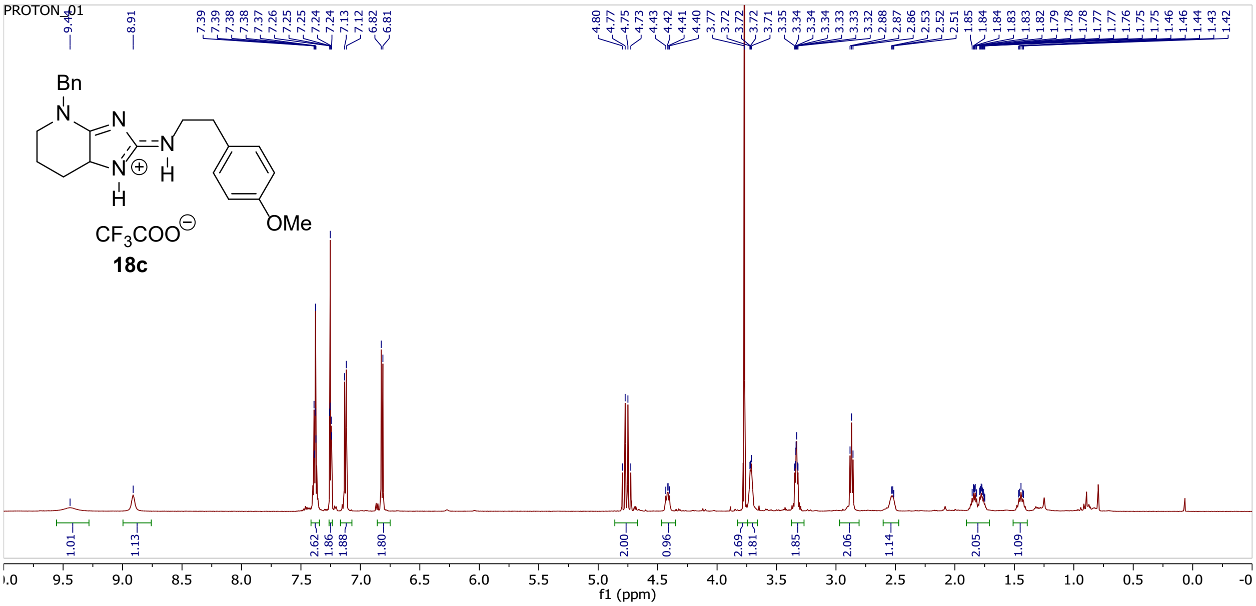

PROTON_01
Bn
N
N
N
N
H
H
CF3COO
OMe
18c
f1 (ppm)

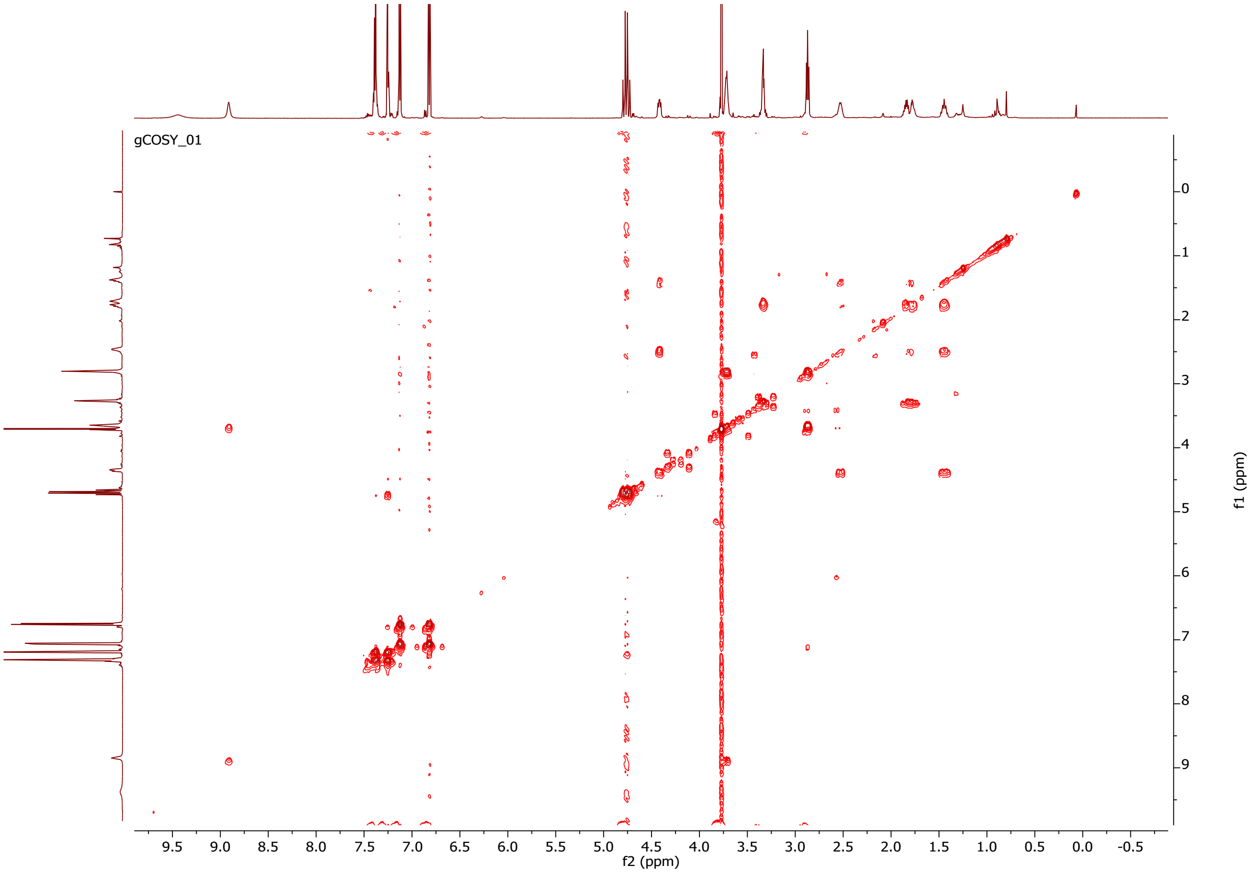

gCOSY_01
f2 (ppm)
f1 (ppm)

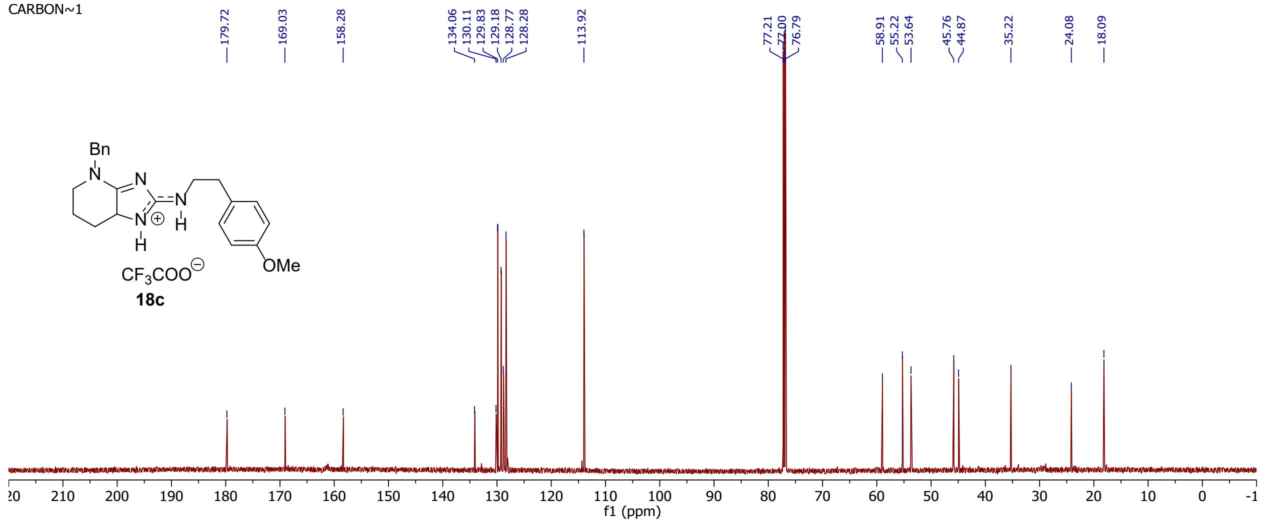
CARBON~1
179.72
169.03
158.28
134.06
130.11
129.83
129.18
128.77
128.28
113.92
77.21
77.00
76.79
58.91
55.22
53.64
45.76
44.87
35.22
24.08
18.09
Bn
N
N
N
H
H
OMe
$CF_3COO^{\ominus}$
18c
f1 (ppm)

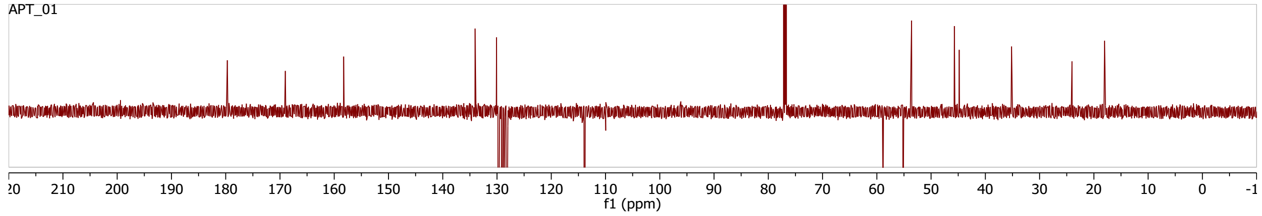
APT_01
f1 (ppm)

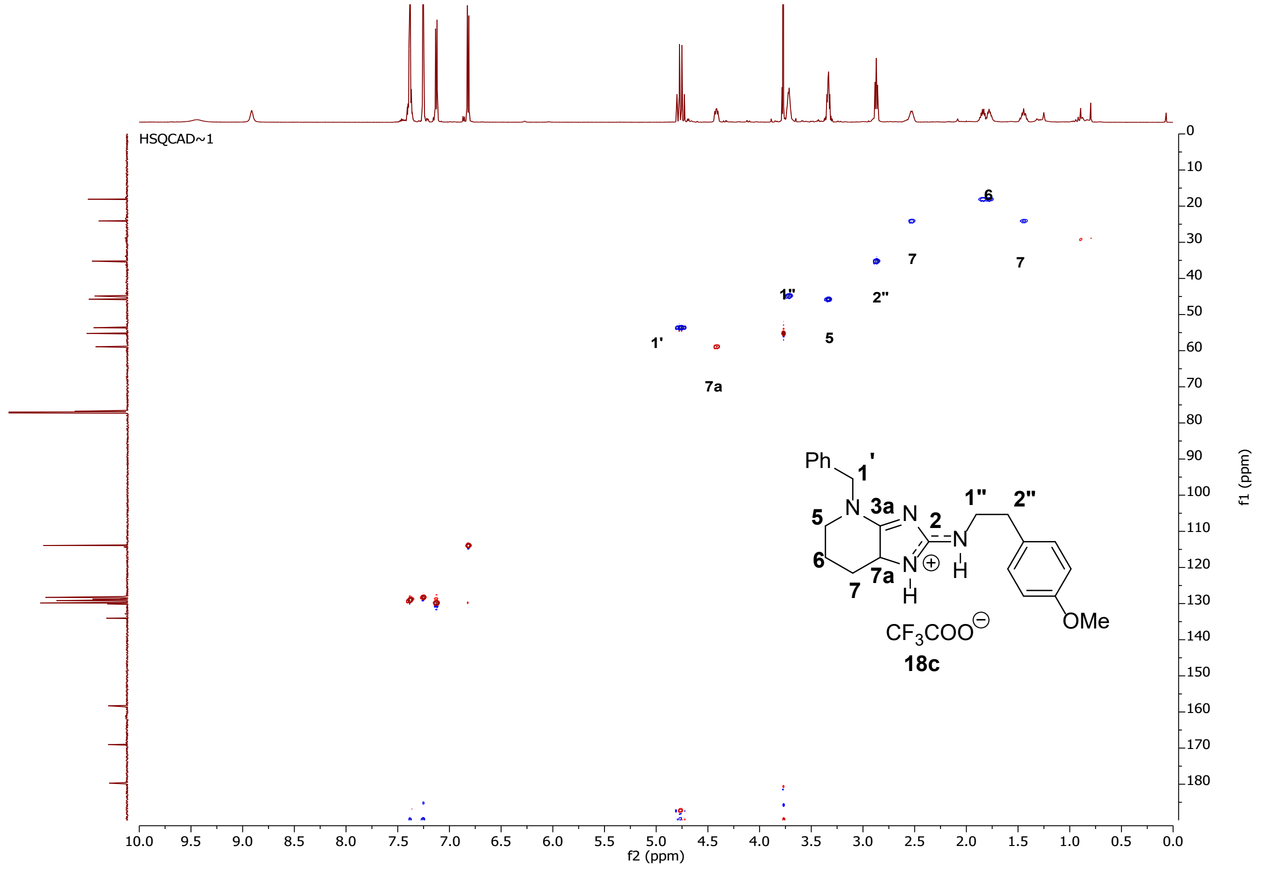
HSQCAD~1
6
7
7
1''
2''
1'
5
7a
Ph
1'
5
3a
2
1''
2''
6
7a
7
OMe
$CF_3COO^{\ominus}$
18c
f2 (ppm)
f1 (ppm)

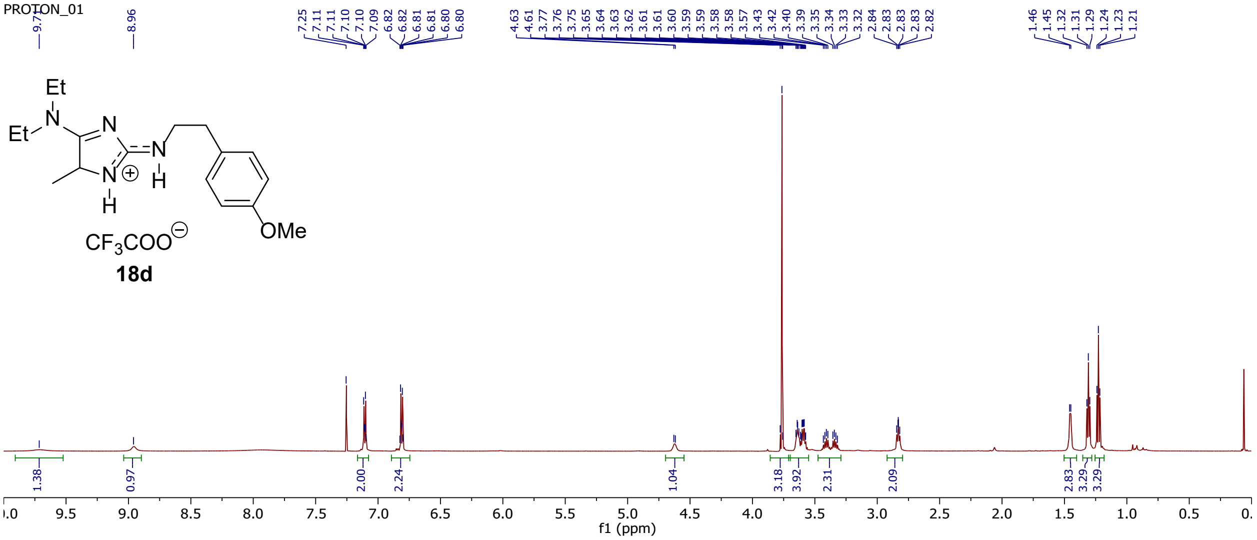
PROTON_01
18d
f1 (ppm)

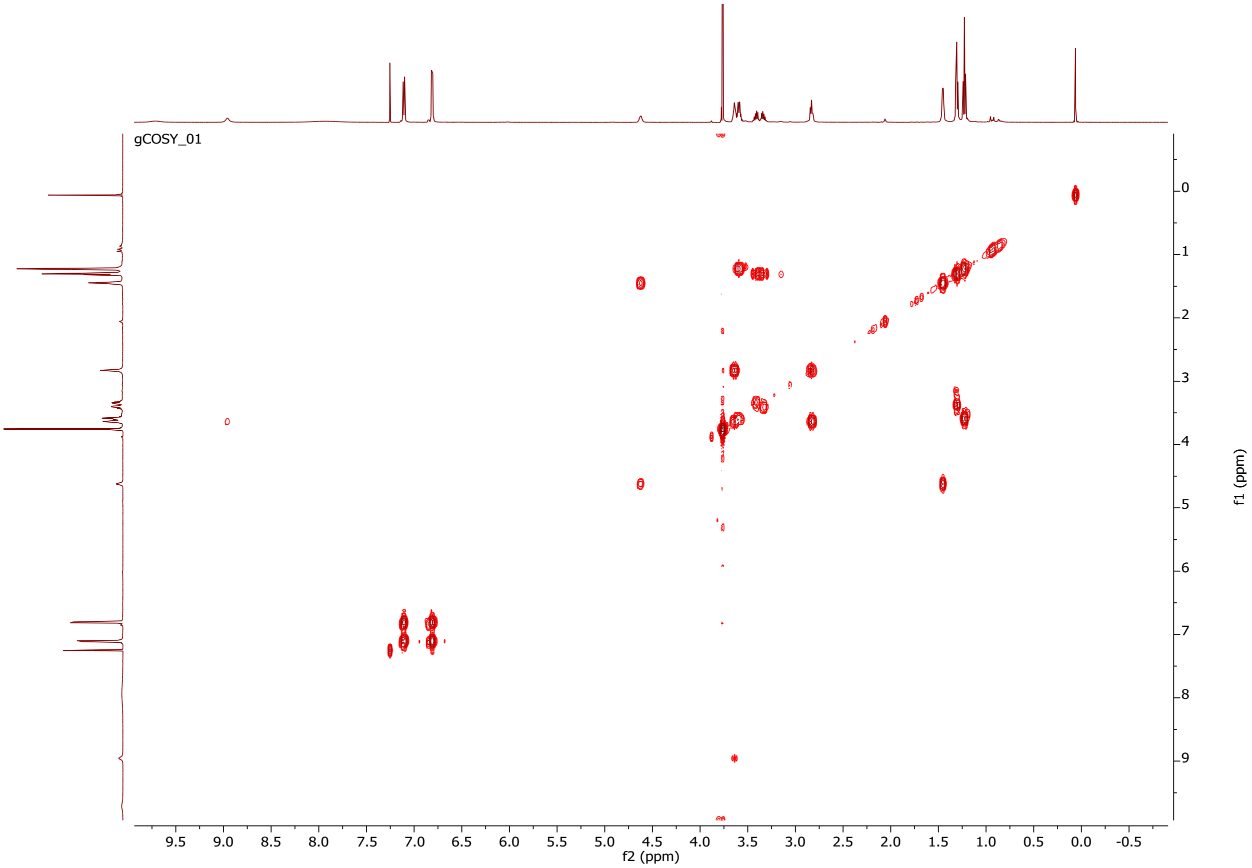
gCOSY_01
f2 (ppm)
f1 (ppm)

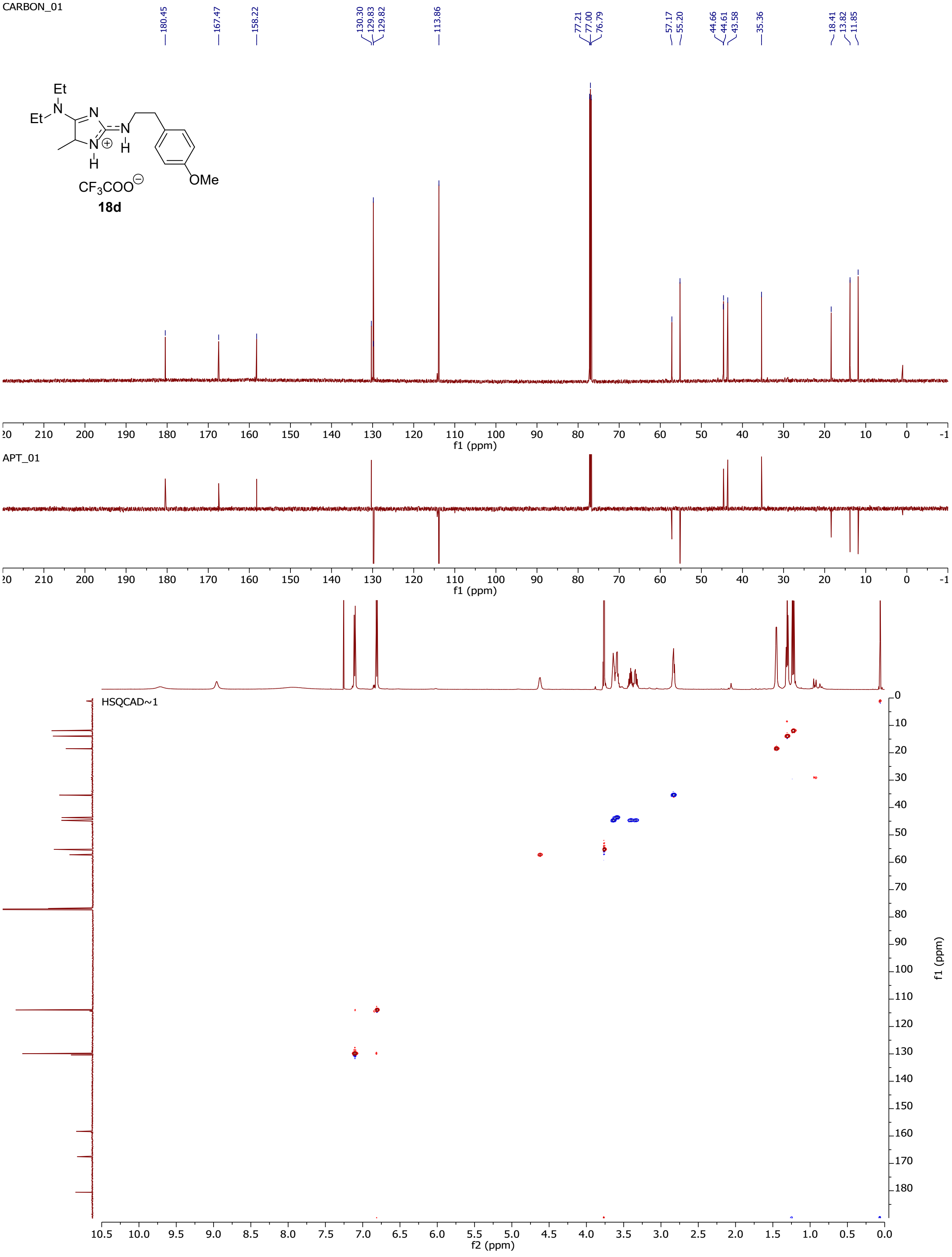
CARBON_01
180.45
167.47
158.22
130.30
129.83
129.82
113.86
77.21
77.00
76.79
57.17
55.20
44.66
44.61
43.58
35.36
18.41
13.82
11.85
Et
N
Et
N
N
H
N
H
OMe
CF3COO
18d
f1 (ppm)
APT_01
f1 (ppm)
HSQCAD~1
f1 (ppm)
f2 (ppm)

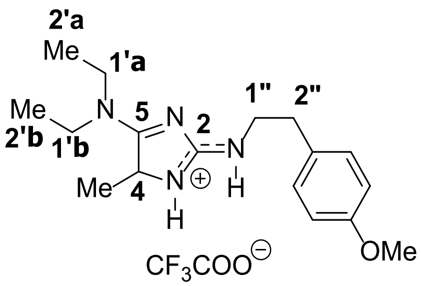
2'a
Me
1'a
Me
N
5
N
2
1"
2"
2'b
1'b
N
Me
4
N
H
H
OMe
CF3COO

18d

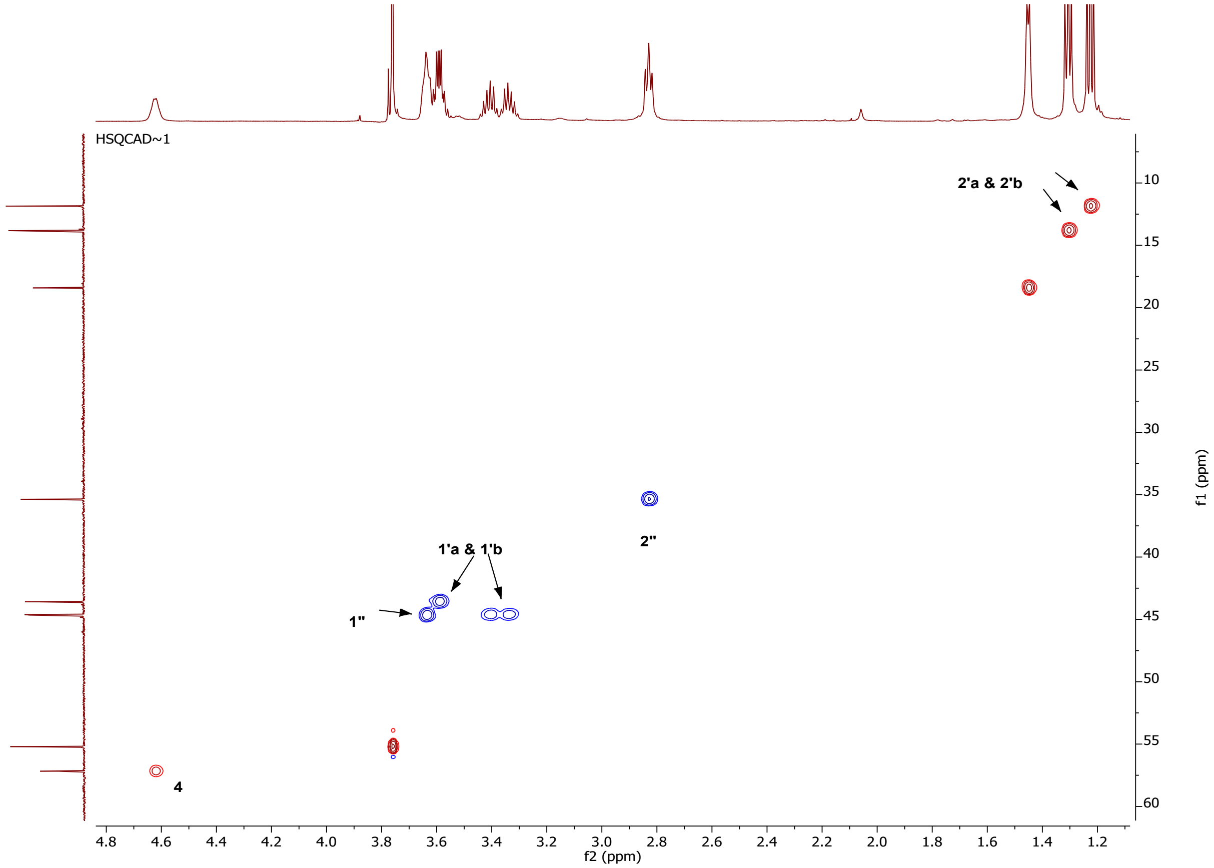
HSQCAD~1
2'a & 2'b
2"
1'a & 1'b
1"
4
4.8
4.6
4.4
4.2
4.0
3.8
3.6
3.4
3.2
3.0
2.8
2.6
2.4
2.2
2.0
1.8
1.6
1.4
1.2
f2 (ppm)
10
15
20
25
30
35
40
45
50
55
60
f1 (ppm)

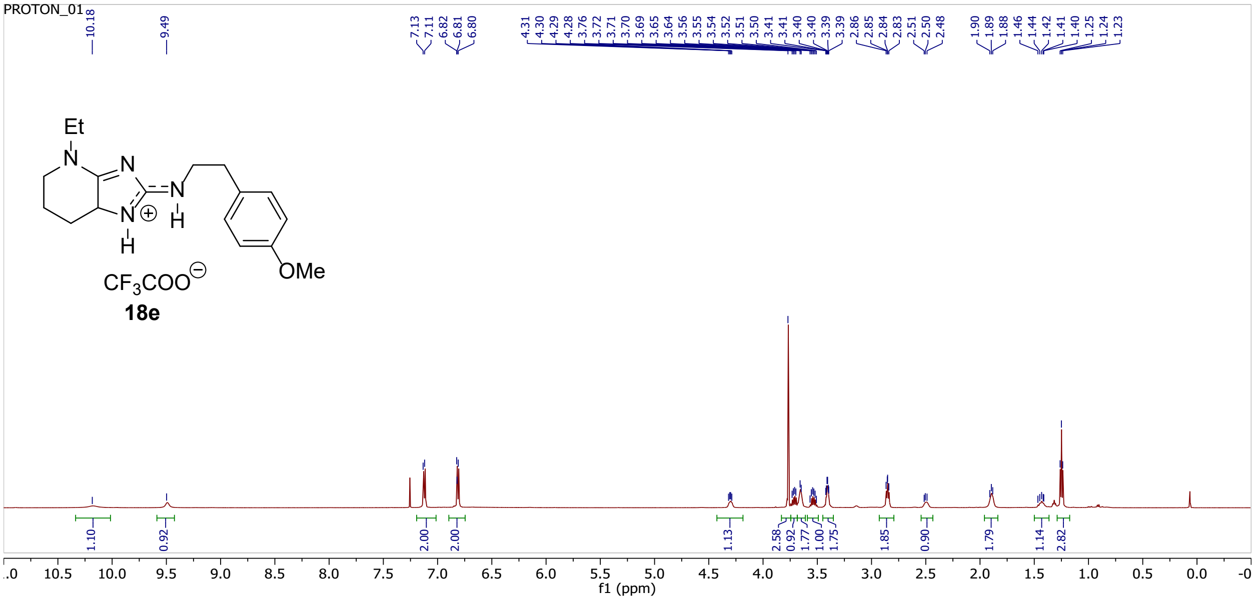
PROTON_01
10.18
9.49
7.13
7.11
6.82
6.81
6.80
4.31
4.30
4.29
4.28
3.76
3.72
3.71
3.70
3.69
3.65
3.64
3.56
3.55
3.54
3.52
3.51
3.50
3.41
3.40
3.40
3.39
3.39
2.86
2.85
2.84
2.83
2.51
2.50
2.48
1.90
1.89
1.88
1.46
1.44
1.42
1.41
1.40
1.25
1.24
1.23
Et
N
N
N
N
H
H
CF3COO
OMe
18e
1.10
0.92
2.00
2.00
1.13
2.58
0.92
1.77
1.00
1.75
1.85
0.90
1.79
1.14
2.82
f1 (ppm)

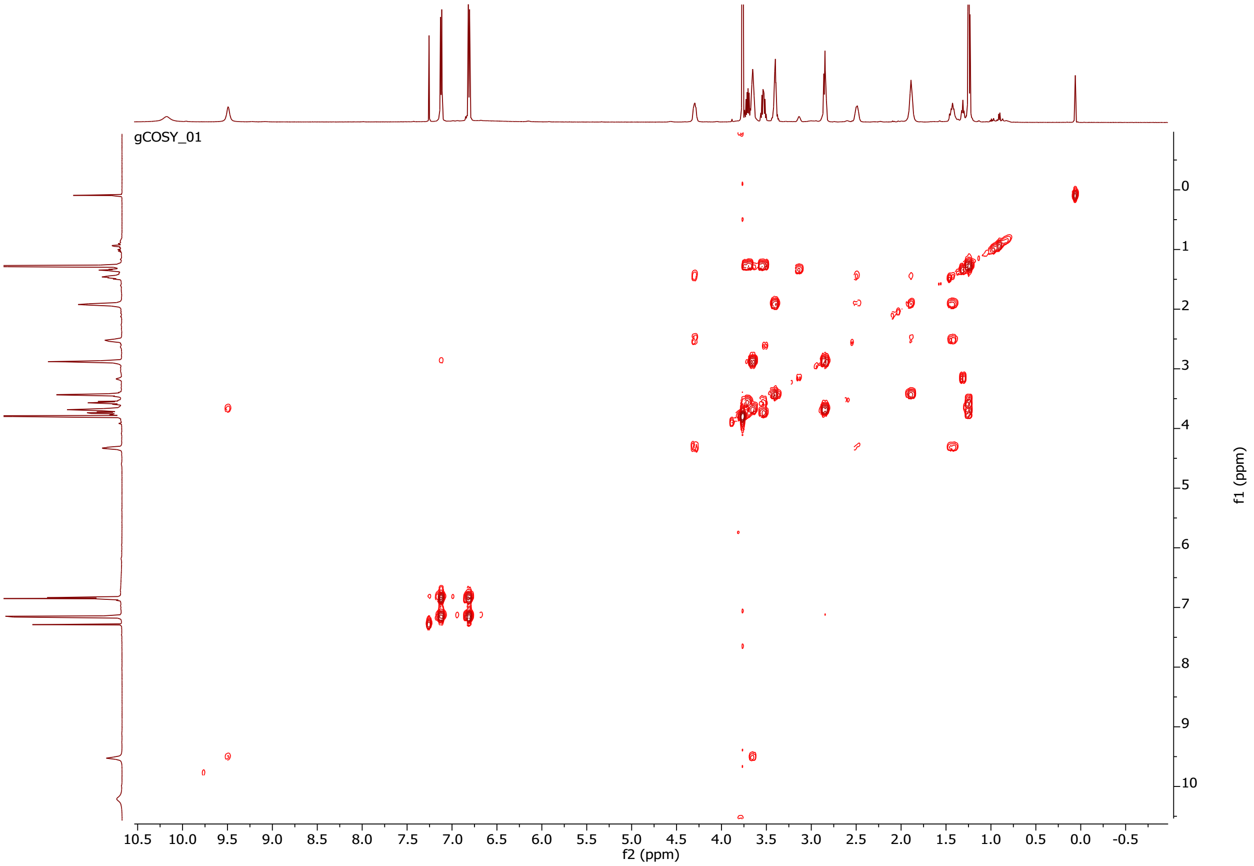
gCOSY_01
f2 (ppm)
f1 (ppm)

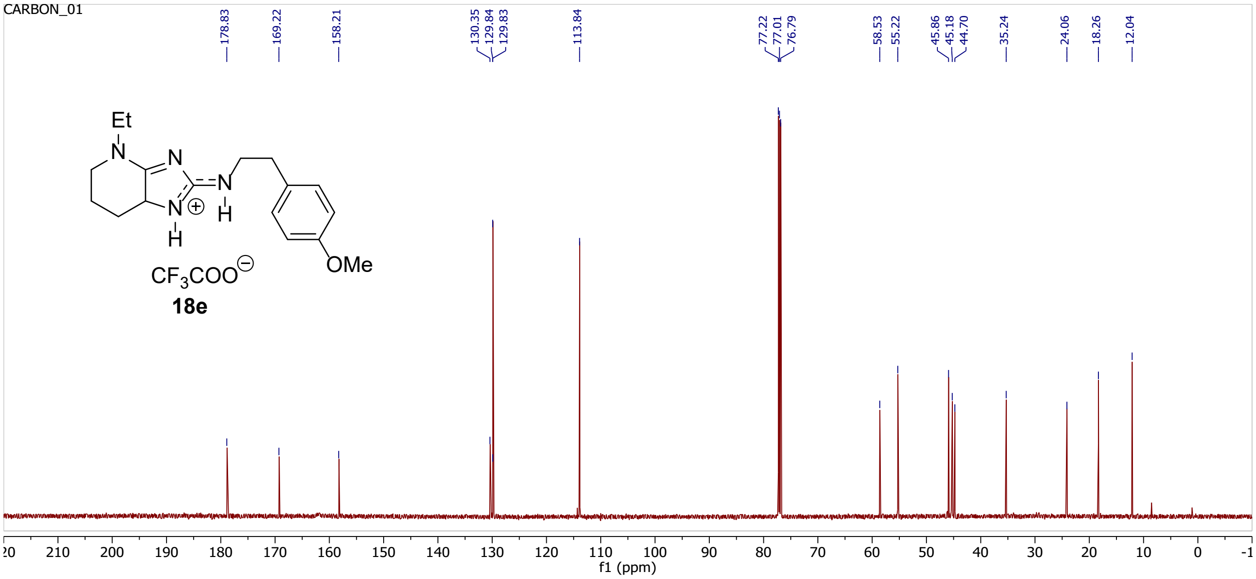
CARBON_01
178.83
169.22
158.21
130.35
129.84
129.83
113.84
77.22
77.01
76.79
58.53
55.22
45.86
45.18
44.70
35.24
24.06
18.26
12.04
Et
N
N
N
H
N
H
OMe
CF3COO
18e
f1 (ppm)

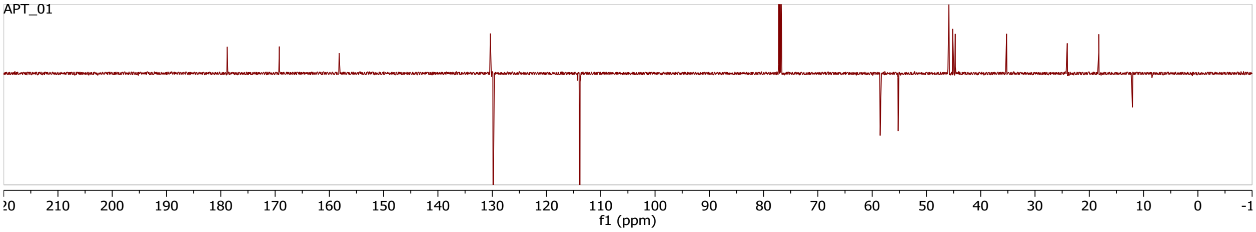
APT_01
f1 (ppm)

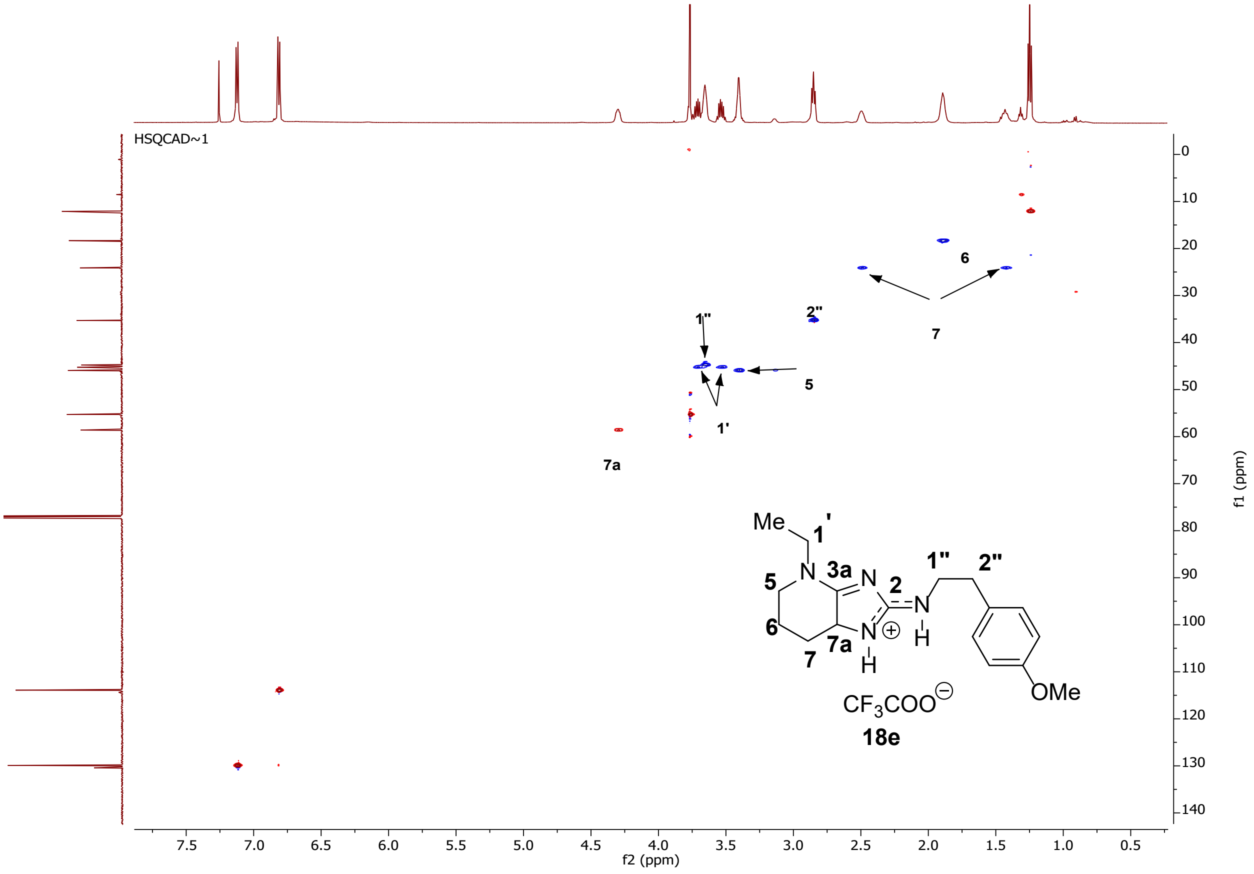
HSQCAD~1
6
7
2"
1"
5
1'
7a
Me
1'
5
N
3a
N
2
1"
2"
6
N
7
7a
N
H
H
OMe
CF3COO
18e
f1 (ppm)
f2 (ppm)

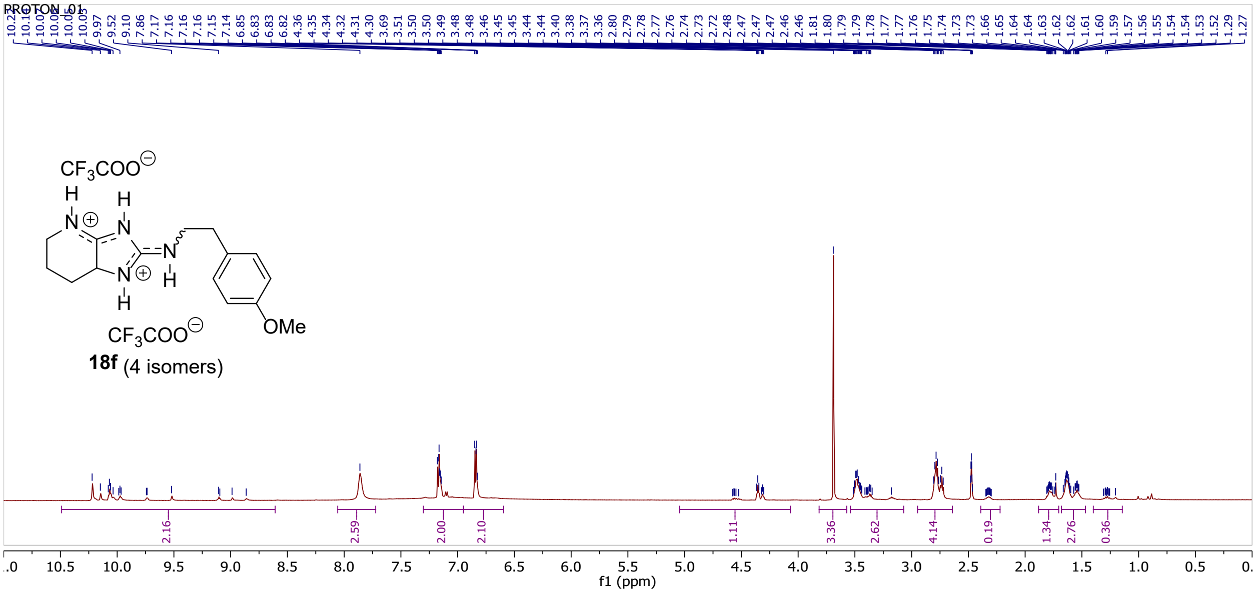

PROTON_01
CF3COO⊖
18f (4 isomers)
OMe
2.16
2.59
2.00
2.10
1.11
3.36
2.62
4.14
0.19
1.34
2.76
0.36
f1 (ppm)

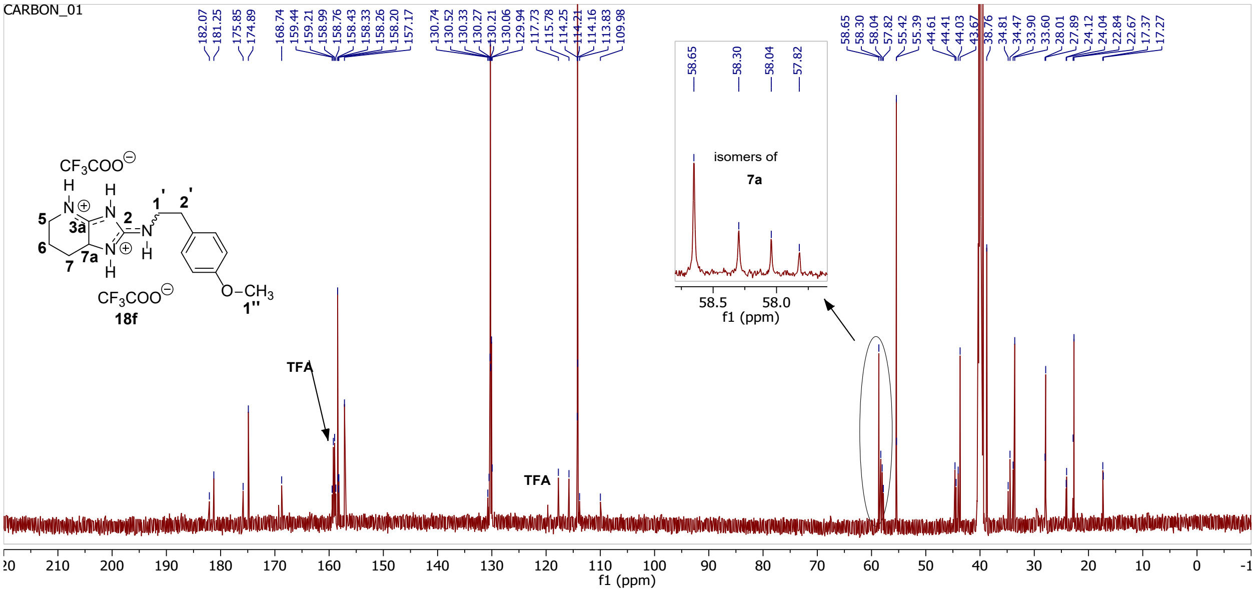

CARBON_01
182.07
181.25
175.85
174.89
168.74
159.44
159.21
158.99
158.76
158.43
158.33
158.26
158.20
157.17
130.74
130.52
130.33
130.27
130.21
130.06
129.94
117.73
115.78
114.25
114.21
114.16
113.83
109.98
58.65
58.30
58.04
57.82
55.42
55.39
44.61
44.41
44.03
43.67
38.76
34.81
34.47
33.90
33.60
28.01
27.89
24.12
24.04
22.84
22.67
17.37
17.27
isomers of
7a
TFA
TFA
18f
f1 (ppm)

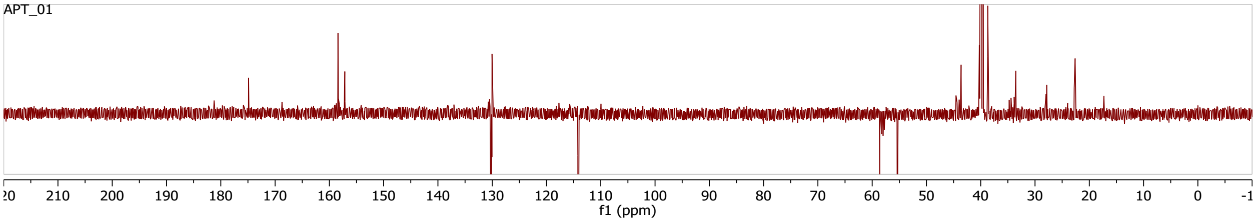

APT_01
f1 (ppm)

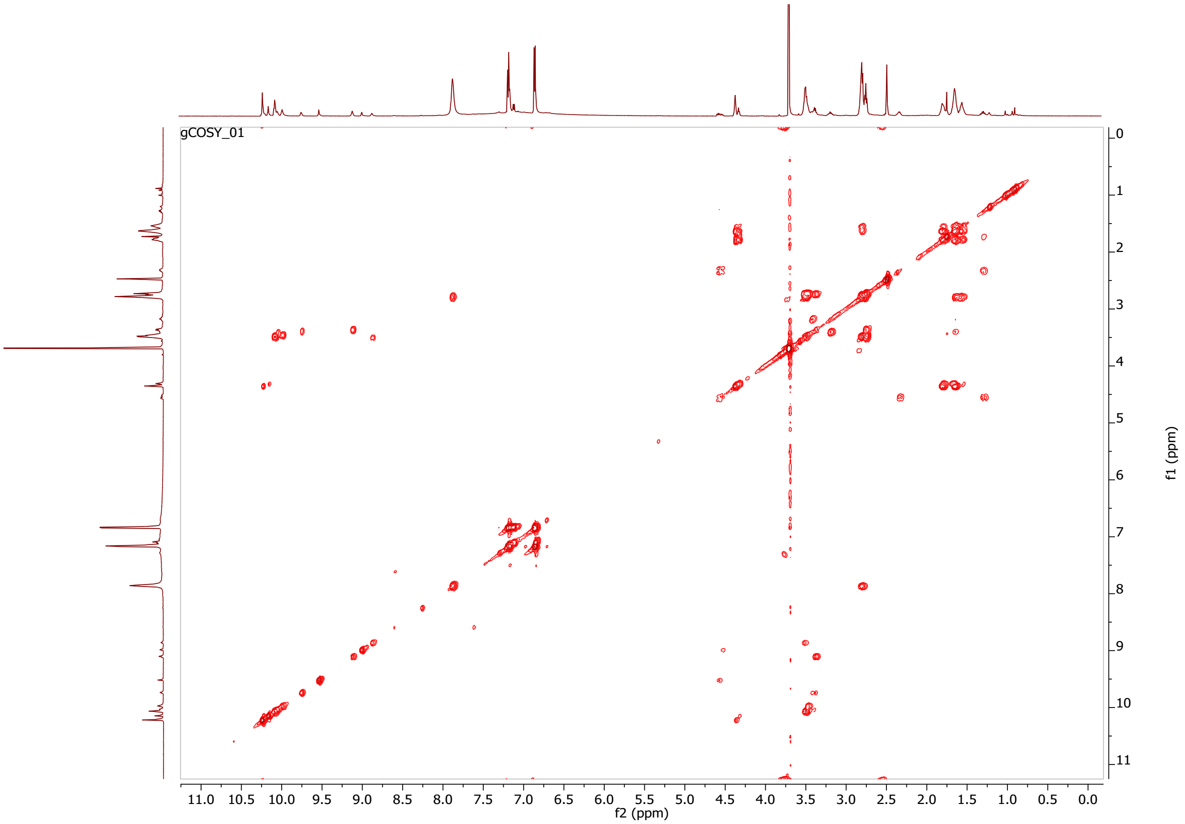
gCOSY_01
f2 (ppm)
f1 (ppm)

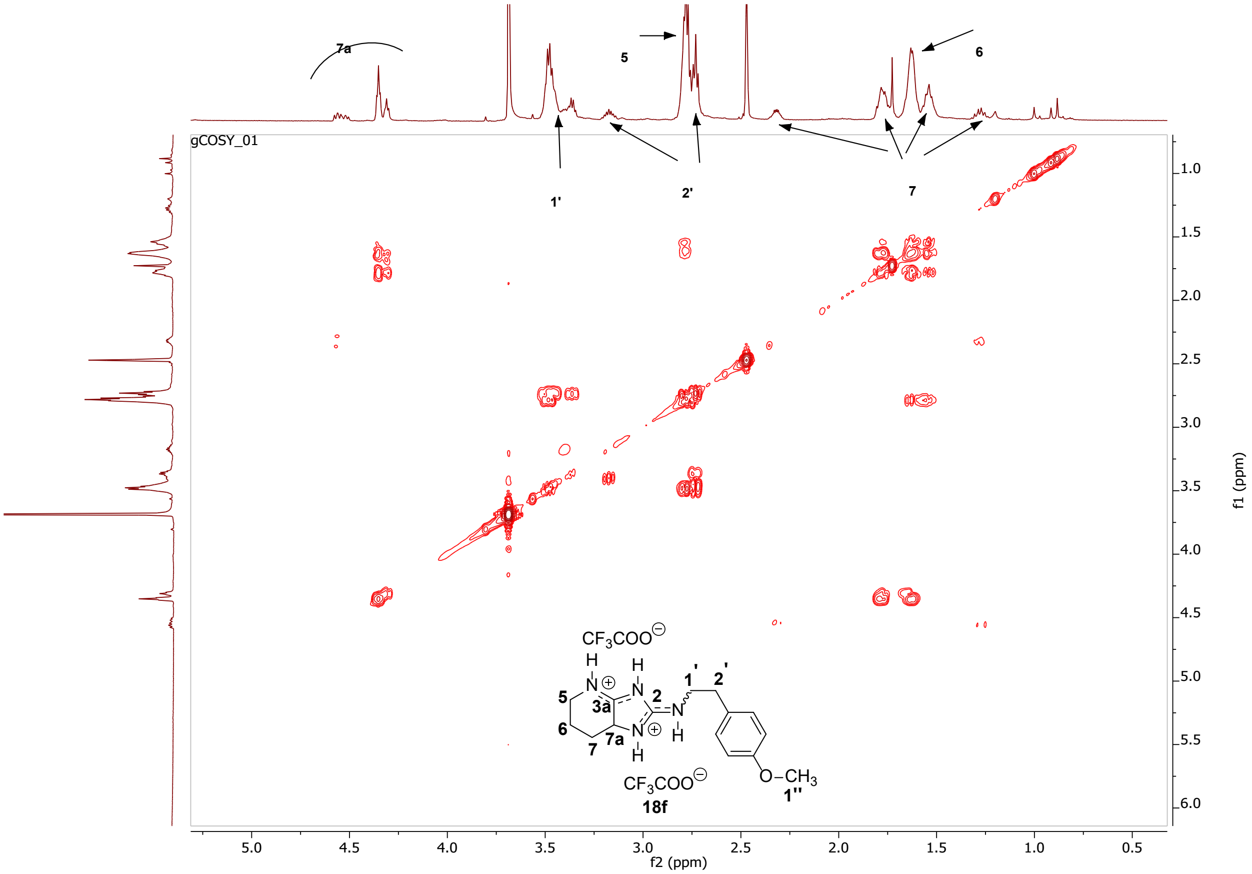
gCOSY_01
7a
5
6
1'
2'
7
f2 (ppm)
f1 (ppm)
CF3COO
18f
1''

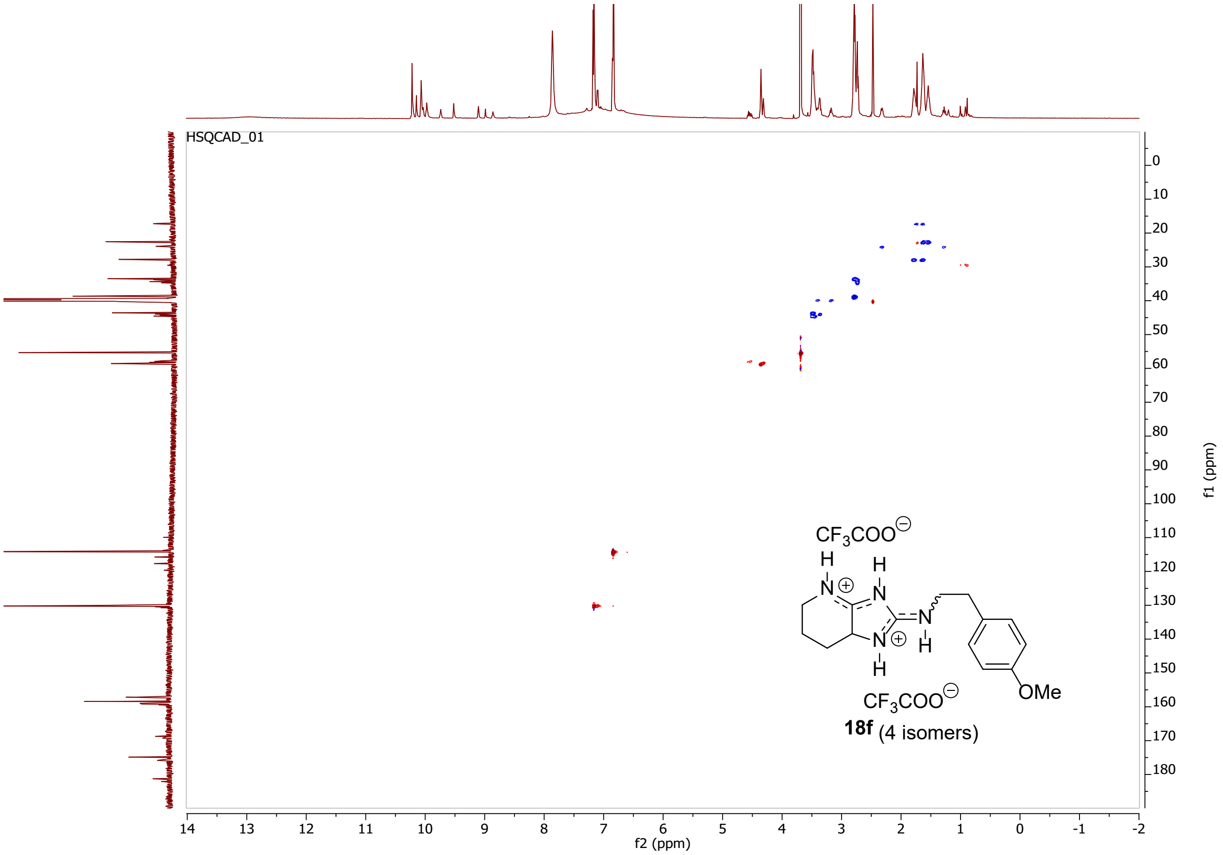
HSQCAD_01
f2 (ppm)
f1 (ppm)
CF3COO
H
N
H
N
N
N
H
H
CF3COO
OMe
18f (4 isomers)

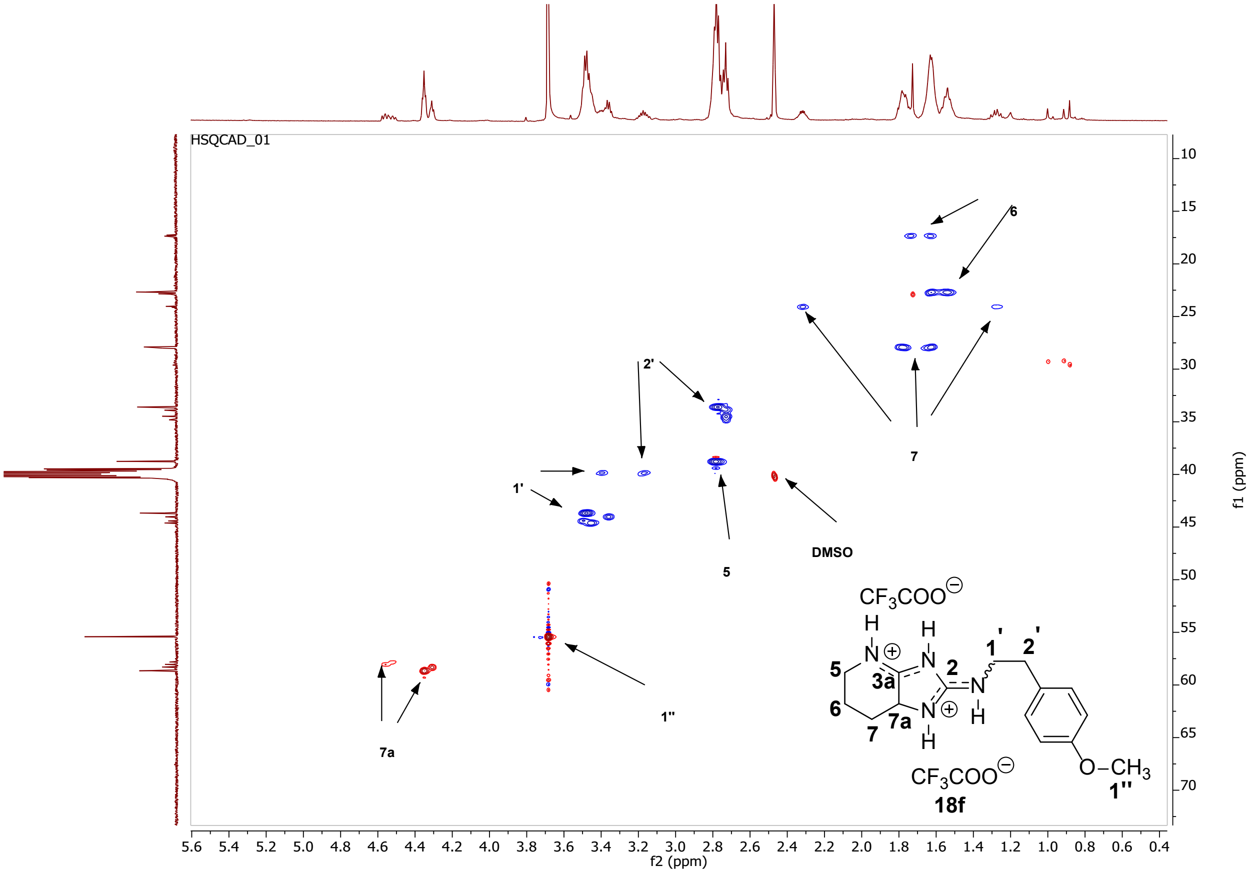
HSQCAD_01
f2 (ppm)
f1 (ppm)
6
2'
7
1'
5
DMSO
1''
7a
CF3COO
H
N
H
N
1'
2'
5
3a
2
N
6
7
7a
N
H
H
O–CH3
1''
CF3COO
18f

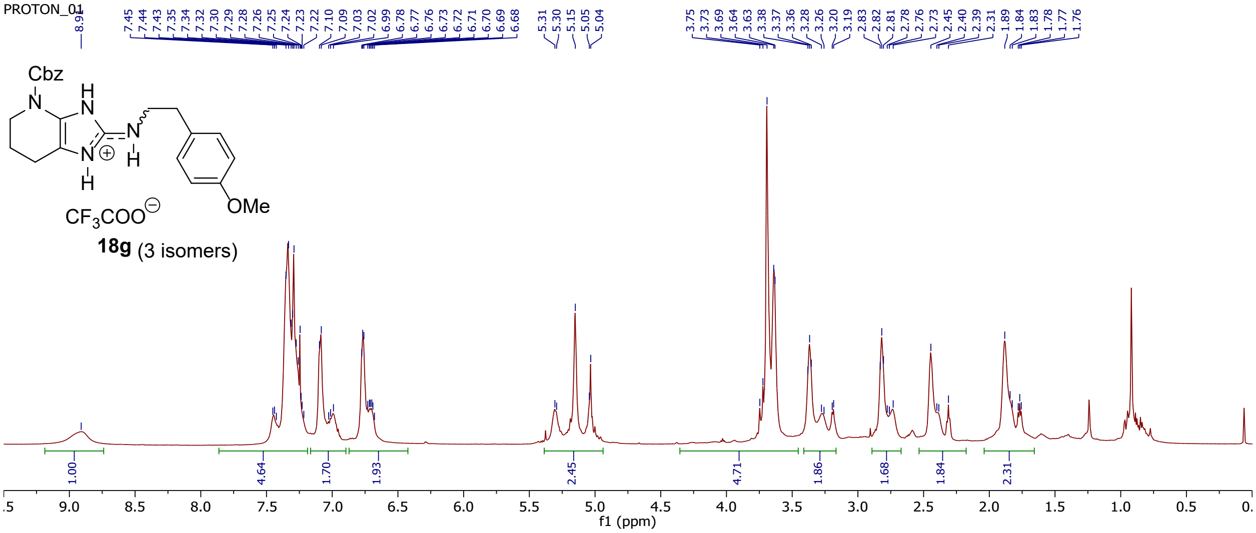
PROTON_01
8.91
7.45 7.44 7.43 7.35 7.34 7.32 7.30 7.29 7.28 7.26 7.25 7.24 7.23 7.22 7.10 7.09 7.03 7.02 6.99 6.78 6.77 6.76 6.73 6.72 6.71 6.70 6.69 6.68
5.31 5.30 5.15 5.05 5.04
3.75 3.73 3.69 3.64 3.63 3.38 3.37 3.36 3.28 3.26 3.20 3.19 2.83 2.82 2.81 2.78 2.76 2.73 2.45 2.40 2.39 2.31 1.89 1.84 1.83 1.78 1.77 1.76
Cbz
N
H
N
N
H
N
H
OMe
CF3COO
18g (3 isomers)
1.00 4.64 1.70 1.93 2.45 4.71 1.86 1.68 1.84 2.31
f1 (ppm)

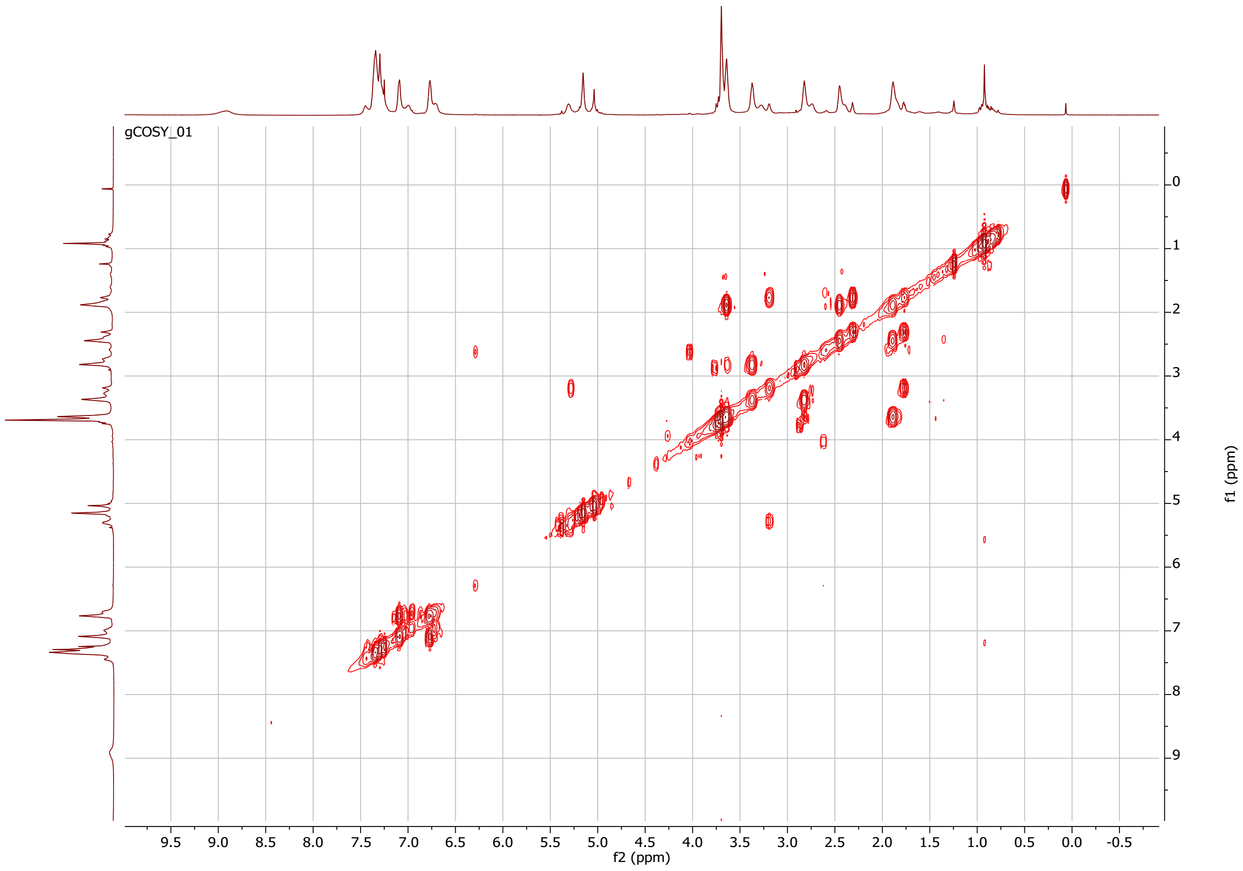
gCOSY_01
f1 (ppm)
f2 (ppm)

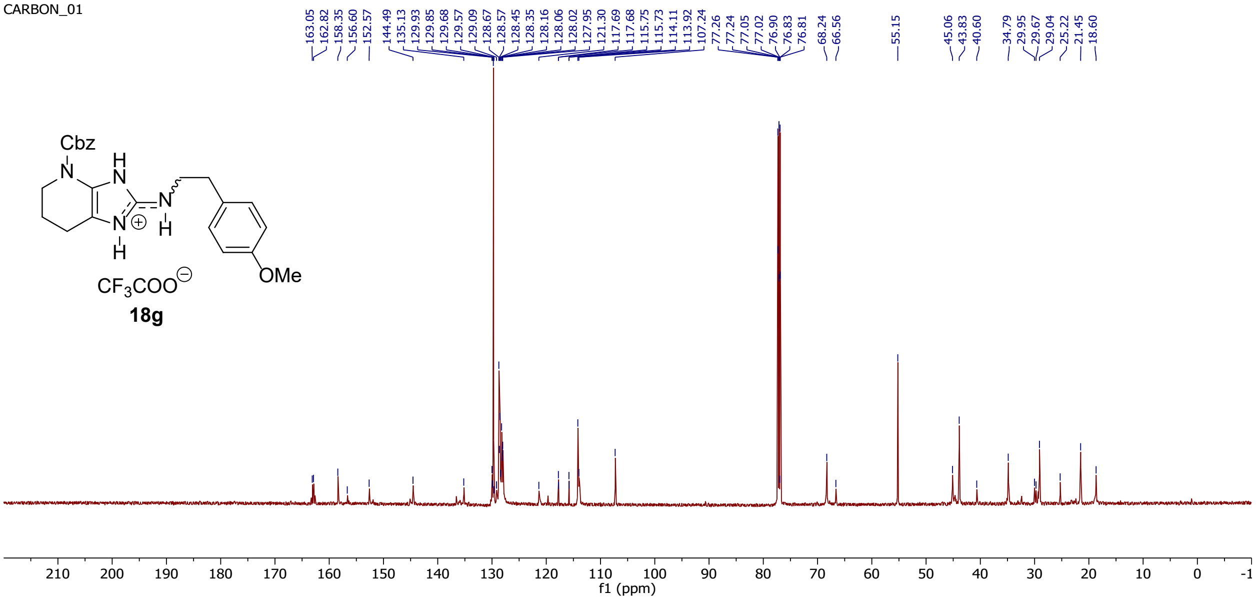
CARBON_01
163.05 162.82 158.35 156.60 152.57 144.49 135.13 129.93 129.85 129.68 129.57 129.09 128.67 128.57 128.45 128.35 128.16 128.06 128.02 127.95 121.30 117.69 117.68 115.75 115.73 114.11 113.92 107.24 77.26 77.24 77.05 77.02 76.90 76.83 76.81 68.24 66.56 55.15 45.06 43.83 40.60 34.79 29.95 29.67 29.04 25.22 21.45 18.60
Cbz
N
H
N
N
H
N
H
CF3COO
OMe
18g
f1 (ppm)

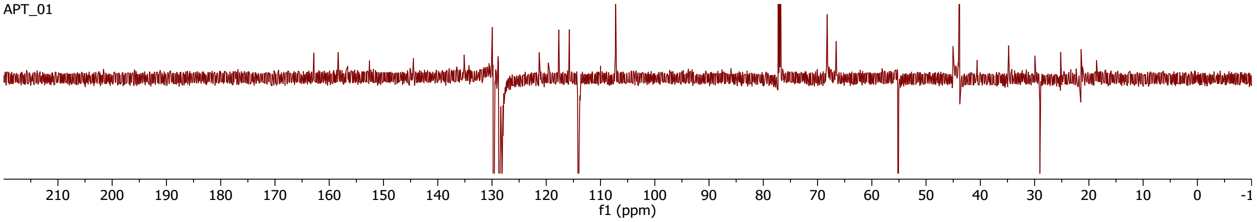
APT_01
f1 (ppm)

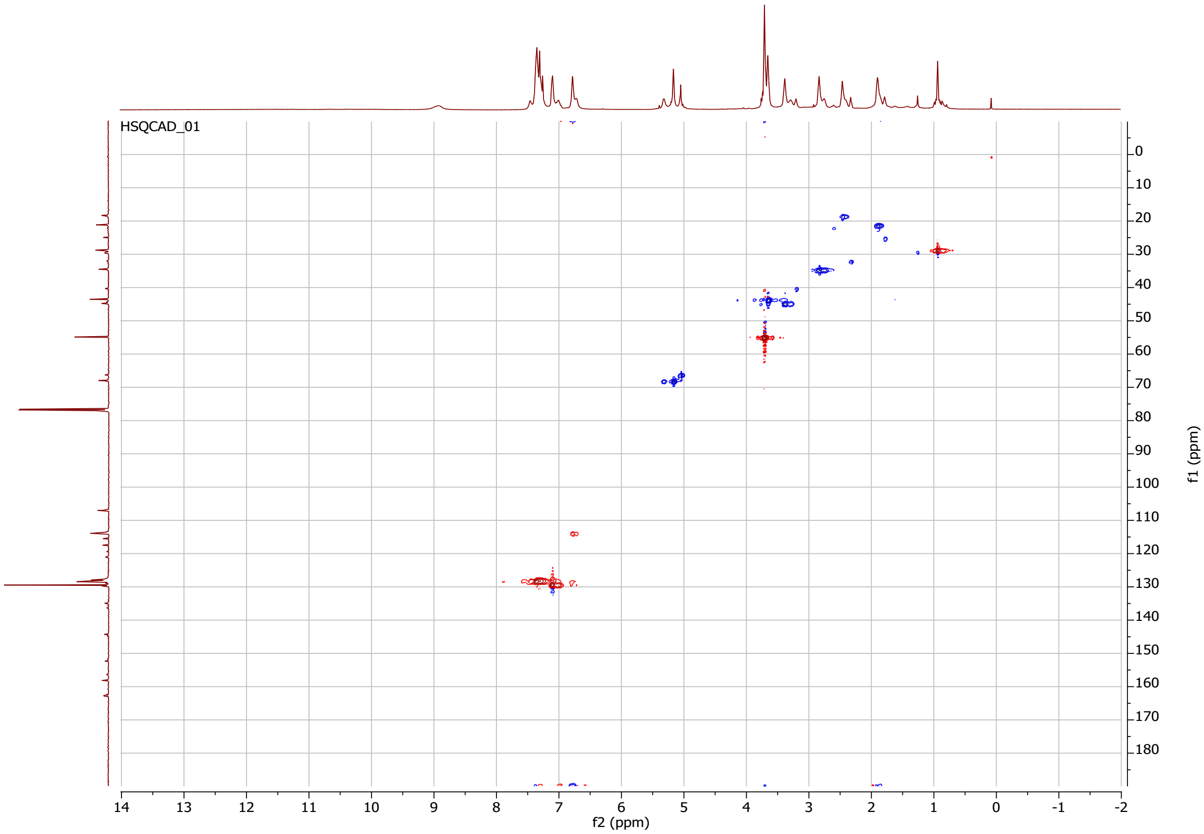
HSQCAD_01
f2 (ppm)
f1 (ppm)

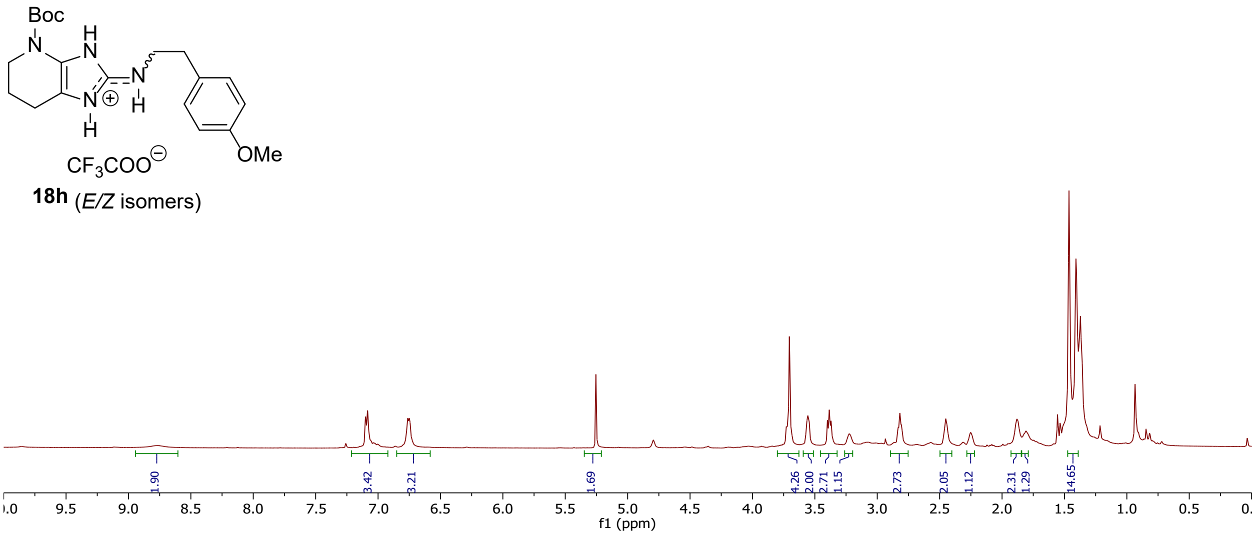
Boc
OMe
CF3COO
18h (E/Z isomers)
f1 (ppm)

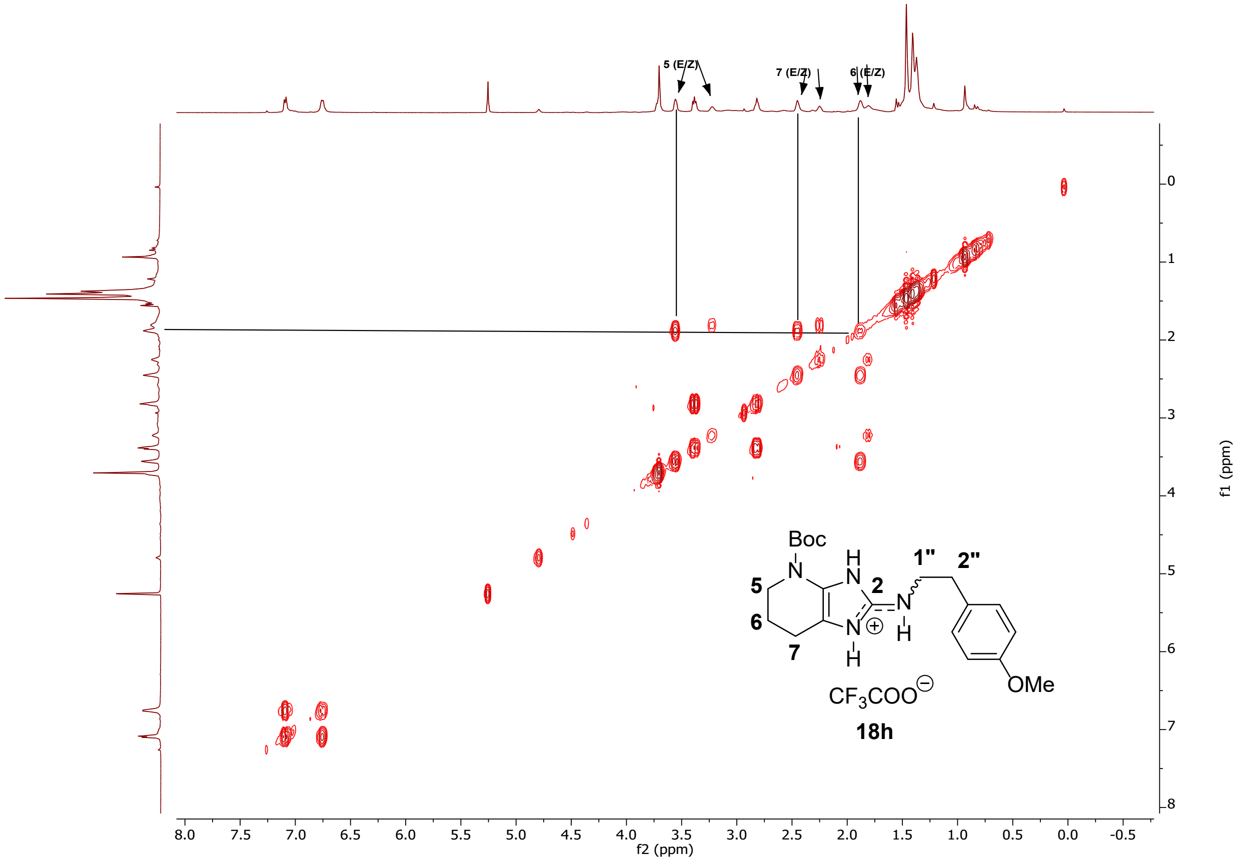
5 (E/Z)
7 (E/Z)
6 (E/Z)
Boc
1"
2"
5
6
7
2
OMe
CF3COO
18h
f2 (ppm)
f1 (ppm)

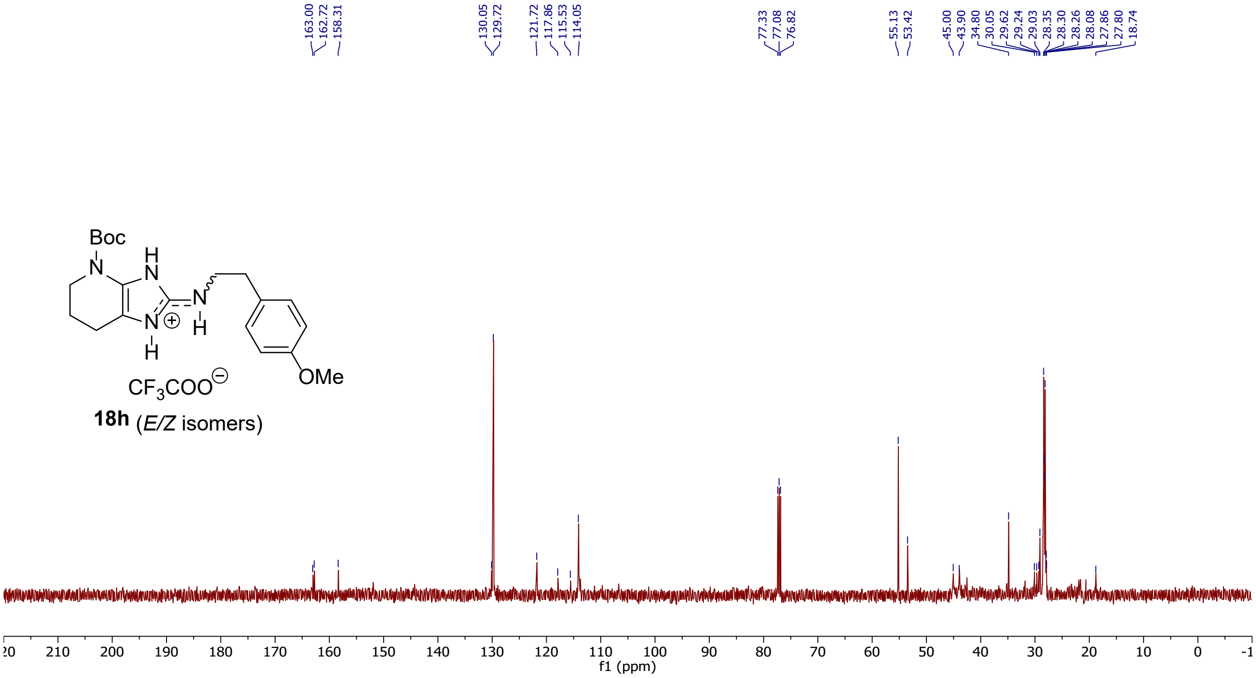

18h (*E*/*Z* isomers)

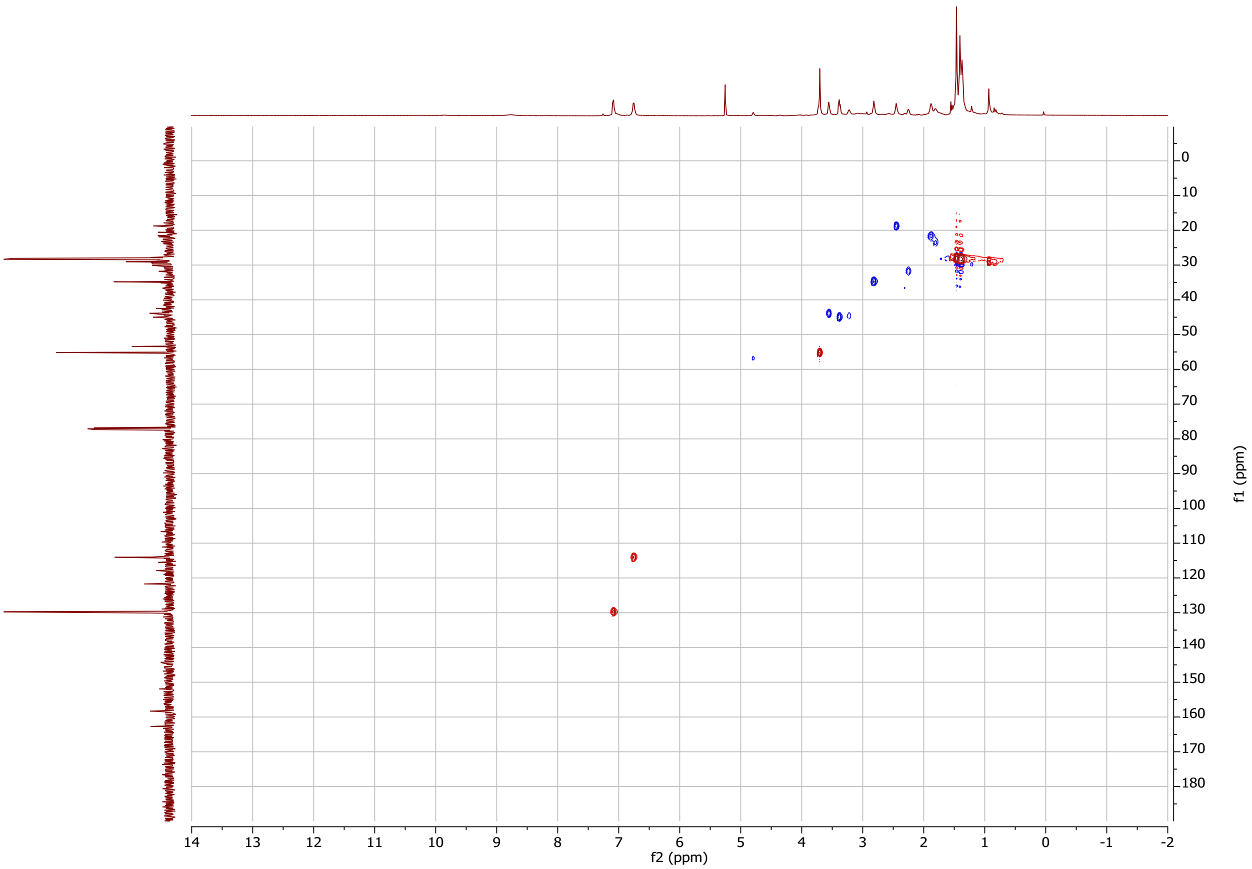

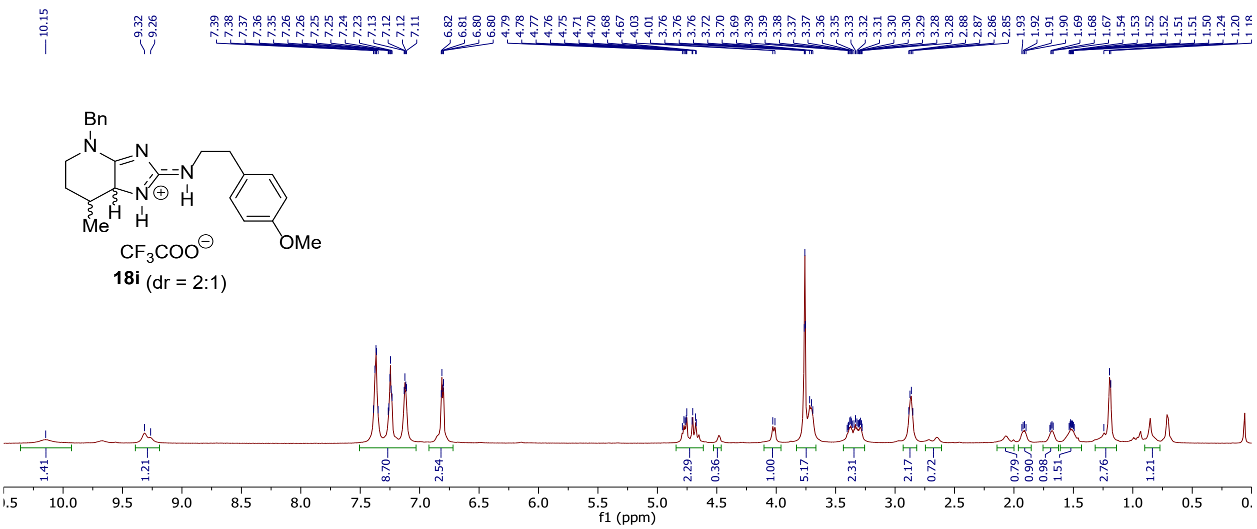

Bn
N
N
N
H
N
H
H
Me
CF3COO
OMe
18i (dr = 2:1)
f1 (ppm)

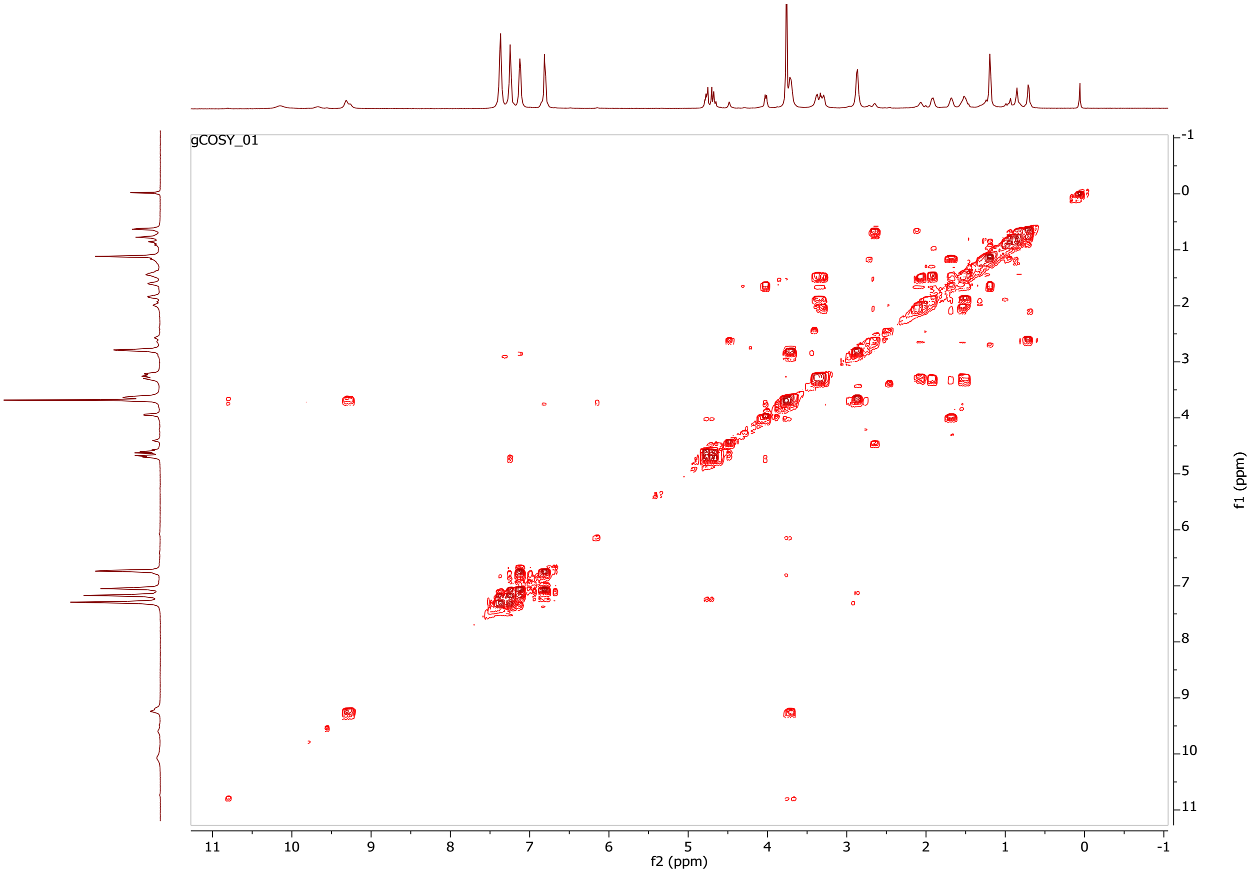

gCOSY_01
f2 (ppm)
f1 (ppm)

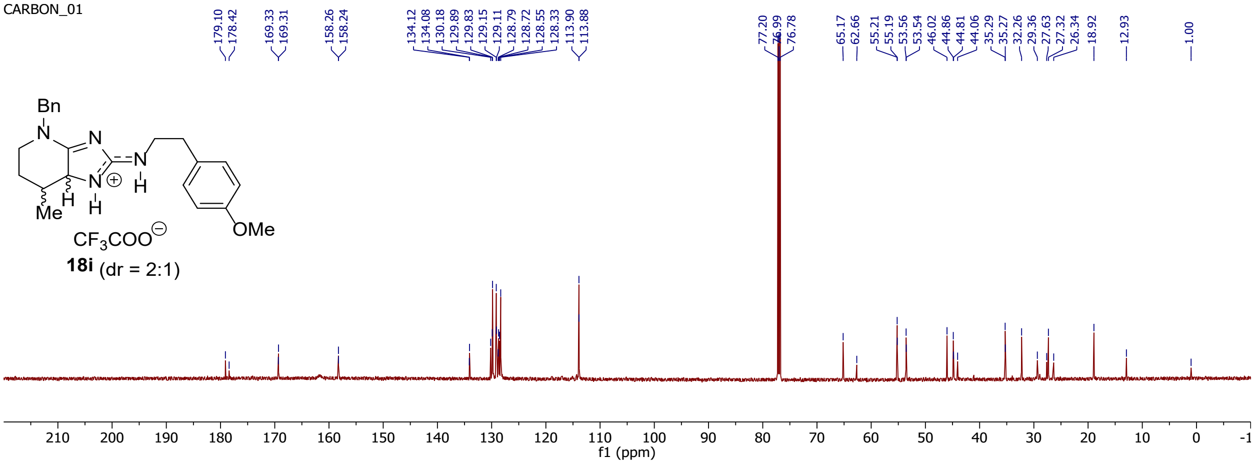
CARBON_01
179.10
178.42
169.33
169.31
158.26
158.24
134.12
134.08
130.18
129.89
129.83
129.15
129.11
128.79
128.72
128.55
128.33
113.90
113.88
77.20
76.99
76.78
65.17
62.66
55.21
55.19
53.56
53.54
46.02
44.86
44.81
44.06
35.29
35.27
32.26
29.36
27.63
27.32
26.34
18.92
12.93
1.00
Bn
N
N
N
N
H
H
H
Me
OMe
$CF_3COO^{\ominus}$
18i (dr = 2:1)
f1 (ppm)

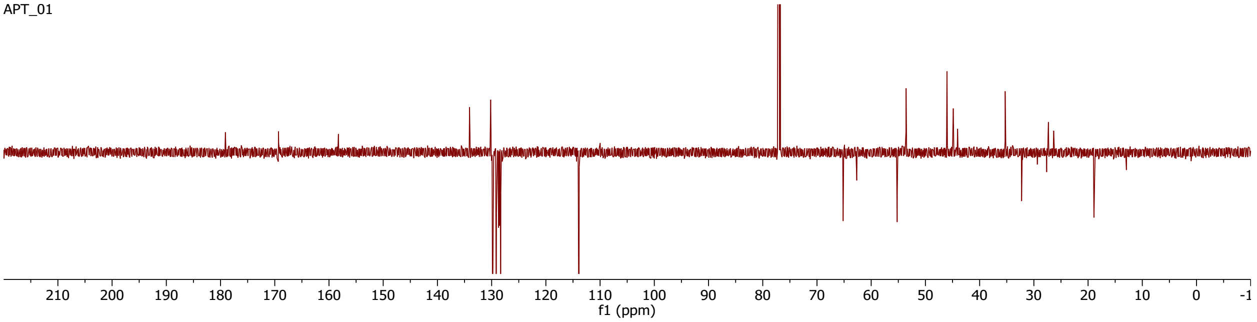
APT_01
f1 (ppm)

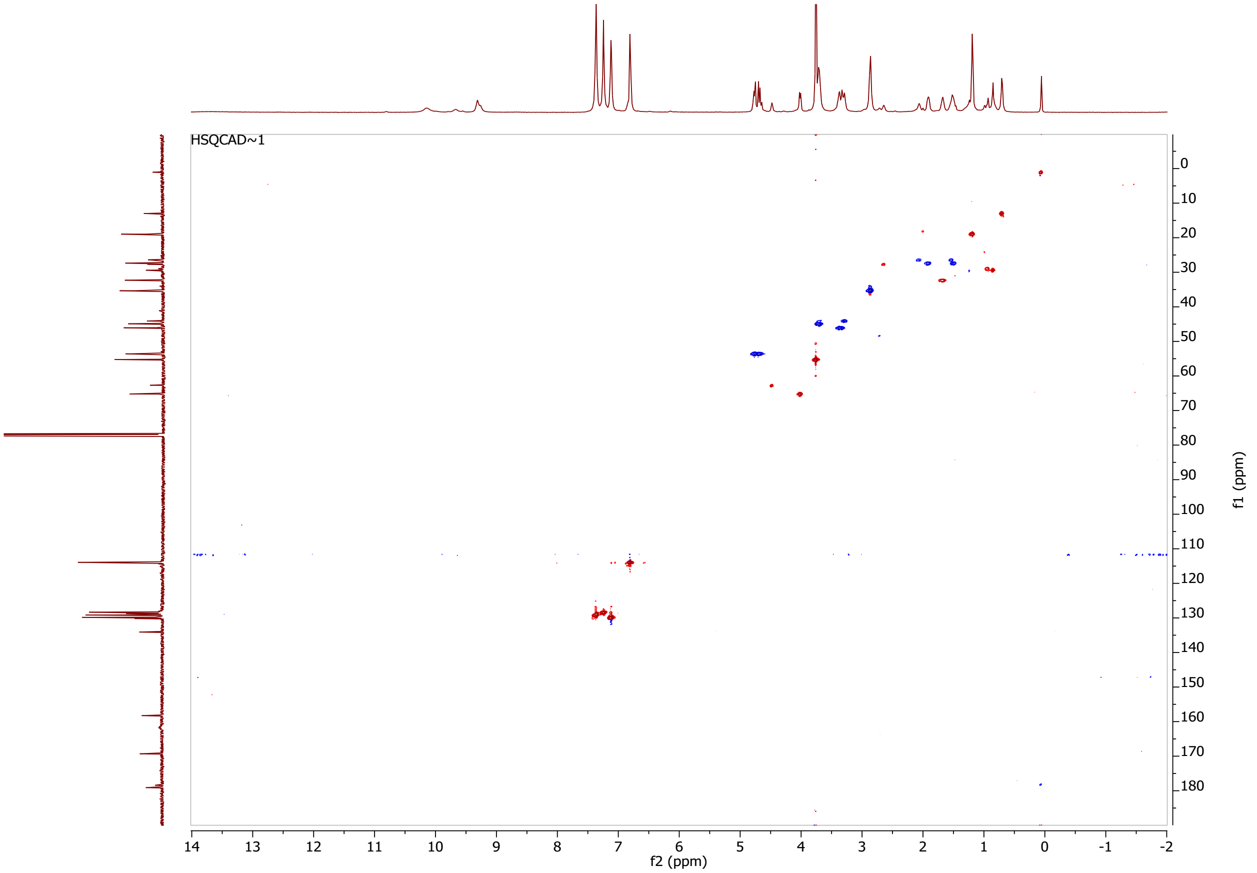
HSQCAD~1
f2 (ppm)
f1 (ppm)

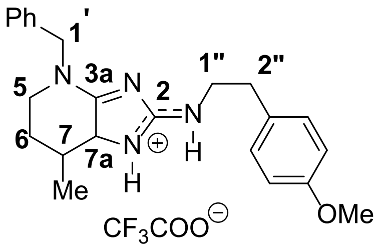
Ph
1'
5
N
3a
N
2
1"
2"
6
7
7a
N
H
Me
OMe
CF3COO

18i

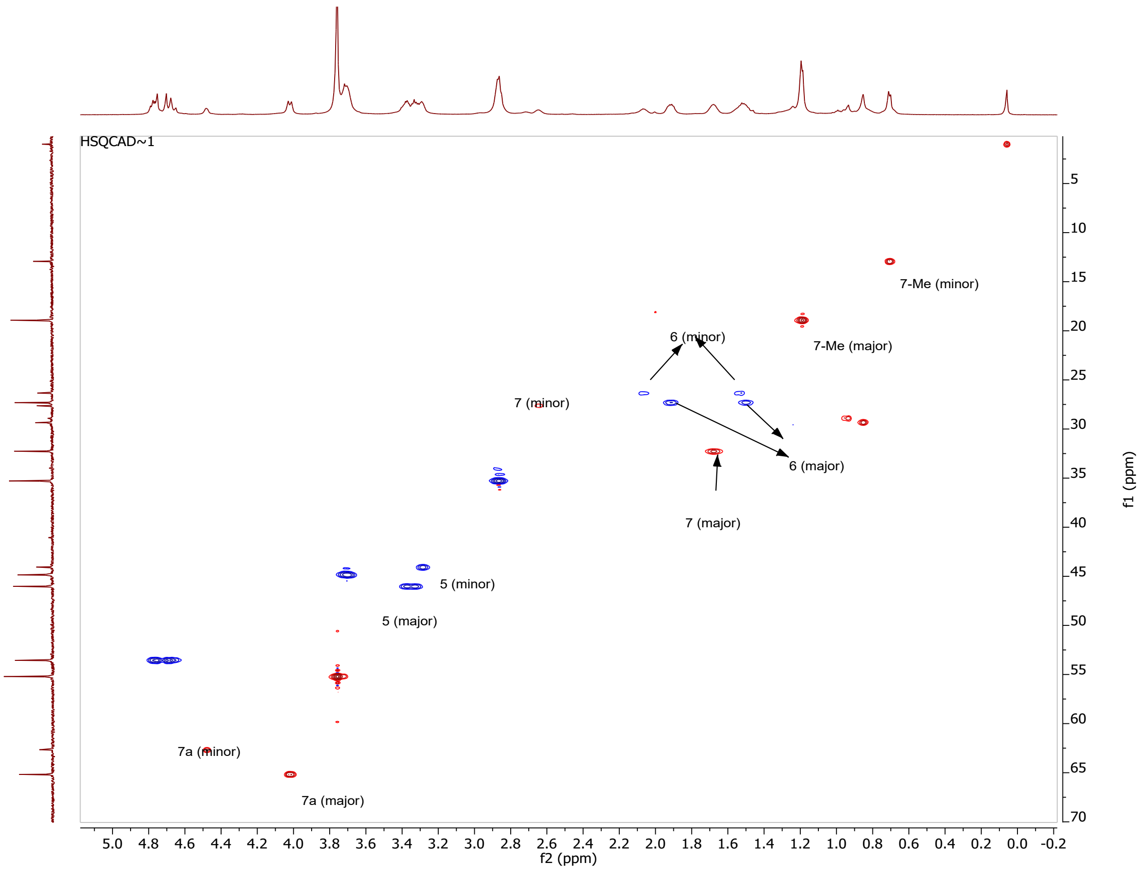
HSQCAD~1
7-Me (minor)
6 (minor)
7-Me (major)
7 (minor)
6 (major)
7 (major)
5 (minor)
5 (major)
7a (minor)
7a (major)
f2 (ppm)
f1 (ppm)

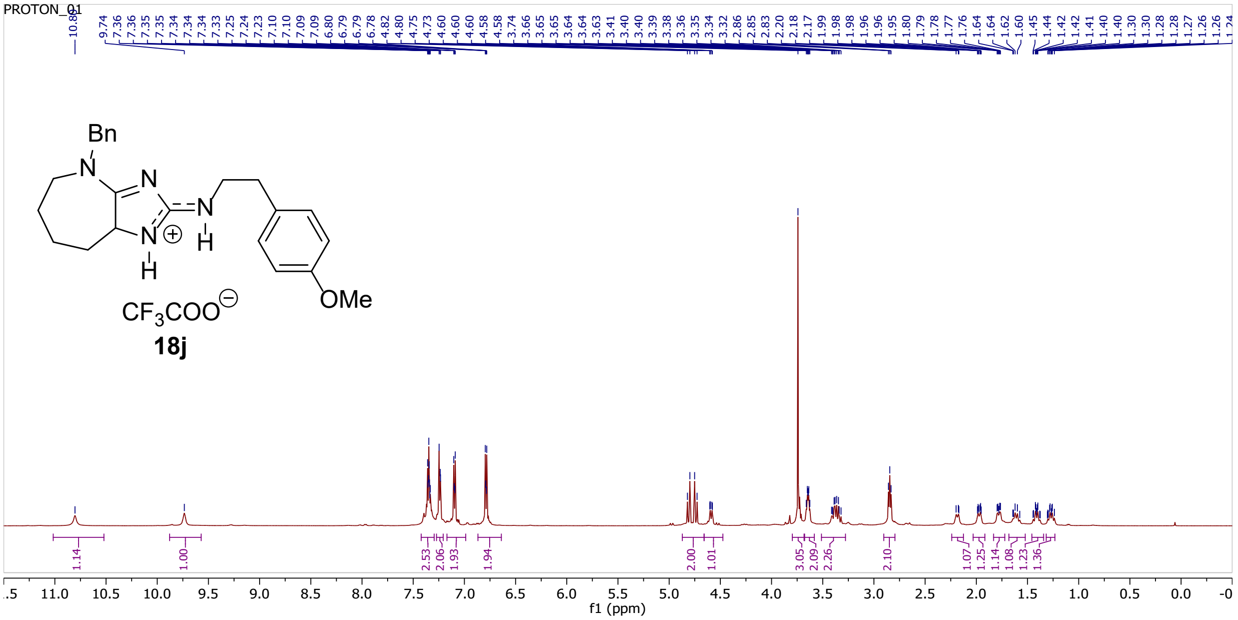
PROTON_01
10.80 9.74 7.36 7.36 7.35 7.35 7.34 7.34 7.34 7.33 7.25 7.24 7.23 7.10 7.10 7.09 7.09 6.80 6.79 6.79 6.78 4.82 4.80 4.75 4.73 4.60 4.60 4.60 4.58 4.58 3.74 3.66 3.65 3.65 3.64 3.64 3.63 3.41 3.40 3.40 3.39 3.38 3.36 3.35 3.34 3.32 2.86 2.85 2.83 2.20 2.18 2.17 1.99 1.98 1.98 1.96 1.96 1.95 1.80 1.79 1.78 1.77 1.76 1.64 1.64 1.62 1.60 1.45 1.44 1.42 1.42 1.41 1.40 1.40 1.30 1.30 1.28 1.28 1.27 1.26 1.24
Bn
N
N
N
N
H
H
OMe
$CF_3COO^{\ominus}$
18j
1.14 1.00 2.53 2.06 1.93 1.94 2.00 1.01 3.05 2.09 2.26 2.10 1.07 1.25 1.14 1.08 1.23 1.36
f1 (ppm)

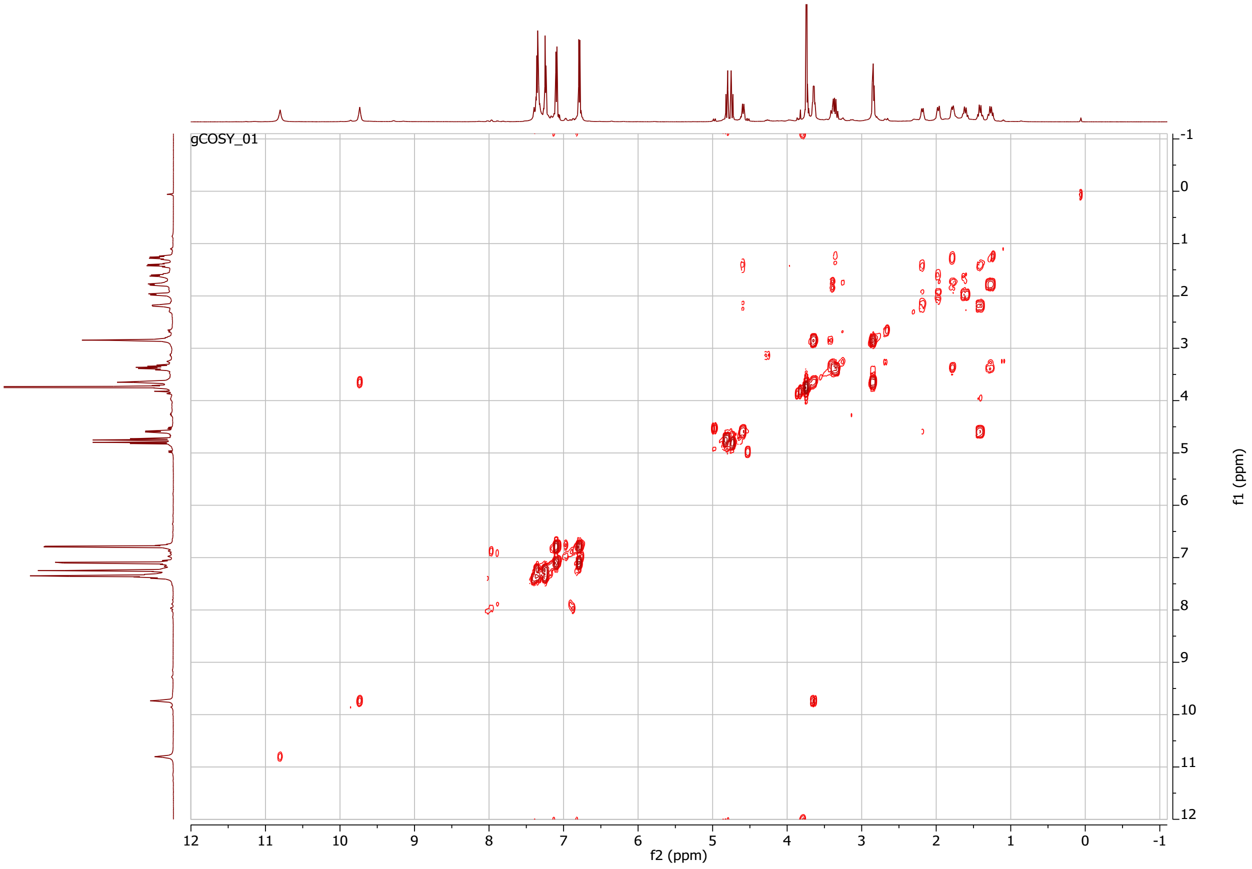
gCOSY_01
f2 (ppm)
f1 (ppm)

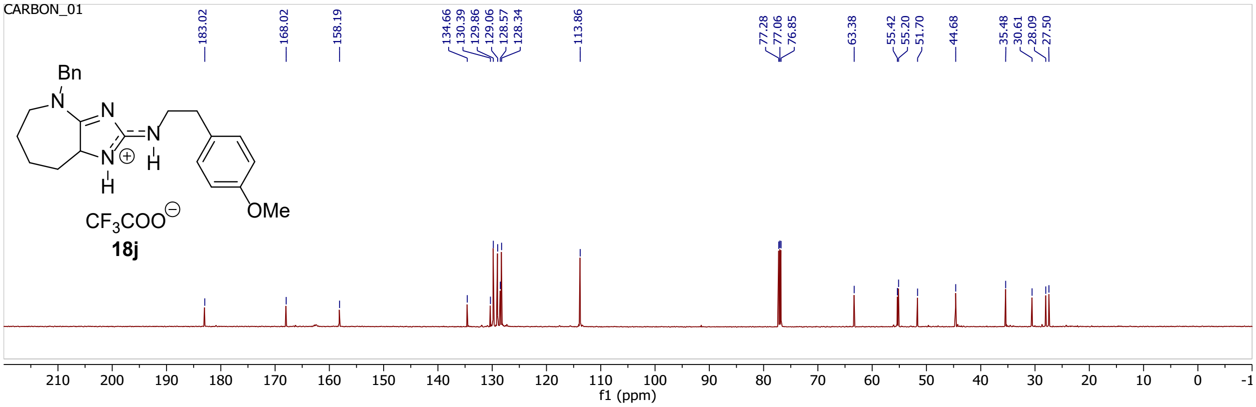
CARBON_01
183.02
168.02
158.19
134.66
130.39
129.86
129.06
128.57
128.34
113.86
77.28
77.06
76.85
63.38
55.42
55.20
51.70
44.68
35.48
30.61
28.09
27.50
Bn
N
N
N
N
H
H
CF3COO
OMe
18j
f1 (ppm)

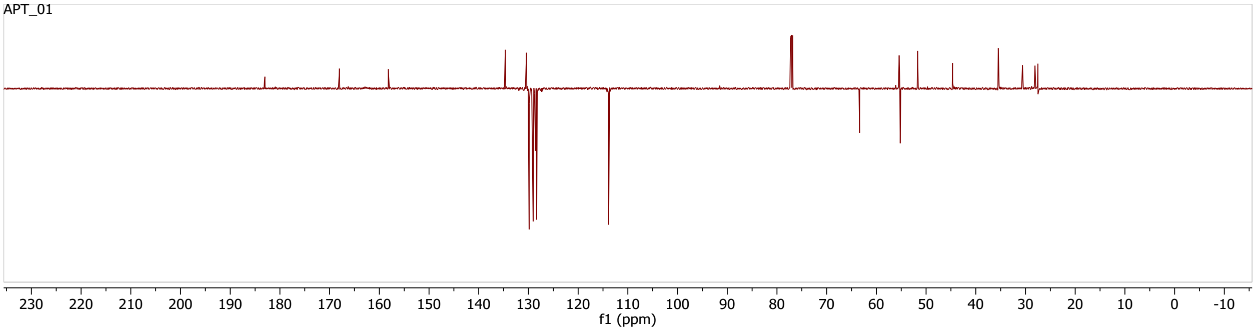
APT_01
f1 (ppm)

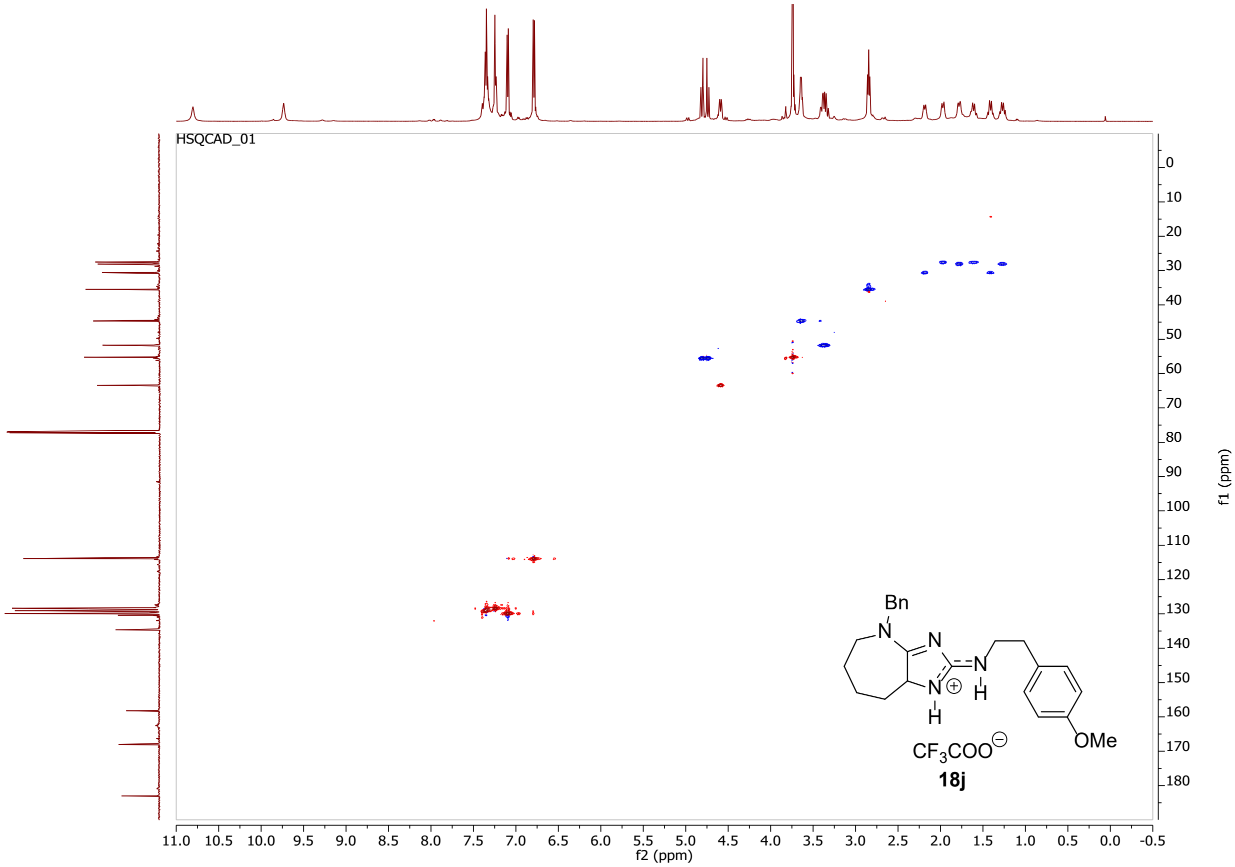
HSQCAD_01
Bn
N
N
N
N
H
H
CF3COO
OMe
18j
f2 (ppm)
f1 (ppm)

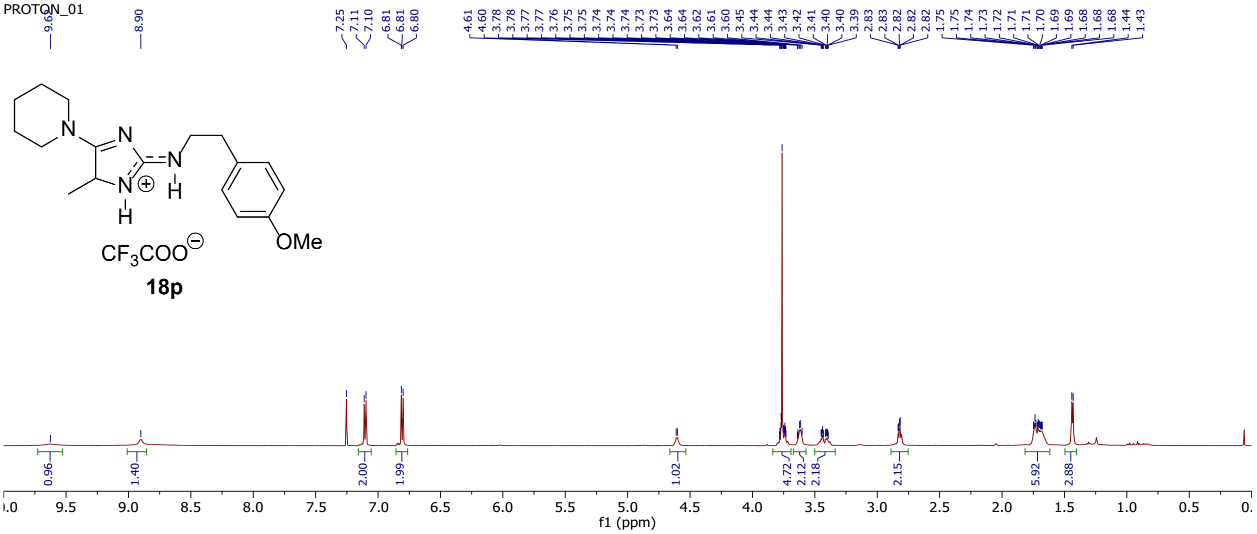
PROTON_01
18p
f1 (ppm)

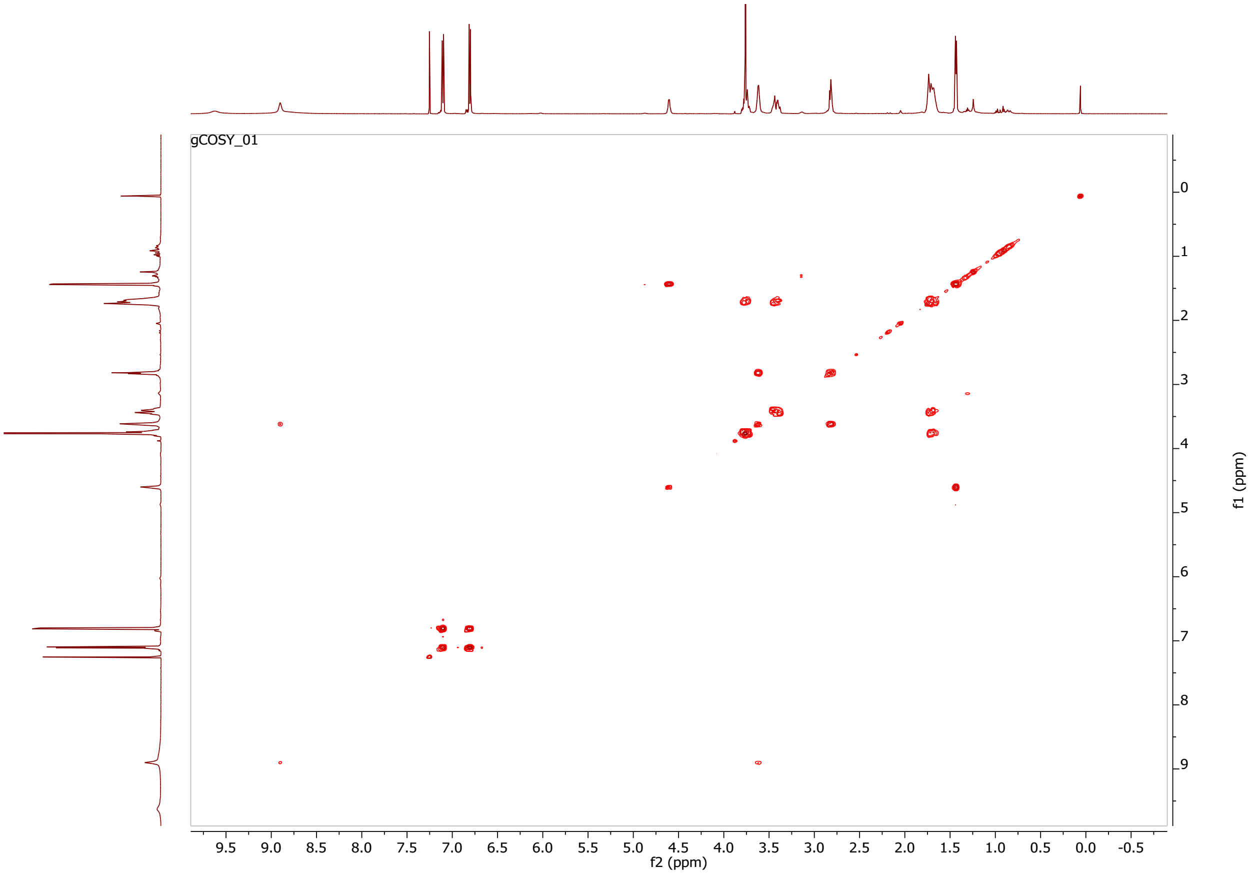
gCOSY_01
f2 (ppm)
f1 (ppm)

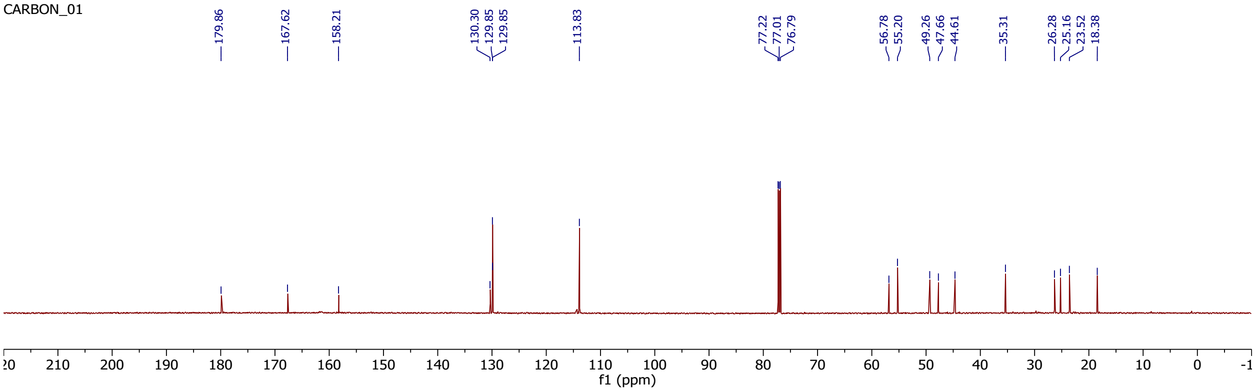
CARBON_01
179.86
167.62
158.21
130.30
129.85
129.85
113.83
77.22
77.01
76.79
56.78
55.20
49.26
47.66
44.61
35.31
26.28
25.16
23.52
18.38
f1 (ppm)

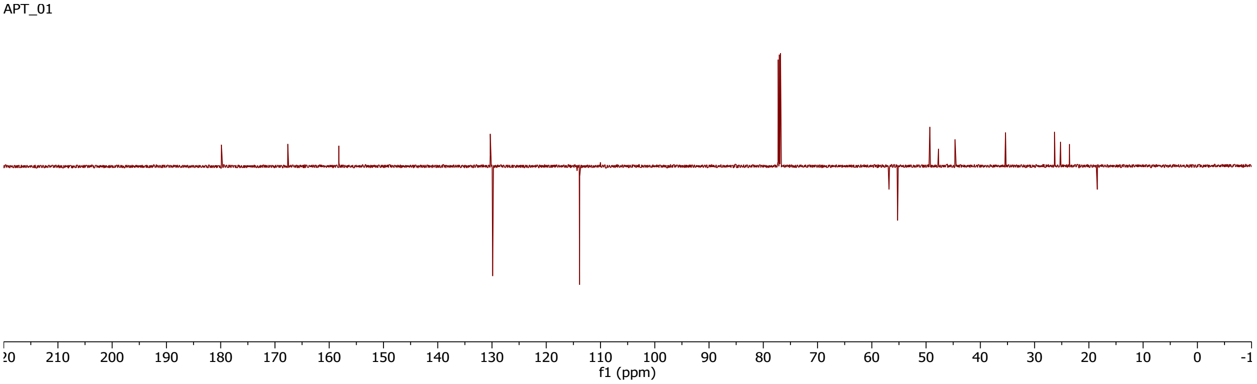
APT_01
f1 (ppm)

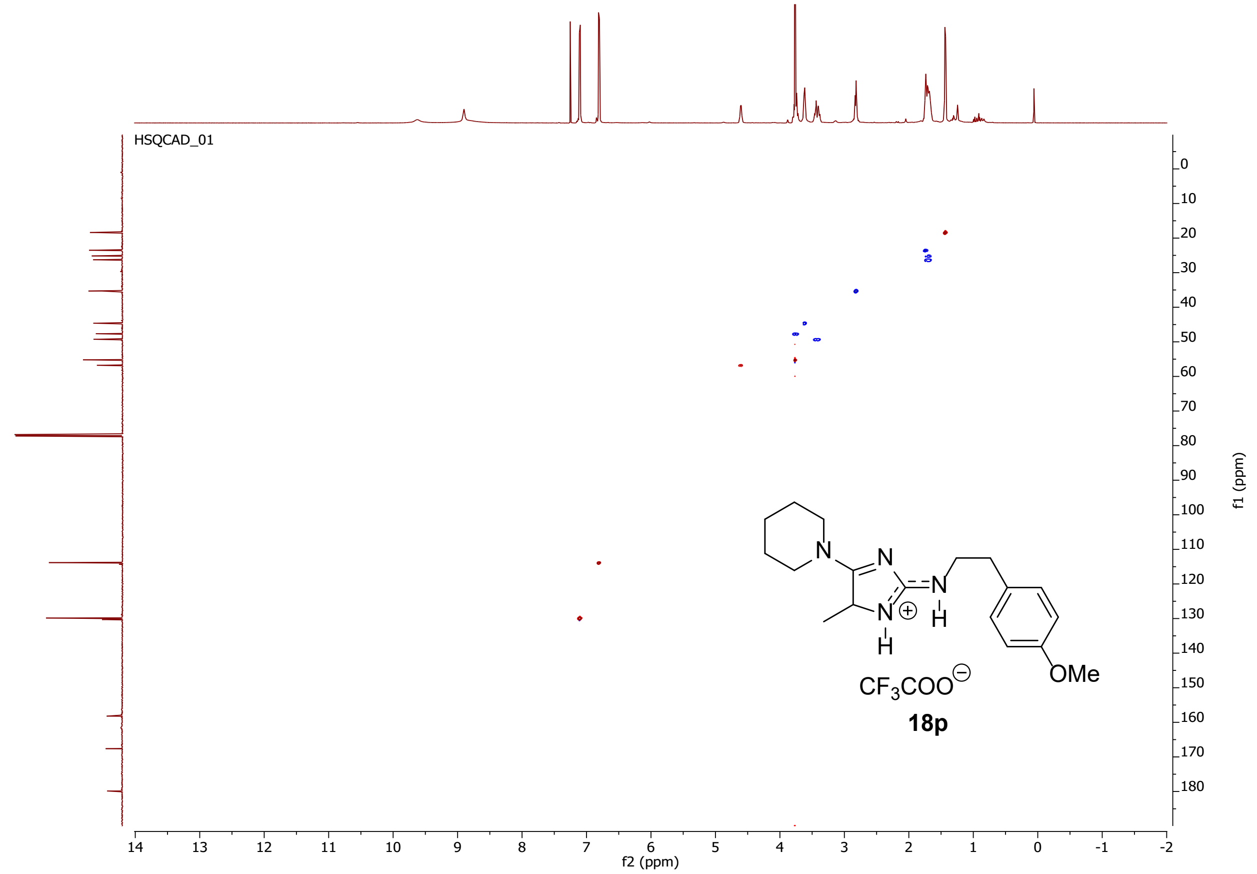
HSQCAD_01
f2 (ppm)
f1 (ppm)
CF3COO
OMe
18p

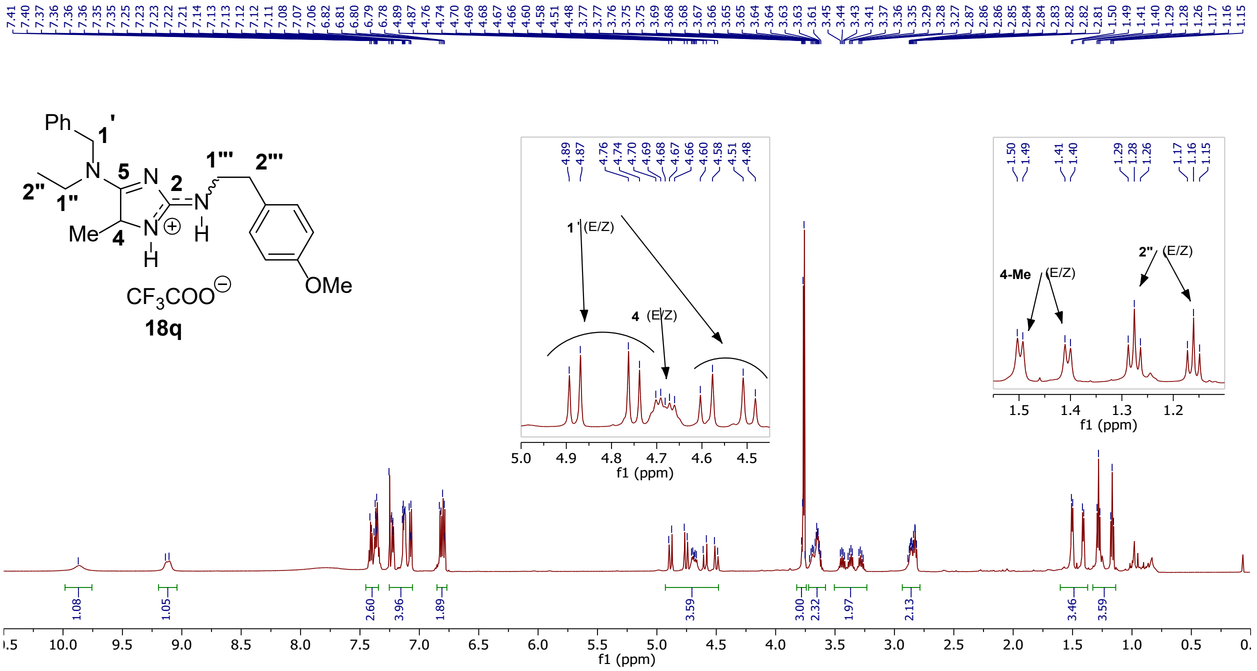
Ph
1'
5
2
1'''
2'''
2''
1''
Me
4
H
CF3COO
OMe
18q
1' (E/Z)
4 (E/Z)
4-Me
(E/Z)
2'' (E/Z)
f1 (ppm)

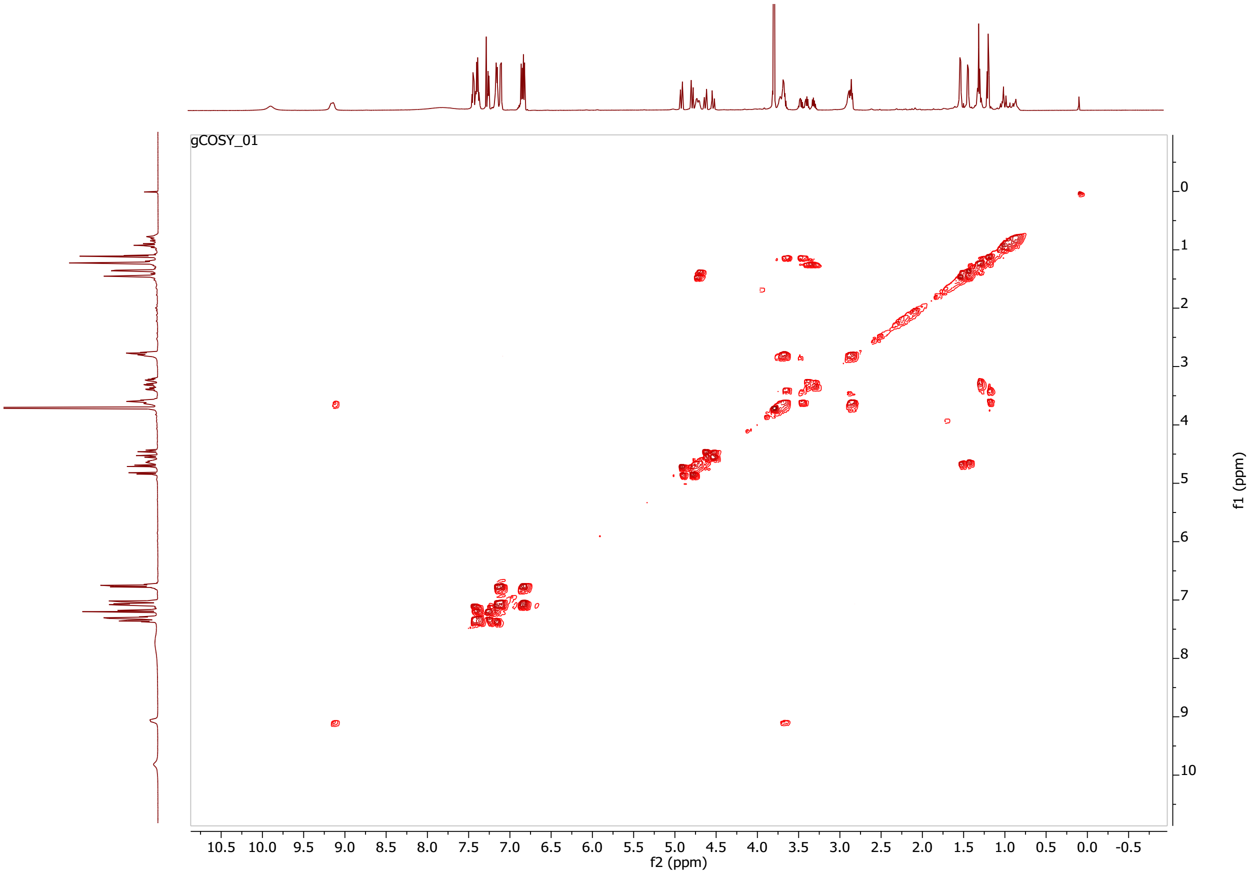
gCOSY_01
f2 (ppm)
f1 (ppm)

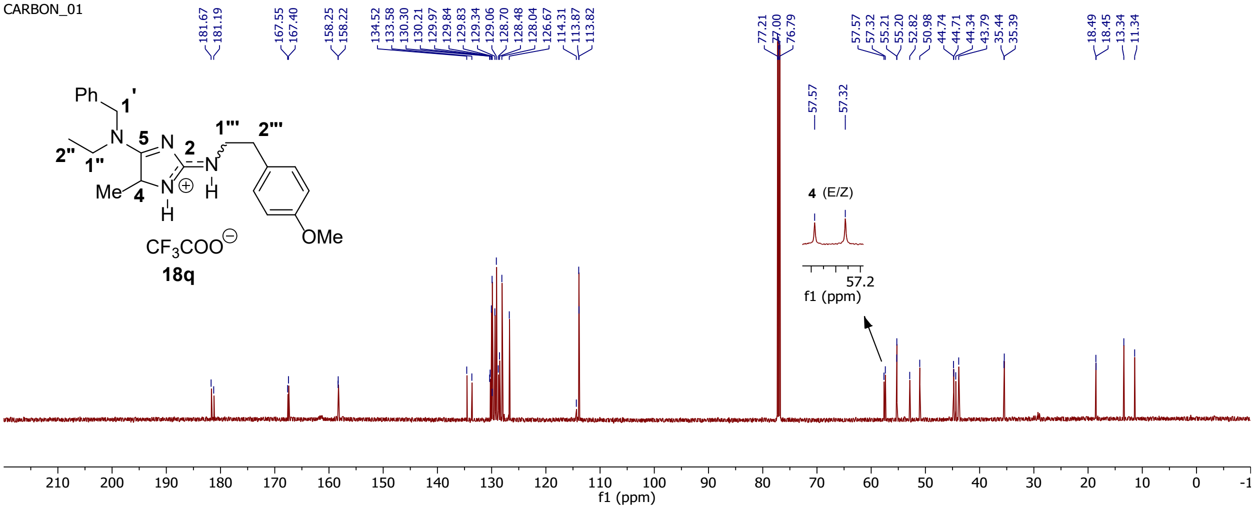
CARBON_01
181.67
181.19
167.55
167.40
158.25
158.22
134.52
133.58
130.30
130.21
129.97
129.84
129.83
129.34
129.06
128.70
128.48
128.04
126.67
114.31
113.87
113.82
77.21
77.00
76.79
57.57
57.32
55.21
55.20
52.82
50.98
44.74
44.71
44.34
43.79
35.44
35.39
18.49
18.45
13.34
11.34
Ph
1'
N
5
N
2
1'''
2'''
2''
1''
N
H
Me
4
N
H
OMe
$CF_3COO^{\ominus}$
18q
57.57
57.32
4 (E/Z)
57.2
f1 (ppm)
210 200 190 180 170 160 150 140 130 120 110 100 90 80 70 60 50 40 30 20 10 0 -1
f1 (ppm)

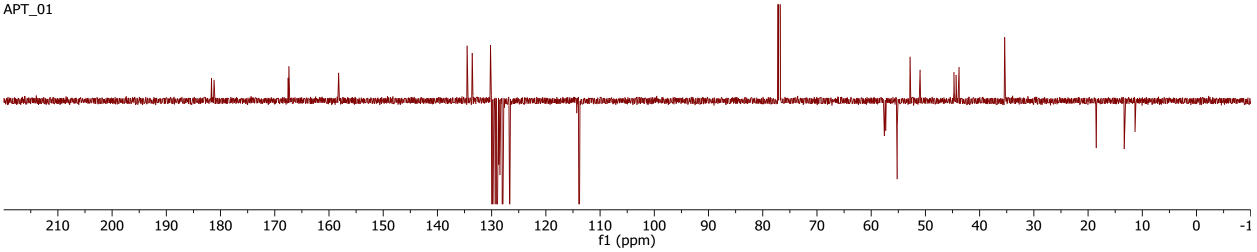
APT_01
210 200 190 180 170 160 150 140 130 120 110 100 90 80 70 60 50 40 30 20 10 0 -1
f1 (ppm)

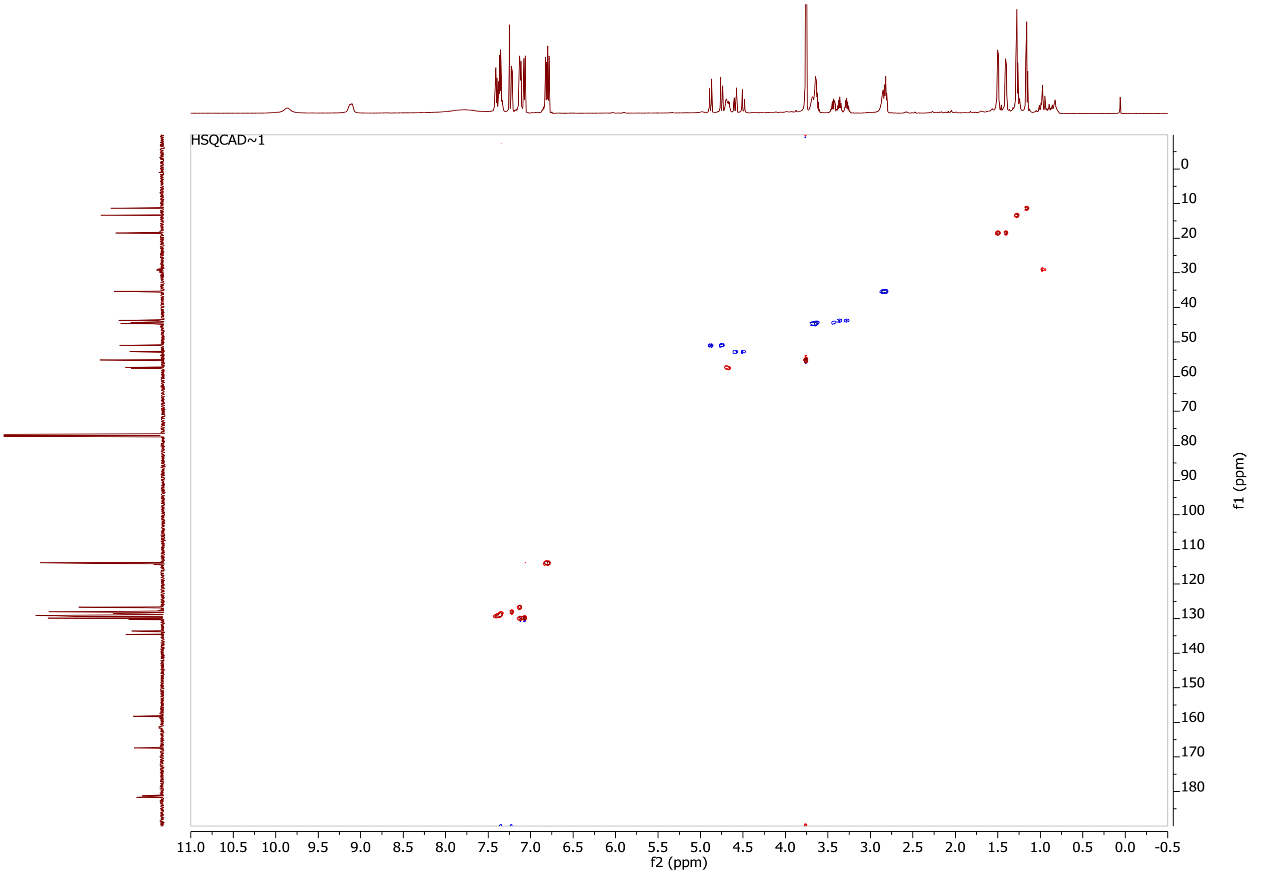
HSQCAD~1
0 10 20 30 40 50 60 70 80 90 100 110 120 130 140 150 160 170 180
f1 (ppm)
11.0 10.5 10.0 9.5 9.0 8.5 8.0 7.5 7.0 6.5 6.0 5.5 5.0 4.5 4.0 3.5 3.0 2.5 2.0 1.5 1.0 0.5 0.0 -0.5
f2 (ppm)

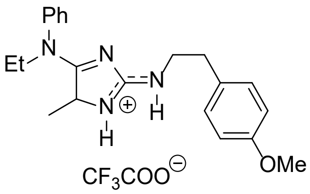

18r

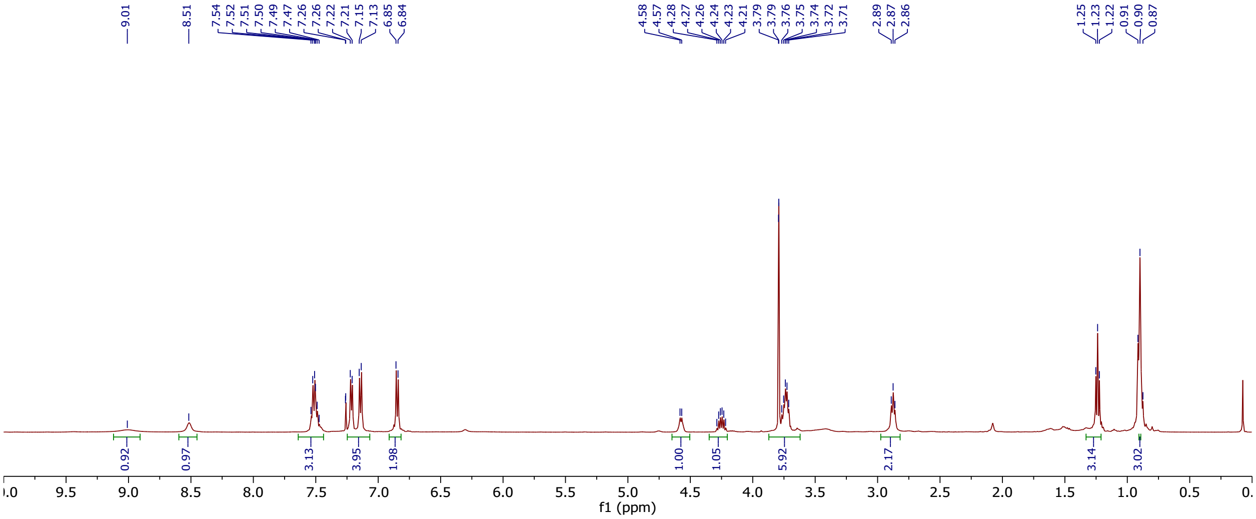

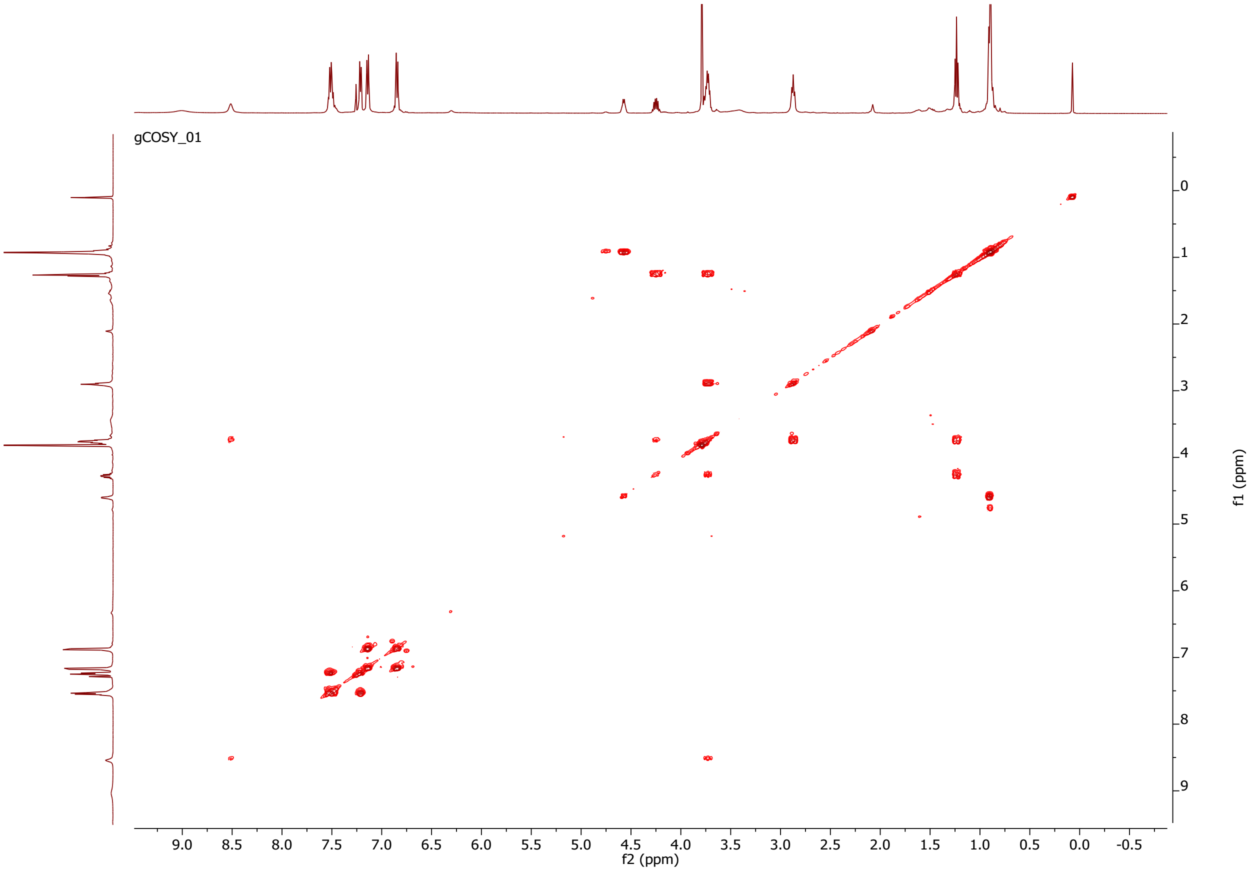

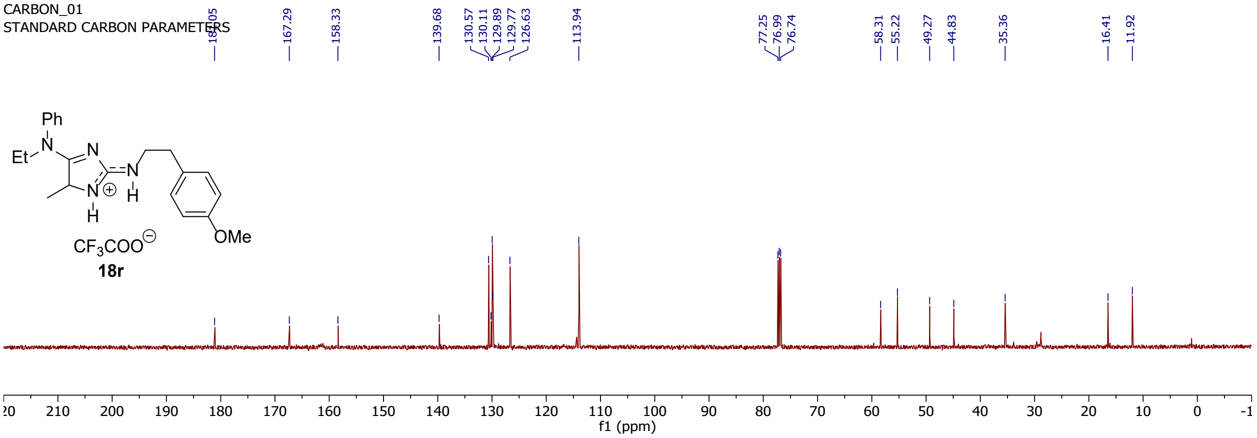
CARBON_01
STANDARD CARBON PARAMETERS
181.05
167.29
158.33
139.68
130.57
130.11
129.89
129.77
126.63
113.94
77.25
76.99
76.74
58.31
55.22
49.27
44.83
35.36
16.41
11.92
Ph
N
Et
N
N
N
H
H
OMe
$CF_3COO^{\ominus}$
18r
f1 (ppm)

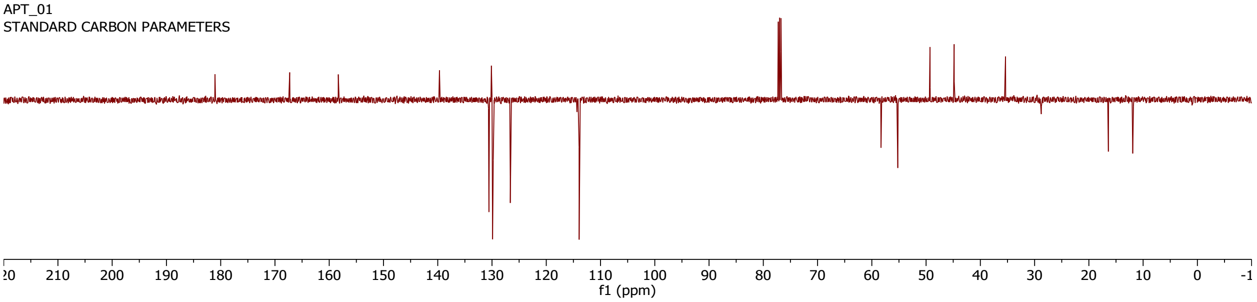
APT_01
STANDARD CARBON PARAMETERS
f1 (ppm)

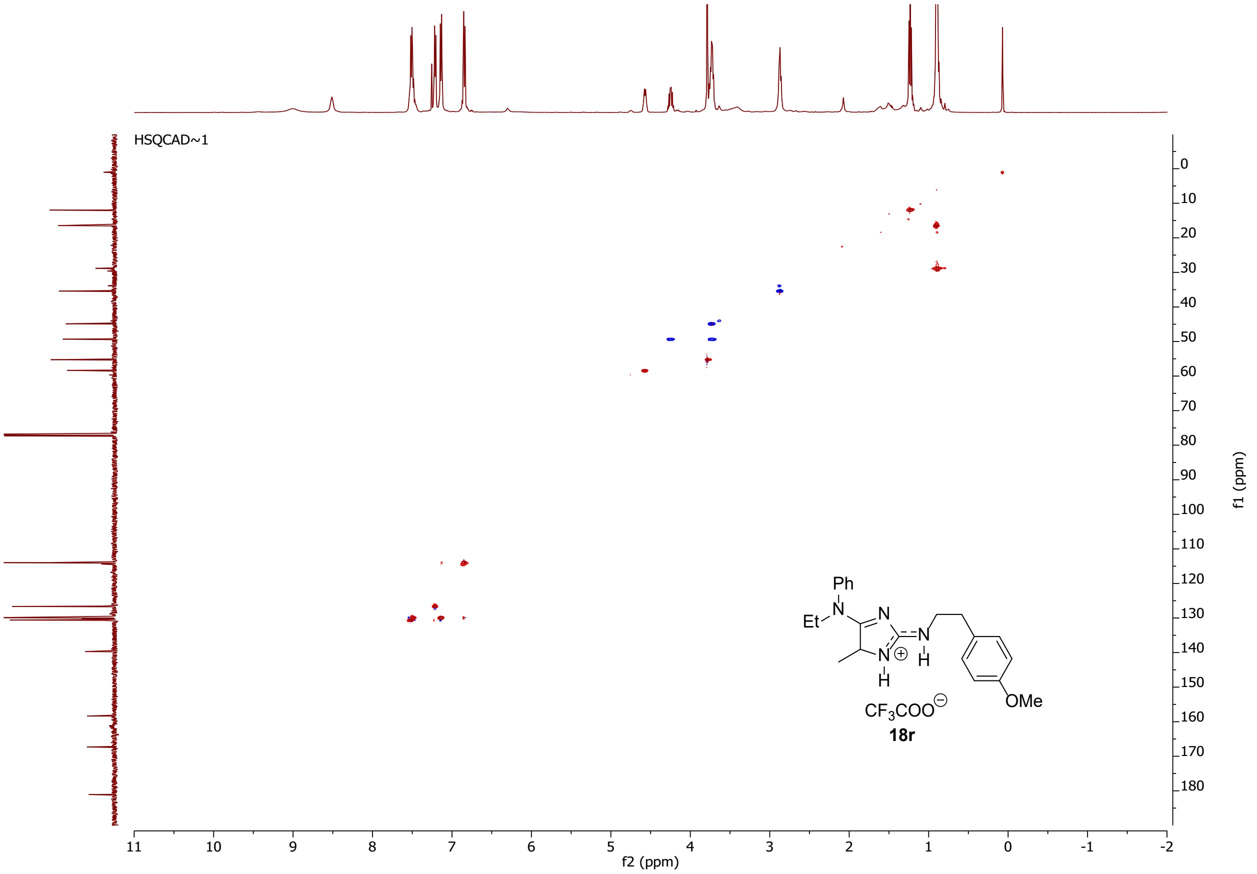
HSQCAD~1
Ph
N
Et
N
N
N
H
H
OMe
$CF_3COO^{\ominus}$
18r
f2 (ppm)
f1 (ppm)

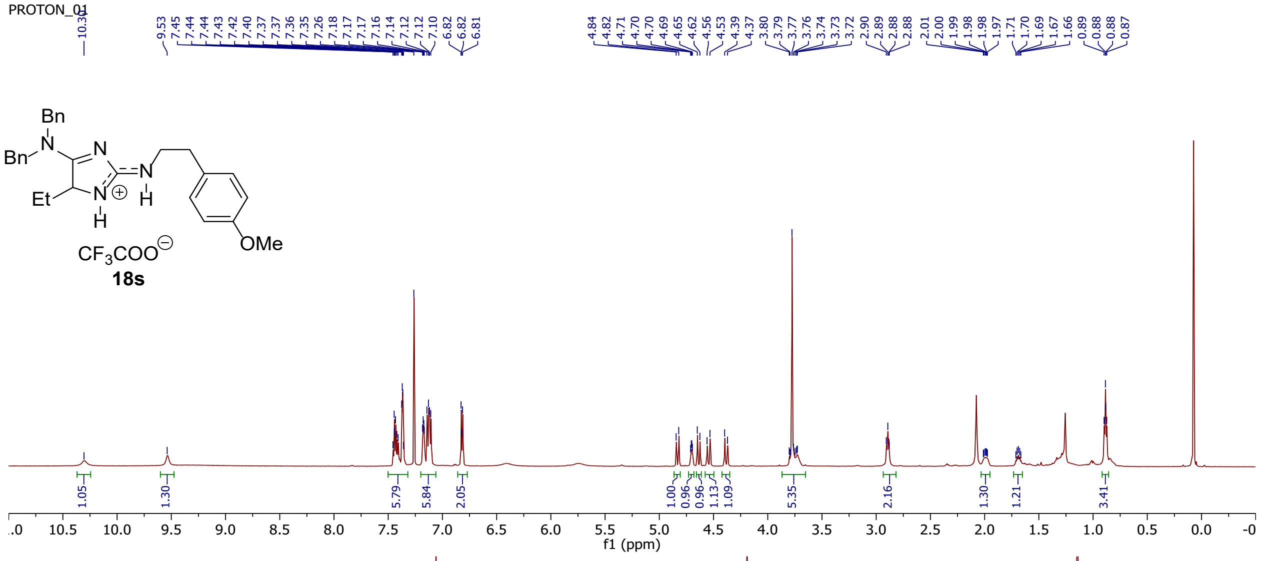
PROTON_01
10.30
9.53 7.45 7.44 7.44 7.43 7.42 7.40 7.37 7.36 7.35 7.26 7.18 7.17 7.17 7.16 7.14 7.12 7.12 7.10 6.82 6.81
4.84 4.82 4.71 4.70 4.70 4.69 4.65 4.62 4.56 4.53 4.39 4.37 3.80 3.79 3.77 3.76 3.74 3.73 3.72 2.90 2.89 2.88 2.88 2.01 2.00 1.99 1.98 1.98 1.97 1.71 1.70 1.69 1.67 1.66 0.89 0.88 0.88 0.87
Bn
Bn
N
N
N
N
H
H
Et
OMe
CF3COO
18s
1.05 1.30 5.79 5.84 2.05 1.00 0.96 0.96 1.13 1.09 5.35 2.16 1.30 1.21 3.41
f1 (ppm)

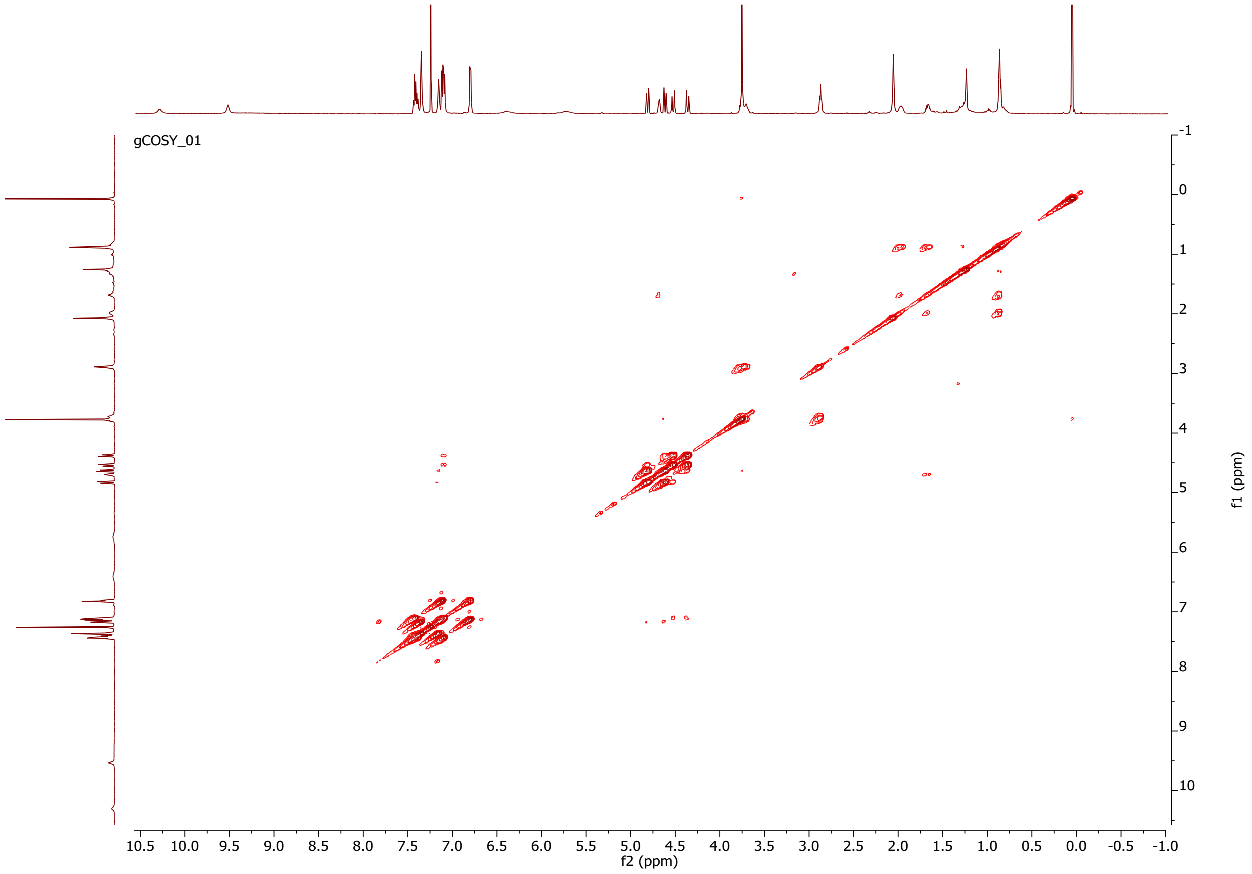
gCOSY_01
f2 (ppm)
f1 (ppm)

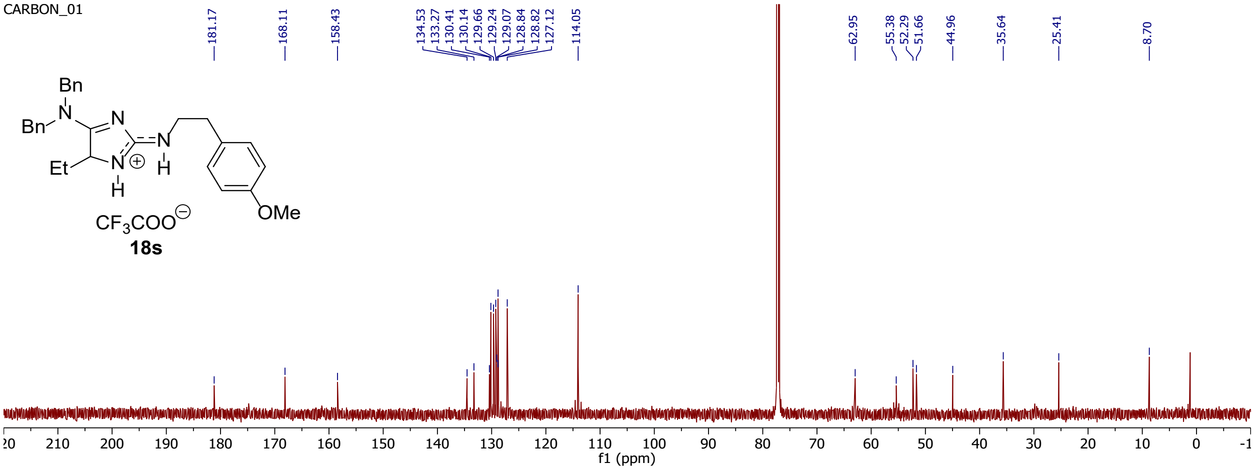
CARBON_01
181.17
168.11
158.43
134.53
133.27
130.41
130.14
129.66
129.24
129.07
128.84
128.82
127.12
114.05
62.95
55.38
52.29
51.66
44.96
35.64
25.41
8.70
Bn
Bn
N
N
N
H
Et
N
H
CF3COO
OMe
18s
f1 (ppm)

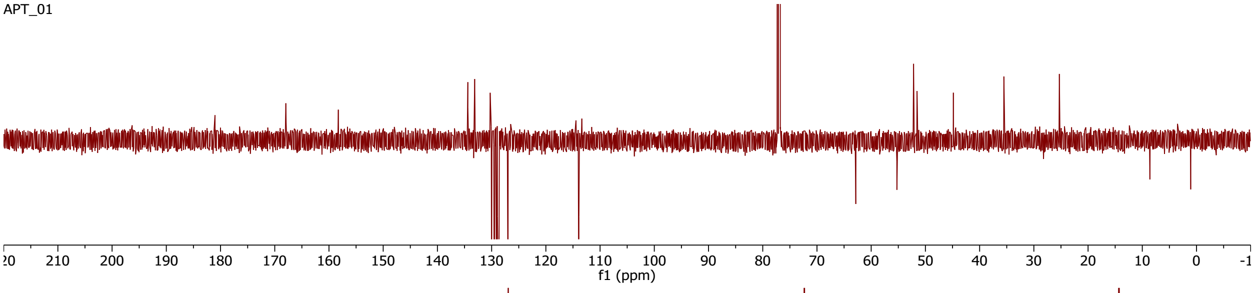
APT_01
f1 (ppm)

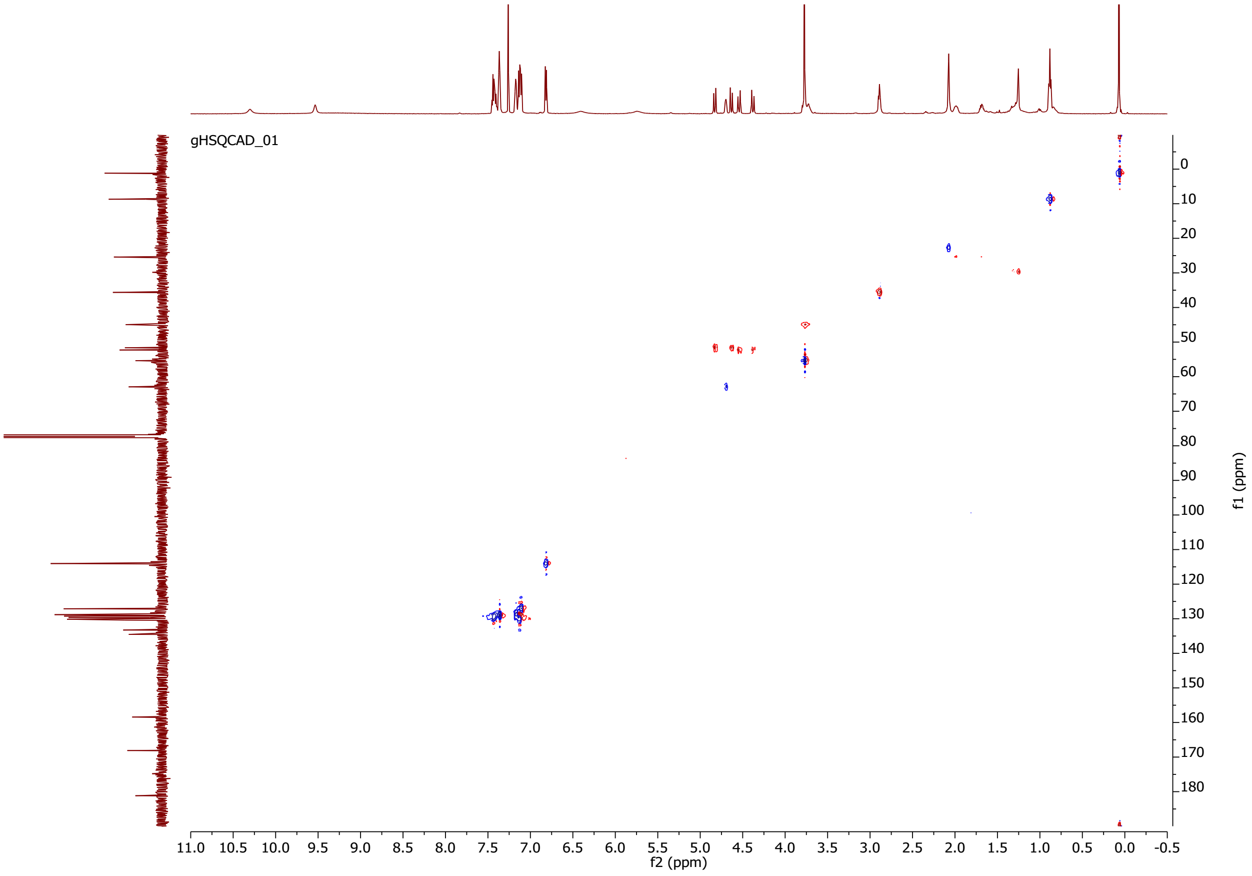
gHSQCAD_01
f1 (ppm)
f2 (ppm)

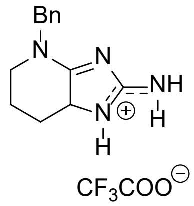

18t

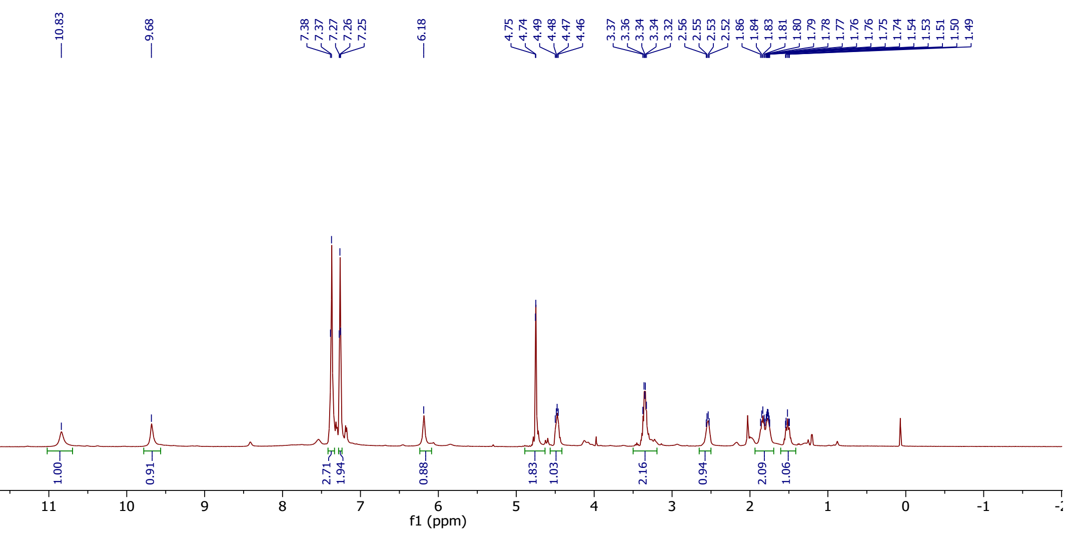

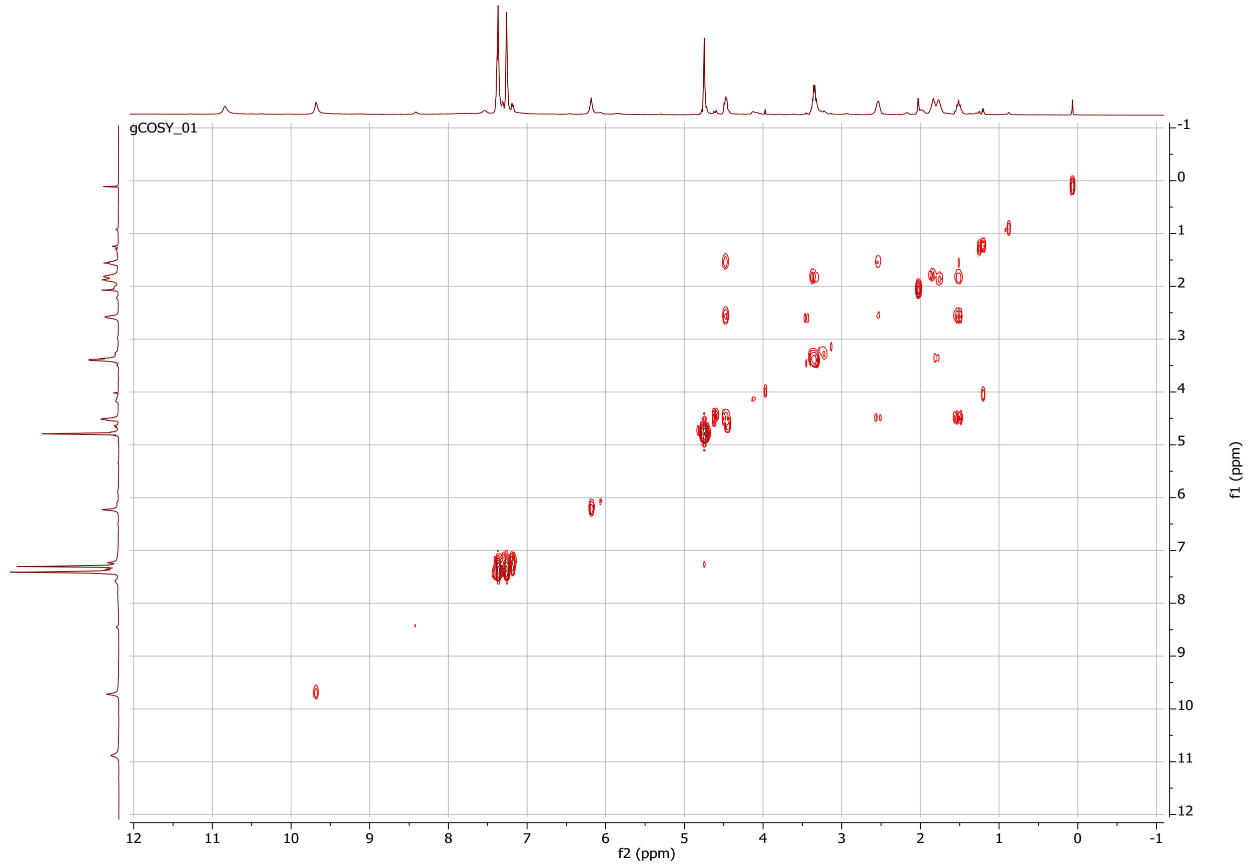

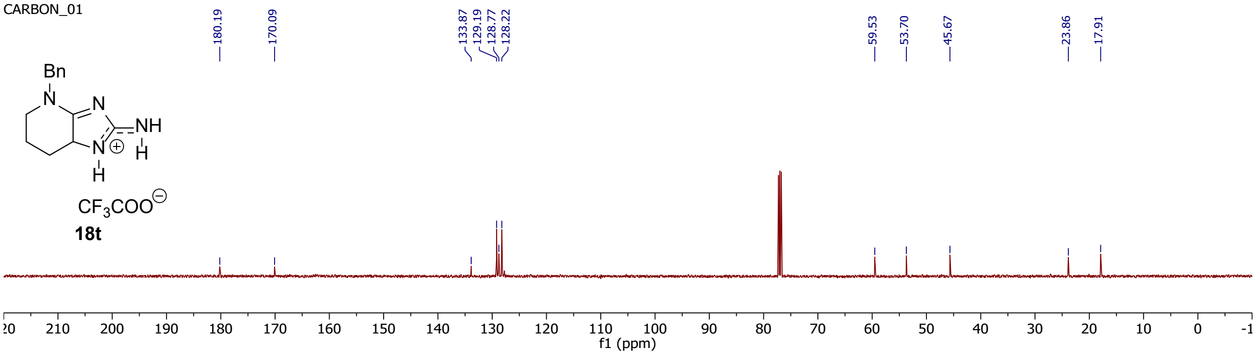
CARBON_01
180.19
170.09
133.87
129.19
128.77
128.22
59.53
53.70
45.67
23.86
17.91
Bn
N
N
NH
N
H
H
CF3COO
18t
f1 (ppm)

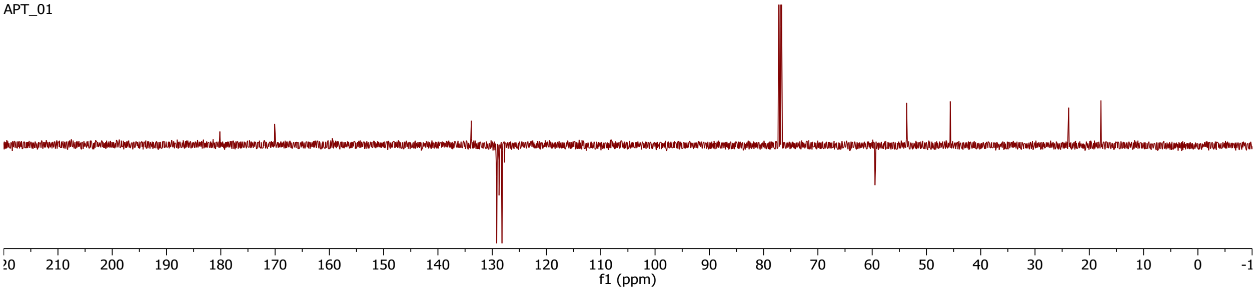
APT_01
f1 (ppm)

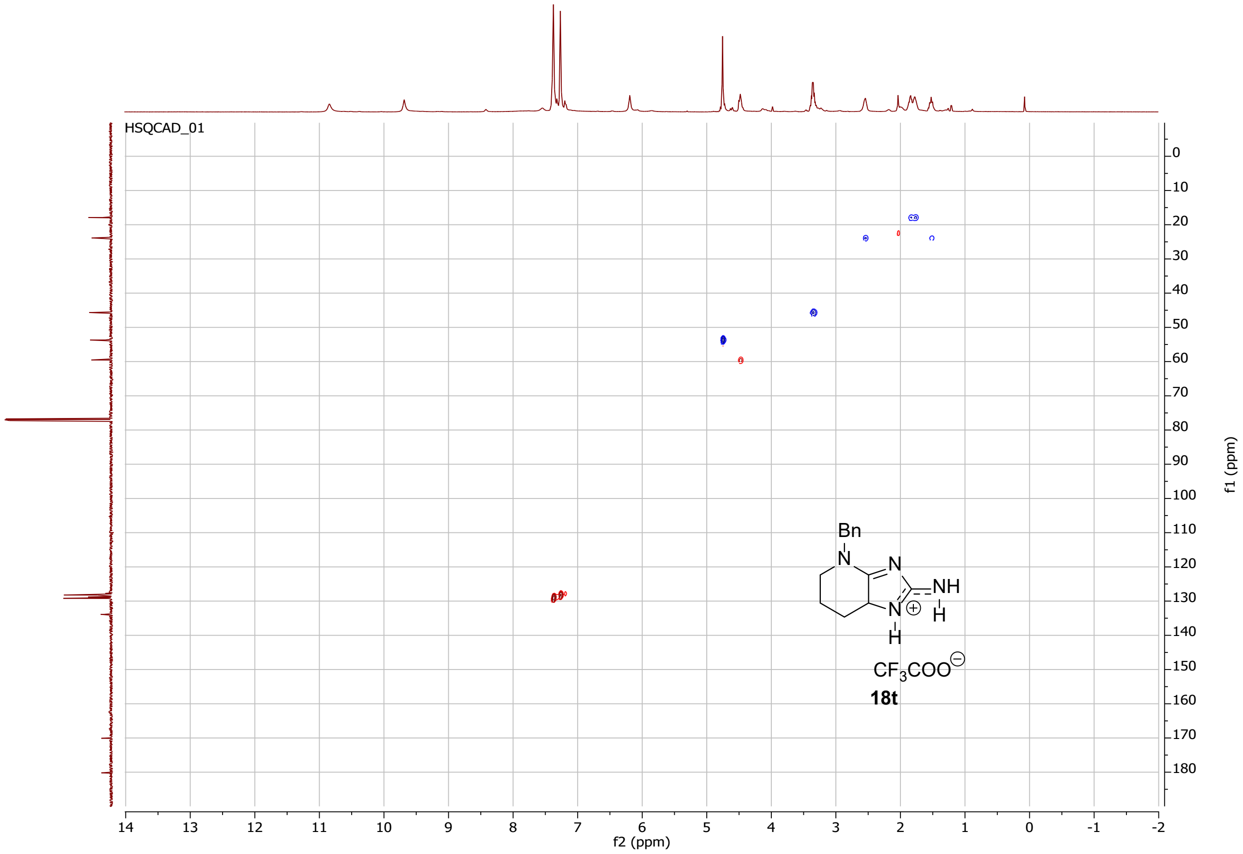
HSQCAD_01
Bn
N
N
NH
N
H
H
CF3COO
18t
f2 (ppm)
f1 (ppm)

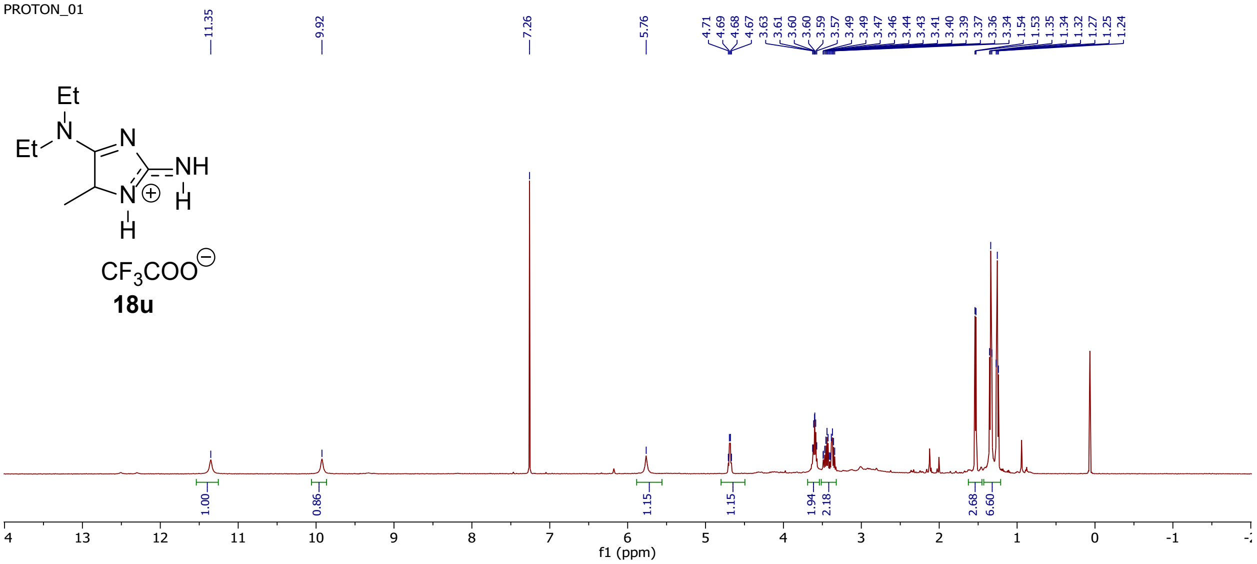
PROTON_01
18u
f1 (ppm)

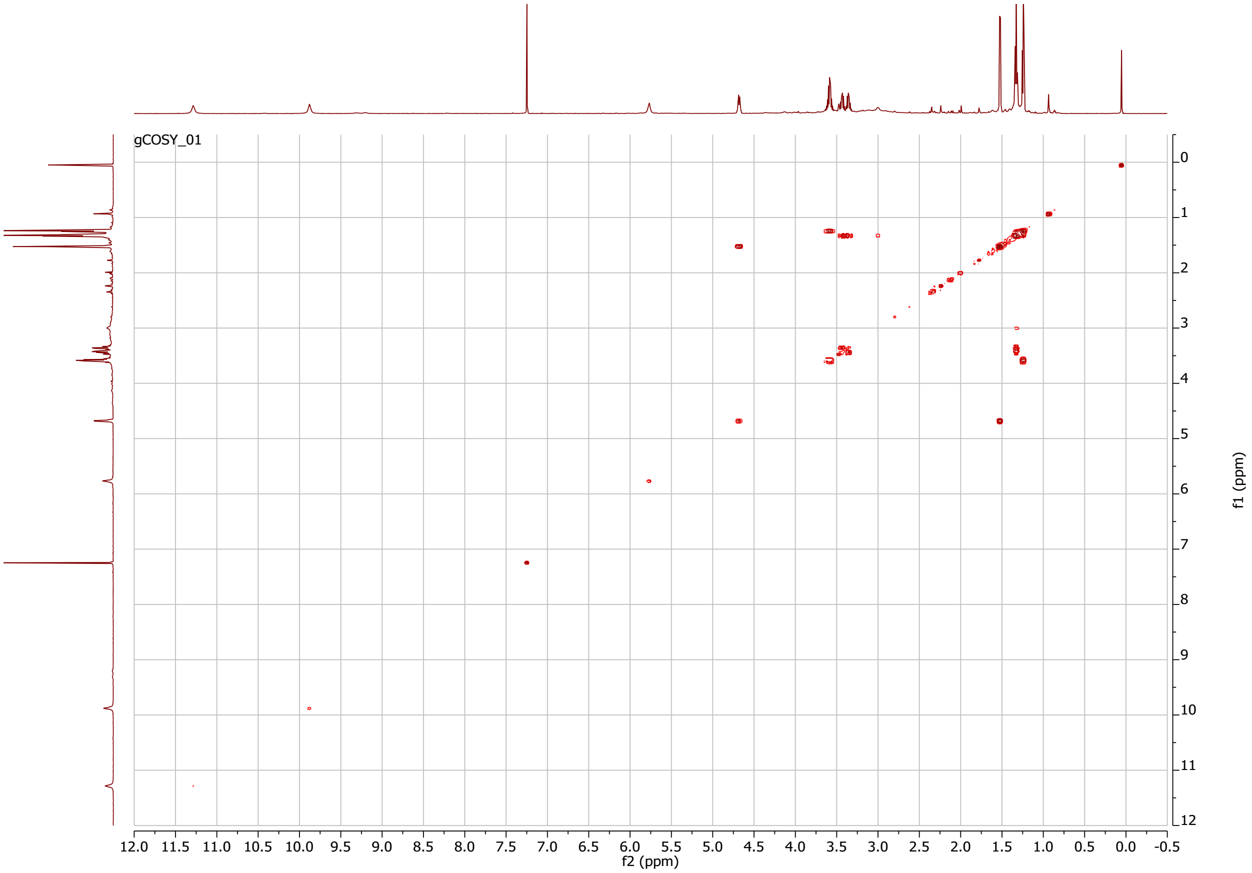
gCOSY_01
f2 (ppm)
f1 (ppm)

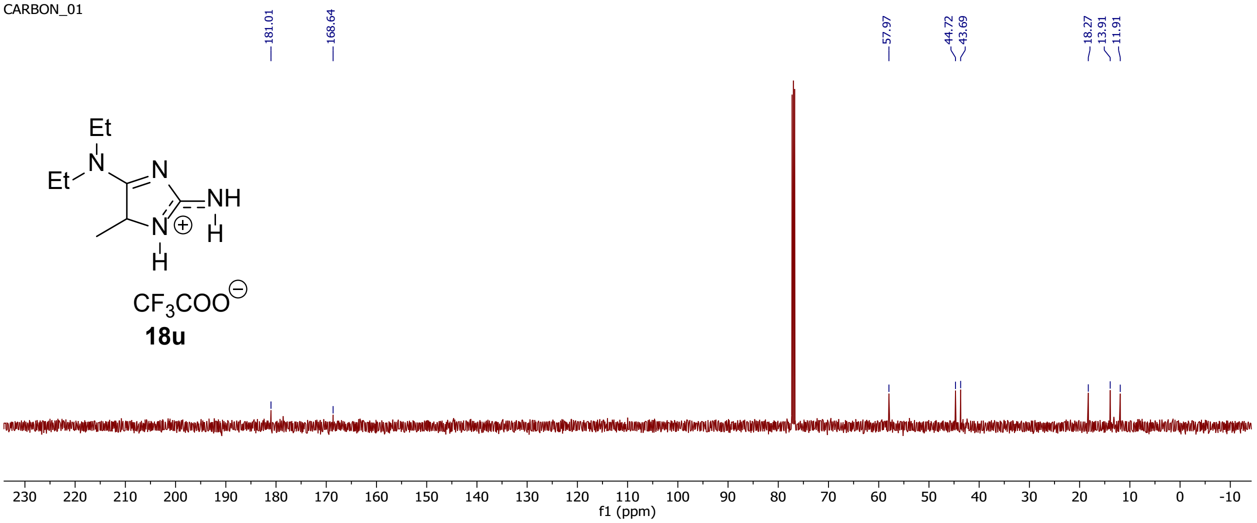
CARBON_01
181.01
168.64
57.97
44.72
43.69
18.27
13.91
11.91
Et
N
Et
N
NH
N
H
H
CF3COO
18u
f1 (ppm)

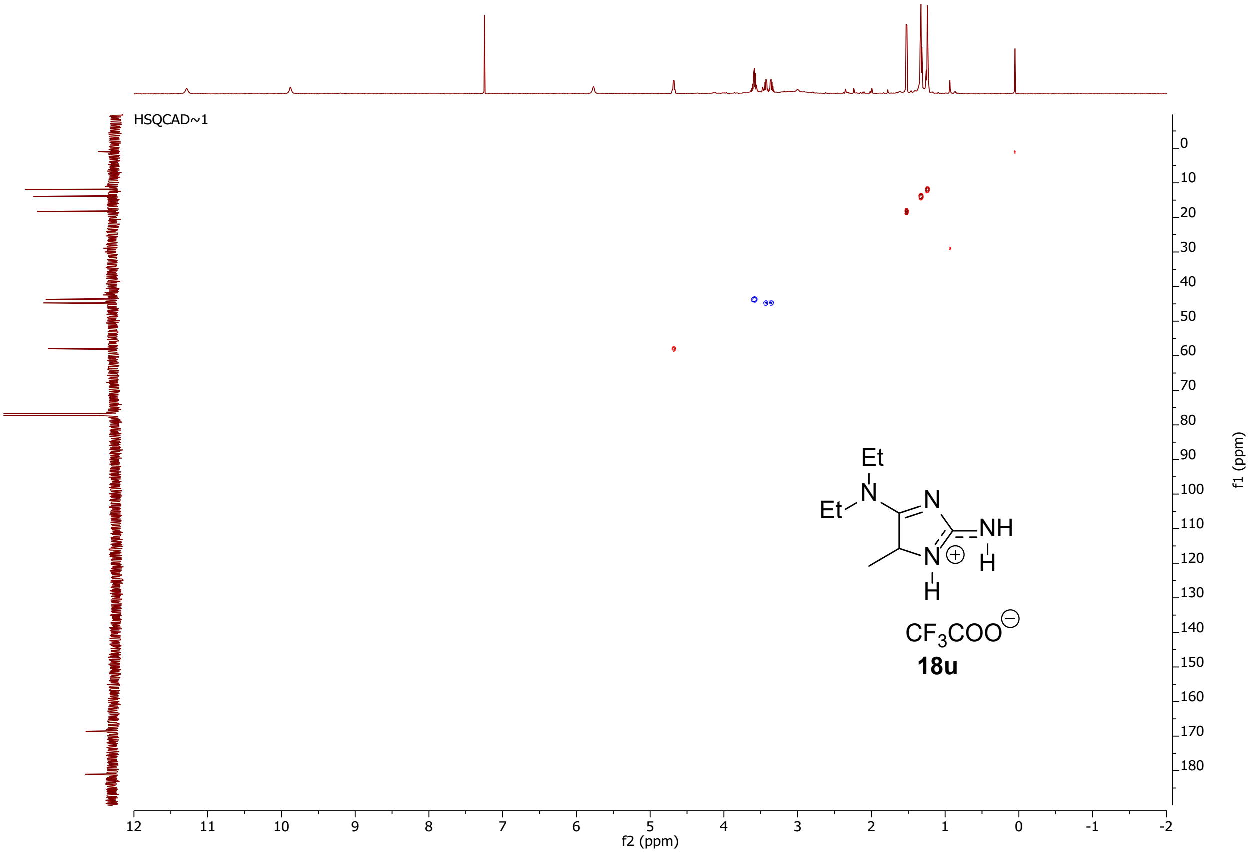
HSQCAD~1
Et
N
Et
N
NH
N
H
H
CF3COO
18u
f2 (ppm)
f1 (ppm)

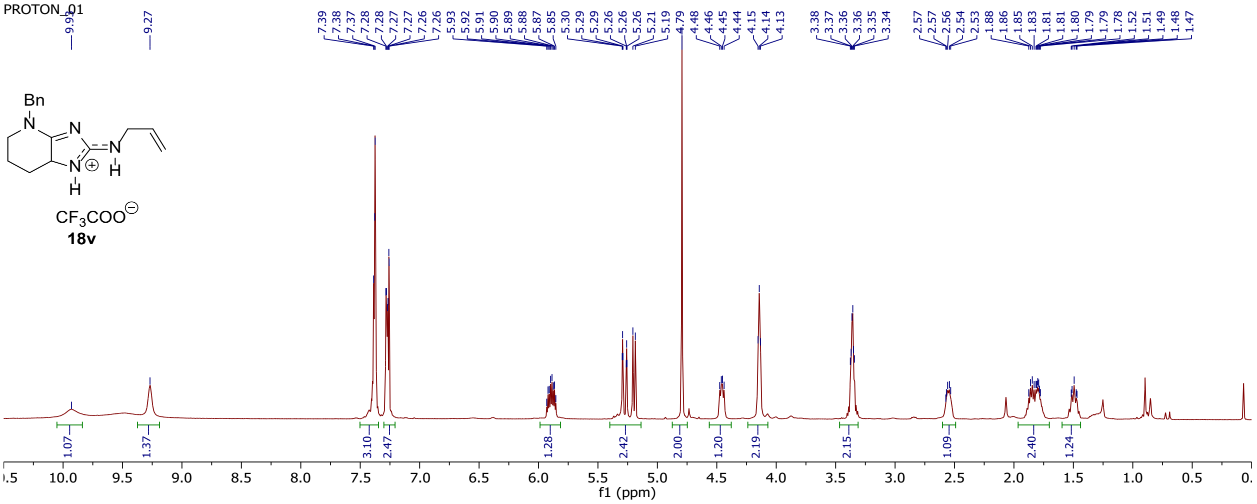
PROTON_01
Bn
N
N
N
H
H
CF3COO
18v
f1 (ppm)

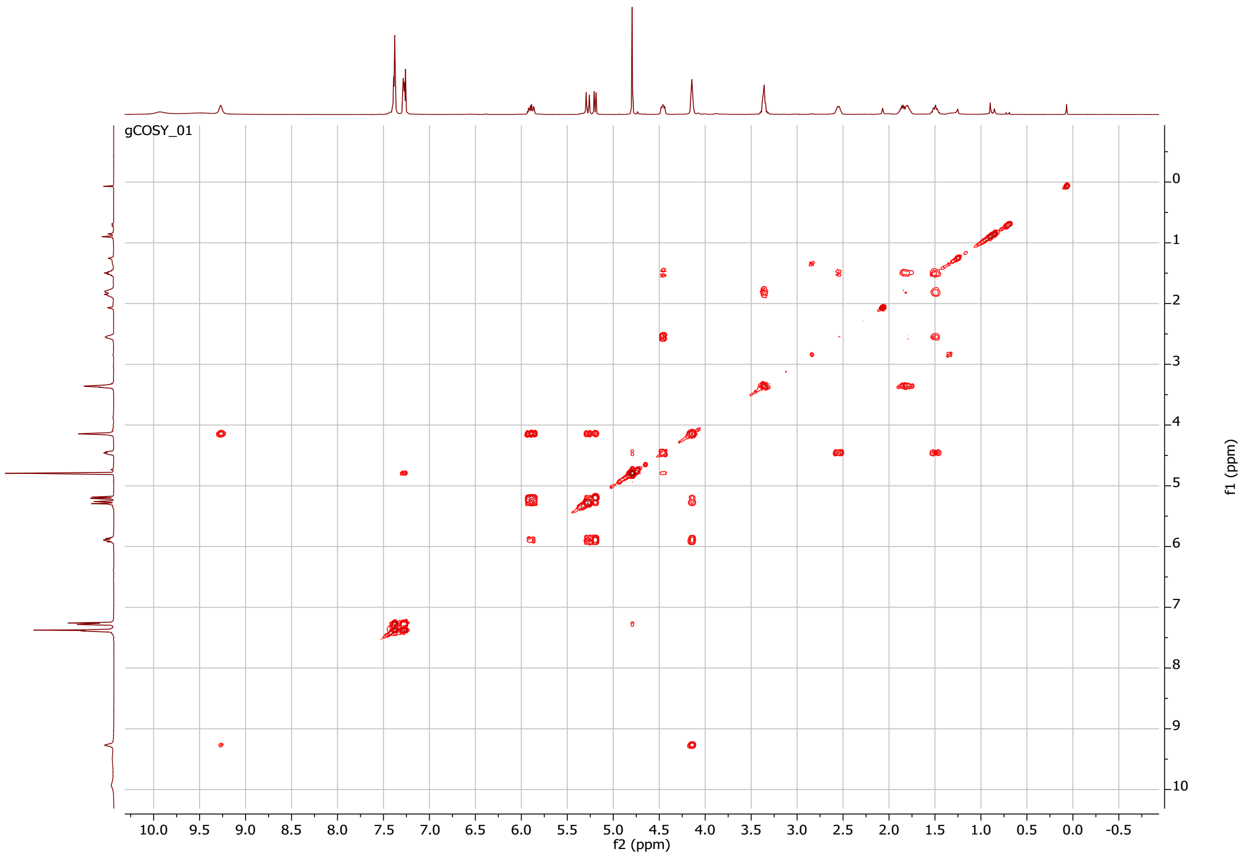
gCOSY_01
f2 (ppm)
f1 (ppm)

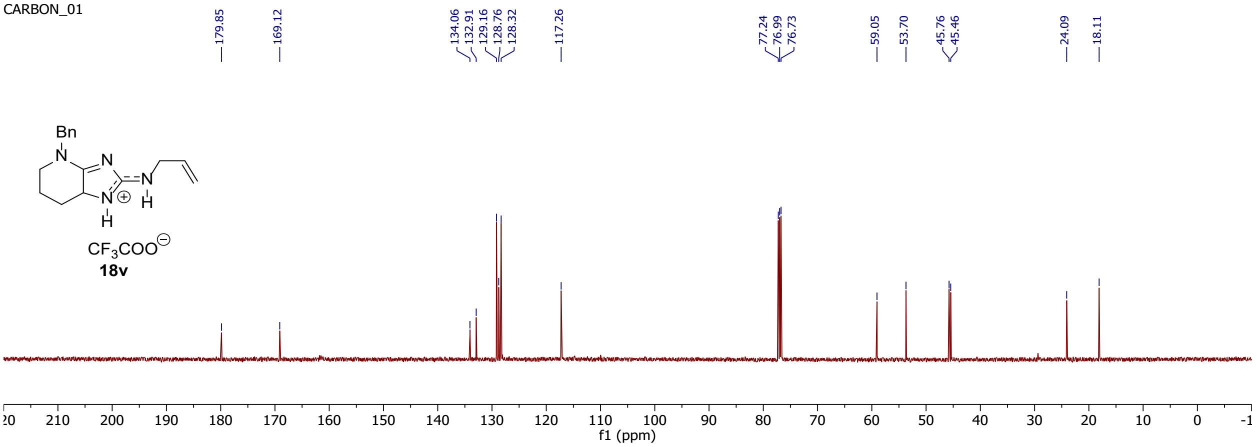
CARBON_01
179.85
169.12
134.06
132.91
129.16
128.76
128.32
117.26
77.24
76.99
76.73
59.05
53.70
45.76
45.46
24.09
18.11
Bn
N
N
N
N
H
H
CF3COO
18v
f1 (ppm)

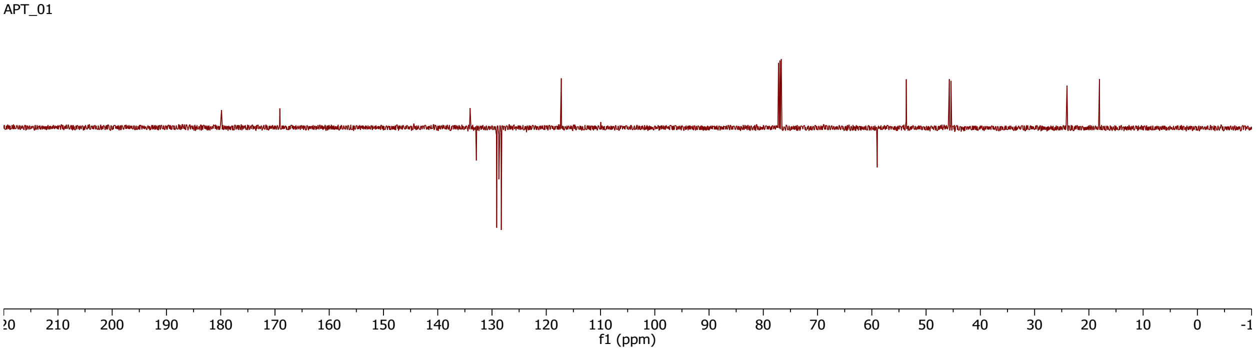
APT_01
f1 (ppm)

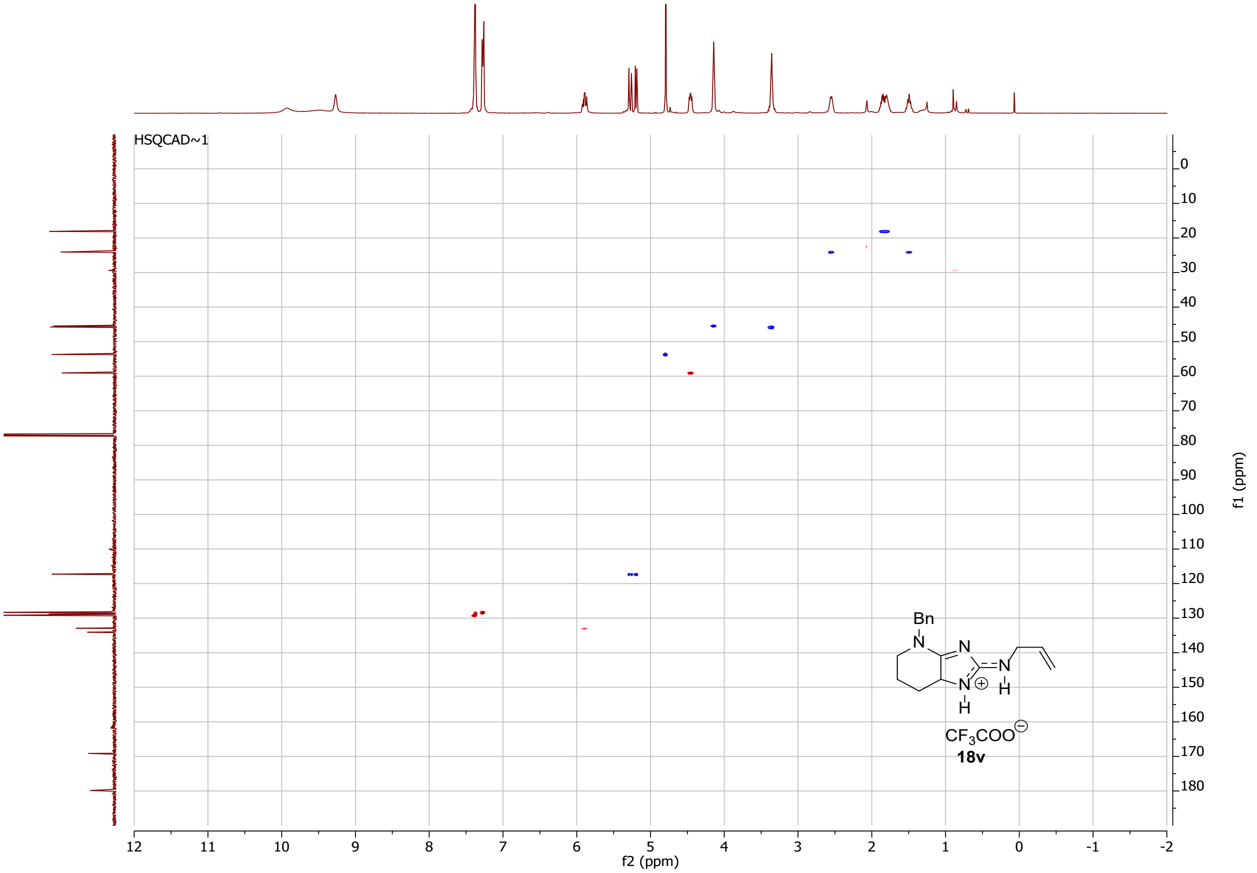
HSQCAD~1
Bn
N
N
N
N
H
H
CF3COO
18v
f2 (ppm)
f1 (ppm)

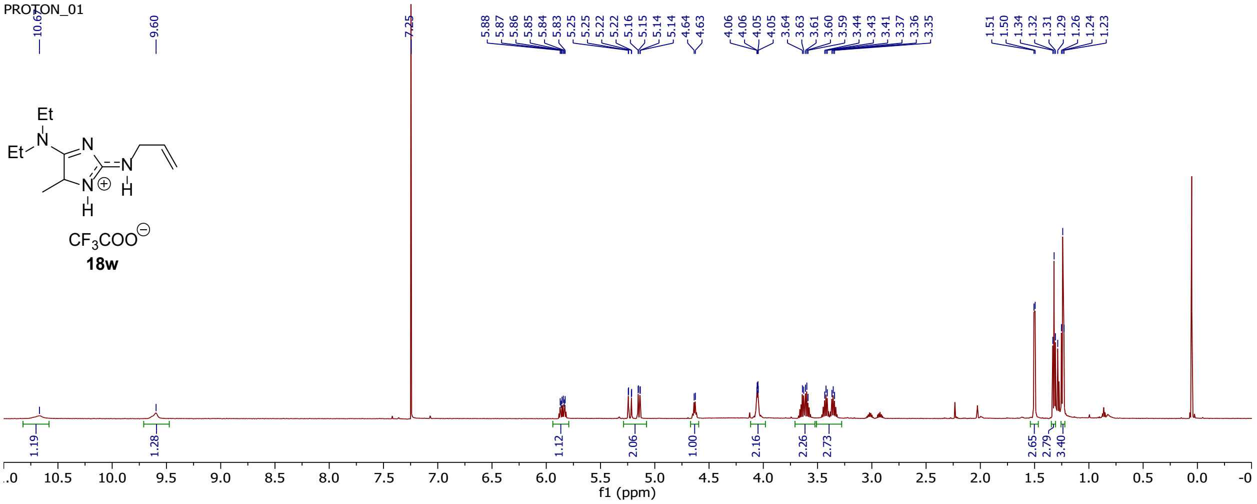
PROTON_01
Et
N
Et
N
N
H
H
CF3COO
18w
f1 (ppm)

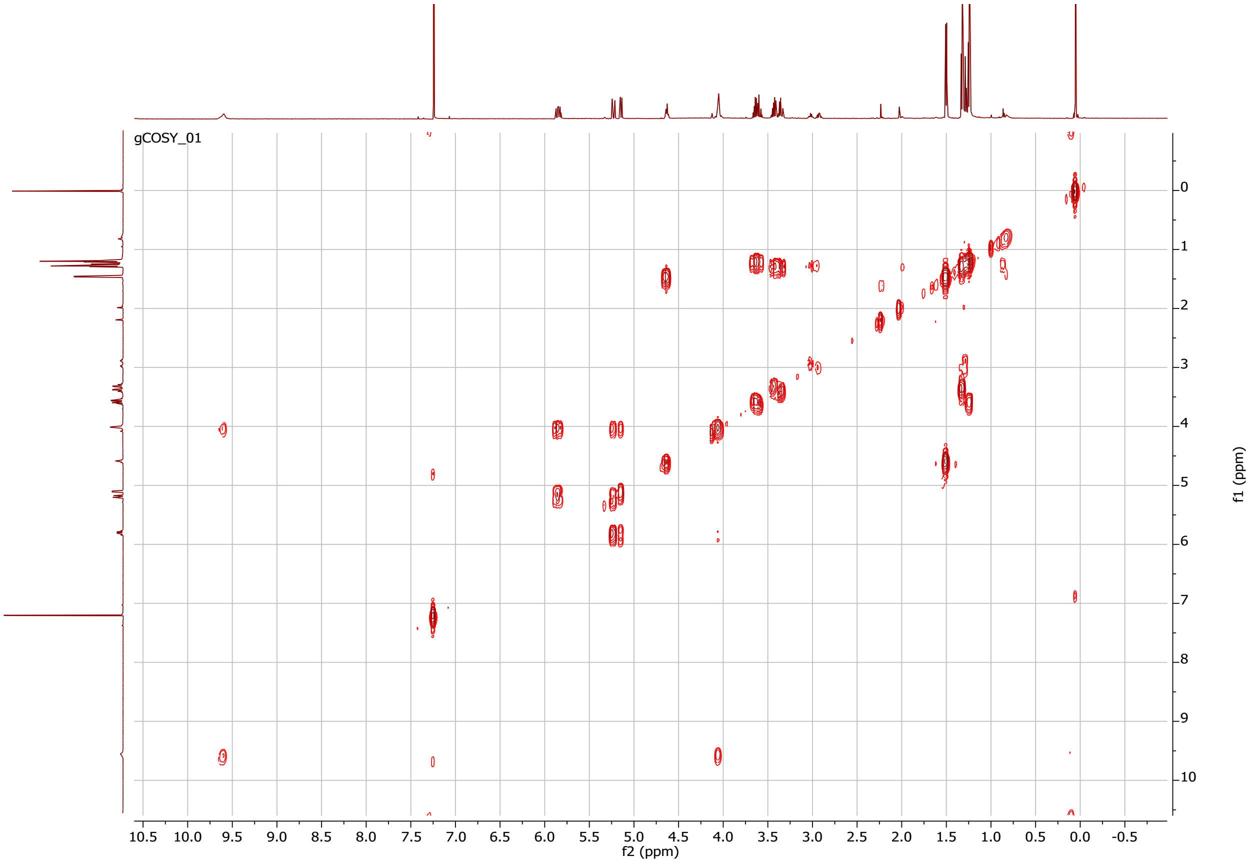
gCOSY_01
f2 (ppm)
f1 (ppm)

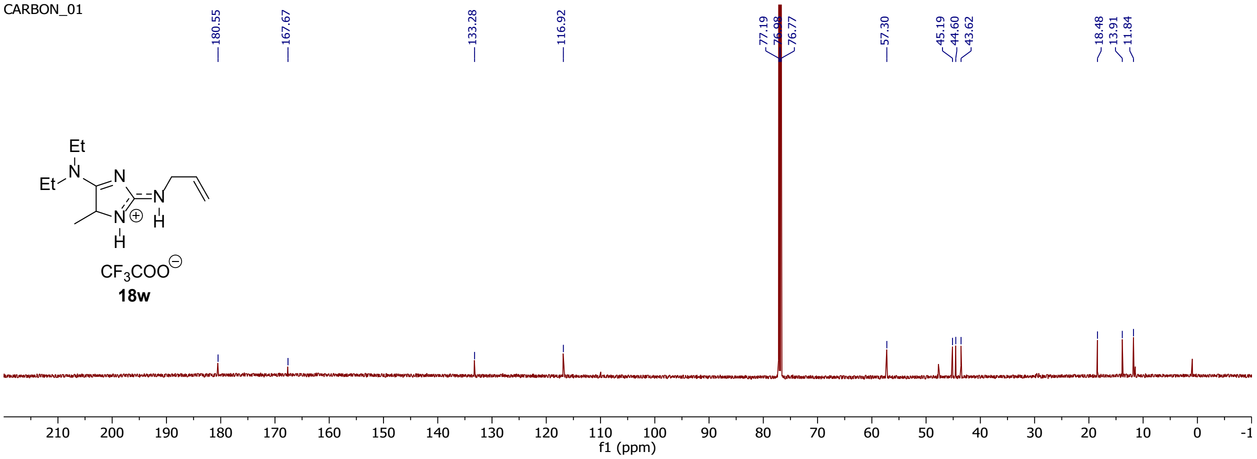
CARBON_01
180.55
167.67
133.28
116.92
77.19
76.98
76.77
57.30
45.19
44.60
43.62
18.48
13.91
11.84
Et
N
Et
N
N
N
H
H
$CF_3COO^{\ominus}$
18w
f1 (ppm)

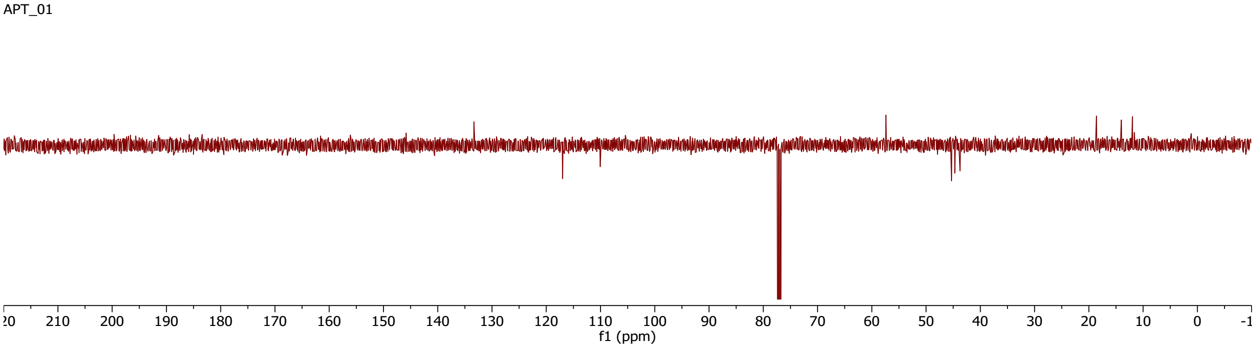
APT_01
f1 (ppm)

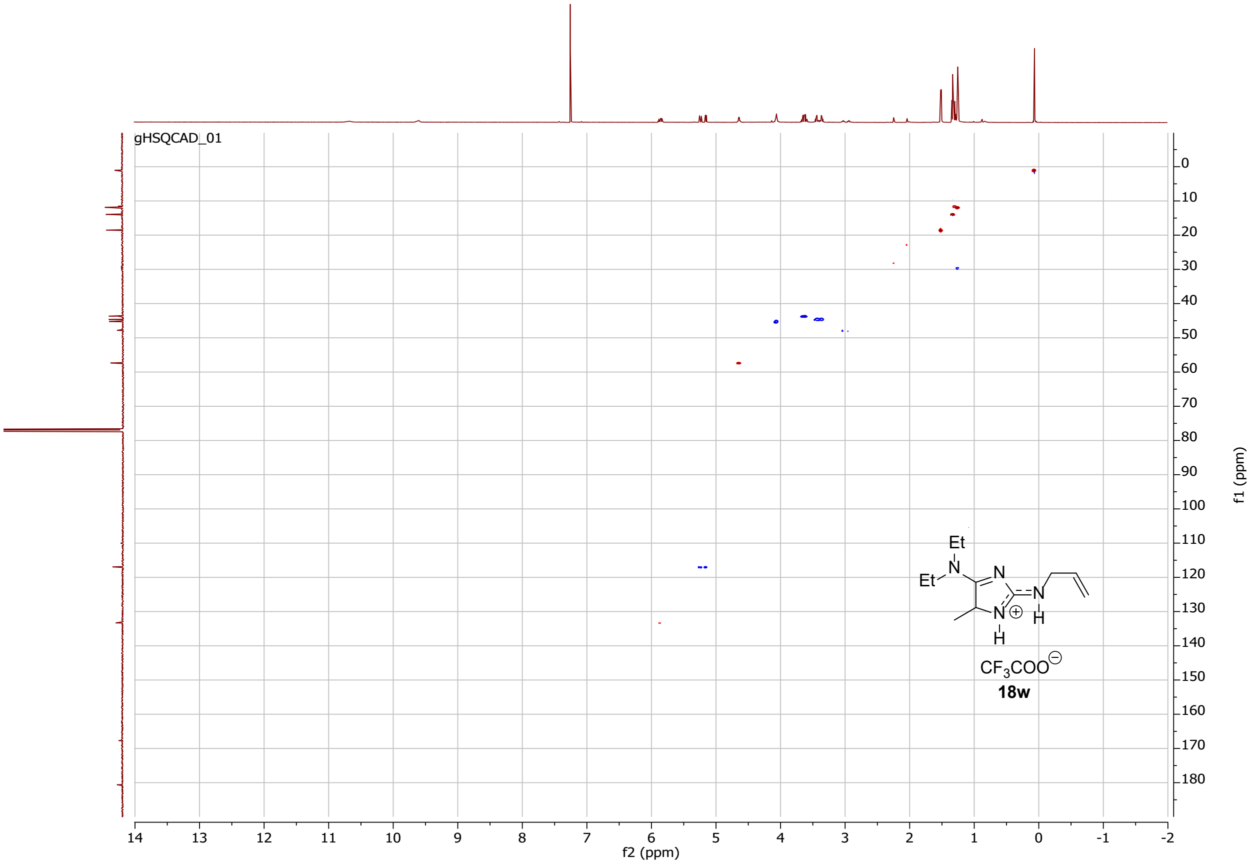
gHSQCAD_01
Et
N
Et
N
N
N
H
H
$CF_3COO^{\ominus}$
18w
f2 (ppm)
f1 (ppm)

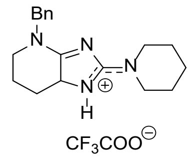
Bn
N
N
N
N
H
CF3COO

18x

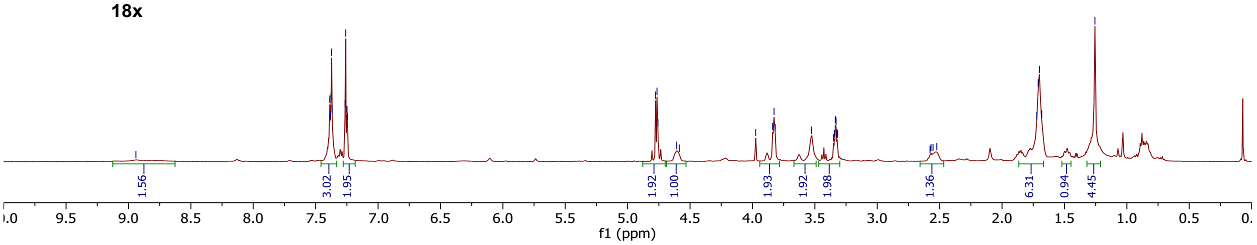
PROTON_01
8.94
7.39 7.38 7.38 7.37 7.37 7.26 7.26 7.25 7.25
4.78 4.76 4.76 4.61 4.59
3.97 3.84 3.83 3.82 3.53 3.35 3.34 3.34 3.33 3.32 3.32
2.58 2.57 2.55 2.52
1.72 1.71 1.70 1.68
1.26
1.56 3.02 1.95 1.92 1.00 1.93 1.92 1.98 1.36 6.31 0.94 4.45
f1 (ppm)

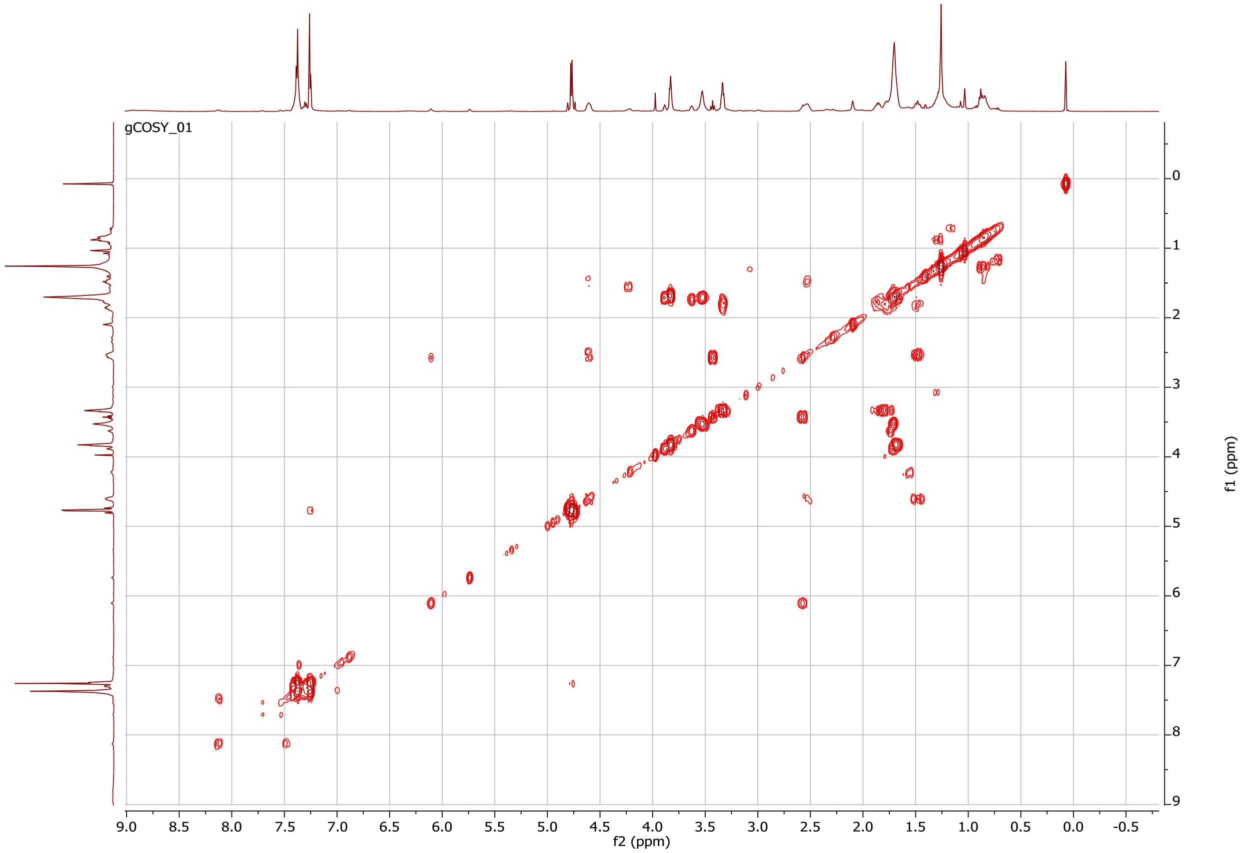
gCOSY_01
f2 (ppm)
f1 (ppm)

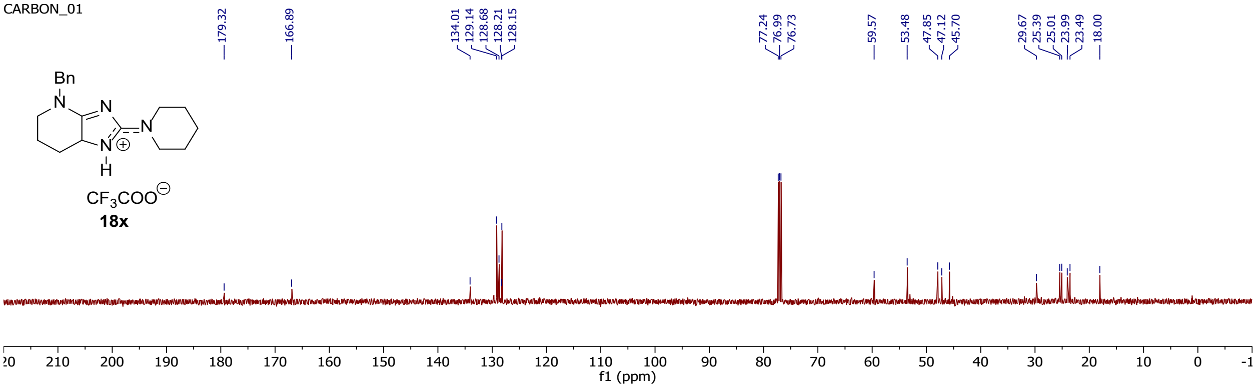

CARBON_01
179.32
166.89
134.01
129.14
128.68
128.21
128.15
77.24
76.99
76.73
59.57
53.48
47.85
47.12
45.70
29.67
25.39
25.01
23.99
23.49
18.00
Bn
N
N
N
N
H
CF3COO
18x
f1 (ppm)

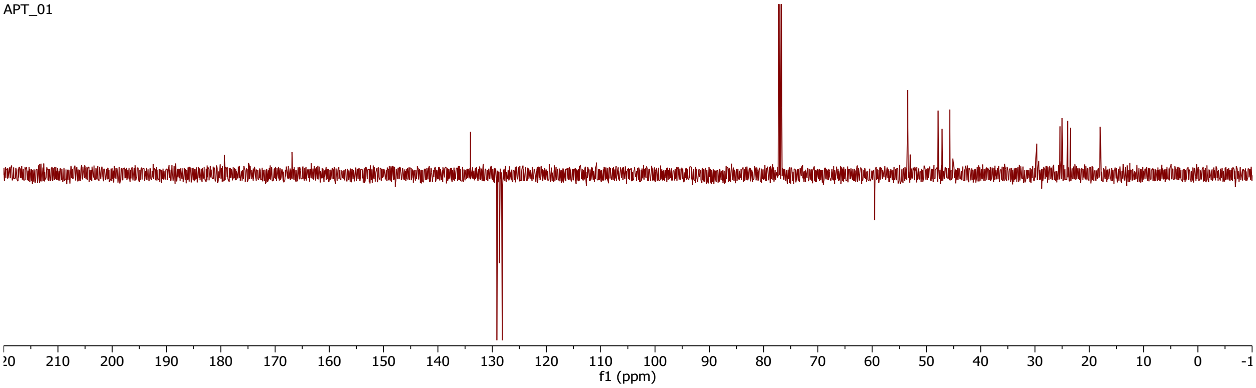

APT_01
f1 (ppm)

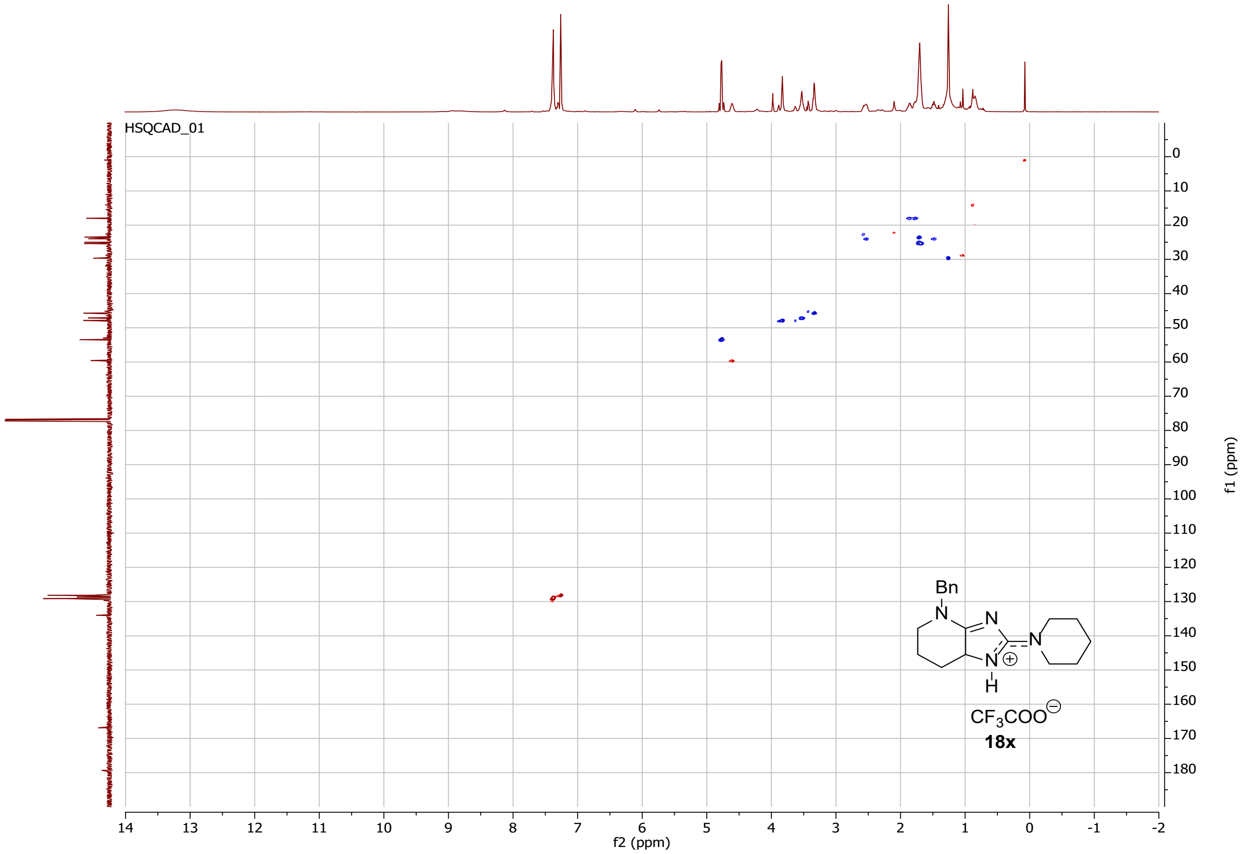

HSQCAD_01
Bn
N
N
N
N
H
CF3COO
18x
f2 (ppm)
f1 (ppm)

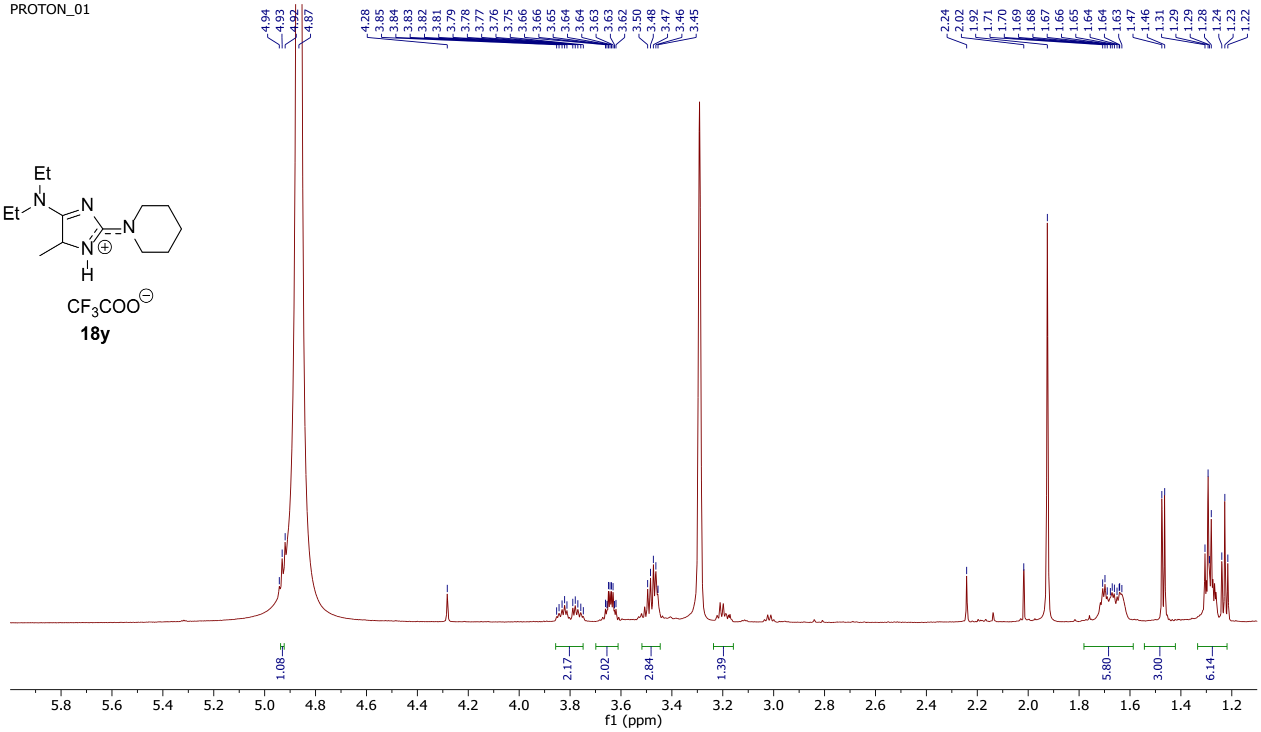

PROTON_01
4.94
4.93
4.92
4.87
4.28
3.85
3.84
3.83
3.82
3.81
3.79
3.78
3.77
3.76
3.75
3.66
3.66
3.65
3.64
3.64
3.63
3.63
3.62
3.50
3.48
3.47
3.46
3.45
2.24
2.02
1.92
1.71
1.70
1.69
1.68
1.67
1.66
1.65
1.64
1.64
1.63
1.47
1.46
1.31
1.29
1.29
1.28
1.24
1.23
1.22
Et
N
Et
N
N
N
H
$CF_3COO^{\ominus}$
1.08
2.17
2.02
2.84
1.39
5.80
3.00
6.14
f1 (ppm)

18y

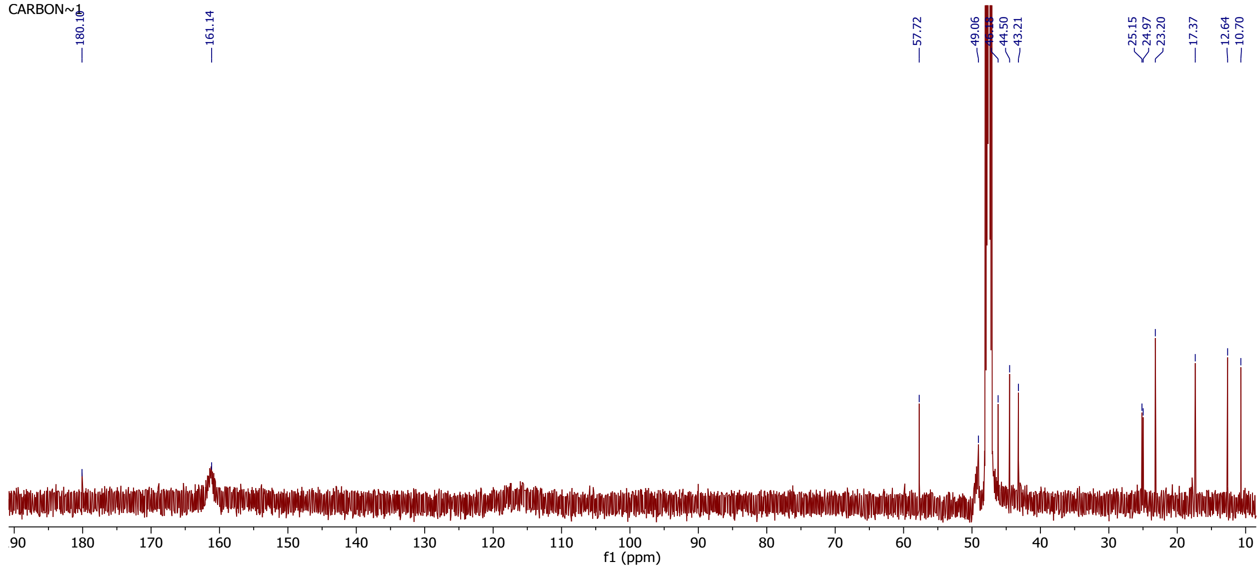

CARBON~1
180.10
161.14
57.72
49.06
44.50
43.21
25.15
24.97
23.20
17.37
12.64
10.70
f1 (ppm)

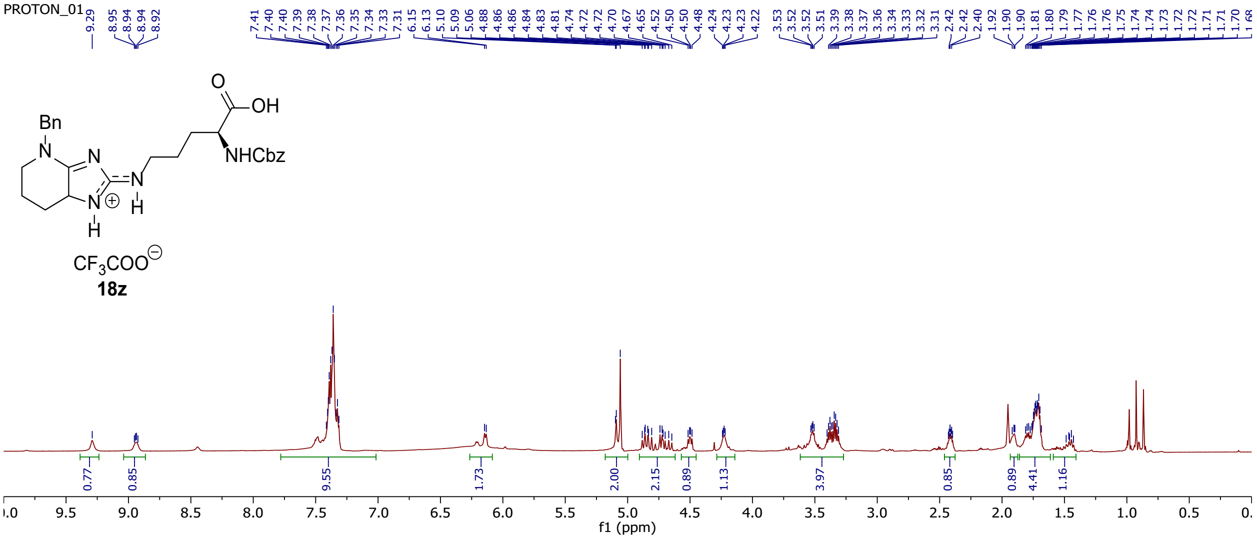
PROTON_01
CF3COO
18z
f1 (ppm)

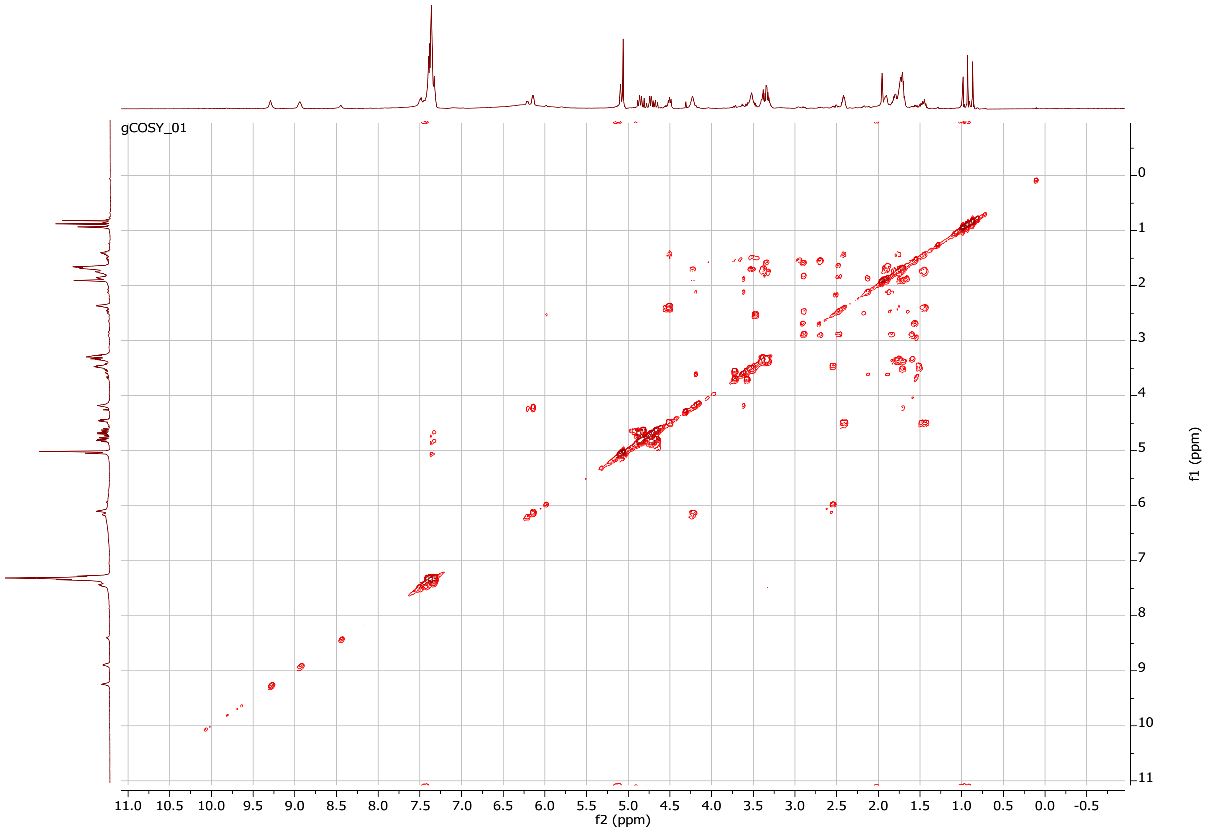
gCOSY_01
f2 (ppm)
f1 (ppm)

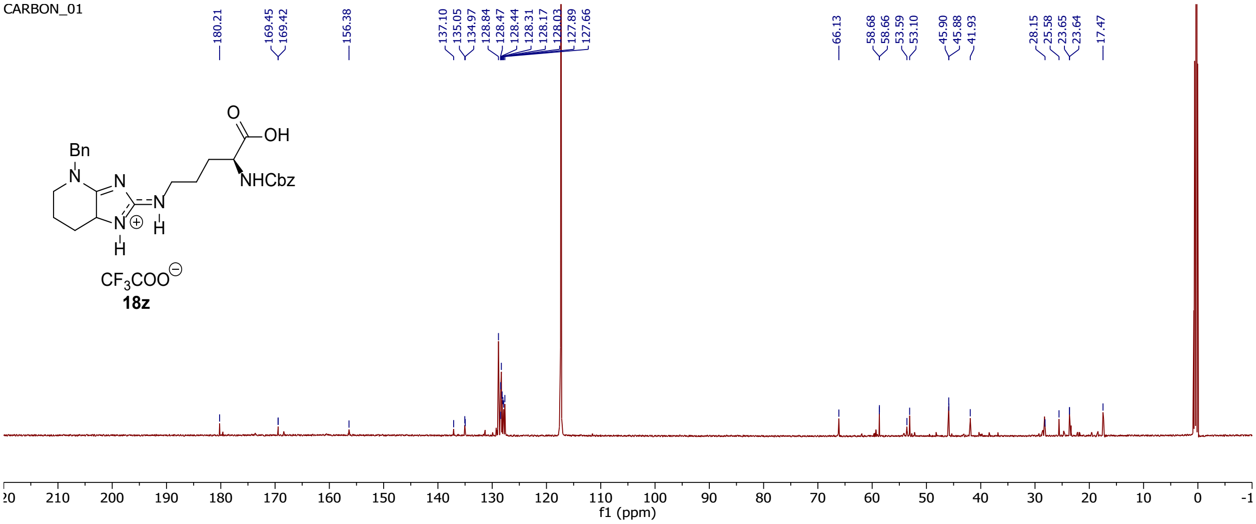
CARBON_01
180.21
169.45
169.42
156.38
137.10
135.05
134.97
128.84
128.47
128.44
128.31
128.17
128.03
127.89
127.66
66.13
58.68
58.66
53.59
53.10
45.90
45.88
41.93
28.15
25.58
23.65
23.64
17.47
Bn
N
N
N
N
H
H
O
OH
NHCbz
$CF_3COO^{\ominus}$
18z
f1 (ppm)

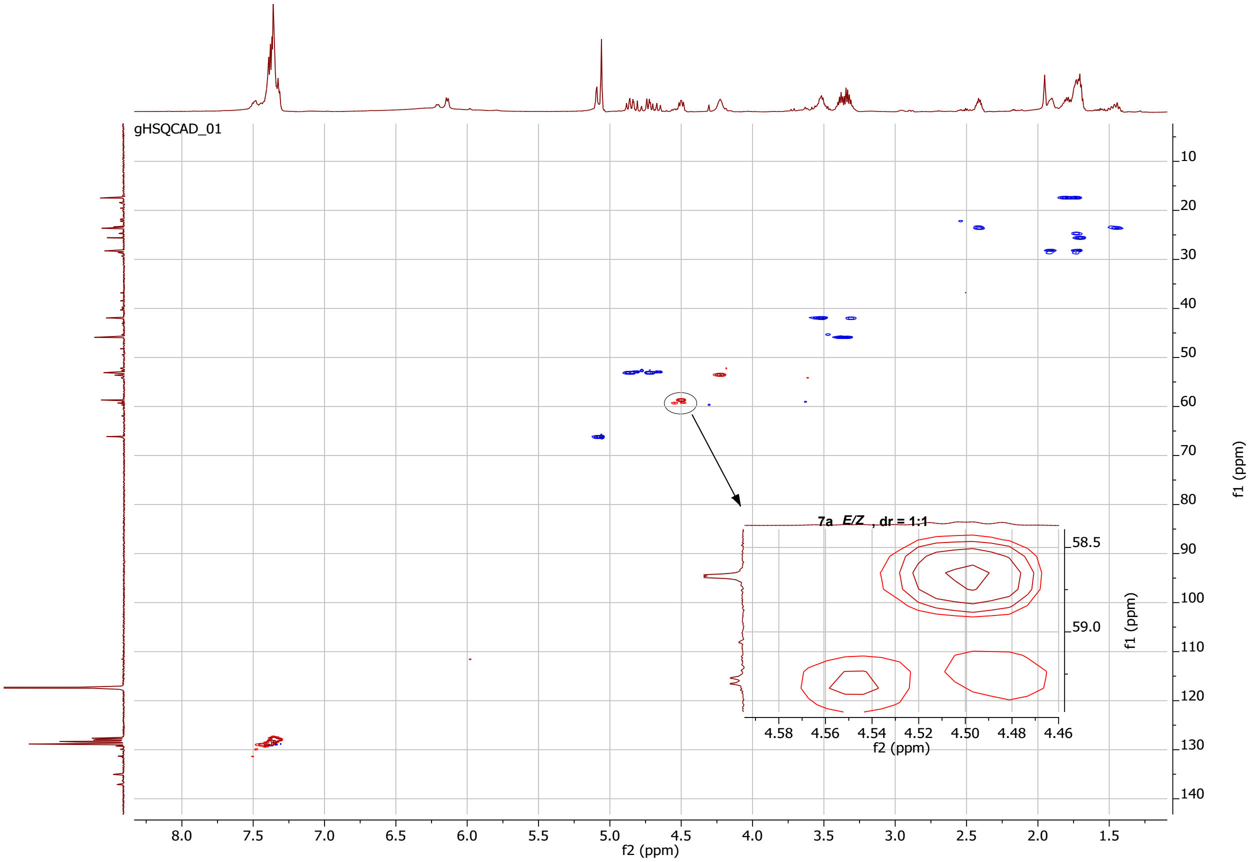
gHSQCAD_01
7a E/Z , dr = 1:1
58.5
59.0
f1 (ppm)
f2 (ppm)

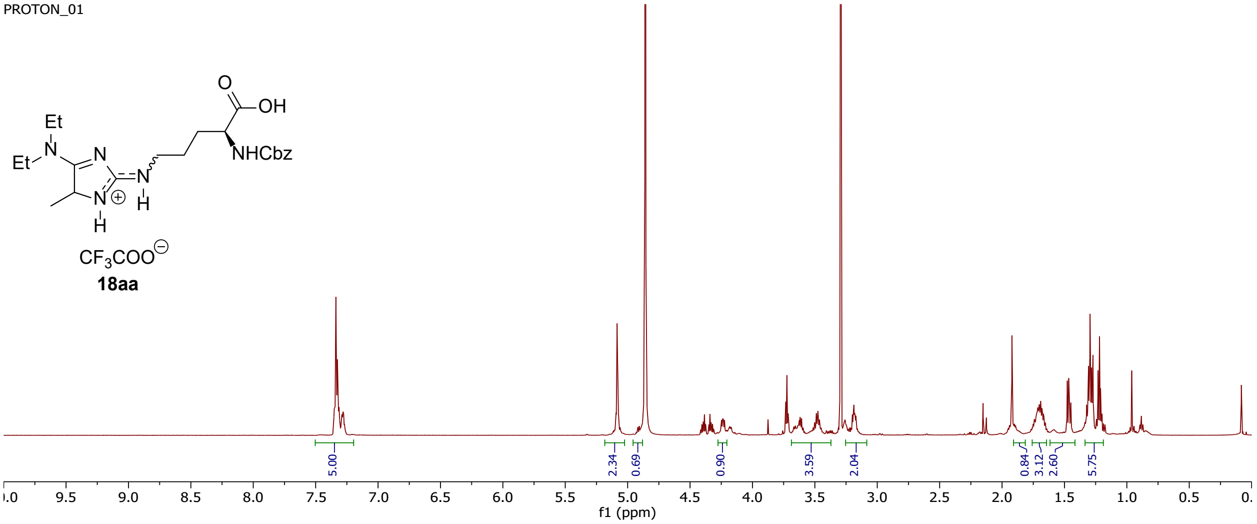
PROTON_01
Et
N
Et
N
N
N
H
H
O
OH
NHCbz
CF3COO
18aa
5.00
2.34
0.69
0.90
3.59
2.04
0.84
3.12
2.60
5.75
f1 (ppm)

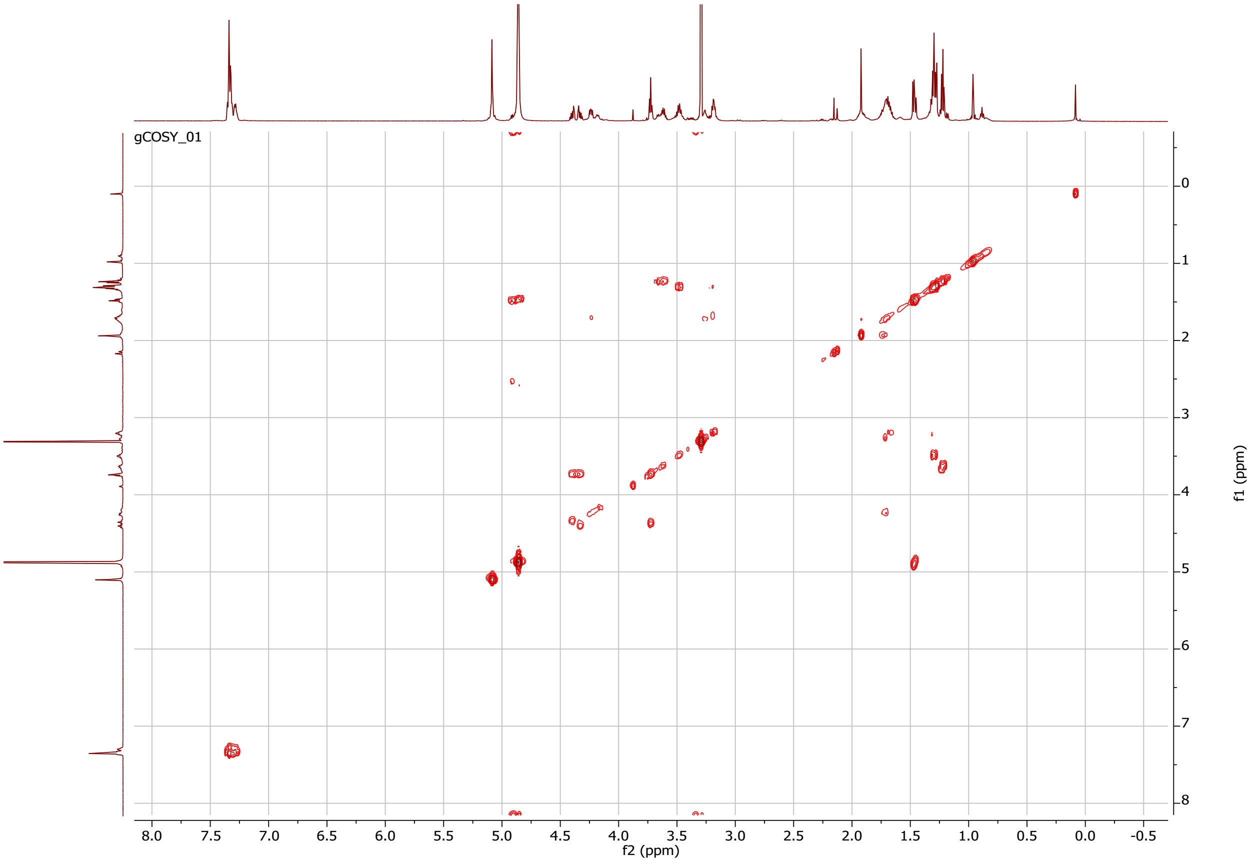
gCOSY_01
f2 (ppm)
f1 (ppm)

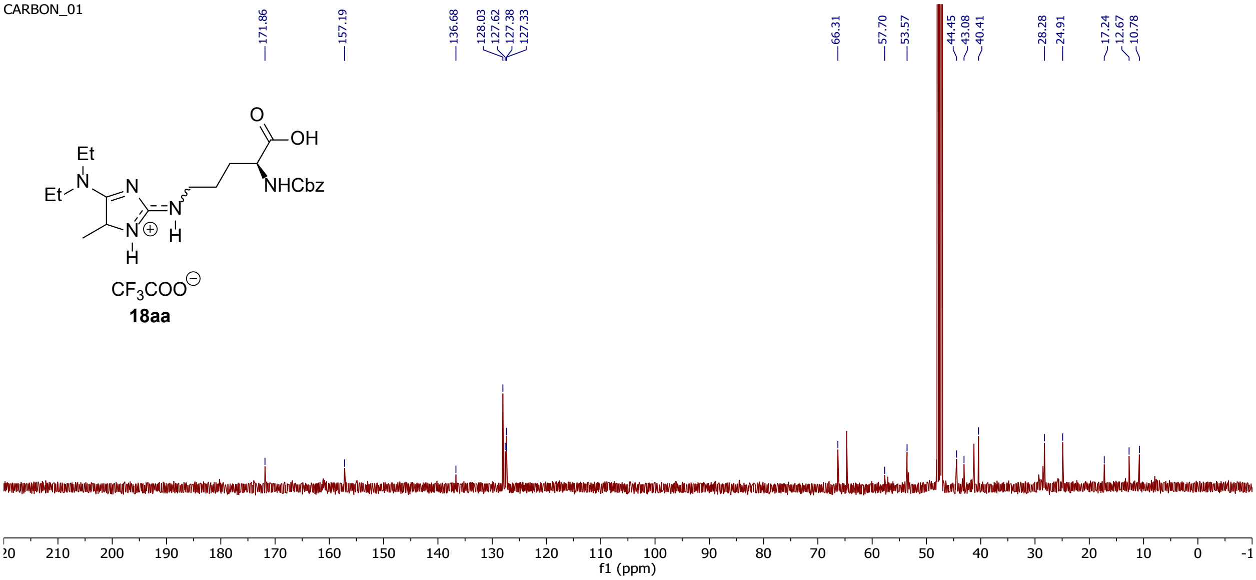
CARBON_01
171.86
157.19
136.68
128.03
127.62
127.38
127.33
66.31
57.70
53.57
44.45
43.08
40.41
28.28
24.91
17.24
12.67
10.78
O
OH
Et
N
Et
N
N
N
H
H
NHCbz
CF3COO
18aa
f1 (ppm)

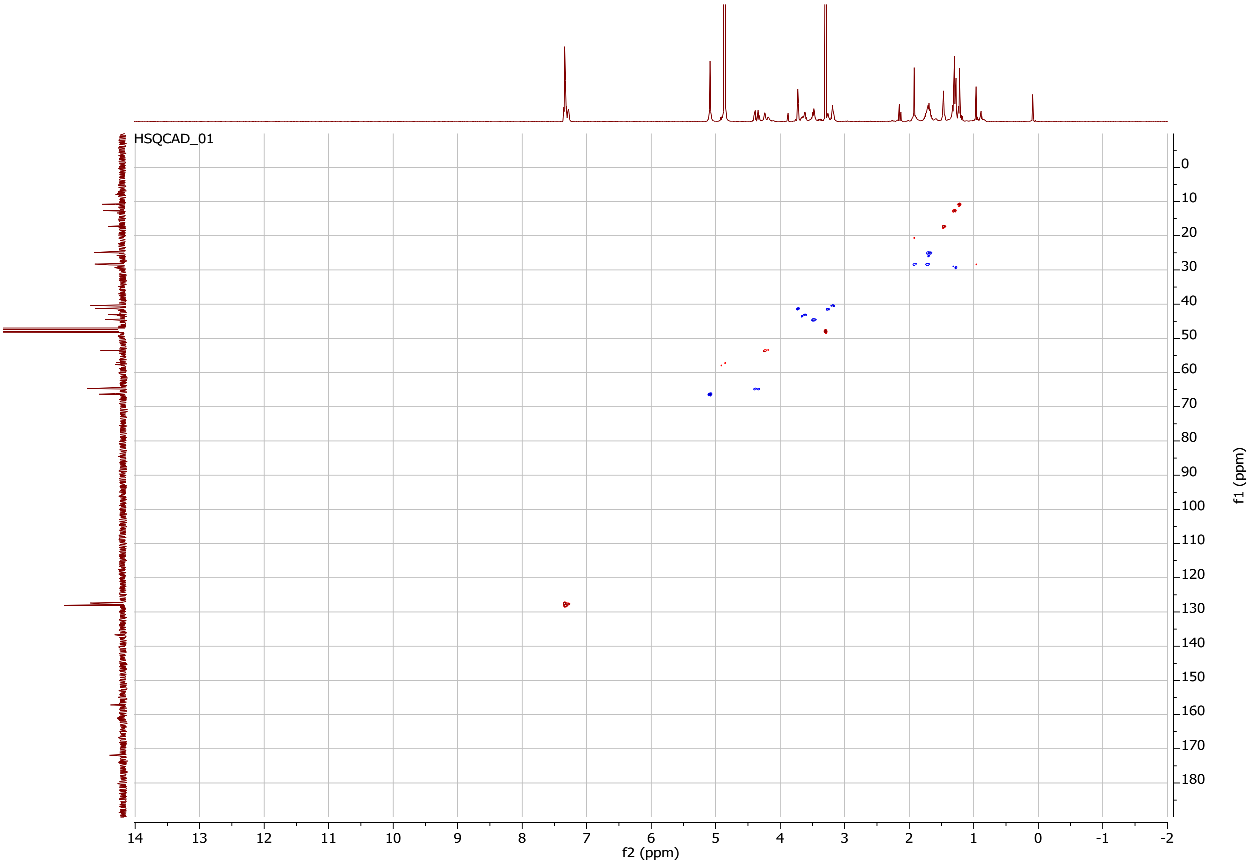
HSQCAD_01
f2 (ppm)
f1 (ppm)

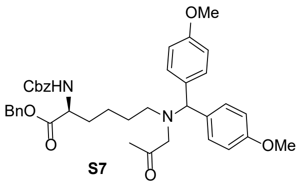

S7

PROTON_01

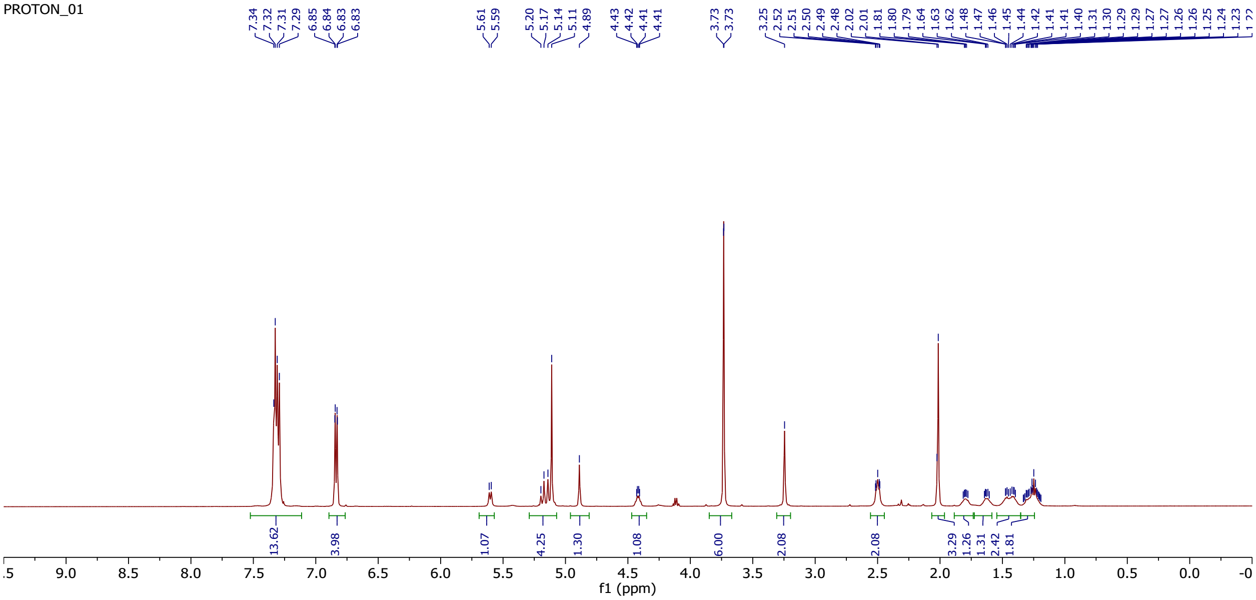

CARBON_01

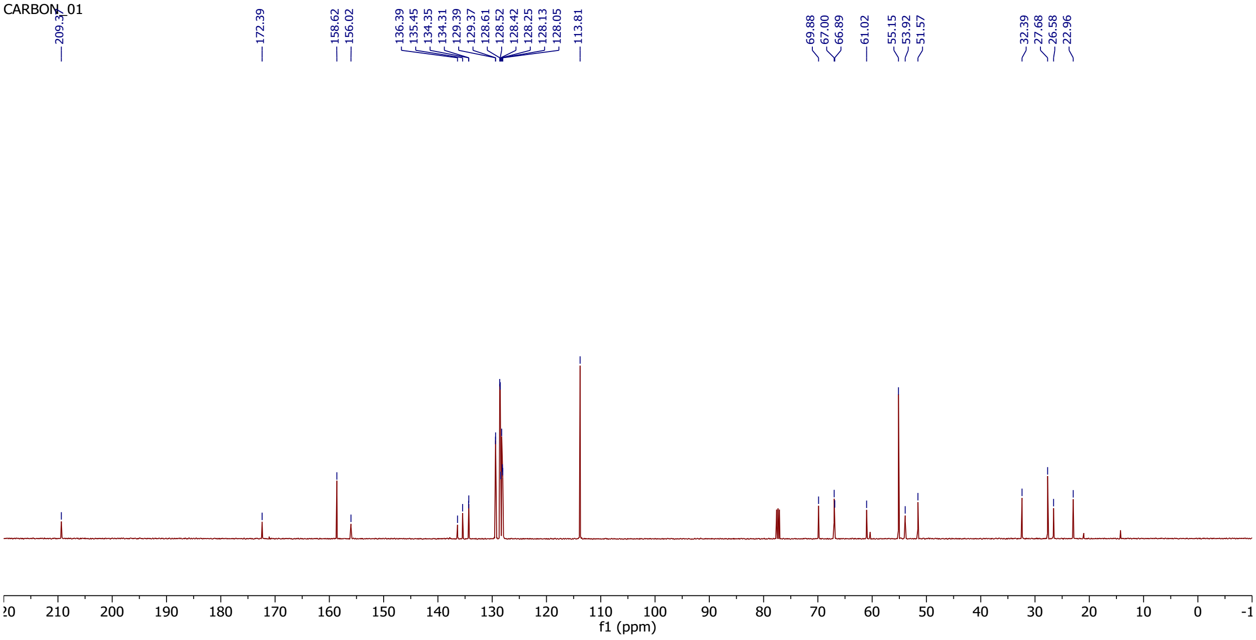

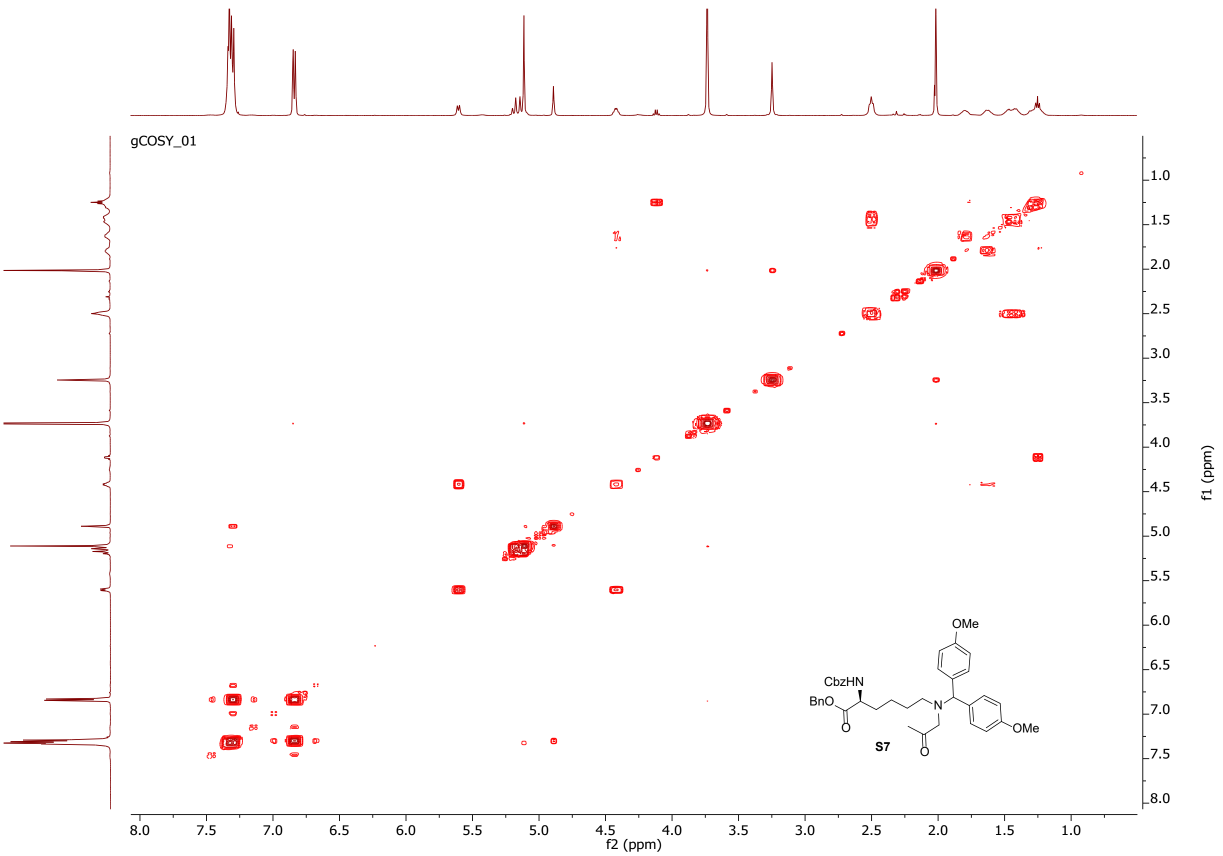

gCOSY_01
OMe
CbzHN
BnO
O
N
OMe
O
S7
f2 (ppm)
f1 (ppm)

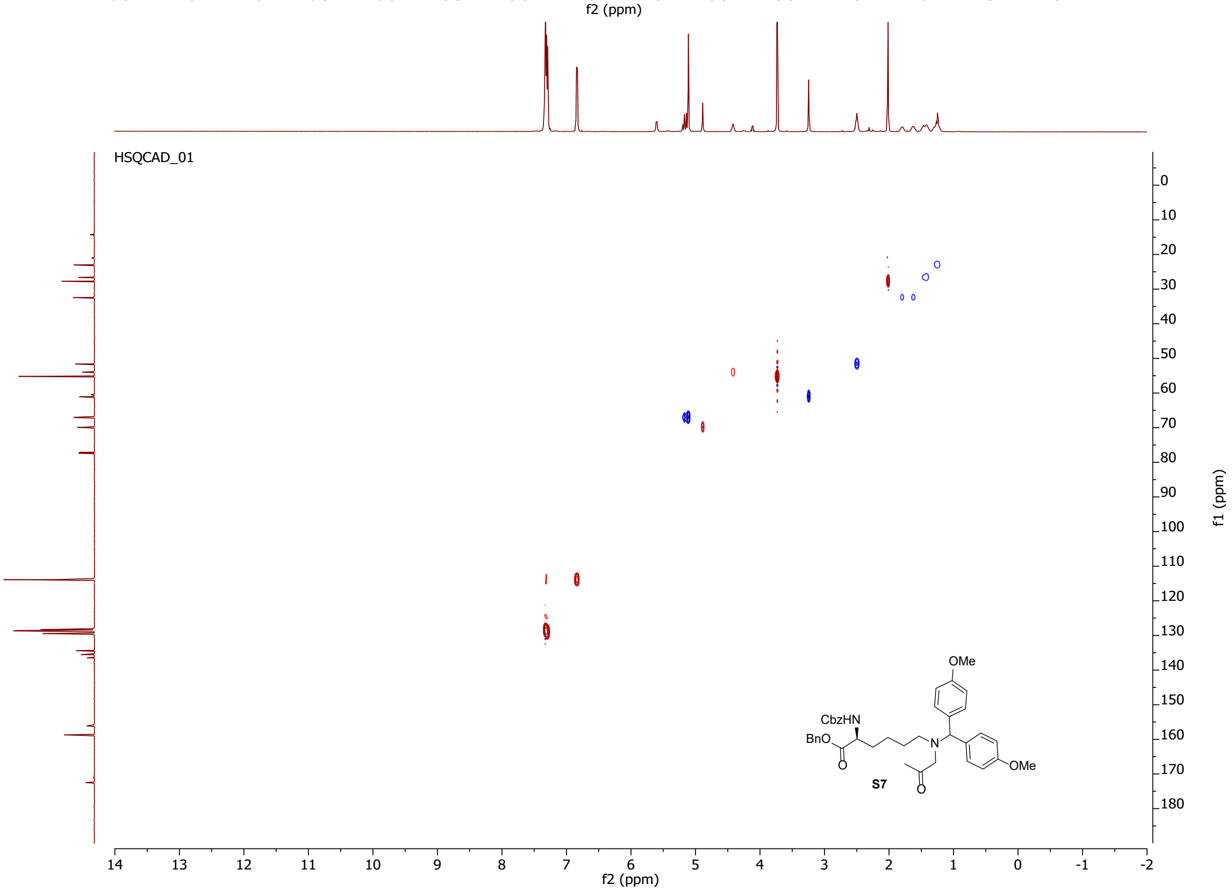

HSQCAD_01
OMe
CbzHN
BnO
O
N
OMe
O
S7
f2 (ppm)
f1 (ppm)

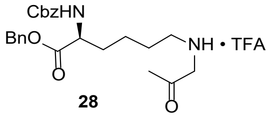

28

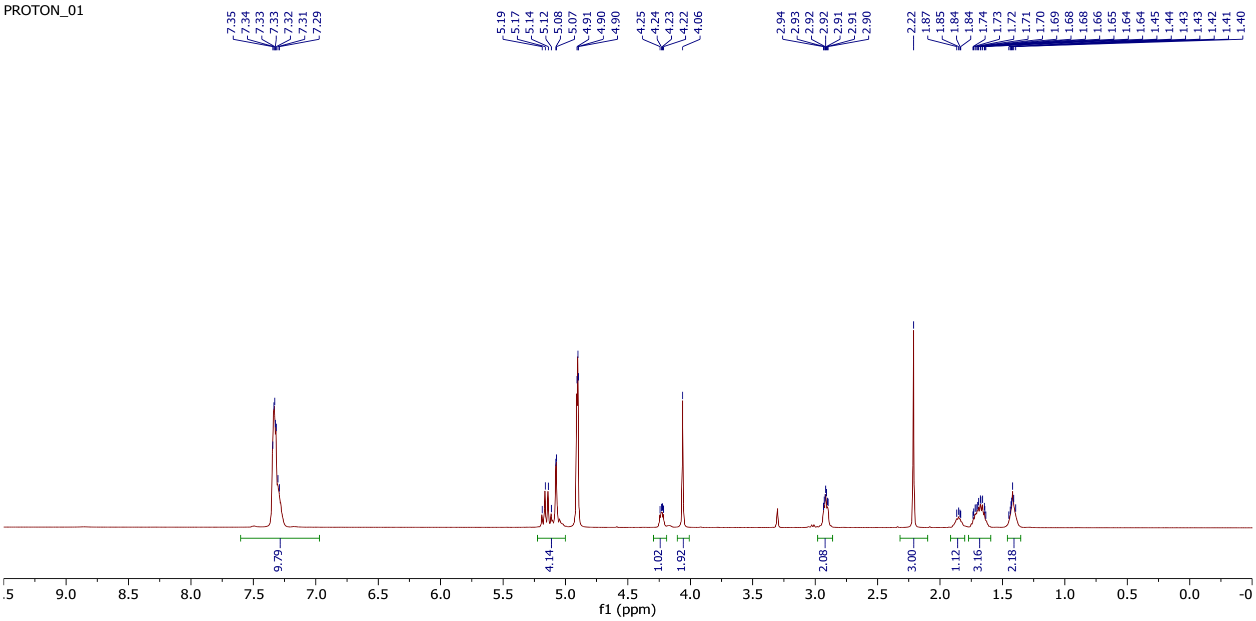
PROTON_01
7.35
7.34
7.33
7.33
7.32
7.31
7.29
5.19
5.17
5.14
5.12
5.08
5.07
4.91
4.90
4.90
4.25
4.24
4.23
4.22
4.06
2.94
2.93
2.92
2.92
2.91
2.91
2.90
2.22
1.87
1.85
1.84
1.84
1.74
1.73
1.72
1.71
1.70
1.69
1.68
1.68
1.66
1.65
1.64
1.64
1.64
1.45
1.44
1.43
1.43
1.42
1.41
1.40
9.79
4.14
1.02
1.92
2.08
3.00
1.12
3.16
2.18
f1 (ppm)

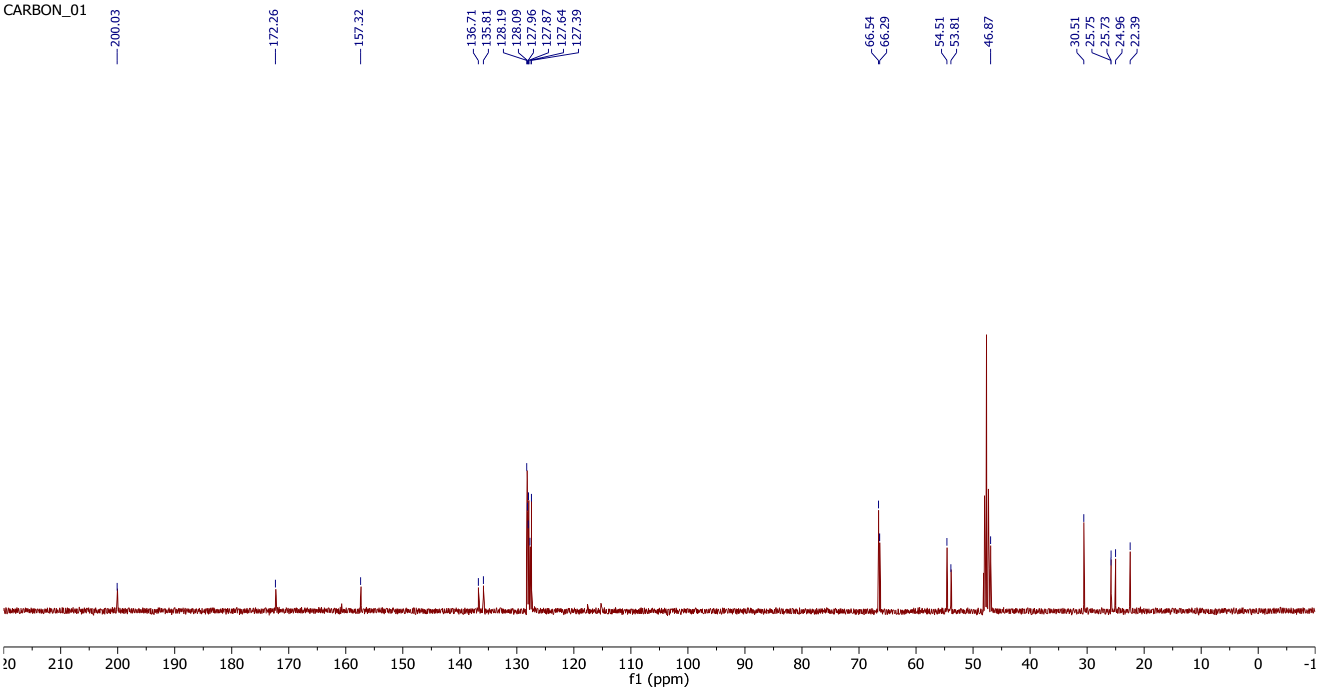
CARBON_01
200.03
172.26
157.32
136.71
135.81
128.19
128.09
127.96
127.87
127.64
127.39
66.54
66.29
54.51
53.81
46.87
30.51
25.75
25.73
24.96
22.39
f1 (ppm)

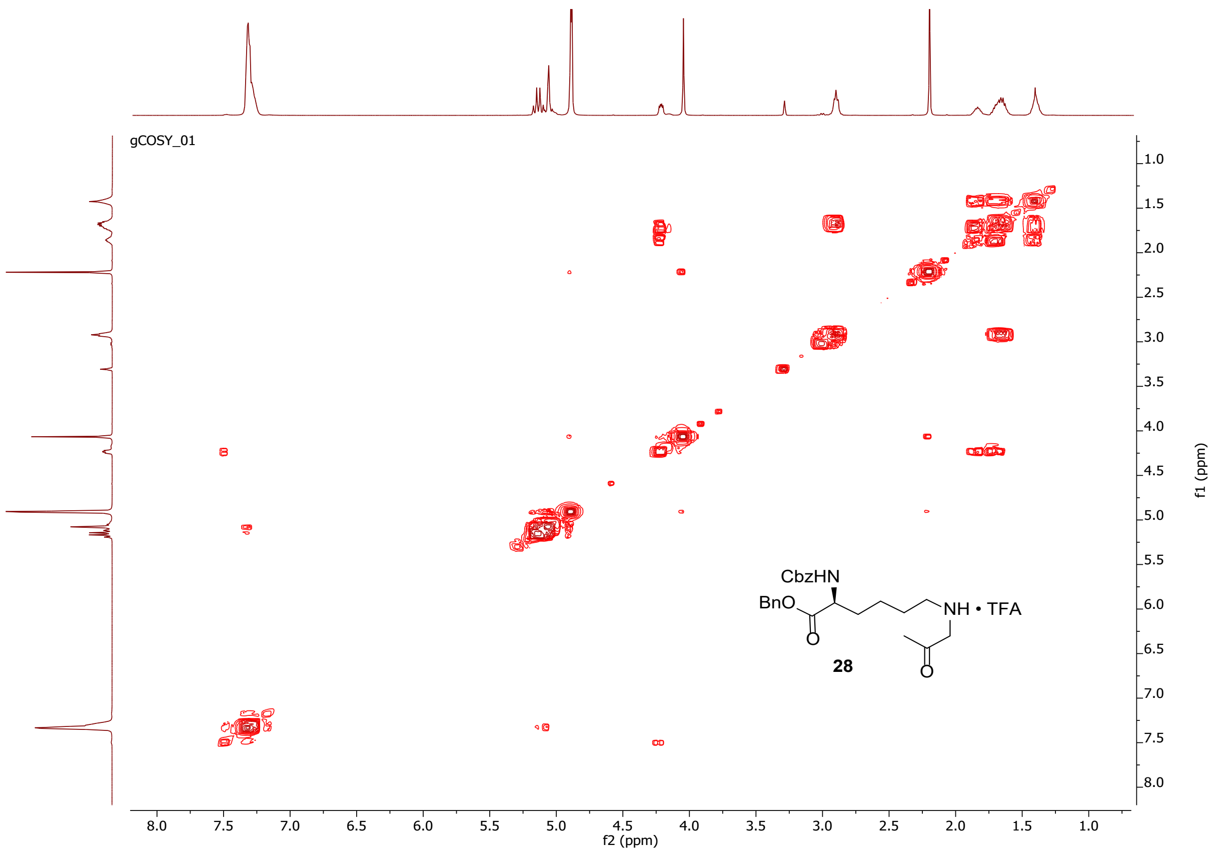
gCOSY_01
CbzHN
BnO
NH • TFA
O
O
28
f2 (ppm)
f1 (ppm)

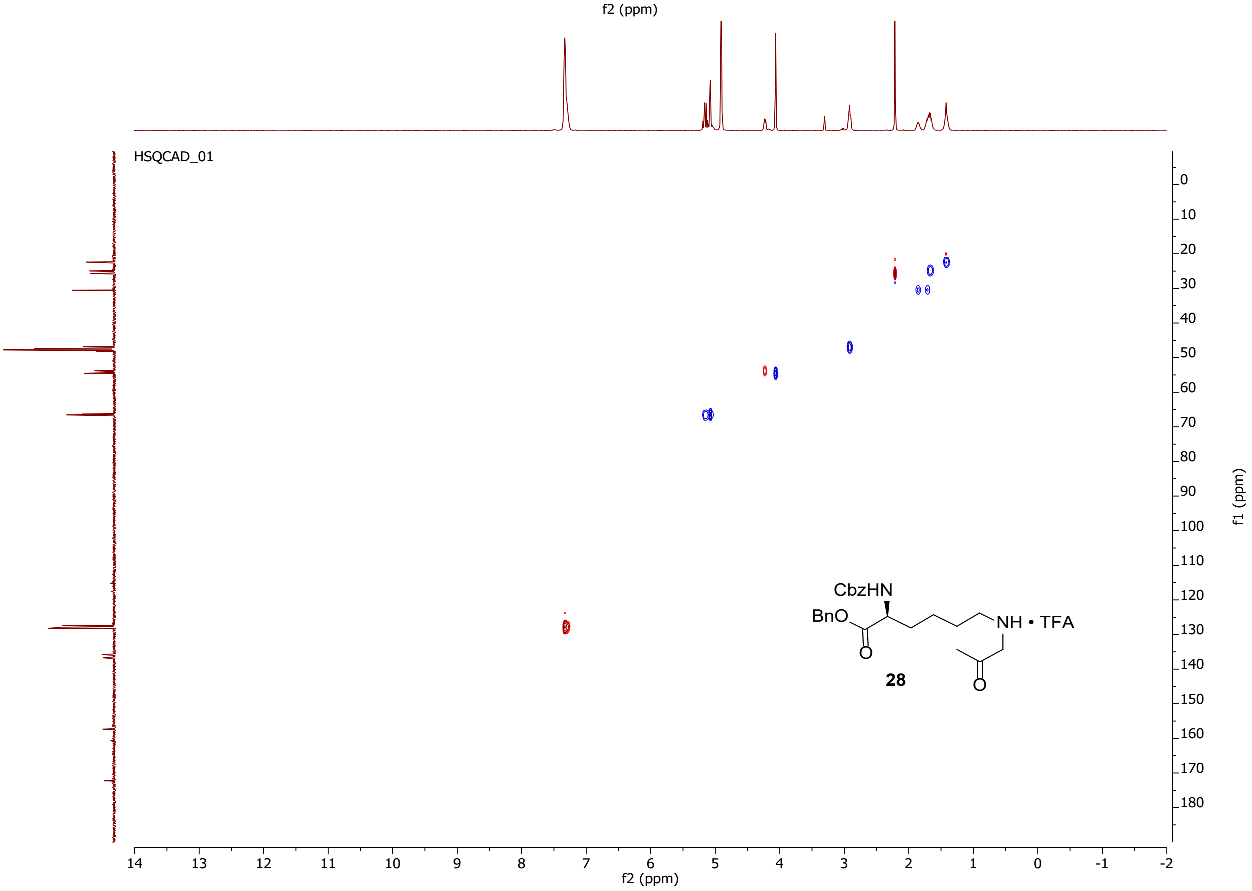
HSQCAD_01
CbzHN
BnO
NH • TFA
O
O
28
f2 (ppm)
f1 (ppm)

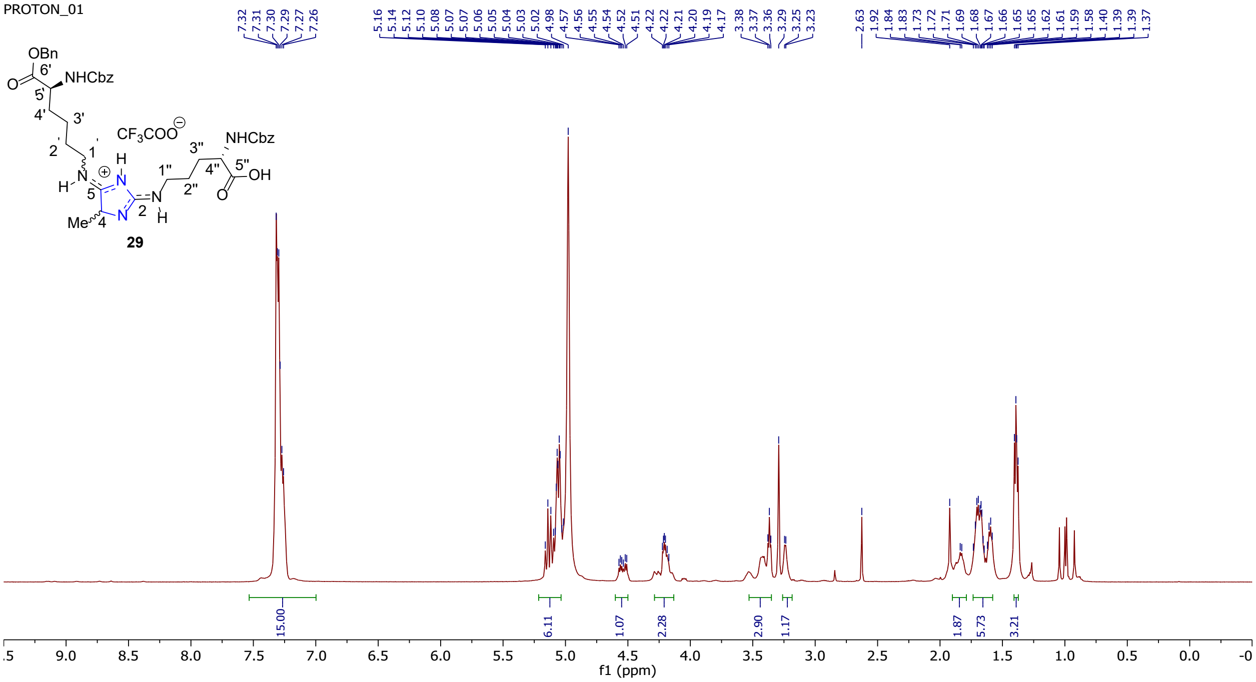
PROTON_01
OBn
NHCbz
CF3COO
NHCbz
OH
Me
29
f1 (ppm)

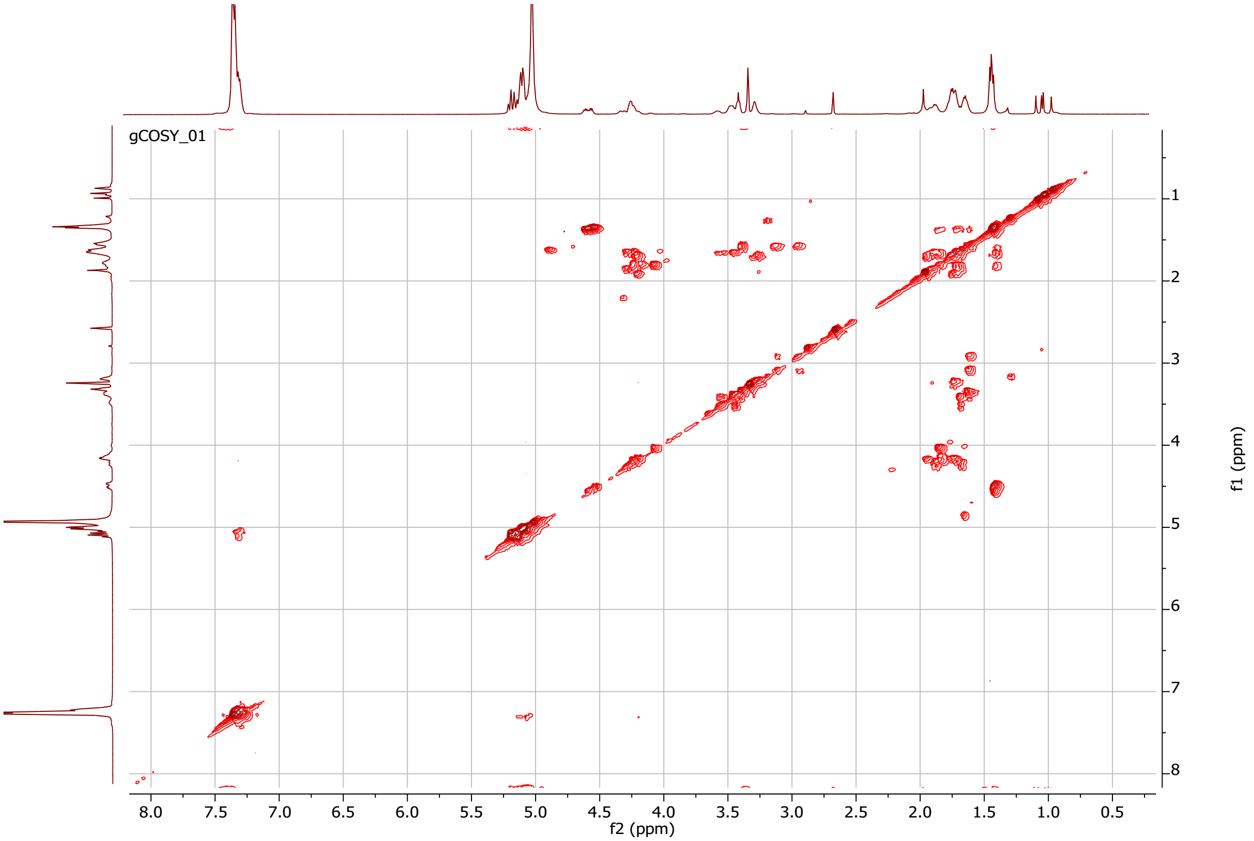
gCOSY_01
f2 (ppm)
f1 (ppm)

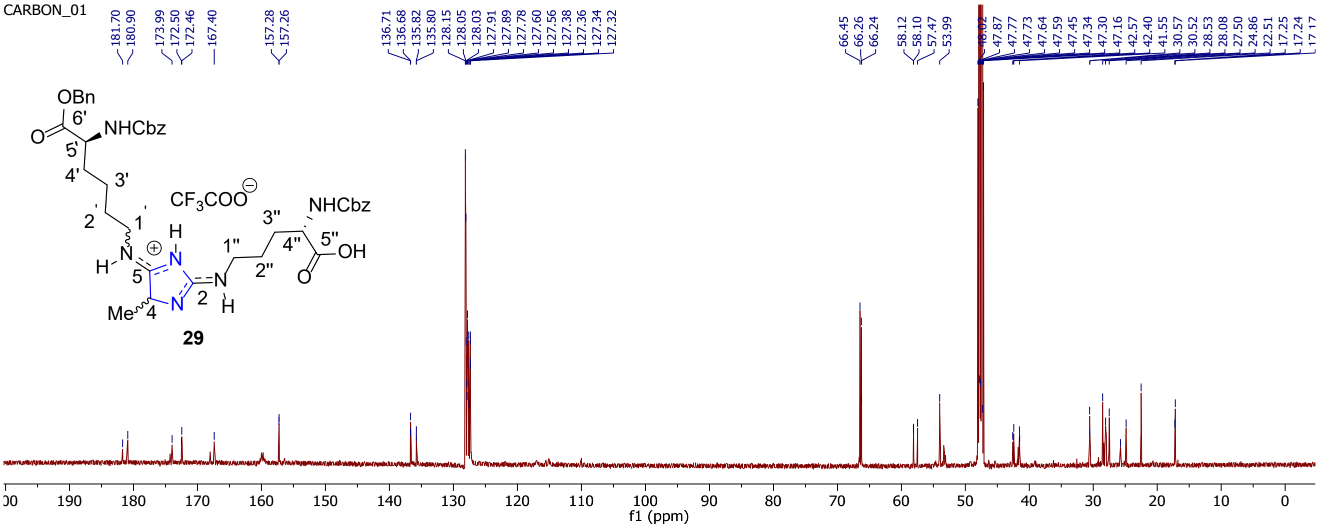
CARBON_01
181.70
180.90
173.99
172.50
172.46
167.40
157.28
157.26
136.71
136.68
135.82
135.80
128.15
128.05
128.03
127.91
127.89
127.78
127.60
127.56
127.38
127.36
127.34
127.32
66.45
66.26
66.24
58.12
58.10
57.47
53.99
47.87
47.77
47.73
47.64
47.59
47.45
47.34
47.30
47.16
42.57
42.40
41.55
30.57
30.52
28.53
28.08
27.50
24.86
22.51
17.25
17.24
OBn
6'
NHCbz
5'
4'
3'
2'
1'
CF3COO
3"
NHCbz
4"
5"
OH
1"
2"
2
4
5
Me
H
29
f1 (ppm)

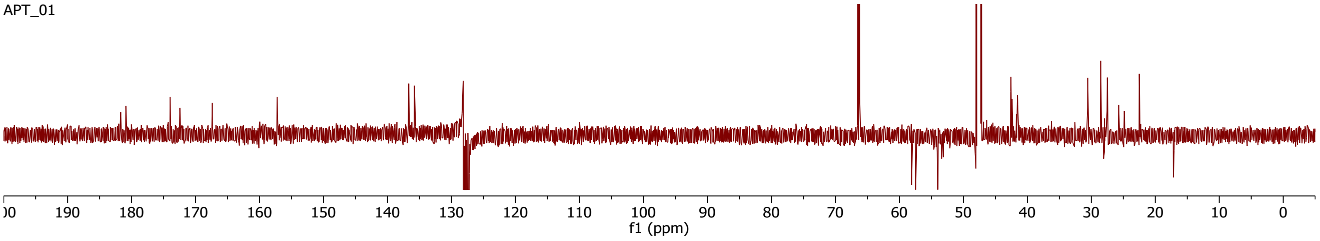
APT_01
f1 (ppm)

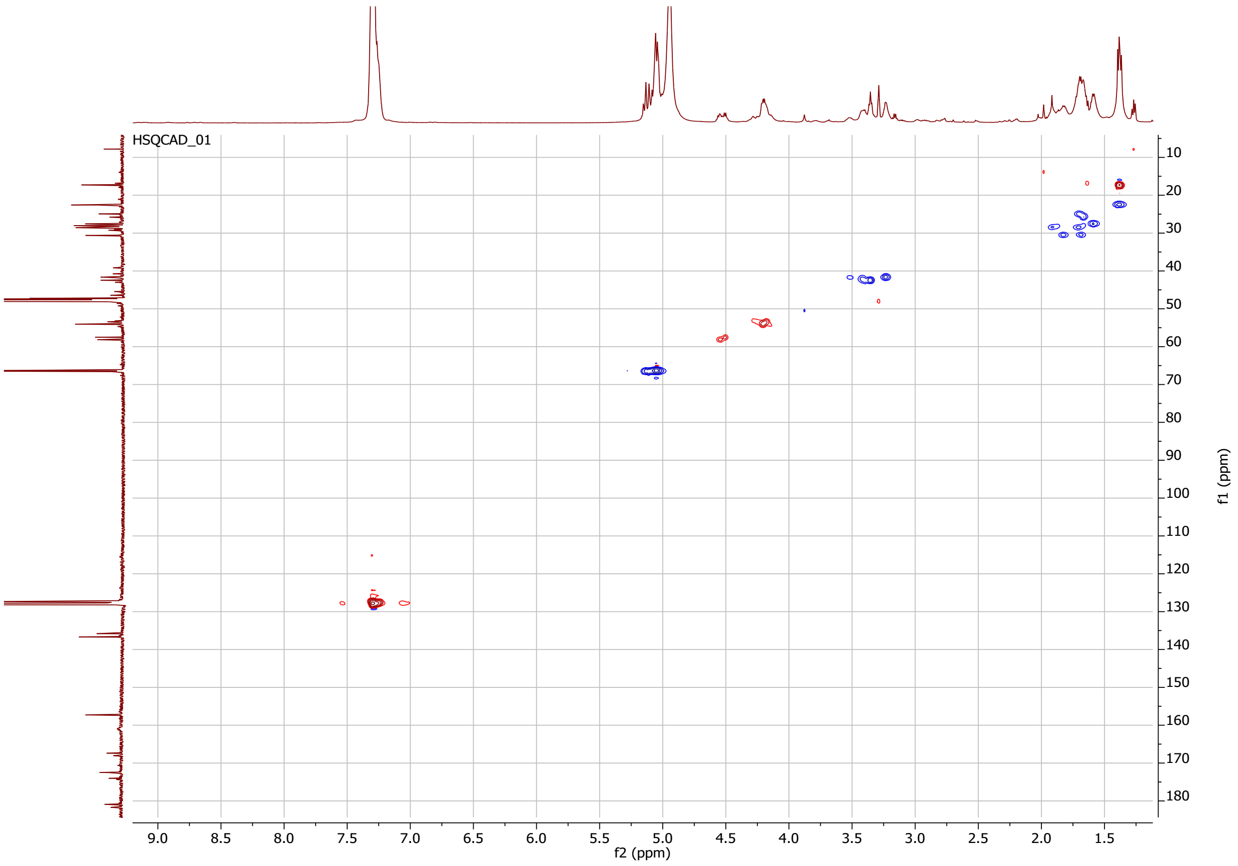
HSQCAD_01
f2 (ppm)
f1 (ppm)

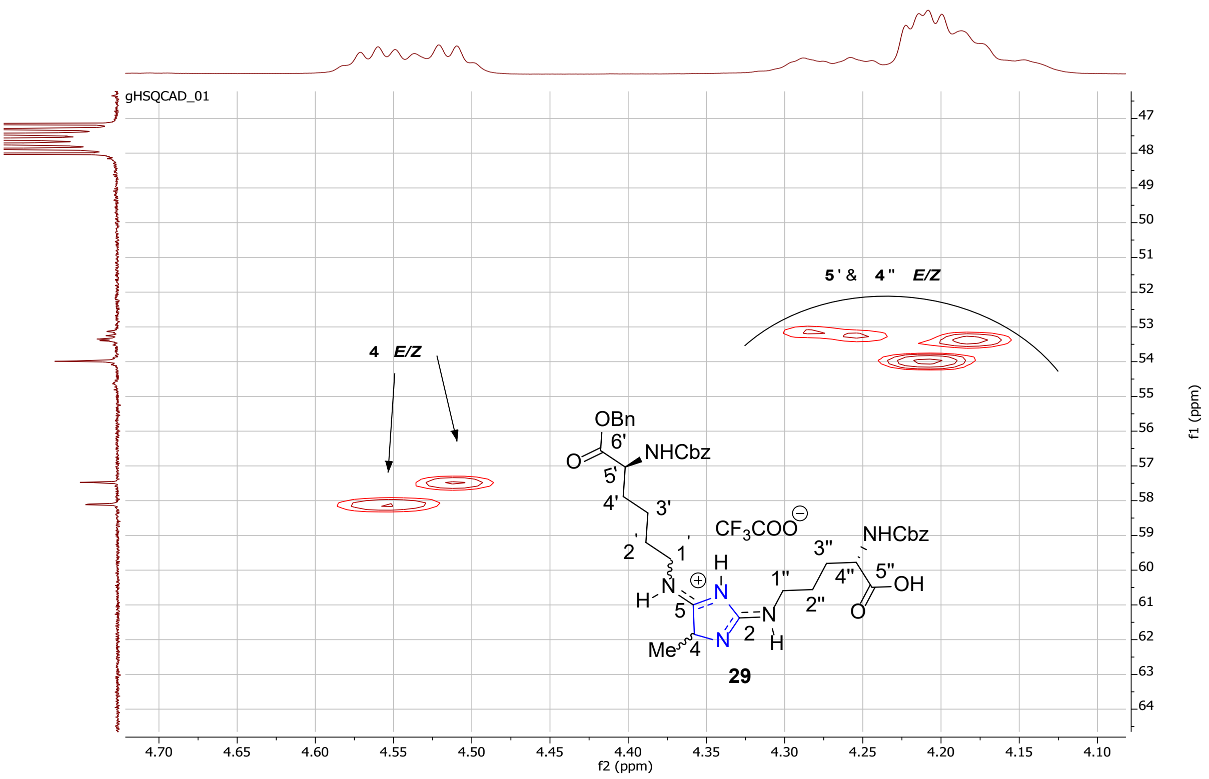
gHSQCAD_01
5'& 4" E/Z
4 E/Z
OBn
6'
NHCbz
5'
4'
3'
2'
1'
CF_3COO^-
NHCbz
3"
H
1"
4"
5"
OH
2"
H
5
2
Me
4
H
29
f1 (ppm)
f2 (ppm)

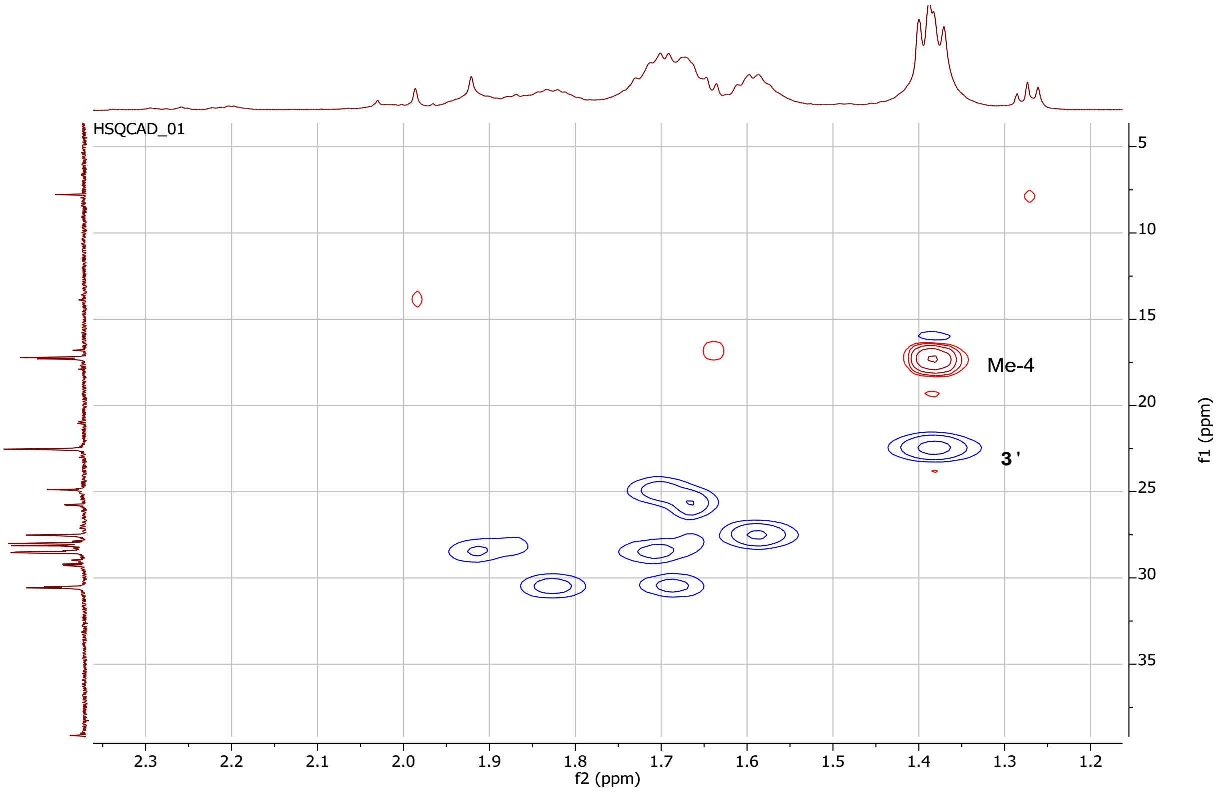
HSQCAD_01
Me-4
3'
f1 (ppm)
f2 (ppm)

MODIC tris-trifluoroacetate salt

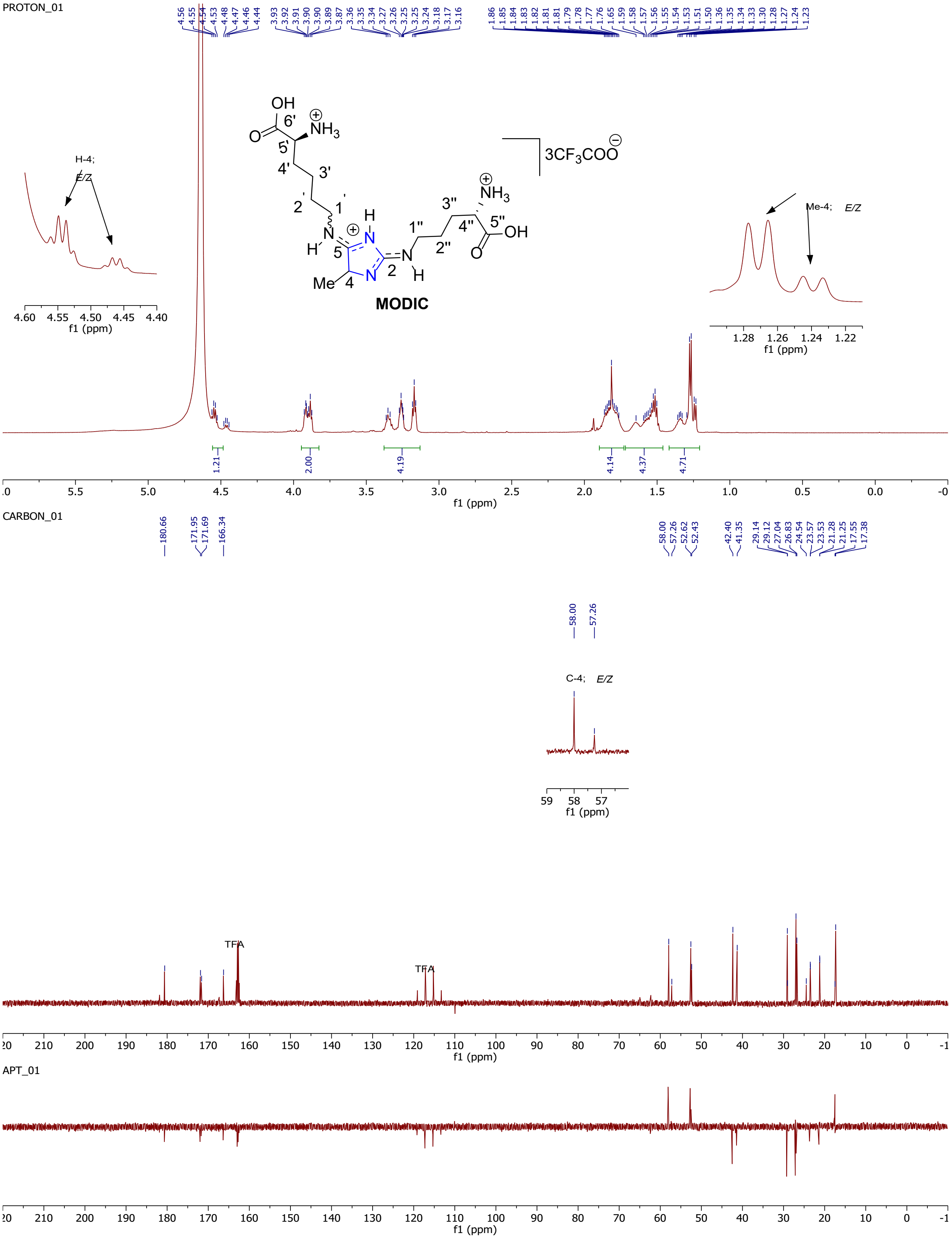

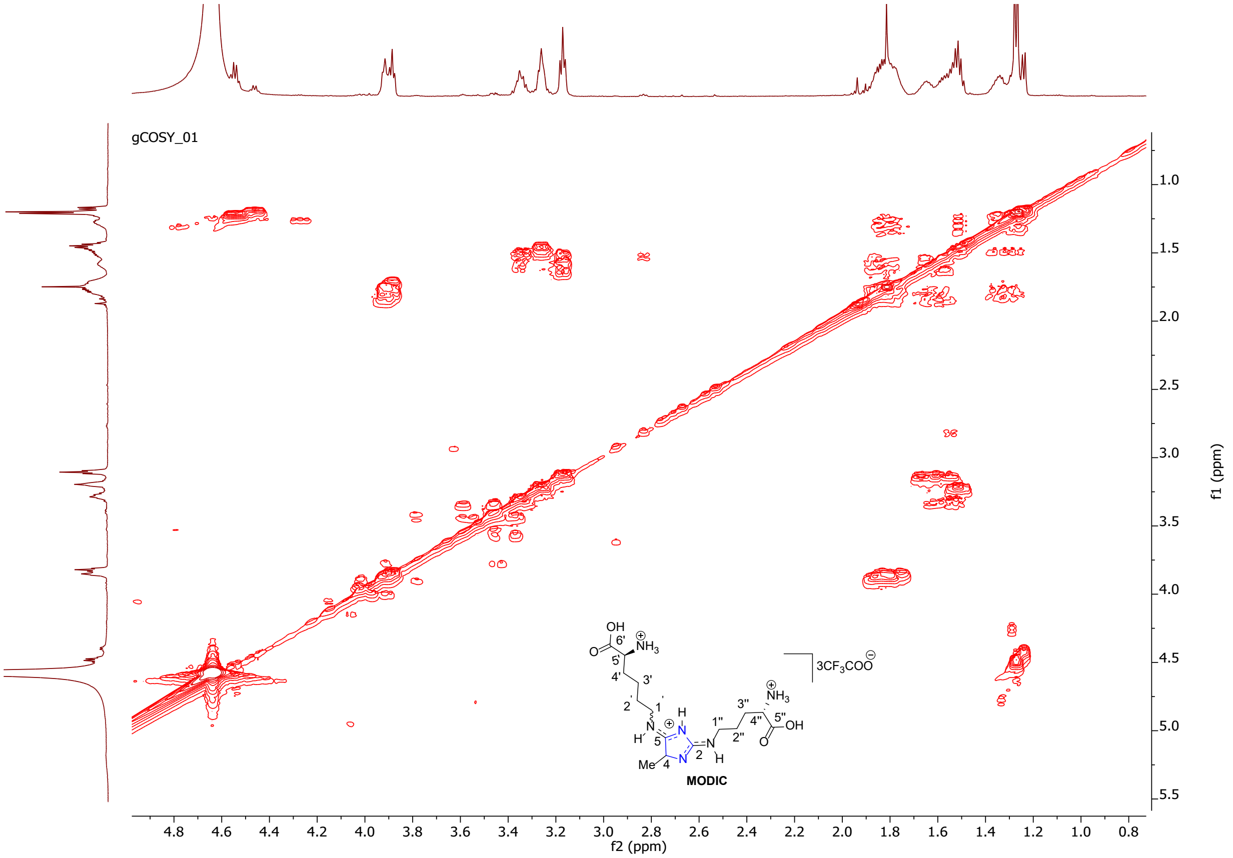
gCOSY_01
f1 (ppm)
f2 (ppm)
MODIC
3CF3COO

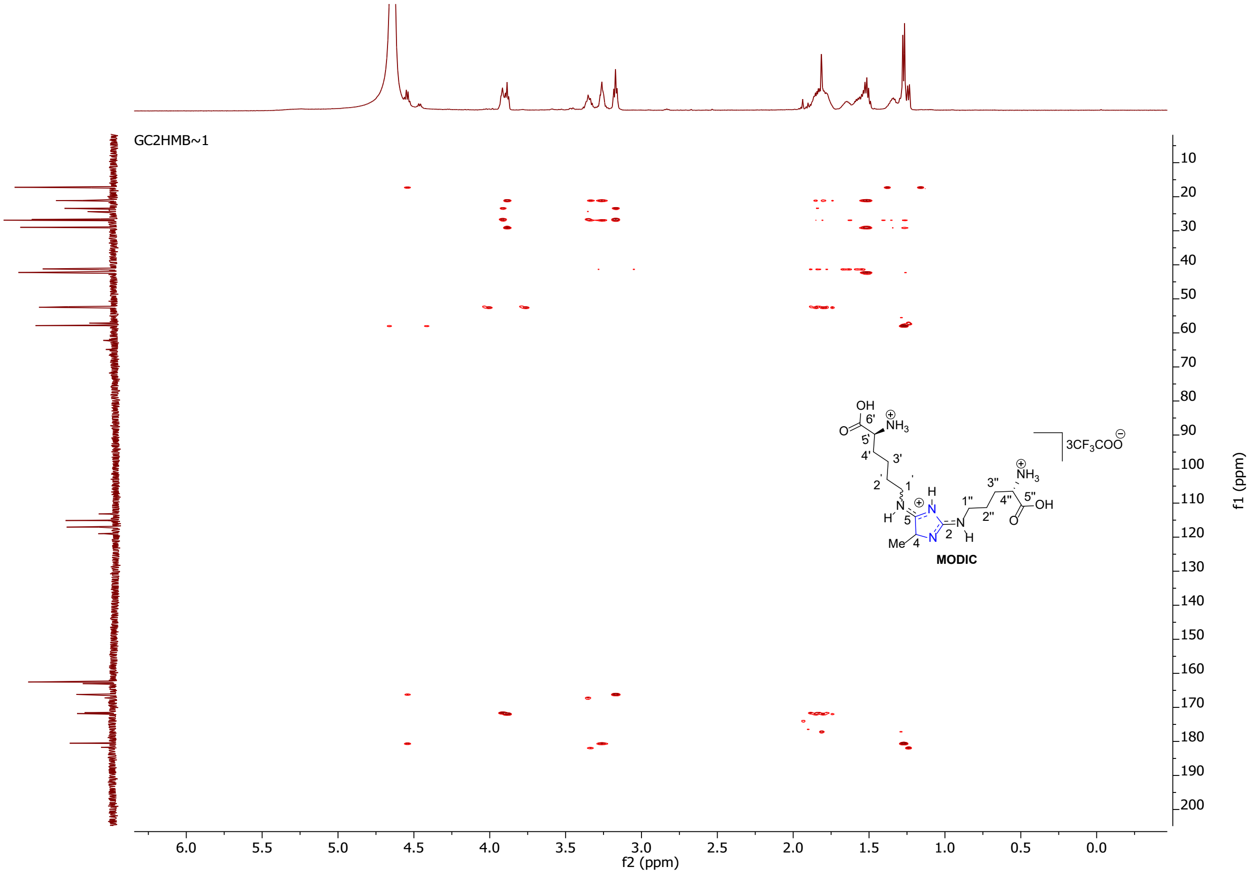
GC2HMB~1
f1 (ppm)
f2 (ppm)
MODIC
3CF3COO

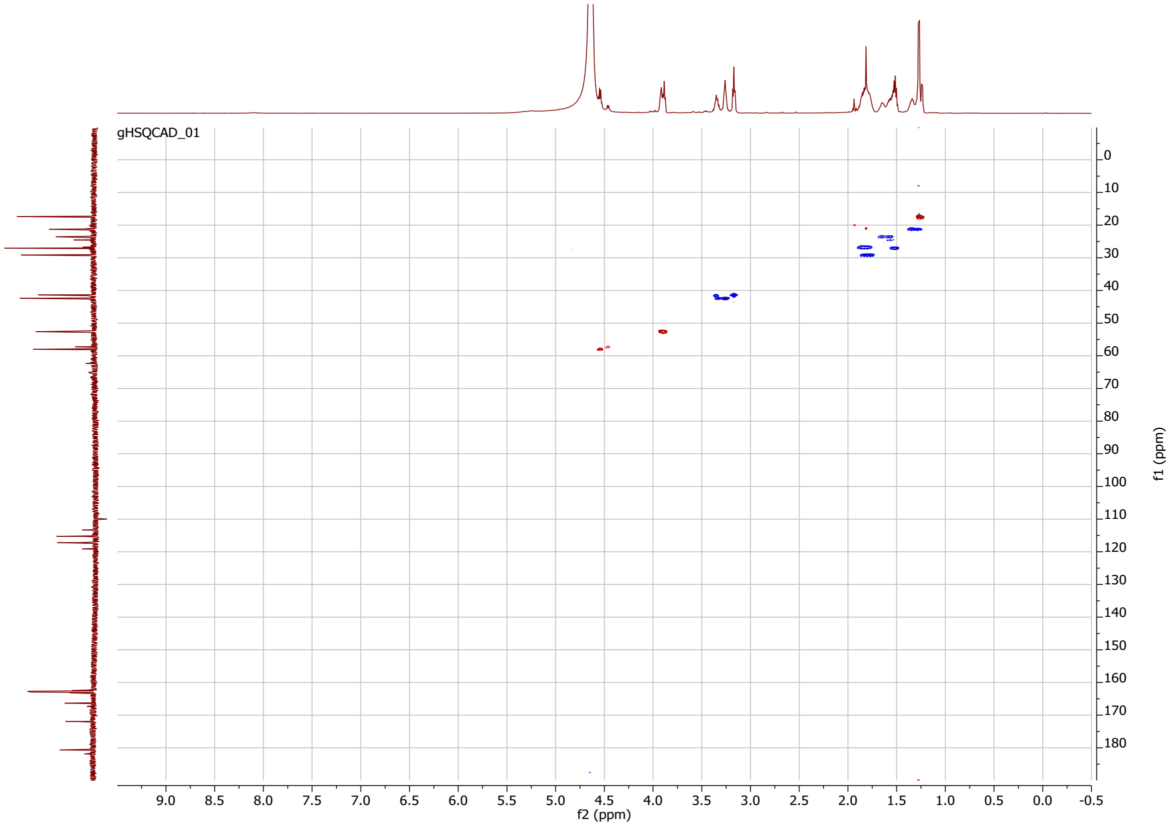
gHSQCAD_01
f1 (ppm)
f2 (ppm)

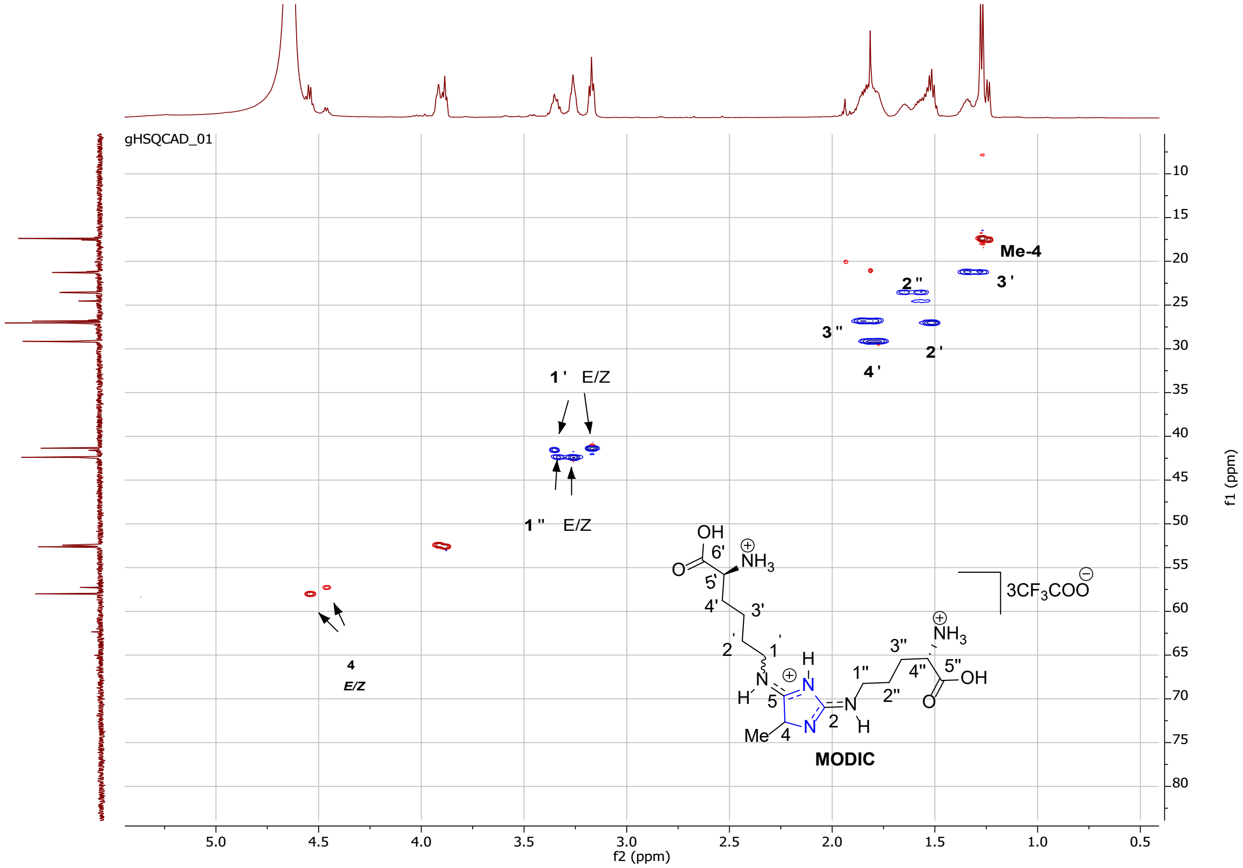
gHSQCAD_01
Me-4
3'
2"
3"
2'
4'
1' E/Z
1" E/Z
4
E/Z
MODIC
3CF3COO
f1 (ppm)
f2 (ppm)

MODIC tris-formate salt

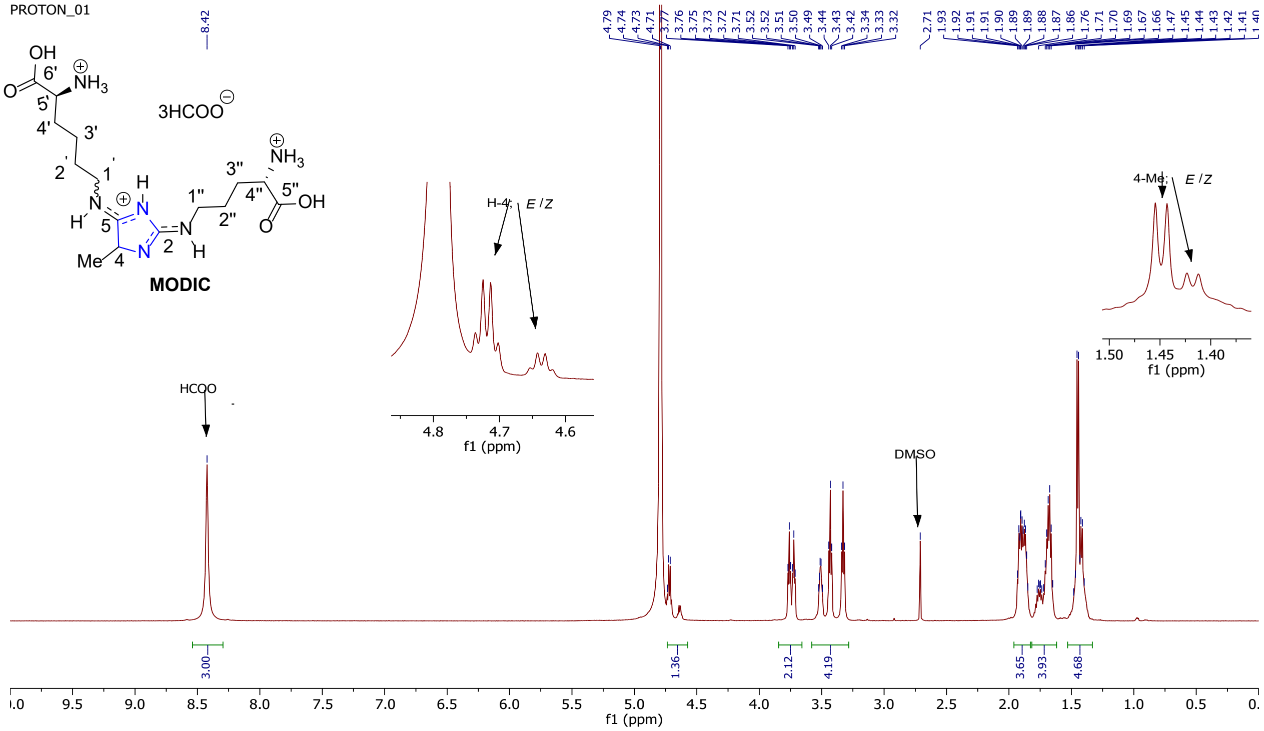

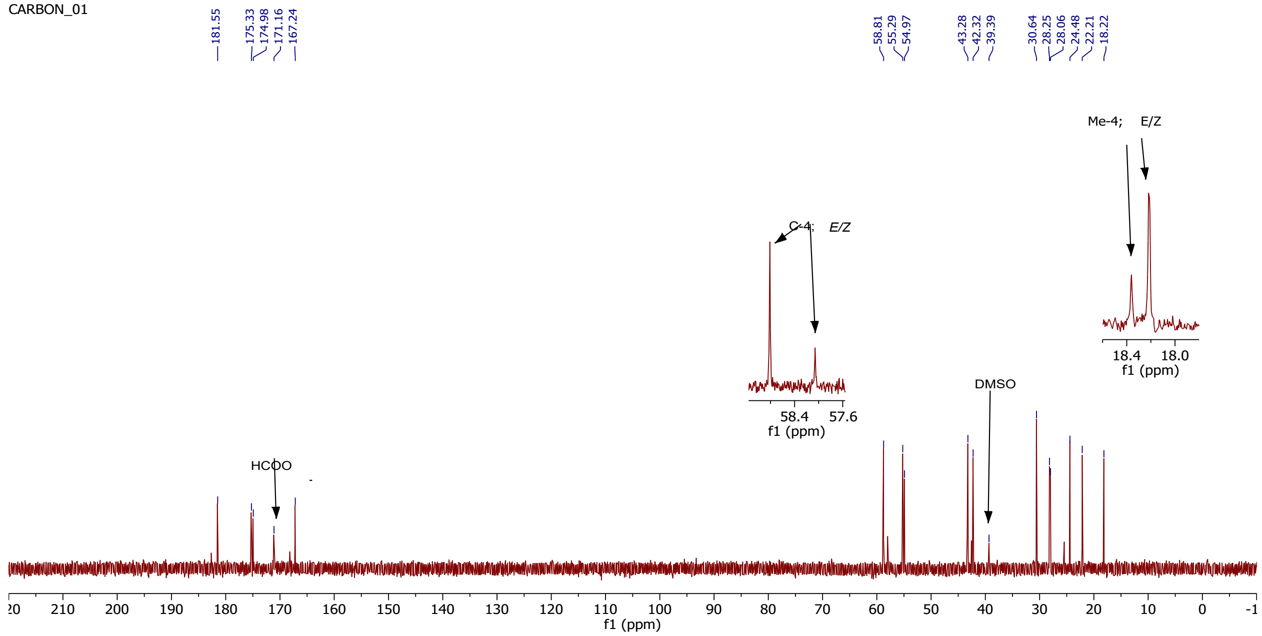

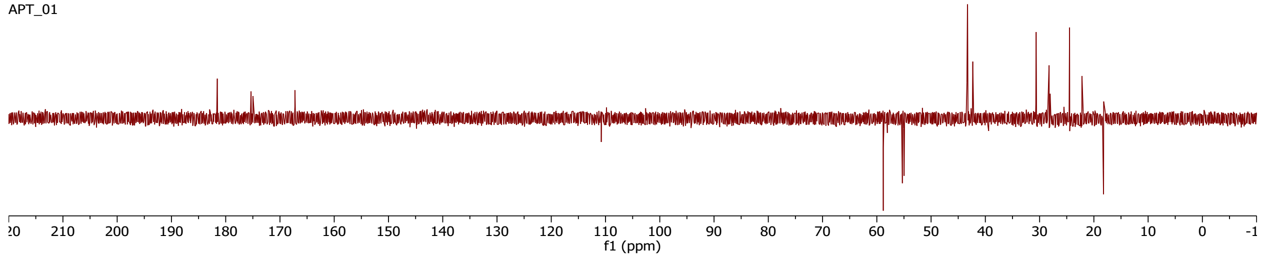

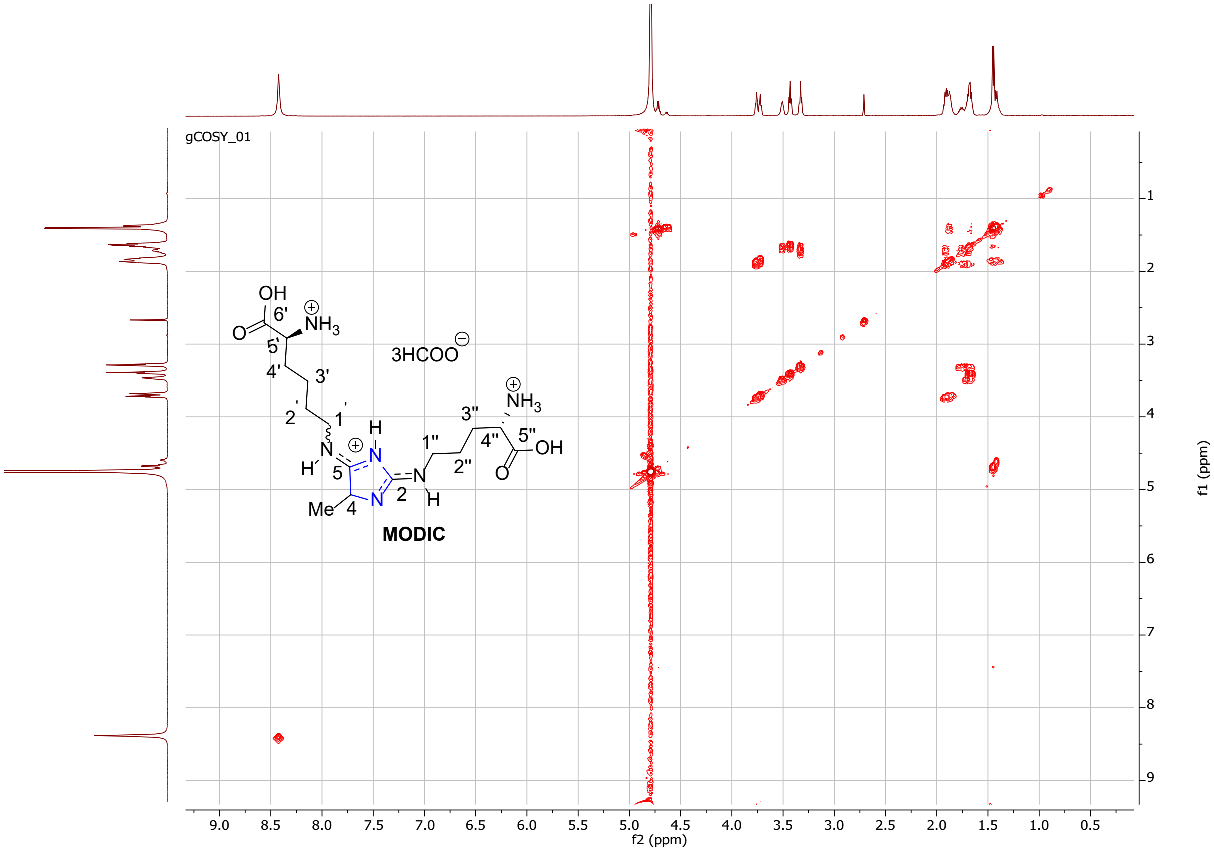
gCOSY_01
MODIC
3HCOO
OH
NH3
Me
f2 (ppm)
f1 (ppm)
9.0 8.5 8.0 7.5 7.0 6.5 6.0 5.5 5.0 4.5 4.0 3.5 3.0 2.5 2.0 1.5 1.0 0.5
1 2 3 4 5 6 7 8 9

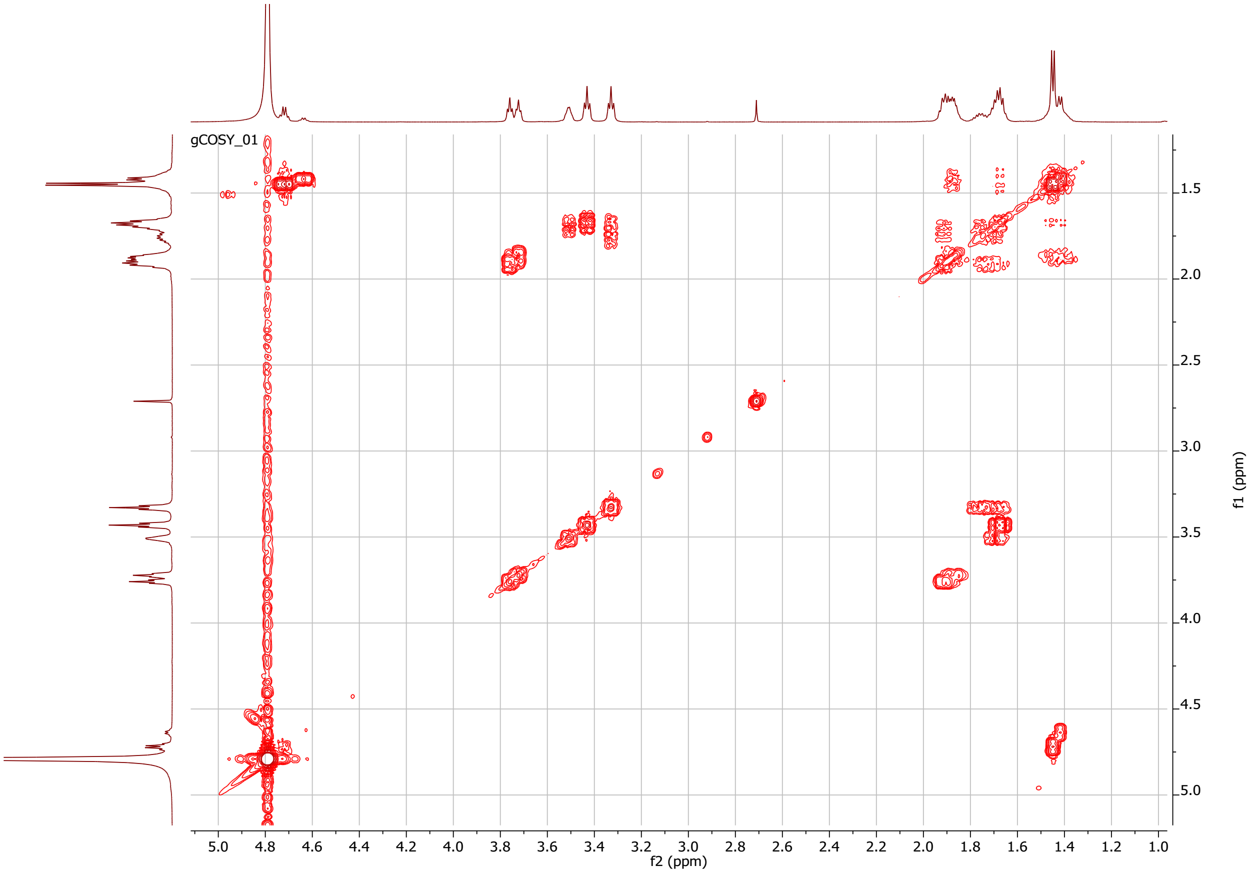
gCOSY_01
f2 (ppm)
f1 (ppm)
5.0 4.8 4.6 4.4 4.2 4.0 3.8 3.6 3.4 3.2 3.0 2.8 2.6 2.4 2.2 2.0 1.8 1.6 1.4 1.2 1.0
1.5 2.0 2.5 3.0 3.5 4.0 4.5 5.0

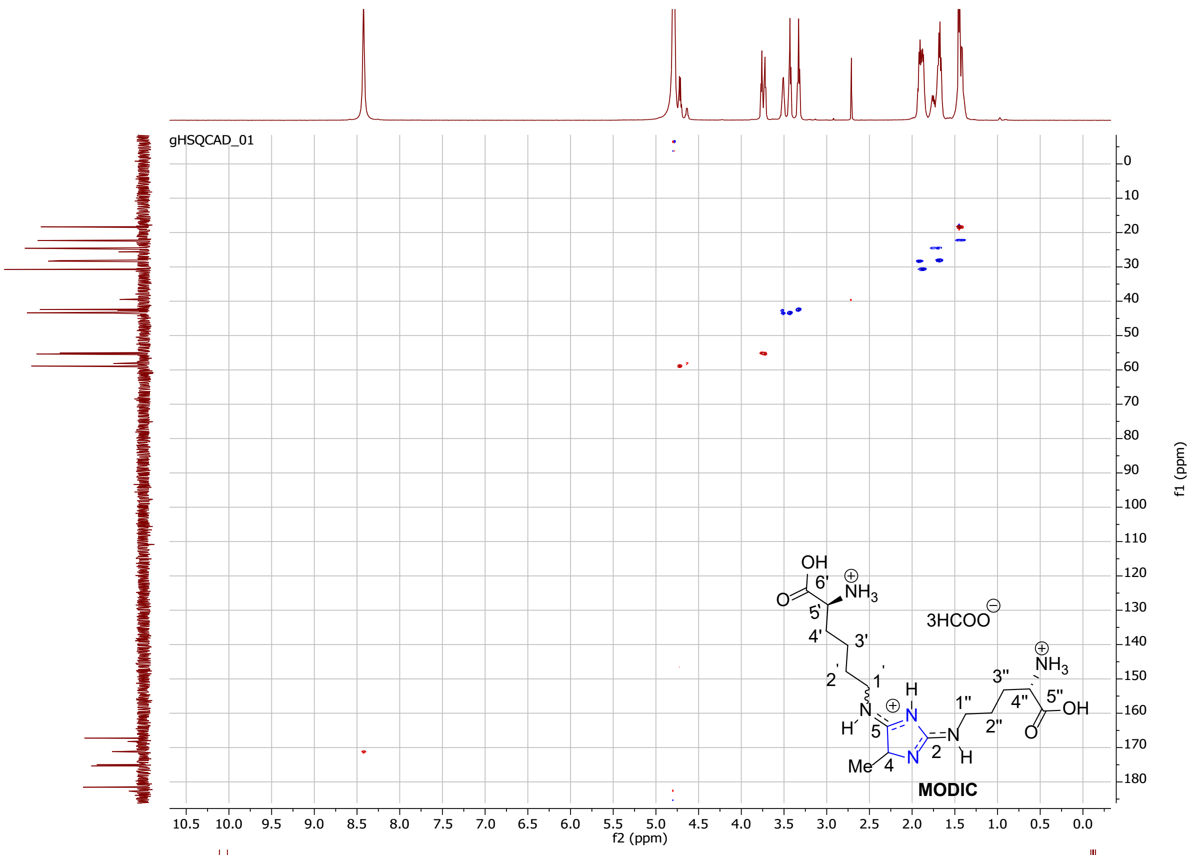
gHSQCAD_01
f2 (ppm)
f1 (ppm)
3HCOO
MODIC
Me
OH
NH3

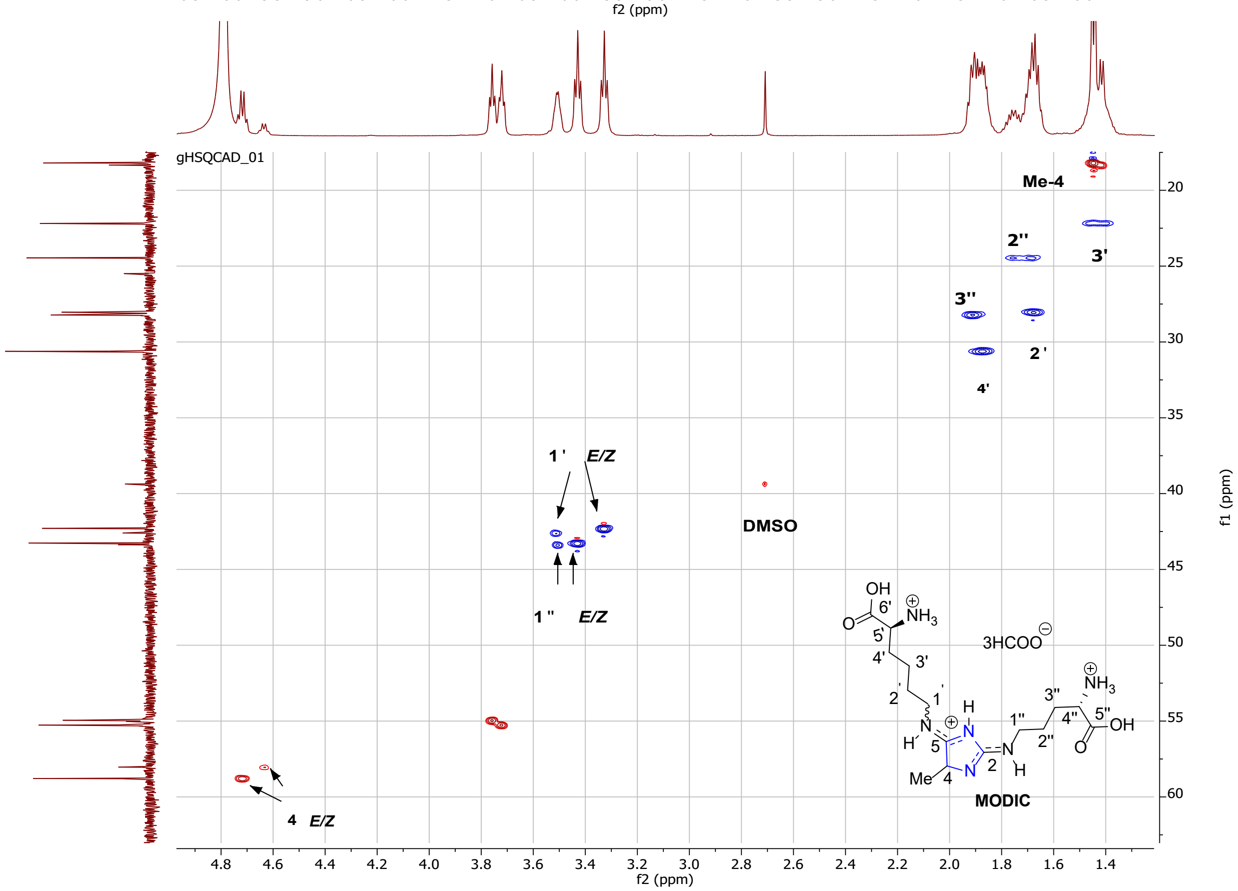
gHSQCAD_01
Me-4
2''
3'
3''
2 '
4'
1 '
E/Z
1 ''
E/Z
DMSO
4 E/Z
3HCOO
MODIC
f2 (ppm)
f1 (ppm)
